# Lengthening the Guanidine–Aryl Linker of Phenylpyrimidinylguanidines Increases Their Potency as Inhibitors of FOXO3-Induced Gene Transcription

**DOI:** 10.1021/acsomega.2c04613

**Published:** 2022-09-14

**Authors:** Klara Kohoutova, Vojtěch Dočekal, Michael J. Ausserlechner, Nora Kaiser, Andrej Tekel, Raju Mandal, Matej Horvath, Veronika Obsilova, Jan Vesely, Judith Hagenbuchner, Tomas Obsil

**Affiliations:** † Department of Physical and Macromolecular Chemistry, Faculty of Science, Charles University, Albertov 6, Prague 12843, Czech Republic; ‡ Institute of Physiology of the Czech Academy of Sciences, Laboratory of Structural Biology of Signaling Proteins, Division BIOCEV, Prumyslova 595, Vestec 25250, Czech Republic; § Department of Organic Chemistry, Faculty of Science, Charles University, Albertov 6, Prague 12843, Czech Republic; ∥ Department of Pediatrics I, Medical University Innsbruck, Innrain 66, Innsbruck 6020, Austria; ⊥ Department of Pediatrics II, Medical University Innsbruck, Innrain 66, Innsbruck 6020, Austria

## Abstract

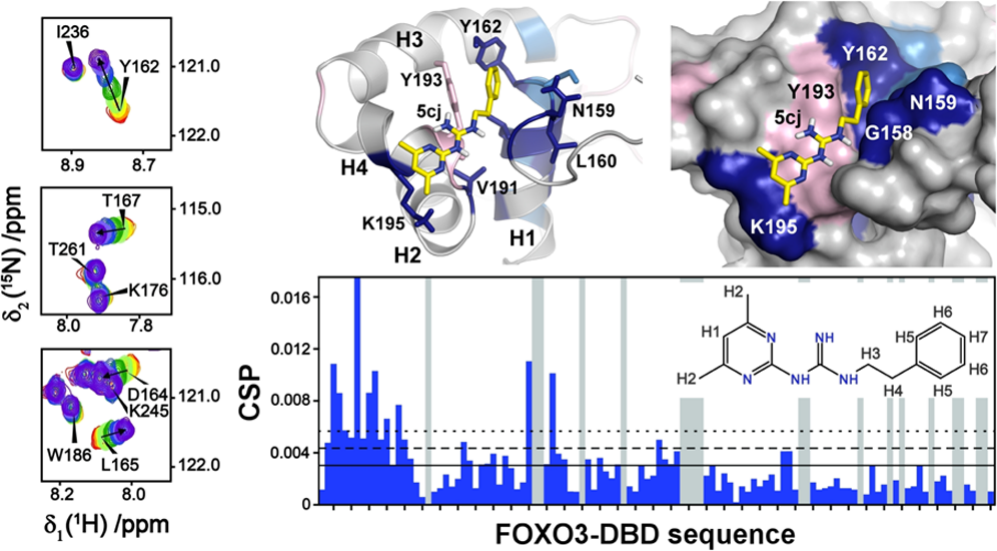

Increased FOXO3 nuclear localization is involved in neuroblastoma chemoresistance and tumor angiogenesis. Accordingly, FOXO3 inhibition is a promising strategy for boosting antitumor immune responses and suppressing FOXO3-mediated therapy resistance in cancer cells. However, no FOXO3 inhibitors are currently available for clinical use. Nevertheless, we have recently identified (4-propoxy)phenylpyrimidinylguanidine as a FOXO3 inhibitor in cancer cells in the low micromolar range. Here, we report the synthesis and structure–activity relationship study of a small library of its derivatives, some of which inhibit FOXO3-induced gene transcription in cancer cells in a submicromolar range and are thus 1 order of magnitude more potent than their parent compound. By NMR and molecular docking, we showed that these compounds differ in their interactions with the DNA-binding domain of FOXO3. These results may provide a foundation for further optimizing (4-propoxy)phenylpyrimidinylguanidine and developing therapeutics for inhibiting the activity of forkhead box (FOX) transcription factors and their interactions with other binding partners.

## Introduction

Forkhead box (FOX) transcription factors display high functional diversity and participate in development, proliferation, differentiation, stress resistance, apoptosis, and metabolic control processes.^1^ This diverse group of transcriptional regulators shares a conserved, 110-amino-acid-long, DNA-binding domain (DBD) known as the Forkhead domain.^2,3^ The O subclass of FOX transcription factors consists of four members, namely, FOXO1, FOXO3, FOXO4, and FOXO6, which are key regulators of cellular homeostasis, longevity, and stress responses.^4−7^

All FOXO proteins recognize the consensus DNA sequence 5′-TTGTTTAC-3′, and their transcriptional activity is negatively regulated by the proproliferative phosphoinositide 3-kinase (PI3K)/protein kinase B (PKB, also known as Akt) signaling pathway.^5,8^ PKB/Akt phosphorylates three Ser/Thr residues and induces the nuclear exclusion of FOXO proteins in a process involving binding to the scaffolding 14-3-3 protein.^9−11^ In addition to phosphorylation by PKB/Akt, FOXO proteins are further regulated through phosphorylation by other kinases, acetylation, ubiquitination, and methylation.^12^ Phosphorylation by stress-induced signaling kinases, such as JNK and MST1, can override the effect of the PKB/Akt-mediated phosphorylation of FOXO3 and induce its nuclear localization and activation, thus promoting FOXO3-triggered therapy resistance in cancer cells.^13−15^

In cancer, FOXO3 stands out for its dual role. On the one hand, FOXO3 induces cell cycle arrest and apoptosis, thus functioning as a typical tumor suppressor.^16^ On the other hand, FOXO3 activity can also promote tumor development and progression by inducing drug resistance,^7,17^ cyclin transcription upregulation,^18^ cellular detoxification,^19,20^ and cancer stem cell maintenance^13^ and by inhibiting apoptosis inducers such as the transcription factor p53.^14^ Moreover, all FOXO proteins also regulate T cell differentiation, especially the pathway that leads to the development and function of regulatory T cells.^21,22^ Therefore, pharmacological inhibition of FOXO3 transcriptional activity is considered a highly promising approach to boosting antitumor immune responses and suppressing FOXO3-mediated therapy resistance in cancer cells.

**Figure 1:**
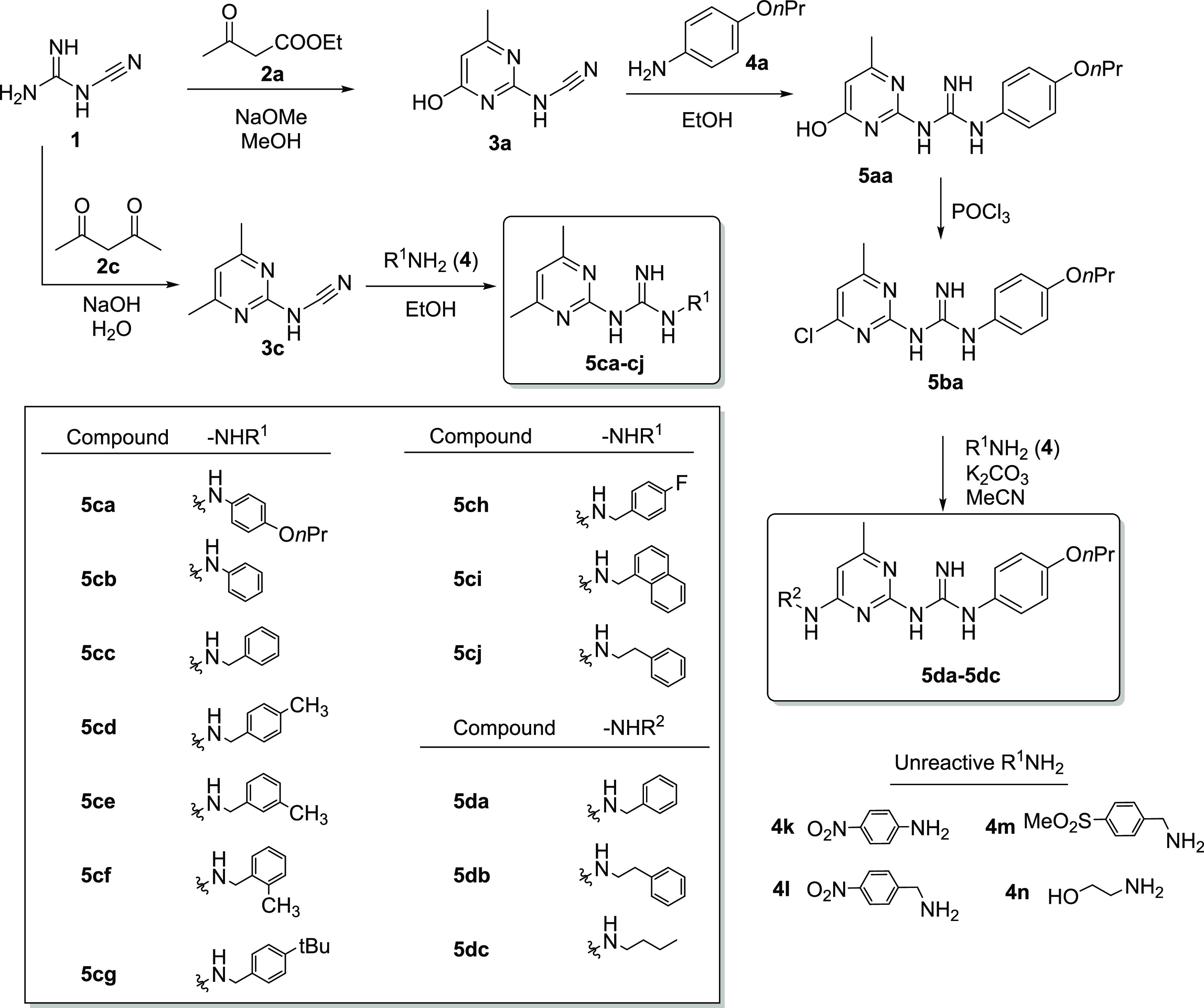
General synthetic route to arylpyrimidylquanidines **5**.

Strong chemotherapy resistance often hampers the treatment of neuroblastoma, a malignancy more prevalent among patients younger than 15 years.^23^ As in other cancers, one of the factors responsible for increased neuroblastoma chemoresistance and tumor angiogenesis is FOXO3 nuclear localization.^14,15^ However, no small-molecule FOXO3 inhibitors are currently available for clinical use. Nevertheless, we have recently identified three FOXO3 inhibitors, namely, carbenoxolone (a glycyrrhetinic acid derivative), repaglinide (an insulin secretagogue belonging to the meglitinide class), and (4-propoxy)phenylpyrimidinylguanidine (**5ca**, Figure 1). These compounds inhibit the transcriptional program of FOXO3 and modulate its physiological function in the low micromolar range in cancer cells.^24,25^ By NMR, we have also demonstrated that **5ca** and its oxalate salt directly interact with the DNA-binding domain (DBD) of FOXO3, thereby blocking its DNA-binding surface in the α-helix H3 recognition region and in the N-terminus of β-strand S2.

Here, we describe the synthesis and structure–activity relationship study of a small library of **5ca** derivatives and show that some of these compounds inhibit FOXO3-induced gene transcription with an IC_50_ in the submicromolar range and thus 1 order of magnitude more potent than the parent **5ca**. Our NMR measurements and molecular docking simulations showed that these compounds differ in their interactions with FOXO3-DBD and target various parts of the FOXO3 DNA-binding surface. Combined, these results may serve as a foundation for further optimizing **5ca** and developing therapeutic agents for inhibiting the transcriptional activity of FOXO by interfering with target promotor binding.

## Results

### Synthesis of the Compounds

New bisarylguanidines for structure–activity relationship studies were synthesized by varying the pyrimidine and aromatic moieties of the original compound **5ca**. First, we modified the pyrimidine ring system by introducing a more polar and electron-rich *N*-substituted amino substituent instead of the lipophilic methyl in position 4 on the 4,6-dimethylpyrimidine core. These compounds were synthesized by treating dicyandiamide (**1**) with ethyl acetoacetate (**2a**) under basic conditions followed by the nucleophilic addition of 4-propoxyaniline **4a** (Figure 1 and Table 1).

**Table 1:** Properties of All Compounds Prepared in This Study

compound	molecular formula	molecular weight (g·mol^–1^)	Clog *P*^a^	Clog *D*^a^(at pH 7.4)	solubility in water (mM)^b^
**5aa**	C_15_H_19_N_5_O_2_	301.35	3.03	3.01	0.1
**5ba**	C_15_H_18_ClN_5_O	319.79	3.56	3.55	20
**5ca**	C_16_H_21_N_5_O	299.38	2.87	2.84	2
**5cb**	C_7_H_9_N	107.16	1.45	1.45	4
**5cc**	C_14_H_17_N_5_	255.33	1.97	1.16	10
**5cd**	C_15_H_19_N_5_	269.35	2.48	1.70	7
**5ce**	C_15_H_19_N_5_	269.35	2.48	1.67	4
**5cf**	C_15_H_19_N_5_	269.35	2.48	1.64	2
**5cg**	C_18_H_25_N_5_	311.43	3.51	2.87	0.2
**5ch**	C_14_H_16_FN_5_	273.32	2.11	1.46	6
**5ci**	C_18_H_19_N_5_	305.39	2.96	2.09	0.7
**5cj**	C_15_H_19_N_5_	269.35	2.26	1.28	13
**5da**	C_22_H_26_N_6_O	390.49	4.53	4.50	0.2
**5db**	C_23_H_28_N_6_O	404.52	4.82	4.79	0.3
**5dc**	C_19_H_28_N_6_O	356.47	4.13	4.10	1.7

a Clog *P* and Clog *D* values were estimated using the Chemicalize server (https://chemicalize.com/).

b Solubility was estimated in 20 mM phosphate (pH 6.5) and 50 mM KCl buffer at 23 °C.

The hydroxy group of bisarylquanidine **5aa** was directly converted into chlorine using a standard chlorination procedure.^26^ Chlorine of **5ba** was subsequently displaced with an *N-*nucleophile in a nucleophilic aromatic substitution reaction, yielding the desired analogues **5da–5dc** in acceptable yields (35–59%). Unfortunately, these compounds showed a significantly decreased solubility in water. For this reason, we continued with the modification of the second part of **5ca**, the aryl substituent.

The key intermediate, **3c**, was prepared by dicyandiamide (**1**) condensation with acetoacetate (**2**) to **3c**.^26^ The following nucleophilic addition of aniline (**4b**) to **3c** afforded compound **5cb** in a low yield (31%). Moreover, **5cb** showed insufficient solubility in water (Table 1). When using, under the same conditions, the more flexible benzylamine (**4c**), the corresponding bisarylguanidine **5cc** was obtained in a moderate yield (46%) and with a significantly increased solubility in water. Based on these results, we aimed to assess this effect using various benzylamines for nucleophilic addition.

Initially, the effect of substitution in the ortho, meta, and para positions of the benzene ring in benzylamine moiety was examined. For this purpose, the corresponding products **5cd–5cf** were isolated in high yields (72–80%). The solubility of **5cd**–**5cf** in water decreased in the ortho-substituted derivative, whereas the para-substituted derivative **5cd** exhibited improved solubility, albeit slightly lower than that of **5cc**. The reaction with the more sterically hindered and lipophilic *tert*-butyl in the para-position of benzylamine resulted in the formation of the derivate **5cg** in a high yield (82%). Unsurprisingly, this change of substituent limited the solubility of **5cg** in water.

Our attempts to prepare guanidines substituted with strong electron-deficient benzylamines (**4l** and **4m**) were unsuccessful. Only with 4-fluorobenzylamine was the corresponding guanidine **5ch** prepared in good yield (68%), also showing sufficient solubility in water. The following modifications, that is, extending the aromatic moiety and linker between guanidine and benzene moiety, led to **5ci** and **5cj**. These compounds were prepared using a standard procedure in high yields (61 and 82%, respectively). Bisarylguanidine **5cj** was identified as the most water-soluble compound.

### Biological Activity and Toxicity

The FOXO3 inhibition potency of the phenylpyrimidinylguanidine derivatives was first examined in a fluorescence polarization (FP) assay using the recombinant DNA-binding domain of human FOXO3 (residues 156–269, denoted as FOXO3-DBD) and FAM-labeled dsDNA containing the insulin-response element (IRE) consensus motif (Figure S1). As noted, all compounds tested at a concentration of 1 μM reduced FOXO3-DBD binding to FAM-IRE dsDNA by approximately 20%, similarly to the original **5ca**, thus suggesting similar binding affinities in a micromolar range.

The ability of these compounds to inhibit FOXO3 transcriptional activity was further examined by assessing their effect on FOXO3 binding to the promoter region of decidual protein induced by progesterone 1 (DEPP1) after transfecting a DEPP1-luciferase reporter plasmid into SH-EP neuroblastoma cells stably expressing a 4-hydroxy-tamoxifen (4OHT)-regulated FOXO3(A3)ERtm transgene. DEPP1 modulates autophagy in a ROS-dependent manner and is strongly induced by FOXO3 via three functional FOXO consensus sequences in its promoter.^20,27^ In parallel, the toxicity of the compounds was tested by assessing their effect on endogenous DEPP1 and actin promoters in SH-EP cells.

**Figure 2:**
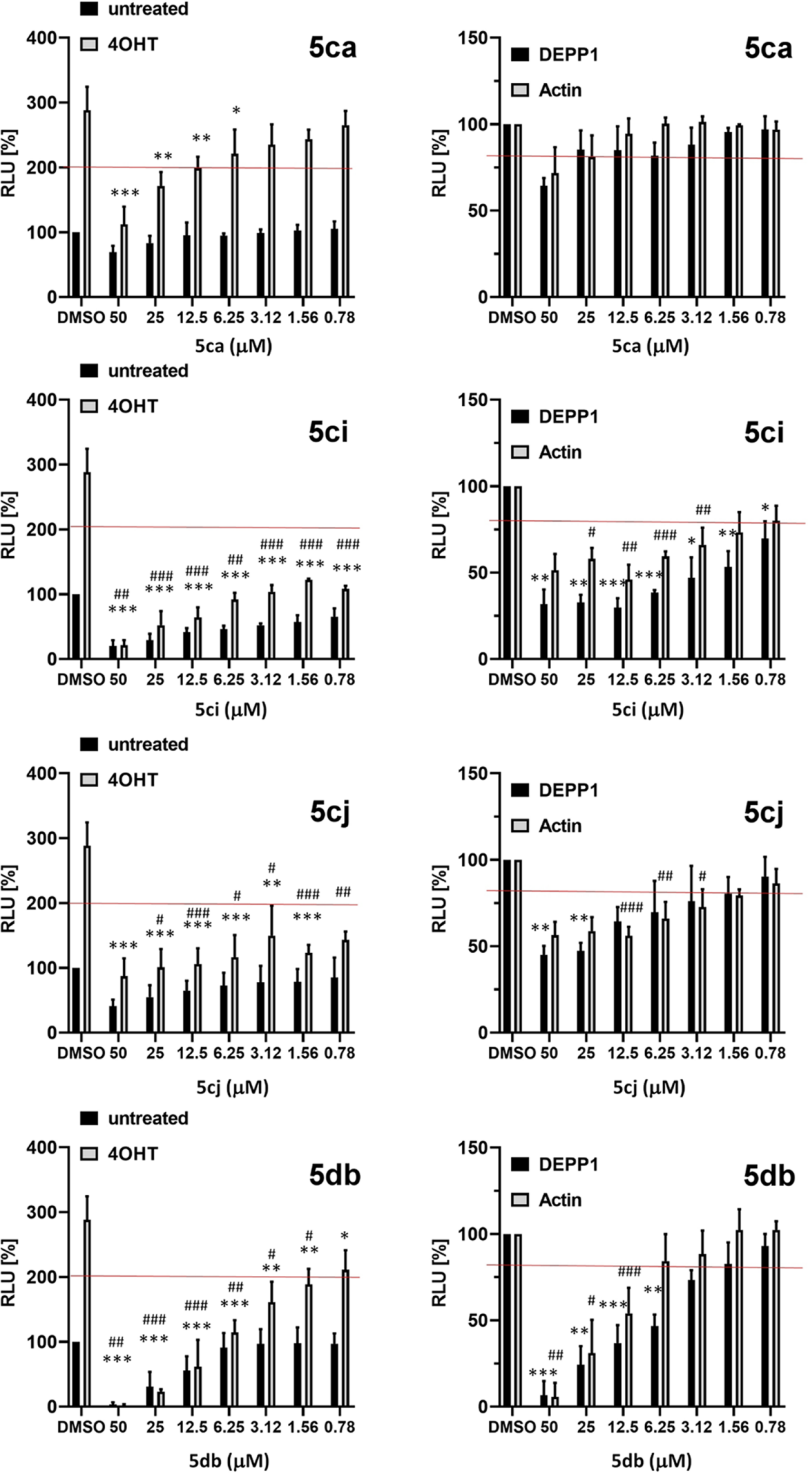
Effects of **5ca** and its derivatives on target gene activation by FOXO3. SH-EP/FOXO3 cells transfected with either DEPP1-luciferase reporter plasmid (DEPP1) or β-actin-luciferase reporter (actin) (as toxicity control) were treated with **5ca** derivatives **5ca**, **5ci**, **5cj**, and **5db** before adding 4-hydroxy-tamoxifen (4OHT) to activate ectopic FOXO3. The increase of firefly-luciferase activity/light emission was calculated as the percentage of dimethyl sulfoxide (DMSO)-only control. The values are expressed as the mean ± standard deviation (SD) of three independent experiments, each of which was performed in triplicate. The red line indicates the efficacy of **5ca** in inhibiting FOXO3-induced relative light unit (RLU) at 12.5 μM. Significant differences between 4OHT treatment and substance + 4OHT: *p < 0.05, **p < 0.01, ***p < 0.001; significant differences between **5ca** treatment and new derivatives at the same concentration: #*p* < 0.05, ##*p* < 0.01, ###*p* < 0.001 (Student *t*-test).

The initial screens performed with 50 μM compounds suggested that **5ba**, **5cg**, **5ci**, **5da**, **5db**, and **5dc** are more potent than **5ca** in blocking FOXO3 binding to the promoter region of DEPP1 (Figure S2). Although the effect of **5cd**, **5cf**, and **5cj** was similar to that of **5ca**, compounds **5cb**, **5cc**, **5ce**, and **5ch** were less potent FOXO3 inhibitors than **5ca**. Further experiments at concentrations of 25 and 12.5 μM revealed that **5cg**, **5ci**, **5cj**, **5da**, **5db**, and **5dc** were the most potent compounds among the set of **5ca** derivatives (Figure 2). However, all of these compounds exhibited significant toxicity at the concentrations tested (Figure S2). Therefore, the inhibitory assay was performed at lower concentrations, down to 0.78 μM (Figures 2 and S3).

Compounds **5ci**, **5cj**, and **5db** blocked FOXO3 binding to the promoter region of DEPP1 more efficiently than **5cg**, **5da**, and **5dc**. Only compounds **5ci** and **5cj** significantly inhibited FOXO3-mediated transcription at the lowest concentration tested in this study (0.78 μM) without any effect on endogenous DEPP1 and actin promoters in SH-EP cells as toxicity markers. Moreover, we also attempted to determine the IC_50_ values of **5ci**, **5cj**, and **5db** using a resazurin early cell death evaluation assay in cells expressing a conditional FOXO3 allele that triggers apoptotic cell death upon activation.

As shown in Figure S4, FOXO3-induced apoptosis was inhibited in a concentration-dependent manner by different compounds. The toxicity of these compounds at concentrations higher than 4 μM in **5ci**, **5cj**, and **5db** and higher than 25 μM in **5ca** prevented us from accurately determining their IC_50_ values. Nevertheless, the data suggest that compounds **5ci** and **5db** have similar IC_50_ values of ∼0.5 μM and that the IC_50_ of derivative **5cj** is ∼ 1 μM and thus approximately 8–16× lower than that of **5ca** (IC_50_ of ∼8 μM; Figure S4). Combined, these data suggest that derivatives **5ci**, **5cj**, and **5db** are significantly more potent than **5ca** in inhibiting FOXO3 transcriptional function in cancer cells.

### Mapping of Interactions between FOXO3-DBD and Selected Compounds

Because no successful crystallization of apo FOXO-DBD has been reported so far (all available apo FOXO-DBD structures are solution structures), we characterized the interaction between FOXO3-DBD and compounds **5ci**, **5cj**, **5db**, and **5dc** by NMR spectroscopy. Our results showed a high potency in blocking the transcriptional function of FOXO3 and sufficient solubility in water (Figures 2 and S3 and Table 1). A similar approach was used in our previous study, wherein we characterized interactions between FOXO3-DBD and bisarylguanidine **5ca**.^25^

**Figure 3:**
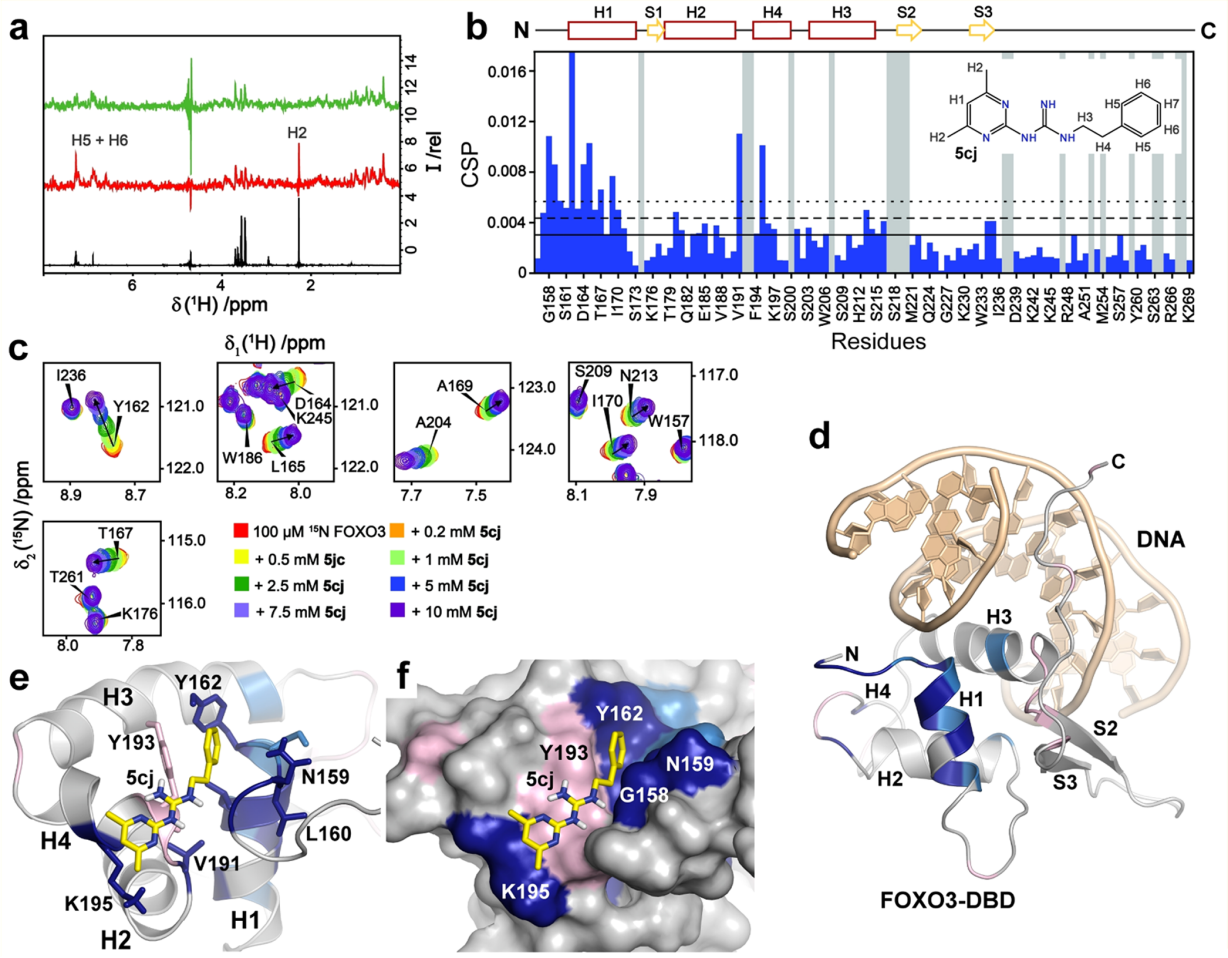
Interaction between FOXO3-DBD and compound **5cj**. (a) ^1^H STD NMR experiment. The black line corresponds to the proton spectrum of 15 μM FOXO3-DBD in the presence of 1 mM **5cj**. The green line corresponds to the ^1^H STD spectrum of 15 μM FOXO3-DBD without **5cj**. The red line corresponds to the ^1^H STD spectrum of 15 μM FOXO3-DBD in the presence of 1 mM **5cj**. (b) Distribution of CSPs observed in residues of 100 μM ^15^N-labeled FOXO3-DBD in the presence of 500 μM **5cj**. Solid, dashed, and dotted lines correspond to the mean, mean + 1σ_cor_^0^, and mean + 2σ_corr_^0^ values of CSPs, respectively. Gray bars represent unassigned residues in ^1^H–^15^N HSQC spectra. The secondary structure of FOXO3-DBD is indicated at the top. The structure of **5cj** is shown in the inset, and equivalent protons visible in the ^1^H spectrum are numbered from 1 to 7. (c) Detailed view of selected peaks of ^1^H–^15^N HSQC spectra of ^15^N-labeled FOXO3-DBD in **5cj**. (d) CSPs of ^15^N-labeled FOXO3-DBD in **5cj** mapped onto the crystal structure of the FOXO3-DBD:DNA complex.^28^ Residues with CSPs larger than the mean + 2σ_corr_^0^ and the mean + 1σ_corr_^0^ (from panel b) are highlighted in dark blue and light blue, respectively. The residues that could not be unambiguously assigned are highlighted in light pink. (e, f) Top-ranked HADDOCK model of the FOXO3-DBD:**5cj** complex; FOXO3-DBD is shown in either ribbon or surface representation. Residues located in close proximity to **5cj** are shown as sticks.

Those interactions were first characterized by ^1^H saturation transfer difference (STD) NMR. However, given the low solubility of **5ci, 5db**, and **5dc**, an STD spectrum was successfully recorded only for **5cj** (Figure 3a). STD signals were detected for protons from both aromatic moieties of **5cj**, thus confirming its interaction with FOXO3-DBD. The binding site of **5cj** in FOXO3-DBD was identified by analyzing ^1^H and ^15^N chemical shift perturbations (CSPs) of the backbone amide groups of ^15^N-labeled FOXO3-DBD in ^1^H–^15^N HSQC spectrum in the presence of 0.5 mM **5cj** (Figures 3b,c and S5). The most significant CSPs (the chemical shift change was greater than 2σ_corr_^0^ above the mean) were observed mainly in residues located within the N-terminal extension (W^157^-S^160^), the helix H1 (Y^162^, D^164^, L^165^, T^167^, A^169^), the loop between H2 and H4 (V^191^), and the helix H4 (K^195^).

Mapping of significant CSPs onto a solution structure of FOXO3-DBD^29^ revealed a well-defined surface mainly involving the N-terminal tail and the N-terminus of α-helix H1, which likely forms the main region of the binding site of **5cj** in FOXO3-DBD. NMR data-driven HADDOCK^30,31^ docking and the solution structure of FOXO3-DBD (PDB ID: 2K86(29)) were used to predict a structural model of the FOXO3-DBD:**5cj** complex (Figure 3e,f). Residues with CSPs higher than the mean + 2σ_corr_^0^ were classified as “active”. The top-ranked resulting structures were clustered according to the root-mean-square deviation (RMSD) and ranked by HADDOCK scores.

The top-ranked pose indicated that the phenyl moiety of **5cj** binds into the groove formed by residues G^158^, N^159^, and Y^162^ from the N-terminal segment, whereas the dimethylpyrimidinyl moiety interacts with Y^193^ and K^195^ from helix H4. These FOXO3-DBD regions either directly participate in DNA binding and/or undergo a conformational change upon FOXO3 binding to DNA, thus supporting the hypothesis that **5cj** inhibits FOXO3 by blocking part of its DNA-binding surface.^28,29,32^ Furthermore, the gradual shift in resonances of FOXO3-DBD residues during the titration (Figure 3c) suggests a fast exchange of bound compound on the NMR time scale, in line with the moderate IC_50_ values assessed by the resazurin early cell death evaluation assay (Figure S4).

**Figure 4:**
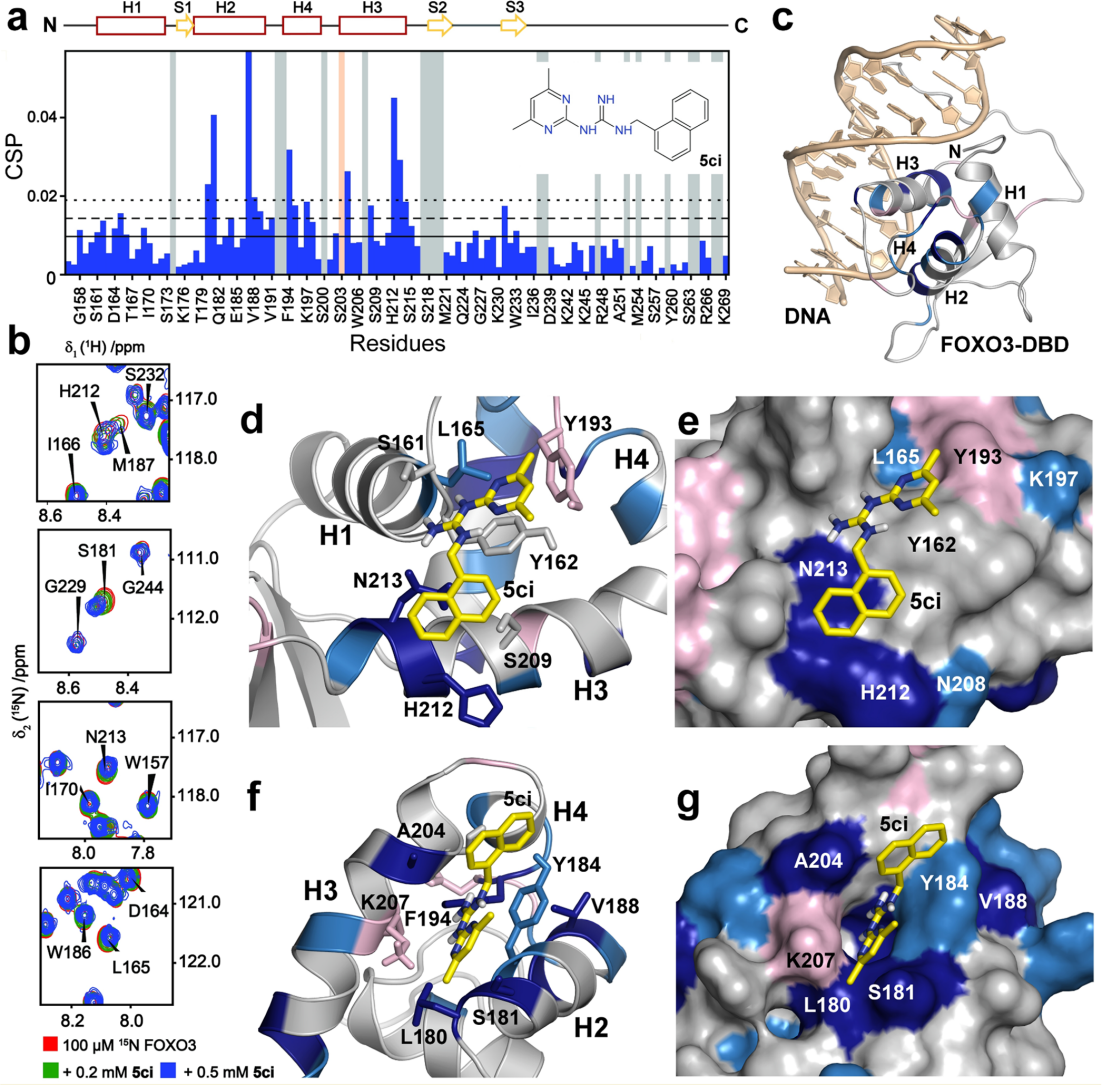
Interaction between FOXO3-DBD and compound **5ci**. (a) Distribution of CSPs observed in residues of 100 μM ^15^N-labeled FOXO3-DBD in the presence of 500 μM **5ci**. Solid, dashed, and dotted lines correspond to the mean, mean + 1σ_corr_^0^, and mean + 2σ_corr_^0^ values of CSPs, respectively. Gray bars represent unassigned residues in ^1^H–^15^N HSQC spectra. The salmon bar corresponds to a residue whose intensity was lost when adding **5ci**. The secondary structure of FOXO3-DBD is indicated at the top. The structure of **5ci** is shown in the inset. (b) Detailed view of selected peaks of ^1^H–^15^N HSQC spectra of ^15^N-labeled FOXO3-DBD in **5ci**. (c) CSPs of ^15^N-labeled FOXO3-DBD in **5ci** mapped onto the crystal structure of the FOXO3-DBD:DNA complex.^28^ Residues with CSPs larger than mean + 2σ_corr_^0^ and mean + 1σ_corr_^0^ (from panel a) are highlighted in dark blue and light blue, respectively. Residues that could not be unambiguously assigned are highlighted in light pink. (d–g) HADDOCK models of the FOXO3-DBD:**5ci** complex from two top-ranked clusters; FOXO3-DBD is shown in either ribbon or surface representation. Residues located near **5ci** are shown as sticks.

A more scattered pattern of CSPs in residues of ^15^N-labeled FOXO3-DBD was observed in 0.5 mM **5ci** (Figure 4a,b). Accordingly, this compound interacts with the FOXO3-DBD surface differently from the others. The most significant CSPs (CSPs higher than the mean + 2σ_corr_^0^) were observed for helices H2 (L^180^, S^181^, M^187^, V^188^), H3 (H^212^, N^213^), and H4 (F^194^, A^204^) residues (Figures 4c and S6). The top-ranked HADDOCK pose predicted that **5ci** binds to the same region of FOXO3-DBD formed by the N-terminus of α-helix H1 and helices H3 and H4, albeit closer to helix H3 than **5cj**. Although the naphthyl moiety of **5ci** interacts with residues H^212^ and N^213^ of this helix, the dimethylpyrimidinyl moiety contacts helix H1 residues Y^162^ and L^165^ and helix H4 residue Y^193^ (Figure 4d,e). This pose, however, does not fully account for the CSP pattern, suggesting the presence of a second binding site. This CSP pattern can be explained, nevertheless, when also considering a HADDOCK pose from the second top-ranked cluster, which indicates that **5ci** binds to the pocket formed by helix H2 residues L^180^, S^181^, and Y^184^ and H3 helix residues A^204^ and F^194^ (Figure 4f,g).

^1^H–^15^N HSQC measurements with **5db** were performed at only 200 μM due to the very low solubility of this compound in water (Figure S7 and Table 1). The most significant CSPs were observed for helices H2 (S^181^, E^185^), H3 (L^214^), and H4 (F^194^, D^196^, K^197^, S^203^) residues (Figures S7a–c and S8), as in **5ci** (Figure 4a). HADDOCK docking suggested that the pyrimidinylguanidine moiety of **5db** interacts with helix H2 residues S^181^ and E^185^, whereas the phenyl moiety binds to the pocket formed by H3 helix residues S^203^, A^204^, and K^207^ (Figure S7d,e). The (4-propoxy)phenyl moiety on the other end of **5db** is positioned next to the side chain of S^181^. As such, this compound should bind to a region similar to the second binding site of **5ci** (Figure 4f,g).

The last compound whose interactions with FOXO3-DBD were mapped was **5dc**. Although this compound had a slightly lower inhibitory efficacy than **5cj**, **5ci**, and **5db** (Figure S3), its solubility in water was higher than that of **5ci** and **5db** (Table 1). ^1^H–^15^N HSQC measurements in the presence of 500 μM **5dc** revealed the highest CSPs in residues of the N-terminal half of helix H1 and, especially, in helix H4 residues located between helices H2 and H3 (Figures S9a–c and S10). The HADDOCK pose predicted that **5dc** binds to the same region as **5cj** with the (4-propoxy)phenyl moiety interacting with helix H1 residue Y^162^, whereas its pyrimidinylguanidine moiety interacts with a surface formed by helix H4 residues Y^193^, K^195^, D^196^, and K^197^ (Figure S9d,e).

Taken together, the results from our detailed characterization indicate different interactions between FOXO3-DBD and **5ci**, **5cj**, **5db**, and **5dc** because these compounds target various parts of the FOXO DNA-binding surface formed by (1) the N-terminal tail, the N-terminus of helix H1 and helix H4 (**5cj** and **5dc**), (2) the N-terminus of helix H1 and the C-terminus of helix H3 (**5ci**), (3) helices H2, H3, and H4 (**5ci**), and (4) the N-terminal halves of helices H2 and H3 (**5db**).

### FOXO-DBDs Differ in Their Interactions with Phenylpyrimidinylguanidines

**Figure 5:**
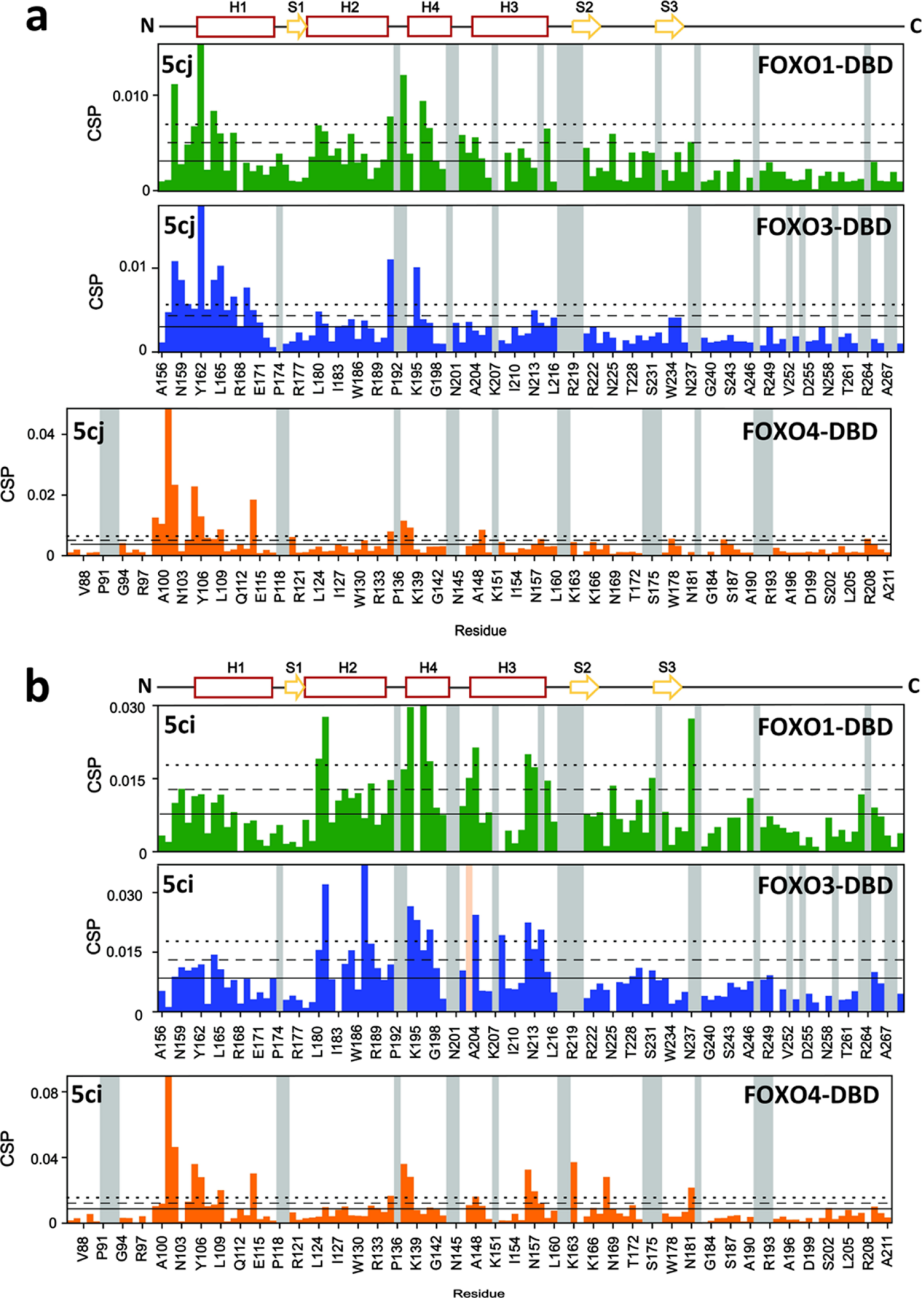
Binding selectivity of compounds **5cj** and **5ci**. Distribution of CSPs observed in residues of 100 μM ^15^N-labeled FOXO-DBDs in the presence of 500 μM **5cj** (a) and 400 μM **5ci** (b). Solid, dashed, and dotted lines correspond to the mean, mean + 1σ_corr_^0^, and mean + 2σ_corr_^0^ values of CSPs, respectively. Gray bars represent unassigned residues in ^1^H–^15^N HSQC spectra. The salmon bar corresponds to a residue whose intensity was lost when adding **5ci**. The secondary structure of FOXO-DBD is indicated at the top.

We investigated differences in the interactions of individual members of the FOXO family with **5ci**, **5cj**, **5db**, and **5dc** by performing ^1^H–^15^N HSQC with ^15^N-labeled FOXO1-DBD and FOXO4-DBD to compare CSP profiles of DBDs of all three FOXO variants (Figures 5 and S11). ^1^H–^15^N HSQC spectra of ^15^N-labeled FOXO1-DBD and FOXO4-DBD with and without **5ci**, **5cj**, **5db**, and **5dc** are shown in Figures S12–S20. For all four compounds, the CSP profiles of FOXO1-DBD and FOXO3-DBD were highly similar, indicating similar interactions between them and DBDs of these FOXO proteins. In contrast, FOXO4-DBD CSP profiles differed from FOXO1-DBD and FOXO3-DBD profiles, especially in the presence of **5ci** and **5db**. In these two compounds, the most significant CSPs (the chemical shift change was greater than 2σ_corr_^0^ above the mean) were mainly observed in residues located in the N-terminus of FOXO4-DBD. Conversely, in FOXO1-DBD and FOXO3-DBD, the largest CSPs were observed in helices H2, H3, and H4 residues (Figures 5b and S7a).

HADDOCK docking predicts that the naphthyl moiety of **5ci** interacts with a FOXO4-DBD surface formed by W^101^, Q^104^, S^105^, and Y^106^, whereas the pyrimidinylguanidine moiety makes contacts with the residues S^105^, H^156^, N^157^, and H^161^ (Figure S21a). In turn, our docking simulation suggested that **5db** is embedded between the N-terminal segment (residues N^99^, A^100^, and W^101^) and the loop between helices H2 and H4 (residues V^135^, Y^137^, and F^138^) and that the phenyl moiety also interacts with the side chain of Y^105^ in the N-terminus of helix H1 (Figure S21b). Taken together, these results indicate that phenylpyrimidinylguanidines may interact differently with different members of the FOXO subfamily, particularly FOXO4.

## Discussion and Conclusions

Three (4-propoxy)phenylpyrimidinylguanidine (**5ca**, Figure 1) derivatives, namely, **5ci**, **5cj**, and **5db**, inhibited FOXO3 binding to the promoter region of DEPP1 in neuroblastoma cells considerably better than their parent compound **5ca**, as shown by bioluminescence in a luciferase reporter assay (Figure 2). These results were further corroborated in a resazurin early cell death evaluation assay, which showed that these compounds have significantly lower IC_50_ values (0.5–1 μM) than **5ca** (Figure S4). The analysis of the effects of these compounds on target gene activation by FOXO3 suggested that their inhibitory potency depends on the length of the linker between the guanidine and aryl moieties (Figures 2, S2, and S3). Accordingly, **5cj** exhibited the highest inhibitory potency, whereas compounds **5cb** and **5cc**, with a shorter linker between the guanidine and benzene moieties, showed a lower ability to inhibit FOXO3, presumably due to lower flexibility.

Although extending the aromatic moiety by adding a methyl group (**5cd**, **5ce**, and **5cf**) or a fluorine atom (**5ch**) did not increase the inhibitory potency, introducing a *tert*-butyl group in the para-position (**5cg**) and replacing benzyl by a naphthyl (**5ci**) group increased FOXO3 inhibition. These findings suggest that the overall size of the aromatic moiety on the (4-propoxy)phenyl side is yet another factor that affects the inhibitory potency of these compounds. In line with this inference, all three compounds with a modified dimethylpyrimidinyl moiety (**5da–c**) were better inhibitors than the parent **5ca**. Of the three, **5db**, which contains the ethylbenzyl moiety, was the strongest inhibitor, albeit more toxic than **5ci** and **5cj** at higher concentrations (Figure 2). Therefore, combining further modifications of the dimethylpyrimidine moiety with changes in the (4-propoxy)phenyl side may open up new opportunities for improving the inhibitory efficacy of phenylpyrimidinylguanidines.

We have previously shown that the original **5ca** binds to the surface formed by the C-terminal half of the DNA recognition helix H3 and the N-terminus of β-strand S2, thereby blocking the interaction between FOXO3 and the target DNA and thus inhibiting the physiological program activated by FOXO3 in cancer cells.^25^ In this study, our structural characterization of the interaction between FOXO3-DBD and **5ci**, **5cj**, **5db**, and **5dc** by NMR and molecular docking revealed that these four compounds target different regions of FOXO3-DBD, but all binding sites directly participate in DNA binding and/or undergo a conformational change upon FOXO3 binding to the target DNA (Figure S22).^25,28,29^

The gradual shift in resonances of several FOXO3-DBD residues during the titrations (Figures 3c, 4b, S7b, and S9b) suggests a fast exchange of bound compounds on the NMR time scale, indicating weak binding affinity (in low mM range) under the conditions used. However, this contrasts with the IC_50_ values of 0.5–1 μM indicated by a resazurin early cell death evaluation assay (Figure S4). One factor responsible for this discrepancy may be the different conditions during NMR measurements compared to cell culture experiments, especially pH (pH 7.4 in cell cultures, pH 6.5 in NMR) and temperature (37 °C in cell cultures vs 25 °C in NMR). Furthermore, the IC_50_ values reflect the inhibition of FOXO3-induced cell death. To induce cell death, FOXO3 activation must reach a certain activation threshold; therefore, it is not necessary to inhibit all intracellular FOXO3 but only the fraction of active FOXO3 required to reach this threshold to trigger cell death.^15^ In addition, these compounds may also inhibit FOXO3-induced transcription not only by blocking its DNA-binding surface but also through another, yet unidentified, mechanism, e.g., binding to the transactivation domain.

By comparing solution structures of individual FOXO DNA-binding domains, we identified differences in both their conformation and flexibility, specifically in the positions of helices H1, H2, and H3 and at the interface between the H2–H3 loop, the helix H3, and the N-terminal segment.^25^ These differences likely reflect variations in protein sequences (Figure S23) and may explain why FOXO-DBDs, especially FOXO4-DBD, interact differently with phenylpyrimidinylguanidines (Figures 5 and S11). In addition, the interaction between the N-terminal segment of FOXO4-DBD and the p53 transactivation domain is essential for the overall stability of the p53:FOXO4 complex.^33−35^ This interaction between FOXO4 and p53 inhibits apoptosis in senescent cells, and its disruption by the D-retro-inverso peptide, which corresponds to the N-terminus of FOXO4-DBD, blocks the transcription of senescence-associated protein p21 and induces nuclear exclusion of active p53, thereby inducing death in senescent cells.^33^ Our results showed that **5cj**, **5ci**, **5db**, and **5dc** binding significantly affect residues of the N-terminal segment of FOXO4-DBD (Figures 5 and S11). Consequently, these compounds may also interfere with the interaction between FOXO4 and p53.

In conclusion, our phenylpyrimidinylguanidine derivatives inhibit gene transcription by FOXO3 with an IC_50_ in a submicromolar range and thus 1 order of magnitude more potent than the parent **5ca**. The inhibitory potency of these compounds depends on the linker length between the guanidine group and aromatic moiety, the overall size of this aromatic moiety, and the modification of the dimethylpyrimidinyl moiety at the other side of the guanidine group. The compounds interact with FOXO3-DBD differently, suggesting the possibility to use these substances to selectively target various parts of the FOXO DNA-binding surface.

The DNA-binding surfaces of transcription factors outside the nuclear receptor family are difficult drug development targets for their lack of well-defined, small-molecule-binding pockets, high solvent exposure, and positively charged residues.^36−38^ Nevertheless, FOXO3 inhibition by **5ca** and other examples of successful targeting of DNA-binding surfaces, such as the inhibition of forkhead transcription factor FOXM1,^39^ androgen receptor,^40^ signal transducer and activator of transcription 3,^41^ or transcription factor Gli1,^42^ have demonstrated that DNA-binding surfaces are feasible drug targets.

The question as to whether these compounds can steer distinct target gene subsets/functions of FOXO transcription factors will undoubtedly be answered in subsequent studies. Further optimization is also required to improve the inhibitory efficacy and toxicity of these substances. Yet, targeting the FOXO3-specific transcription program and the interactions between FOXOs and their binding partners are excellent opportunities for drug development aimed at damaging cancer cells and boosting anticancer immunity. Thus, our results may serve as a foundation for further optimizing **5ca** and developing therapeutic agents for inhibiting the activity of FOXO transcriptional factors and their interactions with other binding partners by identifying the requirements for enhancing the inhibitory potency of such compounds.

## Materials and Methods

### Synthesis of the Compounds

#### General

Chemicals with purity >98% were purchased from Merck KGaA (Darmstadt, Germany) and Fluorochem (Derbyshire, U.K.). Solvents of high-performance liquid chromatography (HPLC) purity were purchased from Lab-Scan (Gliwice, Poland) and Fisher Scientific (Hampton, New Hampshire) and dried by standard techniques. Thin-layer chromatography (TLC) was performed using silica gel plates 60 F254 (Merck KgaA, Darmstadt, Germany), and the compounds were visualized by irradiation with UV light and/or by treatment with a solution ninhydrin followed by heating. Column chromatography was performed on silica gel 60 (0.063–0.200 mm) (Merck KgaA, Darmstadt, Germany). Analytical HPLC analysis was carried out using an Agilent 6530 liquid chromatograph under the following conditions: Agilent Eclipse plus C18 column (3.5 μL, 4.6 mm × 100 mm), UV detection at λ_obs_ = 254 nm, flow rate: 0.4 mL/min, linear gradient elution method (5–100% of CH_3_CN in 0.1% aqueous formic acid over 8 min, then 100% CH_3_CN for 7 min). Samples for analysis were prepared by dissolving the compound in CH_3_CN/DMSO (15/1, v/v) mixture. All compounds are >95% pure by HPLC. ^1^H NMR and ^13^C NMR spectra were recorded on a Bruker Avance III HD 400 MHz spectrometer (Bruker, Billerica, Massachusetts). Chemical shifts for protons are given in δ, and they are referenced to residual protium in the NMR solvent (DMSO-*d*_6_: δ = 2.50 ppm, methanol-*d*_4_: δ = 4.87 ppm, chloroform-*d*_3_: δ = 7.26 ppm). Carbon chemical shifts are referenced to the carbon in the NMR solvent (DMSO-*d*_6_: δ = 39.52 ppm, methanol-*d*_4_: δ = 49.00 ppm, chloroform-*d*_3_: δ = 77.00 ppm). The coupling constants *J* are given in Hz. Structures of all prepared compounds (carbon signals) were verified using the CSEARCH-Robot-Referee server (https://nmrpredict.orc.univie.ac.at/c13robot/robot.php). IR DRIFT was recorded in cm^–1^ with a Nicolet AVATAR 370 FT-IR spectrometer (Thermo Fisher Scientific, Waltham, Massachusetts). High-resolution mass was recorded on an LCQ Fleet spectrometer (Thermo Fisher Scientific, Waltham, Massachusetts).

#### Preparation of Substrates

##### *N*-(4-Hydroxy-6-methylpyrimidin-2-yl)cyanamide (**3a**)

Dicyandiamide **1** (2.0 g, 23.8 mmol, 1.0 equiv) and ethyl acetoacetate (3.7 mL, 28.5 mmol, 1.2 equiv) were suspended in 15 mL of MeOH. Then, NaOMe (1.3 g, 23.8 mmol, 1.0 equiv) was added portionwise at room temperature. The reaction mixture was heated to 70 °C in an oil bath to reflux. With the full conversion of starting dicyandiamide in 24 h based on ^1^H NMR monitoring, the reaction mixture was cooled to room temperature. The formed precipitate was filtered and washed with 3 × 5 mL of MeOH. The filtrate cake was dissolved in 40 mL of water. This solution was neutralized with glacial acetic acid (pH ∼ 5), and the resulting white precipitate was filtered, washed with 3 × 40 mL of water, and dried under a reduced pressure of 0.4 mbar at room temperature. Physical and spectroscopic data were consistent with previously reported data.^26^

White amorphous solid. Yield = 33% (1.2 g). ^1^H NMR (400 MHz, DMSO-*d*_6_): δ 11.86 (s, 2H), 5.60 (s, 1H), 2.10 (s, 3H) ppm. ^13^C{^1^H} NMR (101 MHz, DMSO-*d*_6_): δ 162.1, 155.2, 153.4, 115.0, 102.5, 18.8 ppm. IR (KBr): ν = 3103, 2995, 2941, 2902, 2839, 2591, 2316, 2199, 2125, 1912, 1853, 1748, 1661, 1494, 1470, 1440, 1428, 1392, 1359, 1263, 1192, 1156, 1096, 1039, 1009, 958, 887 cm^–1^. HRMS (ESI+) *m*/*z*: calcd for C_6_H_7_N_4_O [M + H]^+^: 151.0614, found: 151.0614.

##### 1-(4-Hydroxy-6-methylpyrimidin-2-yl)-3-(4-propoxyphenyl)guanidine (**5aa**)

4-Propoxyaniline (400 mg, 2.64 mmol, 1.5 equiv) was added dropwise to a suspension of cyanamide **3a** (264.1 mg, 1.76 mmol, 1.0 equiv) in 5.2 mL of EtOH at room temperature. The reaction mixture was heated in an oil bath at 80 °C to reflux. With the full conversion of **3a** in 48 h based on TLC monitoring, the reaction mixture was cooled to −35 °C, followed by the addition of an aqueous solution of NaOH (10 mL, 0.1 w/w) dropwise. The resulting solids were filtered and washed with 4 × 20 mL of Et_2_O and dried under reduced pressure of 0.4 mbar at room temperature. Physical and spectroscopic data were consistent with previously reported data.^25^

White amorphous solid. Yield = 90% (710 mg). ^1^H NMR (400 MHz, DMSO-*d*_6_): δ 7.44 (d, *J* = 8.4 Hz, 2H), 6.83 (d, *J* = 8.9 Hz, 2H), 5.53 (s, 1H), 3.88 (t, *J* = 6.5 Hz, 2H), 2.06 (s, 3H), 1.71 (q, *J* = 7.0 Hz, 2H), 0.97 (t, *J* = 7.4 Hz, 3H) ppm. ^13^C{^1^H} NMR (101 MHz, DMSO-*d*_6_): δ 158.6, 156.4, 154.7, 131.9, 123.0 (2C), 114.6 (2C), 114.4, 103.4, 79.2, 69.1, 23.7, 22.1, 10.5 ppm. IR (KBr): ν = 3345, 3267, 3252, 3103, 3046, 3016, 2968, 2866, 1649, 1607, 1577, 1512, 1458, 1422, 1404, 1350, 1264, 1234, 1201, 1168, 1147, 1114, 1075, 1030, 985, 970 cm^–1^. HRMS (ESI+) *m*/*z*: calcd for C_15_H_20_N_5_O [M + H]^+^: 302.1617, found: 302.1612.

##### 1-(4-Chloro-6-methylpyrimidin-2-yl)-3-(4-propoxyphenyl)guanidine (**5ba**)

Guanidine **5aa** (100.0 mg, 0.33 mmol, 1.0 equiv) was added portionwise to a stirred 1 mL of POCl_3_ at room temperature. At this temperature, the mixture was stirred to a full conversion of guanidine (20 h based on ^1^H NMR monitoring). Then, the reaction mixture was concentrated using a rotavap. Water (2.0 mL) was added to an oily residue. The resulting white solids were filtered, washed with 3 × 1 mL of water, and dried under a reduced pressure of 0.4 mbar at room temperature.

White amorphous solid. Quantitative yield (105 mg). ^1^H NMR (400 MHz, DMSO-*d*_6_): δ 11.38 (br s, 1H), 10.33 (s, 1H), 8.56 (br s, 2H), 7.44 (s, 1H), 7.30 (d, *J* = 8.6 Hz, 2H), 7.06 (d, *J* = 8.7 Hz, 2H), 3.97 (t, *J* = 6.4 Hz, 2H), 1.75 (h, *J* = 7.0 Hz, 2H), 0.99 (t, *J* = 7.4 Hz, 3H) ppm. ^13^C{^1^H} NMR (101 MHz, DMSO-*d*_6_): δ 171.4, 161.0, 158.8, 157.0, 154.0, 128.2 (2C), 126.4, 116.7 (2C), 116.0, 69.8, 23.7, 22.4, 10.9 ppm. IR (KBr) ν = 3351, 3309, 3058, 2935, 2869, 2367, 1673, 1628, 1601, 1577, 1556, 1512, 1437, 1392, 1383, 1347, 1305, 1266, 1245, 1177, 1144, 1072, 982, 890, 863, 818, 779 cm^–1^. HRMS (ESI+) *m*/*z*: calcd for C_15_H_19_ClN_5_O [M + H]^+^: 320.1283, found: 320.1273.

#### General Procedure for the Preparation of **5da–dc**

The corresponding amine **4** (1.55 mmol, 2.5 equiv) was added in one portion to a stirred suspension of **5ba** (200 mg, 0.62 mmol, 1.0 equiv) and K_2_CO_3_ (214 mg, 1.55 mmol, 2.5 equiv) in 6.5 mL of MeCN. The reaction mixture was heated in an oil bath to 85 °C to reflux. With the full conversion of guanidine (typically overnight based on TLC monitoring), the reaction mixture was cooled to 0 °C (ice/water bath). The resulting solids were filtered and washed with 2 × 1 mL of MeCN followed by water (the *p*H of filtrate should be neutral). The product was dried under a reduced pressure of 0.4 mbar at room temperature.

##### 1-(4-(Benzylamino)-6-methylpyrimidin-2-yl)-3-(4-propoxyphenyl)guanidine (**5da**)

The title compound was synthesized according to the general procedure using benzylamine (170 μL, 1.55 mmol, 2,5 equiv).

White amorphous solid. Yield = 59% (110 mg). HPLC purity = 99% (*t*_R_ = 6.5 min). ^1^H NMR (400 MHz, CDCl_3_): δ 10.62 (br s, 1H), 9.27 (br s, 1H), 7.45–7.10 (m, 5H), 6.98–6.59 (m, 4H), 5.63 (s, 1H), 4.18 (s, 2H), 3.85 (t, *J* = 6.6 Hz, 2H), 2.12 (s, 3H), 1.78 (h, *J* = 7.1 Hz, 2H), 1.02 (t, *J* = 7.4 Hz, 3H) ppm. ^13^C{^1^H} NMR (101 MHz, CDCl_3_): δ = 164.8, 164.03, 159.5, 155.0, 152.3, 140.6, 138.87, 128.4 (2C), 126.9 (2C), 126.7 (2C), 124.6, 115.5 (2C), 94.7, 69.8, 45.7, 24.0, 22.7, 10.6 ppm. IR (KBr): ν = 3452, 3255, 3159, 3058, 3025, 2956, 2929, 2875, 2812, 1649, 1610, 1559, 1545, 1503, 1488, 1467, 1428, 1395, 1341, 1299, 1278, 1231, 1213, 1165, 1135, 1102, 1063, 1048, 1027, 979, 866, 833, 794, 734, 695, 668, 632, 552, 531 cm^–1^. HRMS (ESI+) *m*/*z*: calcd for C_22_H_27_N_6_O [M + H]^+^: 391.2241, found: 291.2241.

##### 1-(4-Methyl-6-(phenethylamino)pyrimidin-2-yl)-3-(4-propoxyphenyl)guanidine (**5db**)

The title compound was synthesized according to the general procedure, using 2-phenylethylamine (200 μL, 1.55 mmol, 2,5 equiv).

White amorphous solid. Yield = 41% (100 mg). HPLC purity = 97% (*t*_R_ = 6.8 min). ^1^H NMR (400 MHz, DMSO-*d*_6_): δ 7.69 (s, 2H), 7.24 (ddt, *J* = 22.0, 14.1, 7.3 Hz, 8H), 7.09 (s, 2H), 6.83 (d, *J* = 8.4 Hz, 2H), 5.85 (s, 1H), 3.86 (t, *J* = 6.5 Hz, 2H), 3.41 (s, 2H), 2.80 (t, *J* = 7.4 Hz, 2H), 2.11 (s, 3H), 1.71 (h, *J* = 7.1 Hz, 2H), 0.97 (t, *J* = 7.4 Hz, 4H) ppm. ^13^C{^1^H} NMR (101 MHz, DMSO-*d*_6_): δ 163.3, 162.8, 154.1, 152.1, 140.0, 129.2 (2C), 128.7 (2C), 126.5 (2C), 123.2, 115.3 (2C), 97.5, 69.5, 42.3, 35.5, 23.9, 22.6, 10.9 ppm (*two qC are missing*). IR (KBr): ν = 3461, 3297, 3159, 3070, 3025, 2977, 2956, 2920, 2666, 1649, 1610, 1559, 1488, 1464, 1428, 1392, 1341, 1299, 1272, 1240, 1189, 1168, 1129, 1108, 1066, 1033, 1006, 979, 937, 860, 833, 812, 794, 746, 698, 644, 534, 519 cm^–1^. HRMS (ESI+) *m*/*z*: calcd for C_23_H_29_N_6_O [M + H]^+^: 405.2397, found: 405.2395.

##### 1-(4-(Butylamino)-6-methylpyrimidin-2-yl)-3-(4-propoxyphenyl)guanidine (**5dc**)

The title compound was synthesized according to the general procedure, using *n*-butylamine (150 μL, 1.55 mmol, 2,5 equiv).

White amorphous solid. Yield = 35% (80 mg). HPLC purity ≥ 99% (*t*_R_ = 6.6 min). ^**1**^H NMR (400 MHz, DMSO-*d*_6_): δ 7.62 (br s, 2H), 7.09 (s, 3H), 6.83 (d, *J* = 8.4 Hz, 2H), 5.83 (s, 1H), 3.86 (t, *J* = 6.5 Hz, 2H), 3.17 (s, 2H), 2.10 (s, 3H), 1.70 (q, *J* = 7.0 Hz, 2H), 1.47 (p, *J* = 7.2 Hz, 2H), 1.32 (p, *J* = 7.3 Hz, 2H), 1.02–0.90 (m, 3H), 0.87 (d, *J* = 7.3 Hz, 3H) ppm. ^13^C{^1^H} NMR (101 MHz, DMSO-*d*_6_): δ 163.3, 154.1, 123.2 (2C), 115.2 (2C), 105.0, 97.1, 69.5, 40.4, 31.4, 23.9, 22.6, 20.1, 14.2, 10.9 ppm *(three qC are missing)*. IR (KBr): ν = 3461, 3414, 3294, 3204, 3162, 3028, 2956, 2926, 2869, 1646, 1610, 1485, 1467, 1431, 1395, 1362, 1341, 1314, 1272, 1237, 1153, 1102, 1057, 1006, 967, 923, 863, 839, 800, 749, 701 cm^–1^. HRMS (ESI+) *m*/*z*: calcd for C_19_H_29_N_6_O [M + H]^+^: 357.2397, found: 357.2407.

##### *N*-(4,6-Dimethylpyrimidin-2-yl)cyanamide (**3c**)

Dicyandiamide **1** (5.0 g, 60 mmol) and acetylacetone (9.0 g, 90 mmol) were added portionwise to 40 mL of a stirred aqueous 0.3 M NaOH solution at room temperature. The resulting suspension was heated in an oil bath to 110 °C to reflux. With the full conversion of dicyandiamine (48 h based on ^1^H NMR monitoring), the reaction mixture was cooled to 0 °C (ice/water bath). The resulting solids were filtered and washed with 1 × 20 mL of water. The crude product was recrystallized from 120 mL of boiling ethanol and dried under a reduced pressure of 0.4 mbar at room temperature. Physical and spectroscopic data were consistent with previously reported data.^26^

White crystalline solid. Yield = 44% (3.9 g). Mp = 228–229 °C (ethanol). ^1^H NMR (400 MHz, DMSO-*d*_6_): δ 12.58 (s, 1H), 6.63 (s, 1H), 2.31 (s, 6H) ppm. ^13^C{^1^H} NMR (101 MHz, DMSO-*d*_6_): δ 166.8, 160.2 (2C), 115.8, 109.7, 21.9 (2C) ppm. IR (KBr) ν = 3503, 3282, 3249, 3064, 3010, 2980, 2857, 2842, 2815, 2621, 2319, 2244, 2202, 2175, 2089, 1838, 1727, 1649, 1610, 1422, 1362, 1323, 1231, 1195, 1165, 1036, 1018, 985 cm^–1^. HRMS (ESI+) *m*/*z*: calcd for C_7_H_9_N_4_ [M + H]^+^: 149.0827, found: 149.0792.

#### General Procedure for the Preparation of Guanidines **5ca–cj**

The corresponding amine **4** (2.0 mmol, 1.5 equiv) was added dropwise to a stirred suspension of cyanamide **3c** (200 mg, 1.35 mmol, 1.0 equiv) in 4 mL of EtOH at room temperature. The reaction mixture was heated in an oil bath to 80 °C to reflux. With the full conversion of cyanamide (48 h based on TLC monitoring), the reaction mixture was cooled to −35 °C in a freezer. At this temperature, the solution of NaOH (10 mL, 0.1 w/w) was added dropwise. The resulting solids were filtered, washed with 4 × 20 mL of Et_2_O and dried under a reduced pressure of 0.4 mbar at room temperature.

##### 1-(4,6-Dimethylpyrimidin-2-yl)-3-(4-propoxyphenyl)guanidine (**5ca**)

The title compound was synthesized according to the general procedure, using 4-propoxyaniline (300 μL, 2.0 mmol, 1.5 equiv).

White amorphous solid. Yield = 47% (193 mg). HPLC purity = 99% (*t*_R_ = 5.8 min). ^1^H NMR (400 MHz, MeOD-*d*_4_): δ 7.21–7.08 (m, 2H), 6.92–6.83 (m, 2H), 6.61 (s, 1H), 3.89 (t, *J* = 6.5 Hz, 2H), 3.27 (p, *J* = 1.6 Hz, 1H), 2.31 (s, 6H), 1.83–1.65 (m, 2H), 1.01 (t, *J* = 7.4 Hz, 3H) ppm. ^13^C{^1^H} NMR (101 MHz, MeOD-*d*_4_): δ 168.6 (2C), 165.2, 158.1, 157.3, 133.7, 126.6 (2C), 116.4 (2C), 112.9, 70.9, 23.74 (2C), 23.69, 10.9 ppm. IR (KBr) ν = 3312, 3106, 3088, 2959, 2893, 2869, 1631, 1577, 1527, 1509, 1419, 1383, 1344, 1237, 1171, 1117, 1075, 1048, 1024 cm^–1^. HRMS (ESI+) *m*/*z*: calcd for C_16_H_22_N_5_O [M + H]^+^: 300.1824, found: 300.1773.

##### 1-(4,6-Dimethylpyrimidin-2-yl)-3-phenylguanidine (**5cb**)

The title compound was synthesized according to the general procedure, using aniline (180 μL, 2.0 mmol, 1.5 equiv).

White amorphous solid. Yield = 31% (100 mg). ^1^H NMR (400 MHz, DMSO-*d*_6_): δ 7.34 (d, *J* = 7.9 Hz, 2H), 7.20–7.07 (m, 2H), 6.72 (tt, *J* = 7.2, 1.2 Hz, 1H), 6.22 (s, 1H), 2.16 (s, 6H) ppm. ^13^C{^1^H} NMR (101 MHz, DMSO-*d*_6_): δ 166.2, 165.5 (2C), 157.7, 147.3, 128.5 (2C), 121.0, 119.3 (2C), 107.6, 24.1 (2C) ppm. IR (KBr): ν = 3306, 3273, 3231, 3150, 3114, 3052, 2962, 2920, 1637, 1604, 1577, 1524, 1500, 1449, 1416, 1407, 1377, 1332, 1299, 1245, 1204, 1174, 1159, 1114, 1102, 1027, 991, 967, 899, 830, 806, 746 cm^–1^. HRMS (ESI+) *m*/*z*: calcd for C_13_H_16_N_5_ [M + H]^+^: 241.1405, found: 242.1400.

##### 1-Benzyl-3-(4,6-dimethylpyrimidin-2-yl)guanidine (**5cc**)

The title compound was synthesized according to the general procedure, using benzylamine (220 μL, 2.0 mmol, 1.5 equiv).

White amorphous solid. Yield = 46% (155 mg). ^1^H NMR (400 MHz, DMSO-*d*_6_): δ 7.33 (d, *J* = 5.0 Hz, 4H), 7.29–7.17 (m, 1H), 6.46 (s, 1H), 4.48 (s, 2H), 2.21 (s, 6H) ppm. ^13^C{^1^H} NMR (101 MHz, DMSO-*d*_6_): δ 166.5, 166.1 (2C), 158.1, 140.7, 128.7 (2C), 127.5 (3C), 127.1, 110.5, 43.9, 24.0 (2C) ppm. IR (KBr): ν = 3276, 3183, 3129, 3108, 3022, 2923, 2866, 1607, 1577, 1419, 1374, 1329, 1240, 1210, 1174, 1099, 1081, 1060, 988, 973, 946, 920, 887, 827, 809, 749, 737, 698 cm^–1^. HRMS (ESI+) *m*/*z*: calcd for C_14_H_18_N_5_ [M + H]^+^: 256.1554, found: 256.1557.

##### 1-(4,6-Dimethylpyrimidin-2-yl)-3-(4-methylbenzyl)guanidine (**5cd**)

The title compound was synthesized according to the general procedure, using 4-methylbenzylamine (260 μL, 2.0 mmol, 1.5 equiv).

White amorphous solid. Yield = 72% (260 mg). ^1^H NMR (400 MHz, DMSO-*d*_6_): δ 7.65 (br s, 1H), 7.21 (d, *J* = 8.0 Hz, 2H), 7.13 (d, *J* = 7.8 Hz, 2H), 6.46 (s, 1H), 4.43 (s, 2H), 2.27 (s, 3H), 2.21 (s, 6H) ppm. ^13^C{^1^H} NMR (101 MHz, DMSO-*d*_6_): δ 166.5, 166.1 (2C), 158.2, 137.6, 136.1, 129.3 (2C), 127.5 (2C), 110.5, 43.8, 24.0 (2C), 21.1 ppm. IR (KBr): ν = 3270, 3252, 3103, 2920, 2884, 2155, 1900, 1607, 1592, 1565, 1521, 1416, 1377, 1338, 1207, 1159, 1135, 1069, 1030, 1021, 985, 964, 952, 911, 833, 812 cm^–1^. HRMS (ESI+) *m*/*z*: calcd for C_15_H_20_N_5_ [M + H]^+^: 270.1717, found: 270.1713.

##### 1-(4,6-Dimethylpyrimidin-2-yl)-3-(3-methylbenzyl)guanidine (**5ce**)

The title compound was synthesized according to general procedure, using 3-methylbenzylamine (250 μL, 2.0 mmol, 1.5 equiv).

White amorphous solid. Yield = 72% (263 mg). ^1^H NMR (400 MHz, DMSO-*d*_6_): δ 7.21 (t, *J* = 7.5 Hz, 1H), 7.16–7.09 (m, 2H), 7.05 (d, *J* = 7.5 Hz, 1H), 6.47 (s, 1H), 4.44 (s, 2H), 2.29 (s, 3H), 2.22 (s, 6H) ppm. ^13^C{^1^H} NMR (101 MHz, DMSO-*d*_6_): δ 166.5, 166.1 (2C), 158.0, 140.5, 137.8, 128.7, 128.1, 127.8, 124.6, 110.6, 43.9, 24.0 (2C), 21.5 ppm. IR (KBr): ν = 3267, 3106, 3049, 3016, 2917, 1939, 1613, 1571, 1539, 1425, 1380, 1332, 1308, 1237, 1160, 1144, 1096, 1069, 1036, 1015, 991, 970, 943, 827, 812 cm^–1^. HRMS (ESI+) *m*/*z*: calcd for C_15_H_20_N_5_ [M + H]^+^: 270.1716, found: 270.1713.

##### 1-(4,6-Dimethylpyrimidin-2-yl)-3-(2-methylbenzyl)guanidine (**5cf**)

The title compound was synthesized according to the general procedure, using 2-methylbenzylamine (250 μL, 2.0 mmol, 1.5 equiv).

White amorphous solid. Yield = 80% (300 mg). ^1^H NMR (400 MHz, DMSO-*d*_6_): δ 7.65 (br s, 1H), 7.21 (d, *J* = 8.0 Hz, 2H), 7.13 (d, *J* = 7.8 Hz, 2H), 6.46 (s, 1H), 4.43 (s, 2H), 2.27 (s, 3H), 2.21 (s, 6H) ppm. ^13^C{^1^H} NMR (101 MHz, DMSO-*d*_6_): δ 166.5, 165.9 (2C), 158.3, 138.6, 135.9, 130.3, 127.6, 127.0, 126.1, 109.9, 42.4, 23.5 (2C), 19.1 ppm. IR (KBr): ν = 3282, 3267, 3108, 3094, 3064, 2983, 1619, 1568, 1530, 1416, 1332, 1314, 1245, 1219, 1192, 1153, 1114, 1060, 1045, 1030, 991, 967, 931, 890, 806 cm^–1^. HRMS (ESI+) *m*/*z*: calcd for C_15_H_20_N_5_ [M + H]^+^: 270.1714, found: 270.1713.

##### 1-(4-(*tert*-Butyl)benzyl)-3-(4,6-dimethylpyrimidin-2-yl)guanidine (**5cg**)

The title compound was synthesized according to the general procedure, using 4-*tert*-butylbenzylamine (350 μL, 2.0 mmol, 1.5 equiv).

White amorphous solid. Yield = 82% (345 mg). HPLC purity = 99% (*t*_R_ = 6.5 min). ^1^H NMR (400 MHz, CDCl_3_): δ 7.36–7.32 (m, 2H), 7.28 (s, 2H), 6.39 (s, 1H), 4.48 (s, 2H), 2.27 (s, 6H), 1.29 (s, 9H) ppm. ^13^C{^1^H} NMR (101 MHz, CDCl_3_): δ 166.4 (2C), 158.3, 150.6, 134.8, 126.7 (2C), 125.8 (2C), 111.1, 45.2, 34.5, 31.3 (3C), 24.00 (2C) ppm, *one qC is missing*. IR (KBr): ν = 3270, 3258, 3108, 3055, 2962, 2866, 1897, 1616, 1571, 1530, 1416, 1380, 1355, 1305, 1272, 1240, 1177, 1162, 1117, 1018, 988, 970, 934, 899, 824, 806 cm^–1^. HRMS (ESI+) *m*/*z*: calcd for C_18_H_26_N_5_ [M + H]^+^: 312.2187, found: 312.2183.

##### 1-(4,6-Dimethylpyrimidin-2-yl)-3-(4-fluorobenzyl)guanidine (**5ch**)

The title compound was synthesized according to the general procedure, using 4-fluorobenzylamine (230 μL, 2.0 mmol, 1.5 equiv).

White amorphous solid. Yield = 68% (250 mg). ^1^H NMR (400 MHz, DMSO-*d*_6_): δ 7.75 (br s, 2H), 7.41–7.30 (m, 2H), 7.19–7.08 (m, 2H), 6.47 (s, 1H), 4.46 (s, 2H), 2.21 (s, 6H) ppm. ^13^C{^1^H} NMR (101 MHz, DMSO-*d*_6_): δ 166.1, 166.7, 162.3, 159.9, 157.8, 136.6, 129.0 (d, *J* = 8.0 Hz, 2C), 115.0 (d, *J* = 21.2 Hz, 2C), 110.2, 42.7, 23.6 (2C) ppm. ^19^F NMR (376 MHz, DMSO-*d*_6_): δ −116.51 ppm. IR (KBr): ν = 3258, 3103, 3088, 3067, 2962, 1885, 1622, 1601, 1577, 1542, 1509, 1419, 1383, 1338, 1216, 1180, 1156, 1096, 1015, 994, 976, 943, 931, 899, 827, 806 cm^–1^. HRMS (ESI+) *m*/*z*: calcd for C_14_H_17_FN_5_ [M + H]^+^: 274.1465, found: 274.1463.

##### 1-(4,6-Dimethylpyrimidin-2-yl)-3-(naphthalen-1-ylmethyl)guanidine (**5ci**)

The title compound was synthesized according to the general procedure, using 1-naphthylmethylamine (290 μL, 2.0 mmol, 1.5 equiv).

White amorphous solid. Yield = 82% (340 mg). HPLC purity = 99% (*t*_R_ = 6.0 min). ^1^H NMR (400 MHz, DMSO-*d*_6_): δ 8.23–8.09 (m, 1H), 8.03–7.92 (m, 1H), 7.86 (dd, *J* = 7.4, 2.0 Hz, 1H), 7.70 (br s, 1H), 7.63–7.37 (m, 4H), 6.48 (s, 1H), 4.97 (s, 2H), 2.21 (s, 6H) ppm. ^13^C{^1^H} NMR (101 MHz, DMSO-*d*_6_): δ 166.5, 166.1 (2C), 158.3, 135.8, 133.7, 131.3, 128.9, 127.8, 126.6, 126.2, 125.9, 125.3, 124.0, 110.6, 42.2, 24.0 (2C) ppm. IR (KBr): ν = 3279, 3117, 3088, 3067, 3052, 2923, 2792, 1607, 1568, 1533, 1416, 1383, 1335, 1257, 1231, 1213, 1177, 1045, 1027, 994, 967, 937, 887, 830, 809, 776 cm^–1^. HRMS (ESI+) *m*/*z*: calcd for C_18_H_20_N_5_, [M + H]^+^: 306.1714, found: 306.1713.

##### 1-(4,6-Dimethylpyrimidin-2-yl)-3-phenethylguanidine (**5cj**)

The title compound was synthesized according to the general procedure, using 2-phenylethylamine (250 μL, 2.0 mmol, 1.5 equiv).

White amorphous solid. Yield = 61% (300 mg). HPLC purity = 98% (*t*_R_ = 5.4 min). ^1^H NMR (400 MHz, DMSO-*d*_6_): δ 7.34–7.24 (m, 5H), 7.24–7.17 (m, 1H), 6.45 (s, 1H), 3.46 (t, *J* = 7.2 Hz, 2H), 2.81 (t, *J* = 7.2 Hz, 2H), 2.20 (d, *J* = 6.5 Hz, 6H) ppm. ^13^C{^1^H} NMR (101 MHz, DMSO-*d*_6_): δ 166.5, 166.0 (2C), 158.0, 140.1, 129.2 (2C), 128.8 (2C), 126.5, 110.4, 42.0, 35.8, 24.1 (2C) ppm. IR (KBr): ν = 3282, 3117, 2998, 2947, 2920, 1619, 1571, 1542, 1419, 1377, 1332, 1287, 1263, 1216, 1177, 1093, 1033, 991, 967, 949, 926, 845, 830, 809, 719 cm^–1^. HRMS (ESI+) *m*/*z*: calcd for C_15_H_20_N_5_ [M + H]^+^: 270.1715, found: 270.1713.

### Estimation of Compound Solubility in Water

To estimate the solubility, we prepared saturated solutions of the compounds by adding 5 mg of each selected compound to 700 μL of 20 mM phosphate buffer (pH 6.5) containing 50 mM KCl. After 48 h of gentle agitation at room temperature (RT), the solutions were spun down, and the supernatant was filtered through 0.2 μm of SPARTAN 13/0.2 RC filter units (Merck KgaA, Darmstadt, Germany) and diluted 100–200× in the same phosphate buffer. The diluted solutions were transferred into the quartz cuvette, recording UV–vis absorption spectra in the range from 200 to 600 nm on a UV-1800 UV/visible scanning spectrophotometer (Shimadzu, Kyoto, Japan) at room temperature. The compound concentrations were estimated by the single point calibration using the absorption at wavelengths higher than 240 nm outside of DMSO absorption of 2000× diluted 50 mM DMSO-dissolved stock solution in phosphate buffer.

### Protein Preparation

FOXO3-DBD and FOXO1-DBD were purified, as described previously.^25,32^ DNA encoding human FOXO3-DBD (residues 156–269) and mouse FOXO1-DBD (residues 156–269) were cloned into pGEX-6P-1 and expressed in E. coli BL21(DE3) as the N-terminal GST-tagged fusion proteins. Proteins for ^1^H–^15^N HSQC NMR experiments were expressed in minimal media supplemented with 1 g/L ^15^N-ammonium chloride (Cambridge Isotope Laboratories, Inc., Tewksbury, Massachusetts) as the sole nitrogen source and purified as described below. Protein expression was induced with 0.5 mM isopropyl β-d-1-thiogalactopyranoside (Sigma-Aldrich s.r.o., St. Louis, Missouri) for 16–20 h at 20 °C. The pellet from 1 L of culture was resuspended in 25 mL of lysis buffer containing 20 mM phosphate (pH 7.4), 250 mM NaCl, 1 mM ethylenediamine tetraacetic acid (EDTA), and 10 mM dithiothreitol (DTT). The cells were disrupted by sonication, and the fusion protein was purified by Glutathione Sepharose 4 Fast Flow (GE Healthcare, Chicago, Illinois) in a buffer containing 20 mM tris–HCl (pH 7.5), 0.5 M NaCl, 1 mM EDTA, and 10 mM DTT. After elution with 10 mM reduced glutathione (pH 8.0), the proteins were dialyzed against a buffer containing 20 mM tris–HCl buffer (pH 7.5), 150 mM NaCl, 1 mM DTT, 1 mM EDTA, and 10% (wt/vol) glycerol. The GST affinity tag was removed by PreScission protease (10 U/mg of recombinant protein) by incubation at 4 °C overnight. The last purification step was size-exclusion chromatography on a HiLoad Superdex 75 26/600 column (GE Healthcare, Chicago, Illinois) in a buffer containing 20 mM phosphate buffer (pH 6.5), 1 mM DTT, 50 mM KCl, and 10% (wt/vol) glycerol.

Human FOXO4-DBD (residues 86–211) was expressed as a His_6_-N-terminal tagged fusion protein and purified as described previously.^35^ The affinity tag was removed by thrombin (Sigma-Aldrich s.r.o., St. Louis, Missouri) (10 U/mg recombinant proteins) by incubation at 4 °C overnight. The protein was then dialyzed against a 20 mM citric acid (pH 6.3) and 1 mM EDTA buffer. Subsequently, FOXO4-DBD was purified by cation-exchange chromatography on a HiTrap SP column (GE Healthcare, Chicago, Illinois). The final purification step was size-exclusion chromatography on a HiLoad Superdex 75 26/600 column (GE Healthcare, Chicago, Illinois) in a buffer containing 20 mM phosphate buffer (pH 6.5), 1 mM DTT, 50 mM KCl, and 10% (wt/vol) glycerol.

### STD NMR Measurements

STD experiment was performed at 298 K on a Bruker Avance III HD 850 MHz spectrometer (Bruker, Billerica, Massachusetts) equipped with a ^13^C/^1^H/^15^N cryoprobe. The 550 μL sample contained 15 μM FOXO3-DBD and 1 mM **5cj** in 20 mM phosphate buffer (pH 6.5) supplemented by 50 mM KCl and 10% ^2^H_2_O. ^1^H STD NMR was performed using a standard “stddiffesgp” pulse sequence with excitation sculpting and pulse-field gradients for water suppression. On-resonance irradiation was done at −0.54 ppm and off-resonance irradiation at 30 ppm, using a 50 ms shaped Eburp2.1000 pulse of power 40 dB for a saturation time of 3 s. The difference spectra were obtained by subtracting the on-resonance spectrum from the off-resonance spectrum. The spectra were analyzed using TopSpin software (v3.6).

### 2D ^1^H–^15^N HSQC NMR Measurements

2D ^1^H–^15^N HSQC spectra were acquired on both Bruker Avance III HD 850 MHz and Bruker Avance III 600 MHz (Bruker, Billerica, Massachusetts) spectrometers equipped with a ^13^C/^1^H/^15^N cryoprobe. All HSQC experiments were measured at 298 K in Shigemi NMR tubes (Shigemi Co., Ltd., Tokyo, Japan) containing 350 μL of sample in 100 mM phosphate buffer (pH 6.5) supplemented with 50 mM KCl and 10% ^2^H_2_O. The 100 mM phosphate buffer was used to avoid unwanted effects caused by pH changes induced by the presence of the tested compounds at high concentrations. 2D ^1^H–^15^N HSQC measurements were performed with 100 μM FOXO-DBDs and a given concentration of the compound. All spectra were processed in TopSpin software (v3.6) and evaluated in Sparky software (v3.1).^43^ CSP values obtained from 2D ^1^H–^15^N HSQC experiments were calculated using the following formula^44^
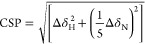


### HADDOCK Docking Calculations

Docking calculations were performed using HADDOCK2.4.^30,31^ FOXO3-DBD and FOXO4-DBD coordinates were taken as the lowest-energy structure of the PDB entries 2K86 and 1E17. The first two N-terminal amino acid residues of FOXO3-DBD from the thrombin cleavage site were removed from the structure. For all docking runs, active residues of FOXO3-DBD were chosen as residues with CSPs higher than the mean + 2σ_corr_^0^. For FOXO3-DBD, residues 151–160 (N-terminus) and 241–251 (C-terminus) were set as flexible. For FOXO4-DBD, residues 96–105 (N-terminus) and 181–185 (C-terminus) were set as flexible. The ligand was set as active and flexible in both cases. Unmentioned running parameters were left on their default values for protein–ligand mode. The resulting structures were clustered by RMSD with a 1.5 Å cutoff and ranked based on their HADDOCK scores. The top-scoring cluster satisfactorily explained the NMR data of all compounds but **5ci**. Its data were satisfactorily explained by combining the top and second clusters, as described in the Results section.

### Cell Lines, Culture Conditions, and Reagents

The neuroblastoma cell line SH-EP/FOXO3 was cultured in RPMI1640 (Lonza, Basel, Switzerland) with 10% fetal bovine serum (GIBCO BRL, Paisley, U.K.), 100 U/mL penicillin, 100 μg/mL streptomycin, and 2 mM l-glutamine (Sigma-Aldrich, Vienna, Austria) at 5% CO_2_. All cultures were routinely tested for mycoplasma contamination using the Venor GeM-mycoplasma detection kit (Minerva Biolabs, Berlin, Germany).

### Promoter Activity

Transcriptional activation of the DEPP1 promoter by conditional FOXO3 was assessed after transfection of a DEPP1-luciferase reporter plasmid pGS c10orf10 Prom wt (DEPP1-LUC)^20^ into SH-EP/FOXO3 cells using the JetPrime reagent (Polyplus, Berkeley). After 24 h of transfection, mTFP fluorescent protein expression from a cotransfected expression vector was assessed by live cell fluorescence microscopy to verify equal transfection rates before seeding cells into 24-well plates. After 48 h of transfection, the cells were pretreated with indicated concentrations of each compound (30 min) before adding 100 nM 4-hydroxy-tamoxifen (4OHT) for 3 h to activate ectopic FOXO3. Firefly-luciferase was analyzed using the Luciferase Assay System (Promega, Madison). Luminescence intensity was measured in a Hidex Chameleon—Multilabel Microplate Reader (Hidex, Turku, Finland).

The effects of these compounds on the endogenous DEPP1 promoter and on the actin promoter, which does not contain a FOXO consensus sequence, were assessed in SH-EP cells. For this purpose, 2 × 10^6^ SH-EP cells were transfected with either a DEPP1-LUC-reporter plasmid or an actin-LUC reporter plasmid using JetPrime reagent (Polyplus, Berkeley). Actin-LUC was constructed by inserting a 1.3 kb fragment of the chicken β actin promoter into pGL3-luc reporter vector (Promega, Madison). After 24 h of transfection, the cells were seeded into 24-well plates. After another 24 h, the cells were pretreated with indicated concentrations of compound for 3.5 h. Firefly-luciferase was analyzed using the Luciferase Assay System (Promega, Madison). Luminescence intensity was measured in a Hidex Chameleon—Multilabel Microplate Reader (Hidex, Turku, Finland).

### Fluorescence Polarization (FP) Analyses

The binding of compound **5ca** and its derivatives to FOXO3-DBD was measured *in vitro* by FP in black, flat-bottom, 96-well plates (HVD Life Sciences, Vienna, Austria) in a Hidex Chameleon—Multilabel Microplate Reader (Hidex, Turku, Finland). Each compound (dissolved in DMSO, 1 μM final concentration) was added to 100 μL of reaction mix containing 125 nM FOXO3-DBD and 25 nM FAM-labeled double-strand FOXO3 consensus sequence oligonucleotides (5′-CTA TCA AAA CAA CGC-3′) in assay buffer (20 mM tris–HCl, 100 mM NaCl, 1 mM EDTA, pH 7.5). The final concentration of DMSO in all mixtures was adjusted to 5% (v/v). Positive (only assay buffer and FAM-labeled oligonucleotide) and negative (FOXO3-DBD and FAM-labeled oligonucleotide) controls were analyzed on each plate. Milli polarization values (mP) were measured at an excitation wavelength of 485 nm and an emission wavelength of 530 nm.

### Resazurin Viability Measurement to Determine IC_50_ Values for FOXO3-Interacting **5ca** Derivatives

Neuroblastoma cells SH-EP/FOXO3 were seeded into 96-well plates and treated with concentrations 0.05, 0.1, 0.2, 0.39, 0.78, 1.56, 3.125, 6.25, 12.5, 18.75, and 25 μM of **5ca** and its derivatives 30 min before activating ectopic FOXO3 by 100 nM 4OHT. For 5ca derivatives, concentrations above 3.125 μM had to be excluded from the analysis due to spontaneous cell death induction. Resazurin salt (Sigma-Aldrich s.r.o., St. Louis, Missouri) was diluted to a final concentration of 0.01% (wt/vol), and 20 μL of this dilution was added to 200 μL of the cell culture in each well 24 h after FOXO3 activation. Resazurin is reduced in viable cells to red-fluorescent resoflurin, which was quantified in a Hidex Chameleon—Multilabel Microplate Reader (Hidex, Turku, Finland) at filter settings 544 nm *E*_x_/616 nm *E*_m_.

## Supporting Information Available

Forty-eight supplemental figures, HPLC traces (Figures S24–S30), and NMR spectra (Figures S31–S48) of the prepared compounds (PDF)
CSEARCH_reports (ZIP)
