# Supplementary material for: Lengthening the Guanidine–Aryl Linker of Phenylpyrimidinylguanidines Increases Their Potency as Inhibitors of FOXO3-Induced Gene Transcription
Source: ACS Omega. 2022 Sep 14;7(38):34632–46. doi: 10.1021/acsomega.2c04613 (PMC9521028; doi:10.1021/acsomega.2c04613)
Supplement: Supplementary file 1 — ao2c04613_si_001.pdf [file ao2c04613_si_001.pdf]

Supporting information

Lengthening the Guanidine-Aryl Linker of Phenylpyrimidinylguanidines Increases their Potency as Inhibitors of FOXO3-Induced Gene Transcription

Klara Kohoutova[a,b], Vojtěch Dočekal[c], Michael J. Ausserlechner[d], Nora Kaiser[d], Andrej Tekel[a], Raju Mandal[a], Matej Horvath[a], Veronika Obsilova[b], Jan Vesely[c], Judith Hagenbuchner[e,*], and Tomas Obsil[a,b,*]

[a] *Department of Physical and Macromolecular Chemistry, Faculty of Science, Charles University, Prague 12843, Czech Republic;*

[b] *Institute of Physiology of the Czech Academy of Sciences, Laboratory of Structural Biology of Signaling Proteins, Division BIOCEV, Prumyslova 595, Vestec 25250, Czech Republic;*

[c] *Department of Organic Chemistry, Faculty of Science, Charles University in Prague, Hlavova 2030, 128 43 Praha 2, Czech Republic;*

[d] *Department of Pediatrics I, Medical University Innsbruck, Innrain 66, Innsbruck, Austria;*

[e] *Department of Pediatrics II, Medical University Innsbruck, Innrain 66, Innsbruck, Austria*

***Correspondence:** Tomas Obsil, Faculty of Science, Charles University, Albertov 6, Prague, 12843, Czech Republic, Email: obsil@natur.cuni.cz, Tel. +420-221951303. Judith Hagenbuchner, Department of Pediatrics II, Medical University Innsbruck, Innrain 66, Innsbruck, Austria, Email: Judith.Hagenbuchner@i-med.ac.at, Tel.: +43-51250481578.

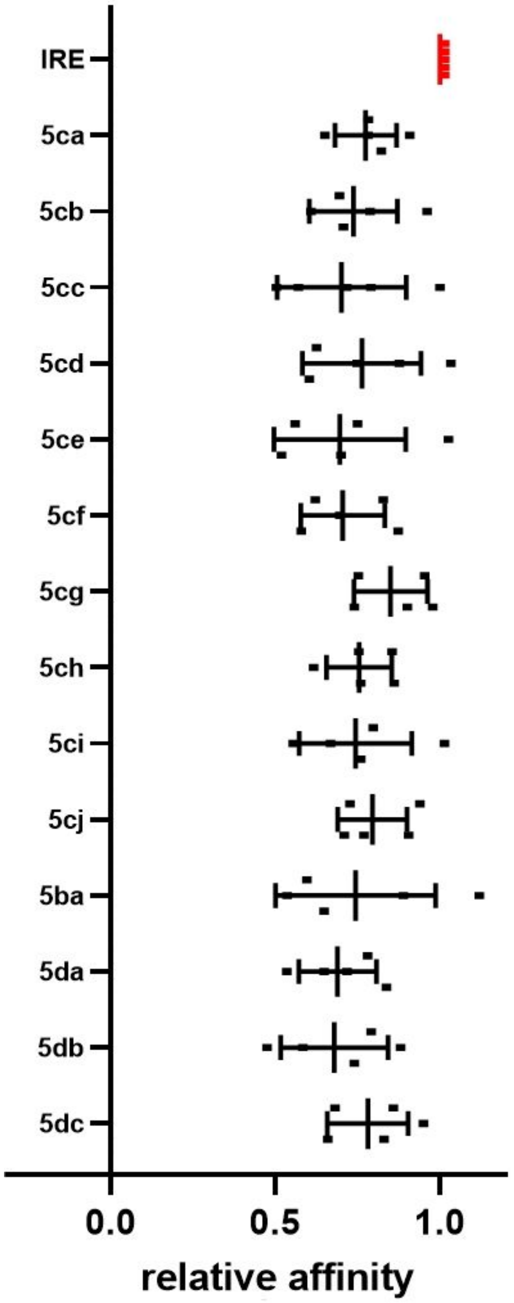

Figure S1. Fluorescence polarization assay (FP)-analyses of 5ca-derivatives binding to FOXO3-DBD. Shown is the mean of five independent experiments.

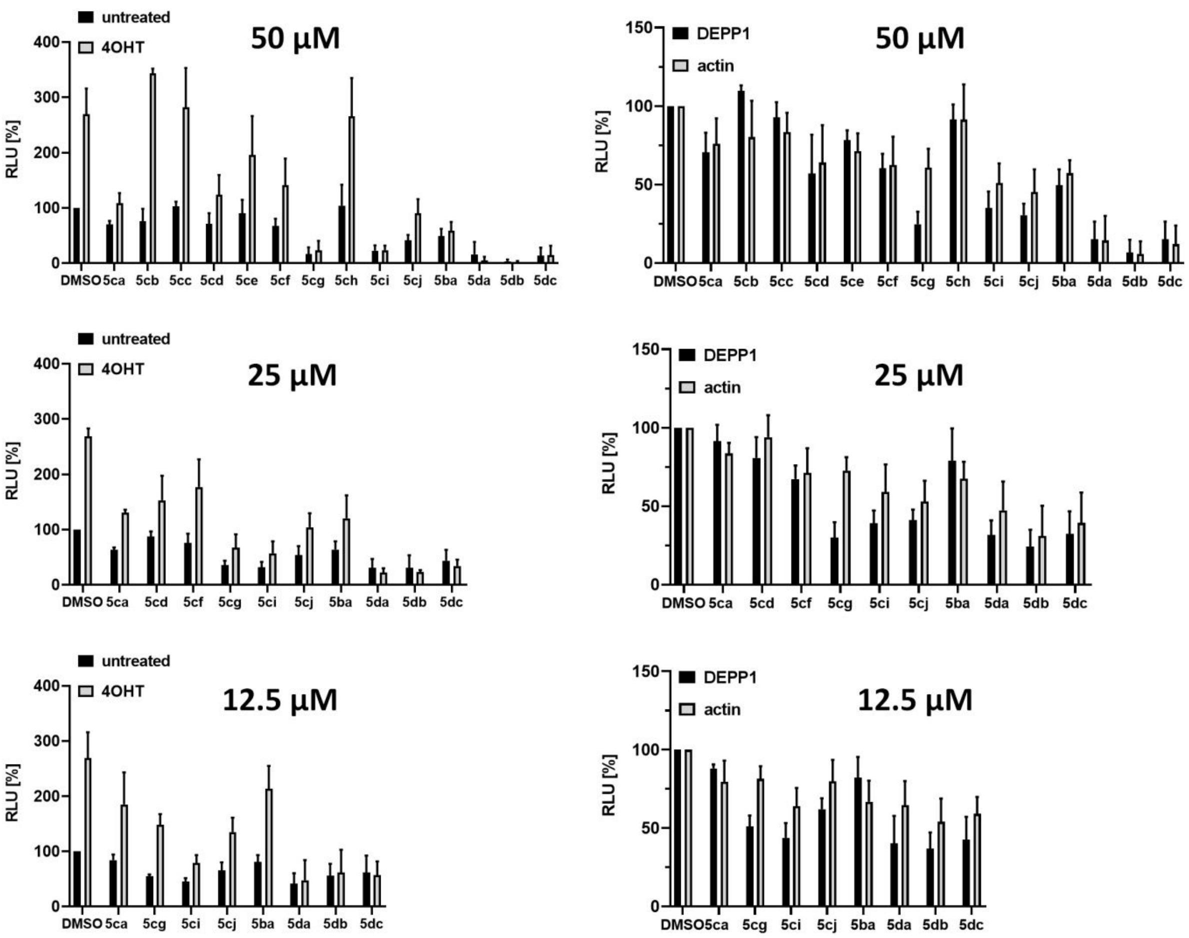

Figure S2. Initial Screen for compound-mediated inhibition of FOXO3 transcriptional activity. SH-EP/FOXO3 cells transfected with either DEPP1-LUC or Actin-LUC (as toxicity control) were treated with indicated concentrations (50 µM, 25 µM and 12.5 µM) of **5ca** and **5ca**-derivatives before 4OHT was added to activate ectopic FOXO3. The increase of firefly-luciferase activity/light emission was calculated as percentage of DMSO-only control. Shown are mean values +SD of three independent experiments, each performed in triplicates.

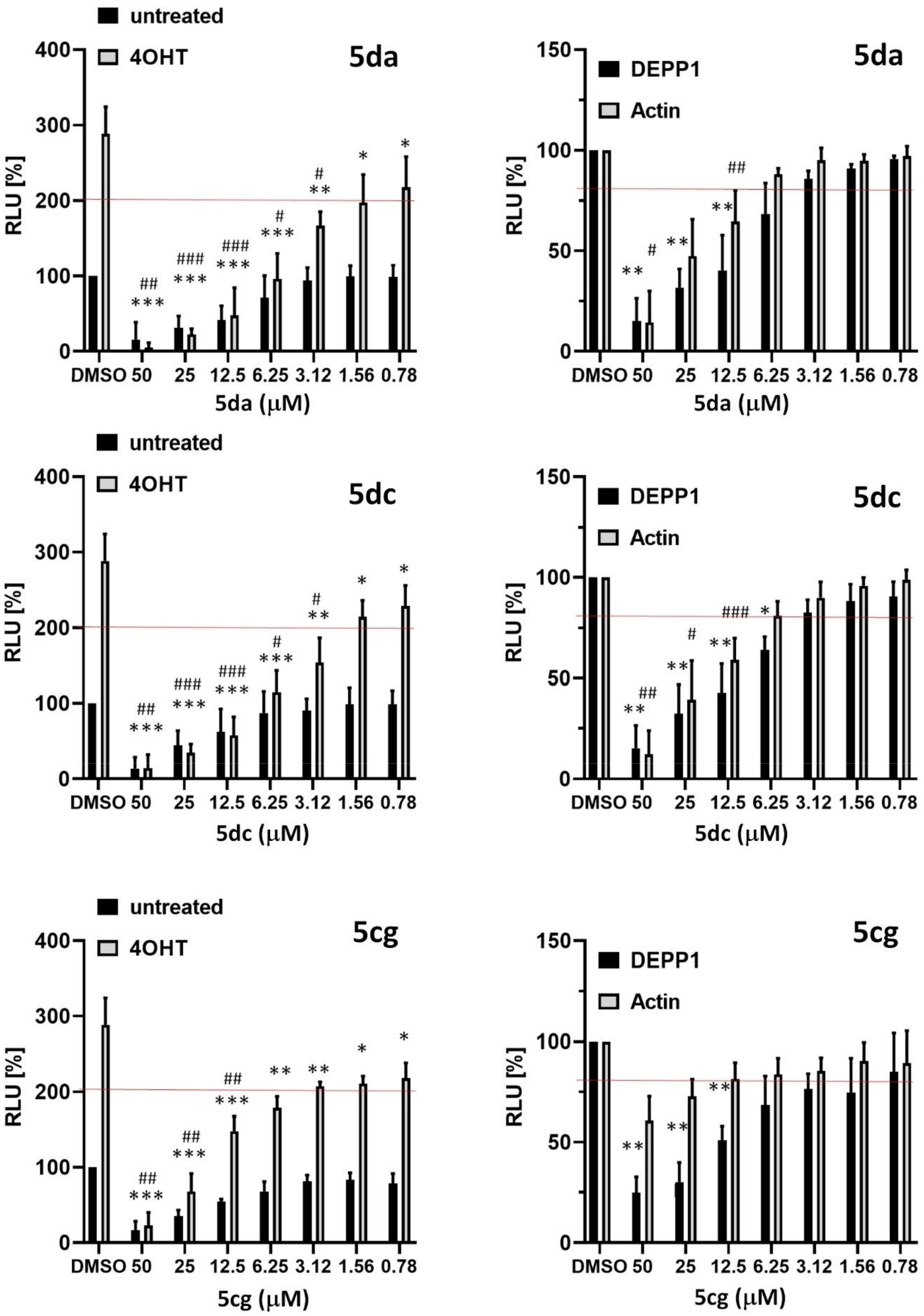

Figure S3. **Inhibition of FOXO3 transcriptional activity by selected 5ca-derivatives.** SH-EP/FOXO3 cells transfected with either DEPP1-LUC or Actin-LUC (as toxicity control) were treated with indicated concentrations of **5ca**-derivatives **5da**, **5dc** and **5cg** before 4OHT was

added to activate ectopic FOXO3. Shown are mean values +SD of three independent experiments, each performed in triplicates. The red line indicated efficacy of original **5ca** in inhibiting FOXO3-induced RLU at 12.5 μM. Significant differences between 4OHT treatment and substance+4OHT: *p<0.05, **p<0.01, ***p<0.001; Significant differences between **5ca** treatment and new derivates at the same concentration: #p<0.05, ##p<0.01, ###p<0.001 (students t-test).

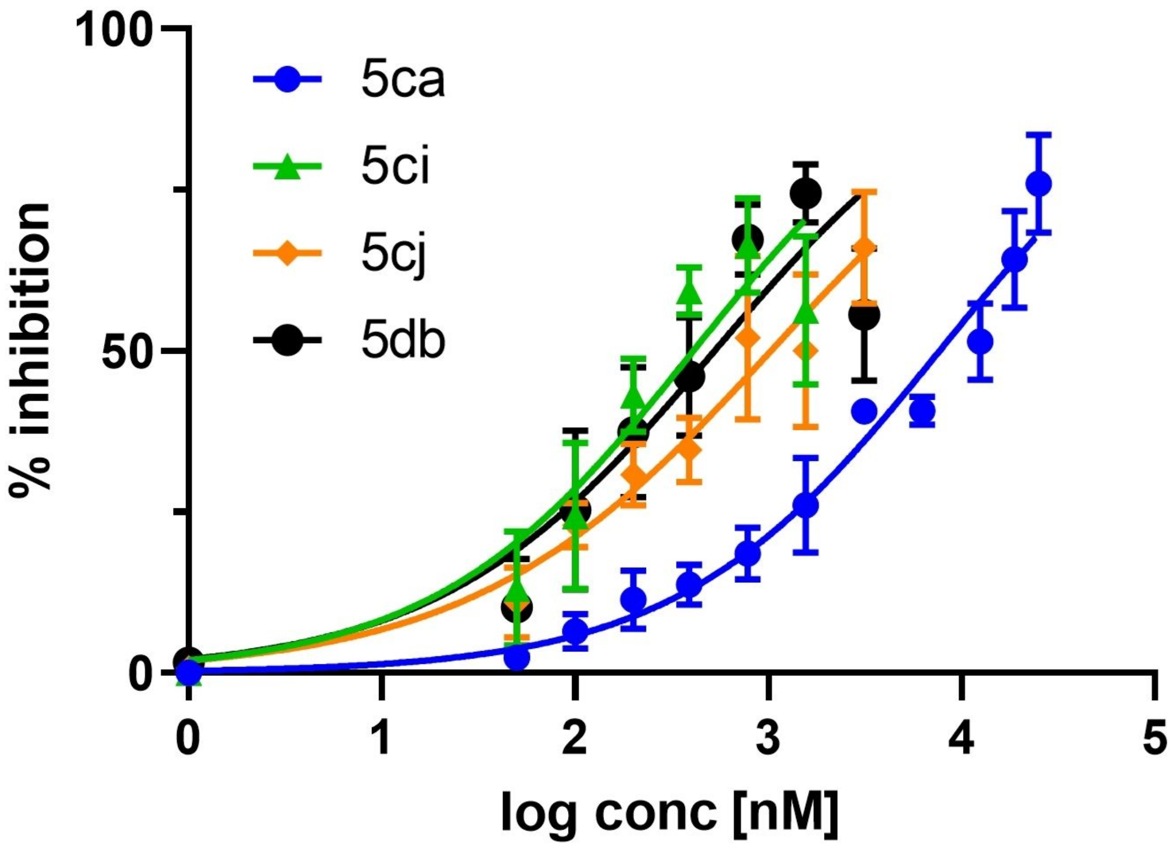

Figure S4. Inhibition of early cell death by 5ca derivatives. Shown is the mean ± SD of three independent experiments.

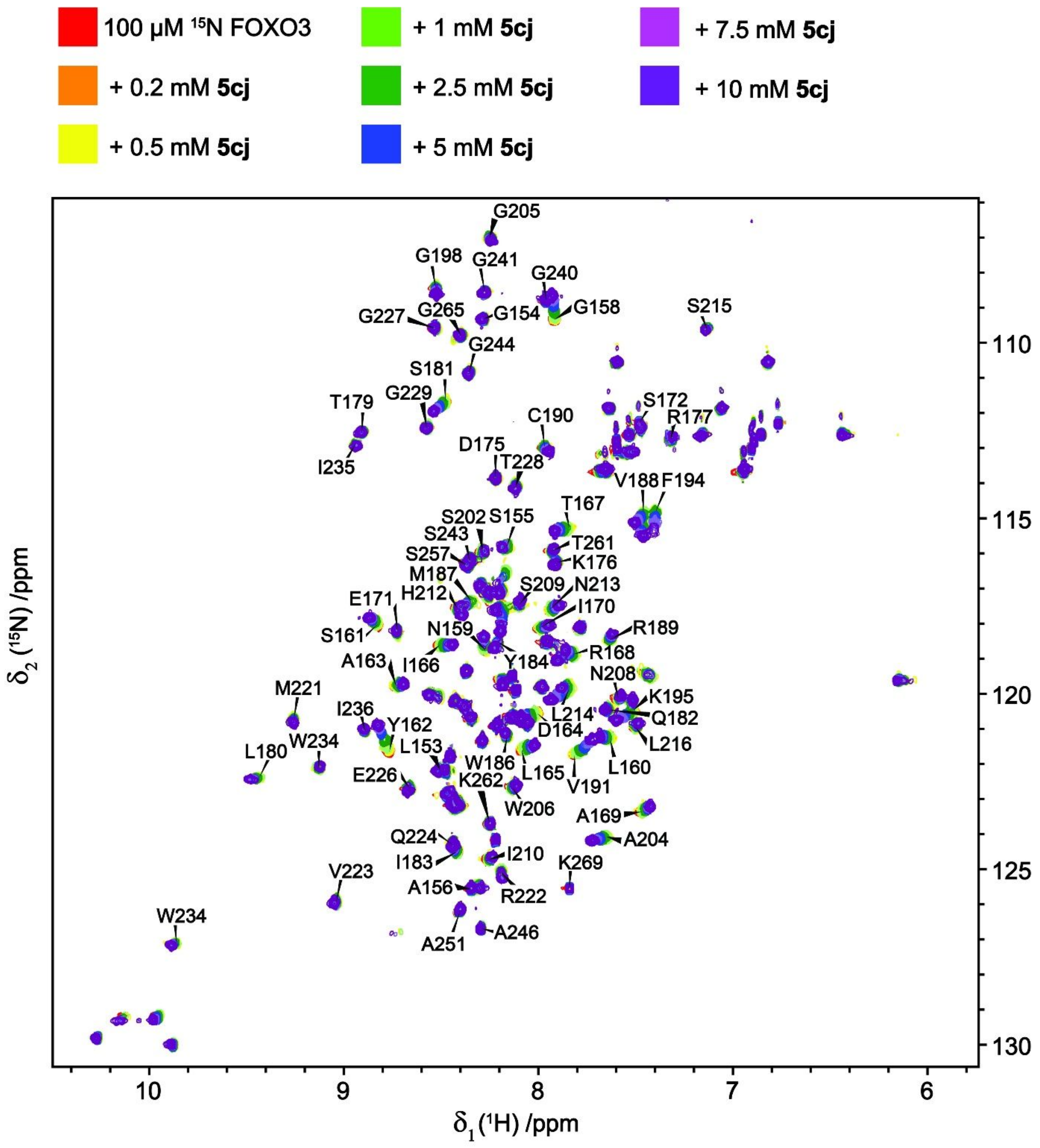

Figure S5. ^{1}H-^{15}N HSQC spectrum of ^{15}N-labeled FOXO3-DBD in the absence and presence of 0.2-10 mM **5cj**.

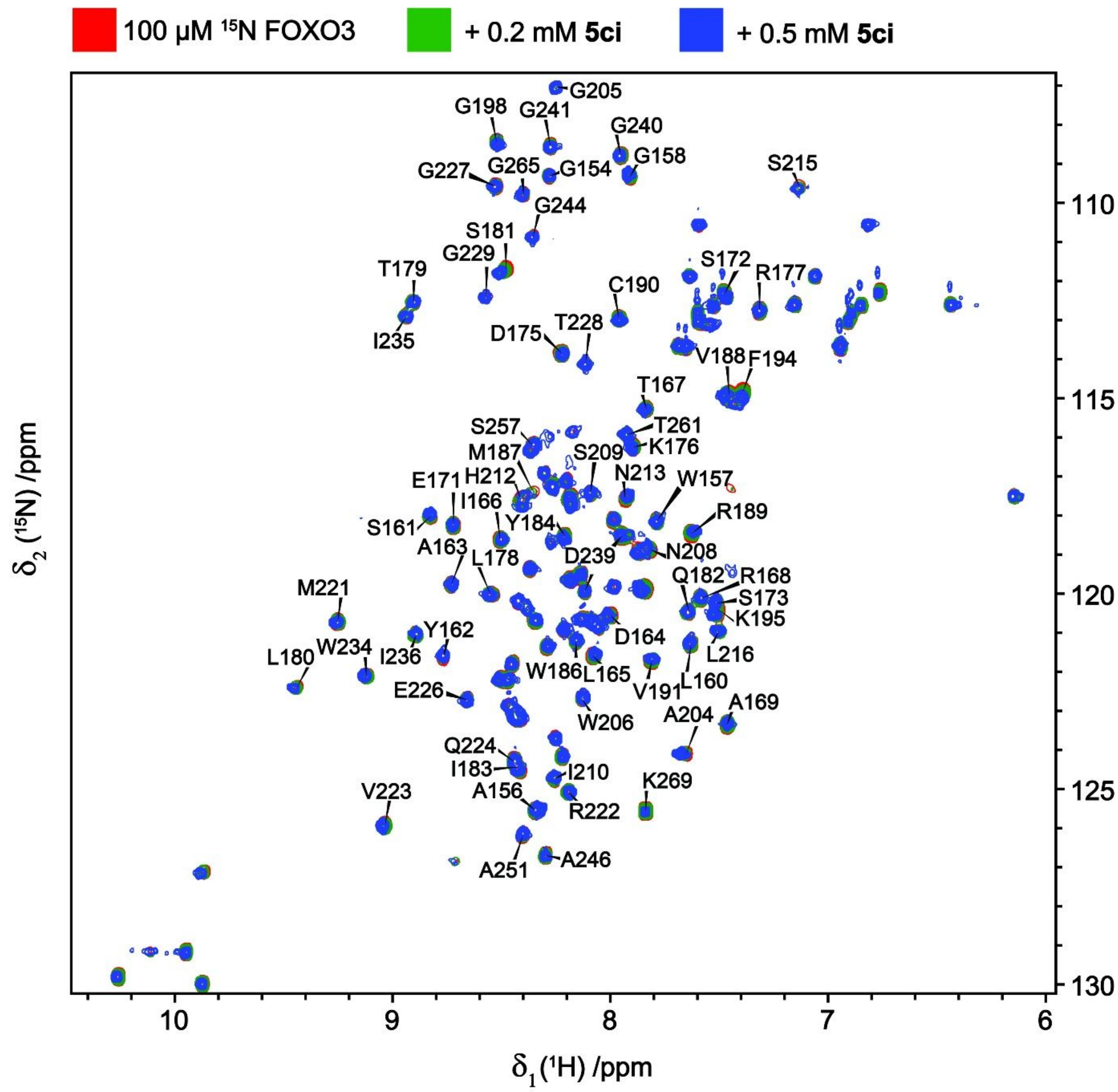

Figure S6. ^{1}H-^{15}N HSQC spectrum of ^{15}N-labeled FOXO3-DBD in the absence and presence of 0.2-0.5 mM **5ci**.

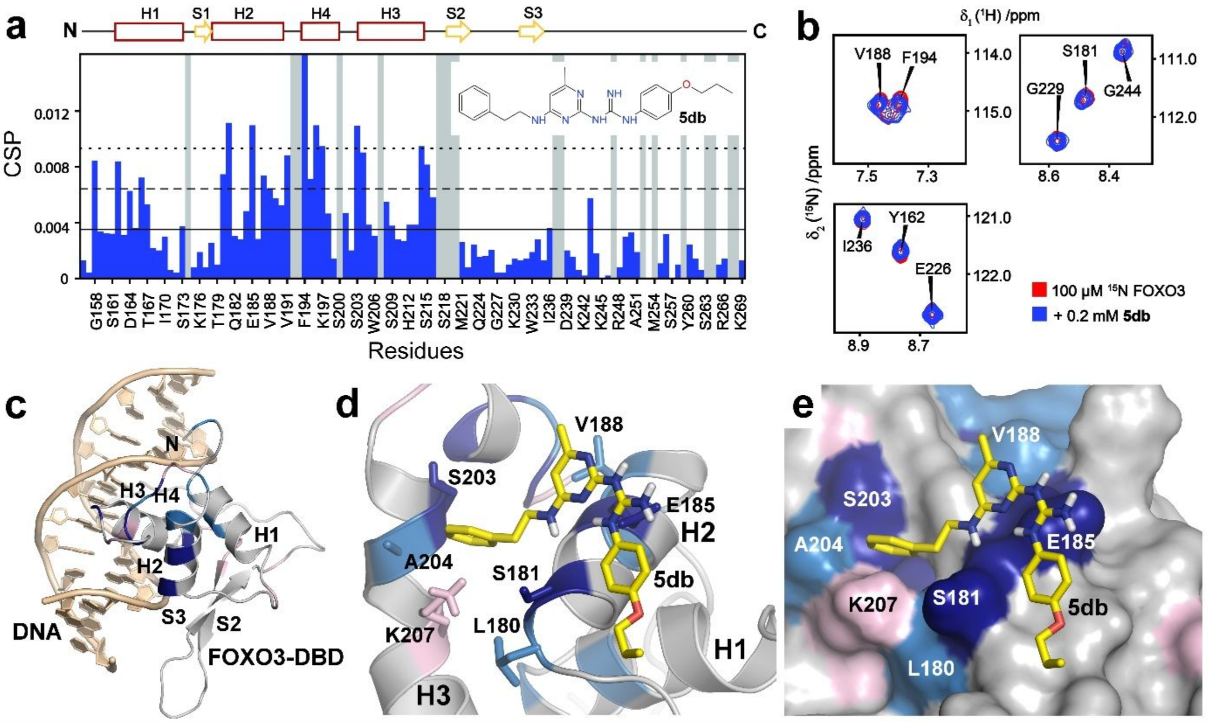

Figure S7. Interaction between FOXO3-DBD and compound 5db. (a) Distribution of CSPs observed in residues of 100 μM ^{15}N-labeled FOXO3-DBD in the presence of 200 μM **5db**. Solid, dashed, and dotted lines correspond to the mean, mean + $1\sigma_{cor}$, and mean + $2\sigma_{cor}$ values of CSPs, respectively. Grey bars represent unassigned residues in ^{1}H-^{15}N HSQC spectra. Secondary structure of FOXO3-DBD is indicated at the top. (b) Detailed view of selected peaks from ^{1}H-^{15}N HSQC spectra of ^{15}N-labeled FOXO3-DBD in the presence of **5db**. (c) CSPs of ^{15}N-labeled FOXO3-DBD in the presence of **5db** mapped onto the crystal structure of the FOXO-DBD:DNA complex. Residues with CSPs larger than the mean + $2\sigma^{0}_{cor}$ and the mean + $1\sigma^{0}_{cor}$ (from panel a) are highlighted in dark blue and light blue, respectively. Residues that could not be unambiguously assigned are highlighted in light pink. (d,e) The top-ranked HADDOCK model of the FOXO3-DBD:**5db** complex. FOXO3-DBD is shown either in ribbon or surface representation. Residues located in close proximity to **5db** are shown as sticks.

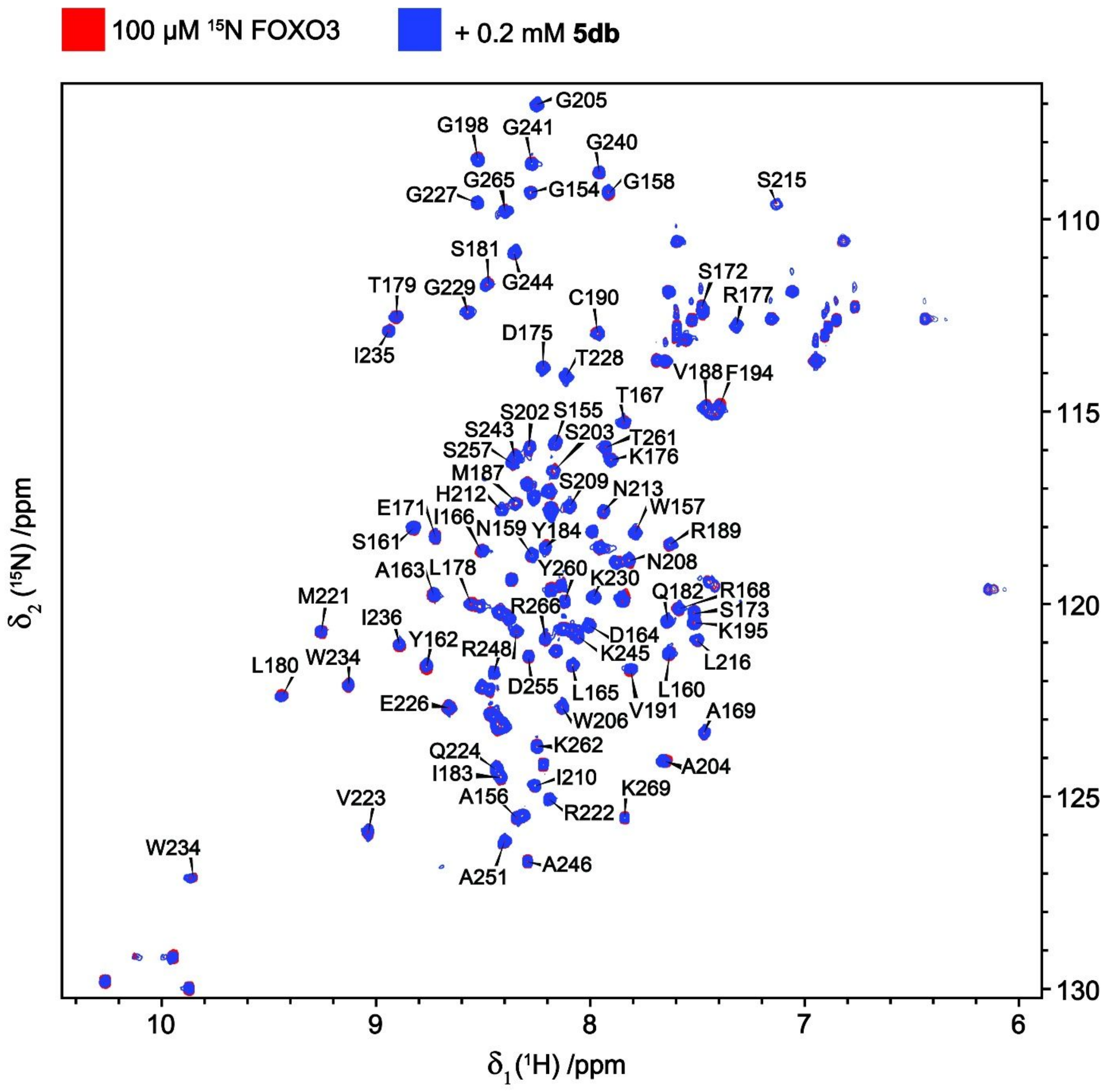

Figure S8. ^{1}H-^{15}N HSQC spectrum of ^{15}N-labeled FOXO3-DBD in the absence and presence of 0.2 mM **5db**.

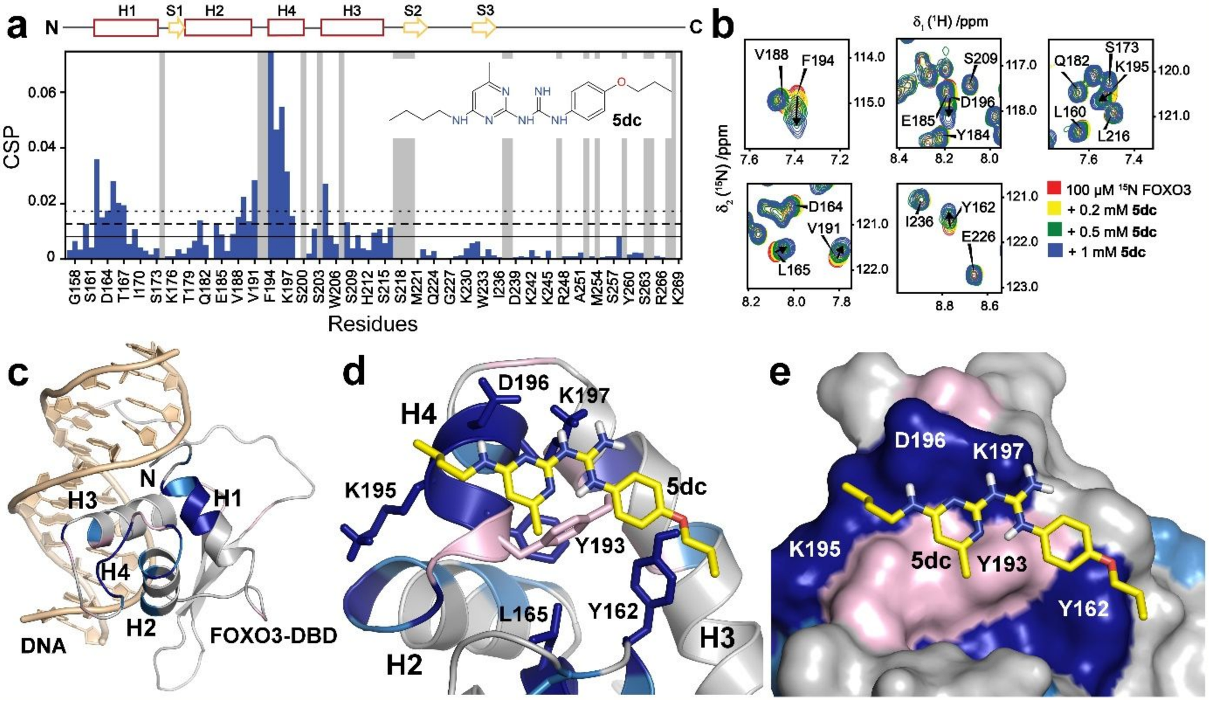

Figure S9. Interaction between FOXO3-DBD and compound 5dc. (a) Distribution of CSPs observed in residues of 100 µM ^{15}N-labeled FOXO3-DBD in the presence of 500 µM **5dc**. Solid, dashed, and dotted lines correspond to the mean, mean + 1σ_{cor}, and mean + 2σ_{cor} values of CSPs, respectively. Grey bars represent unassigned residues in ^{1}H-^{15}N HSQC spectra. Secondary structure of FOXO3-DBD is indicated at the top. (b) Detailed view of selected peaks from ^{1}H-^{15}N HSQC spectra of ^{15}N-labeled FOXO3-DBD in the presence of **5dc**. (c) CSPs of ^{15}N-labeled FOXO3-DBD in the presence of **5dc** mapped onto the crystal structure of the FOXO-DBD:DNA complex. Residues with CSPs larger than the mean + 2σ^0_{cor} and the mean + 1σ^0_{cor} (from panel a) are highlighted in dark blue and light blue, respectively. Residues that could not be unambiguously assigned are highlighted in light pink. (d,e) The top-ranked HADDOCK model of the FOXO3-DBD:**5dc** complex. FOXO3-DBD is shown either in ribbon or surface representation. Residues located in close proximity to **5dc** are shown as sticks.

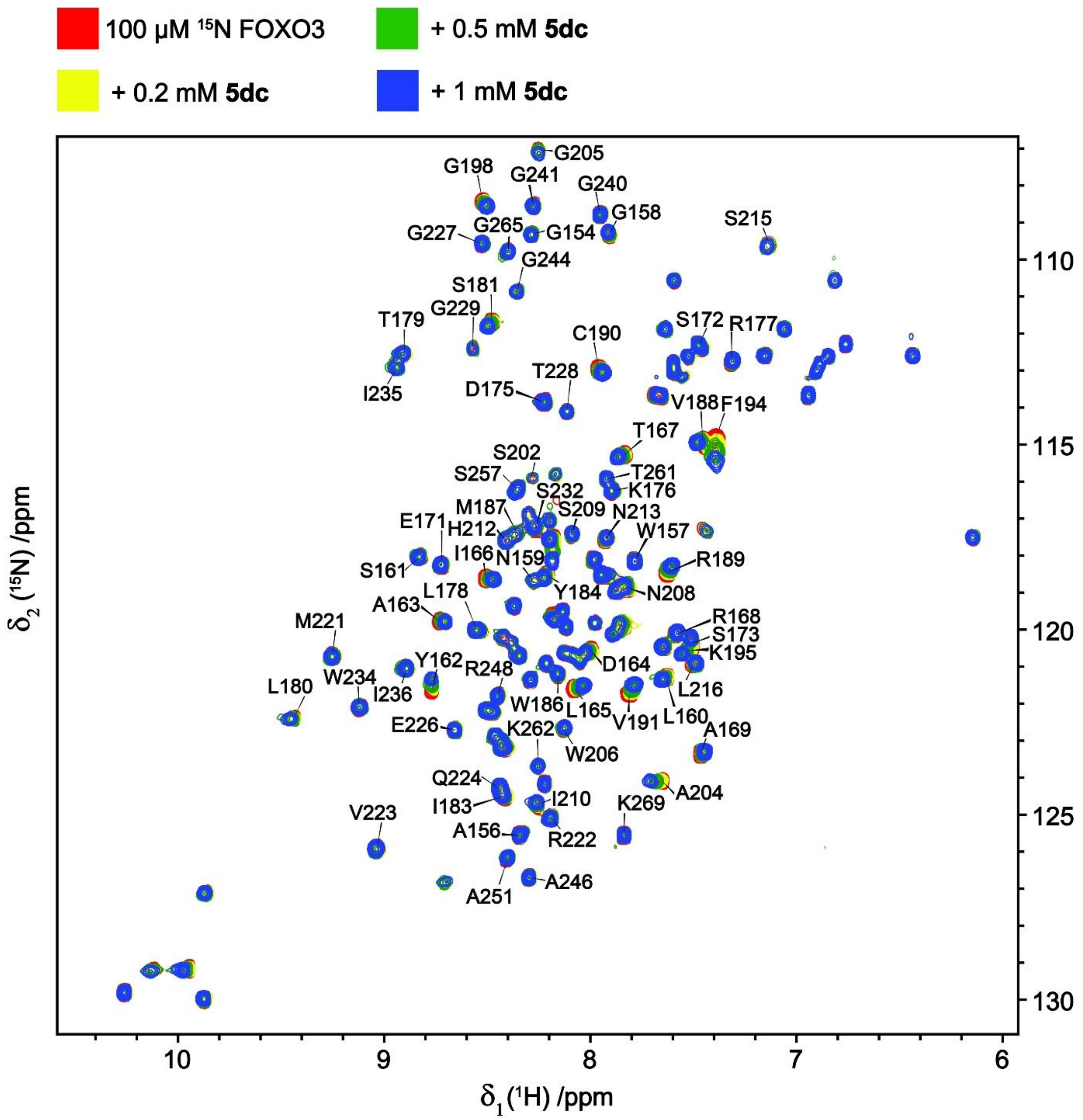

Figure S10. ^{1}H-^{15}N HSQC spectrum of ^{15}N-labeled FOXO3-DBD in the absence and presence of 0.2-1 mM **5dc**.

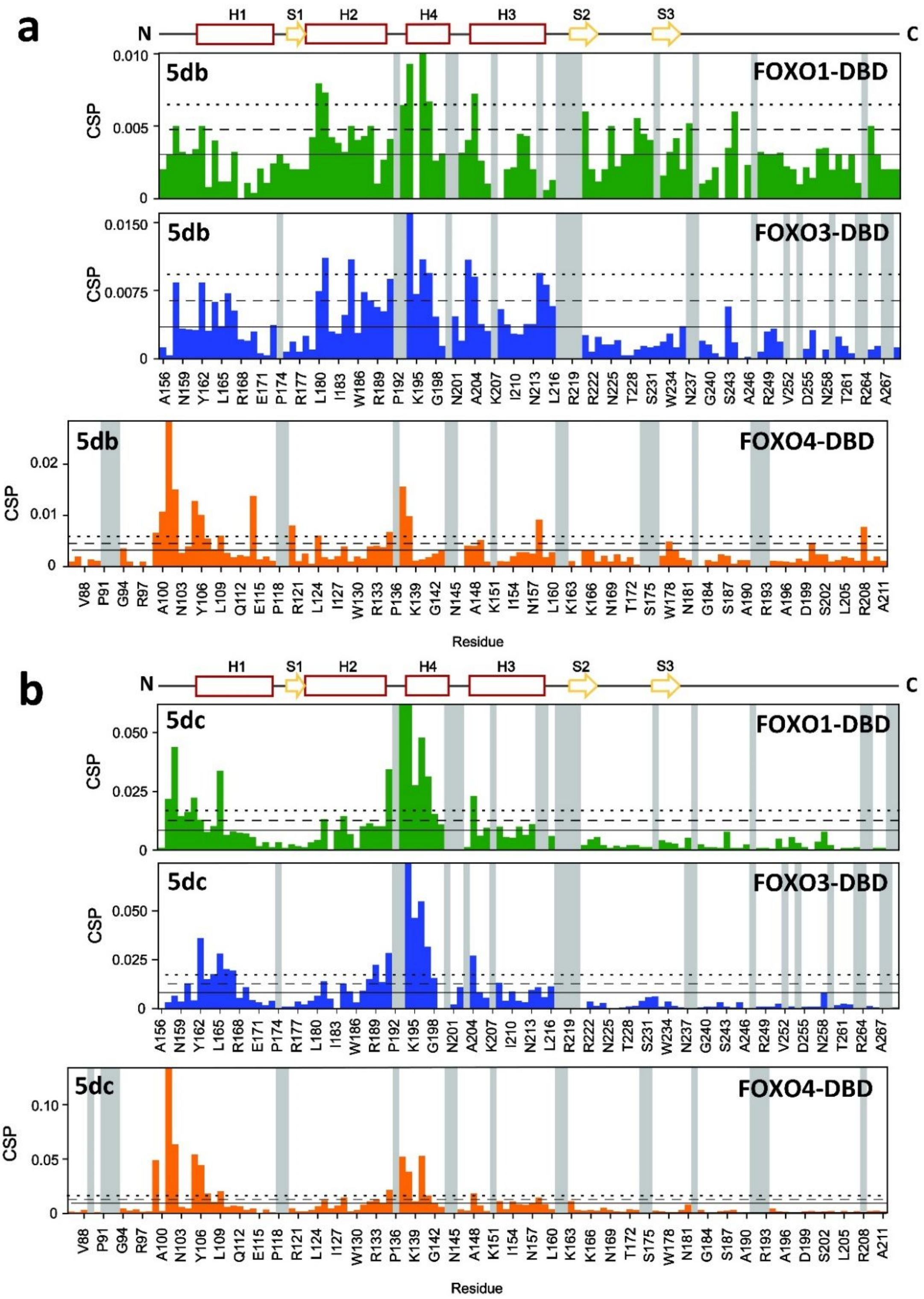

Figure S11. Binding selectivity of compounds 5db and 5dc. Distribution of CSPs observed in residues of 100 μM ^{15}N-labeled FOXO-DBDs in the presence of 200 μM **5db** (a) and 500 μM **5dc** (b). Solid, dashed, and dotted lines correspond to the mean, mean + $1\sigma^0_{cor}$, and mean + $2\sigma^0_{cor}$ values of CSPs, respectively. Grey bars represent unassigned residues in ^{1}H-^{15}N HSQC spectra. Secondary structure of FOXO3-DBD is indicated at the top.

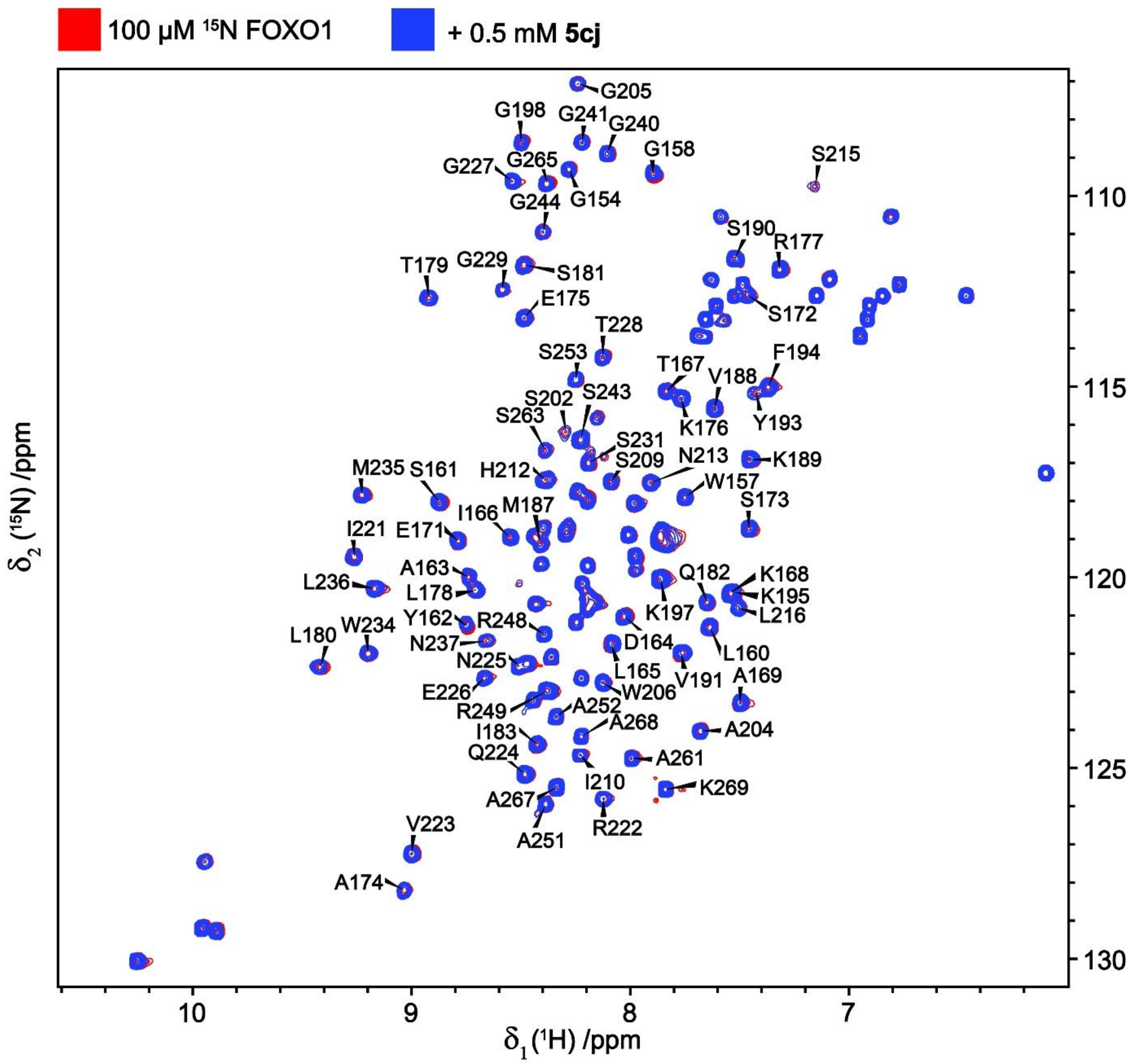

Figure S12. ^{1}H-^{15}N HSQC spectrum of 100 μM ^{15}N-labeled FOXO1-DBD in the absence and presence of 500 μM **5cj**.

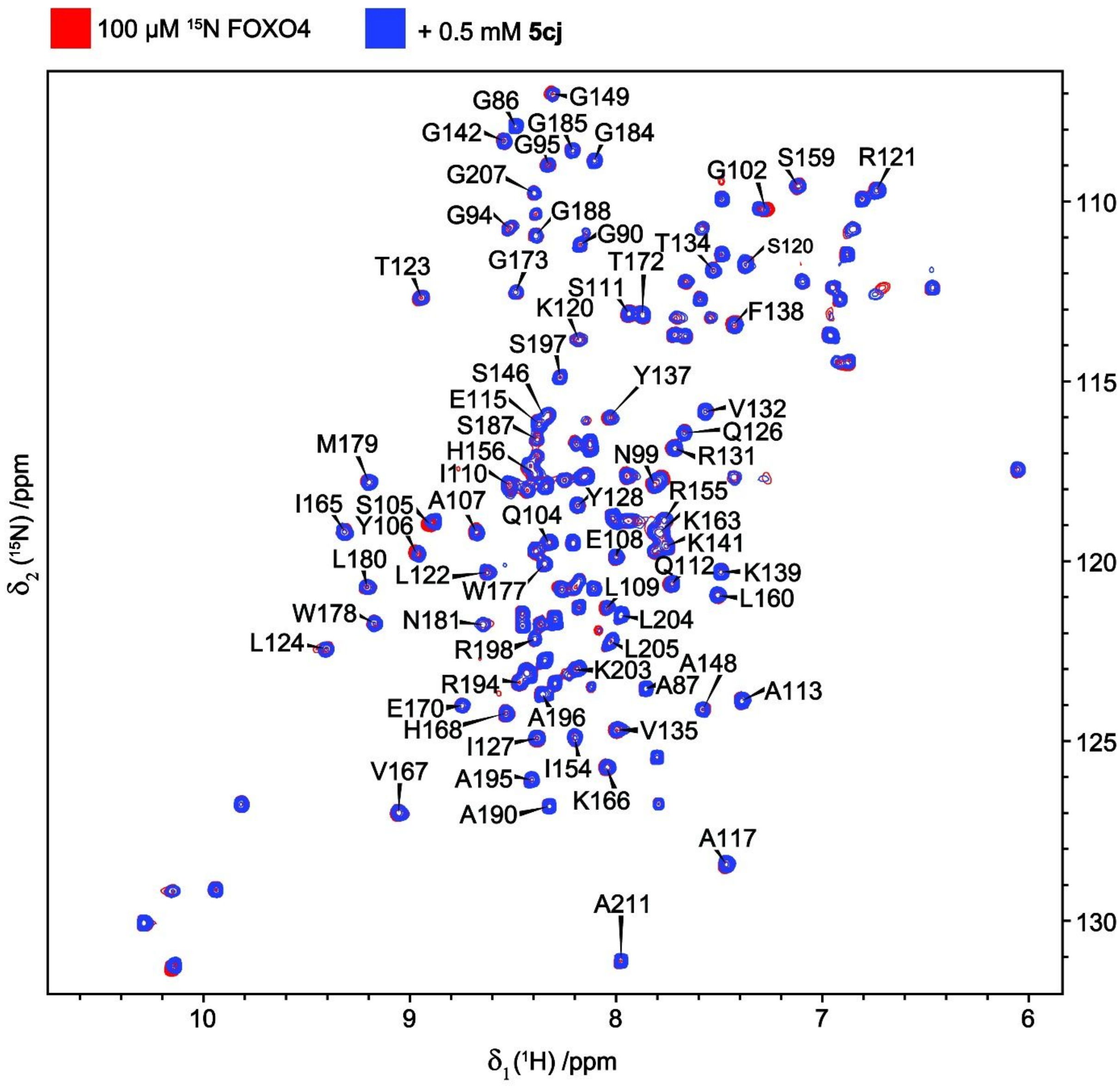

Figure S13. ^{1}H-^{15}N HSQC spectrum of 100 μM ^{15}N-labeled FOXO4-DBD in the absence and presence of 500 μM **5cj**.

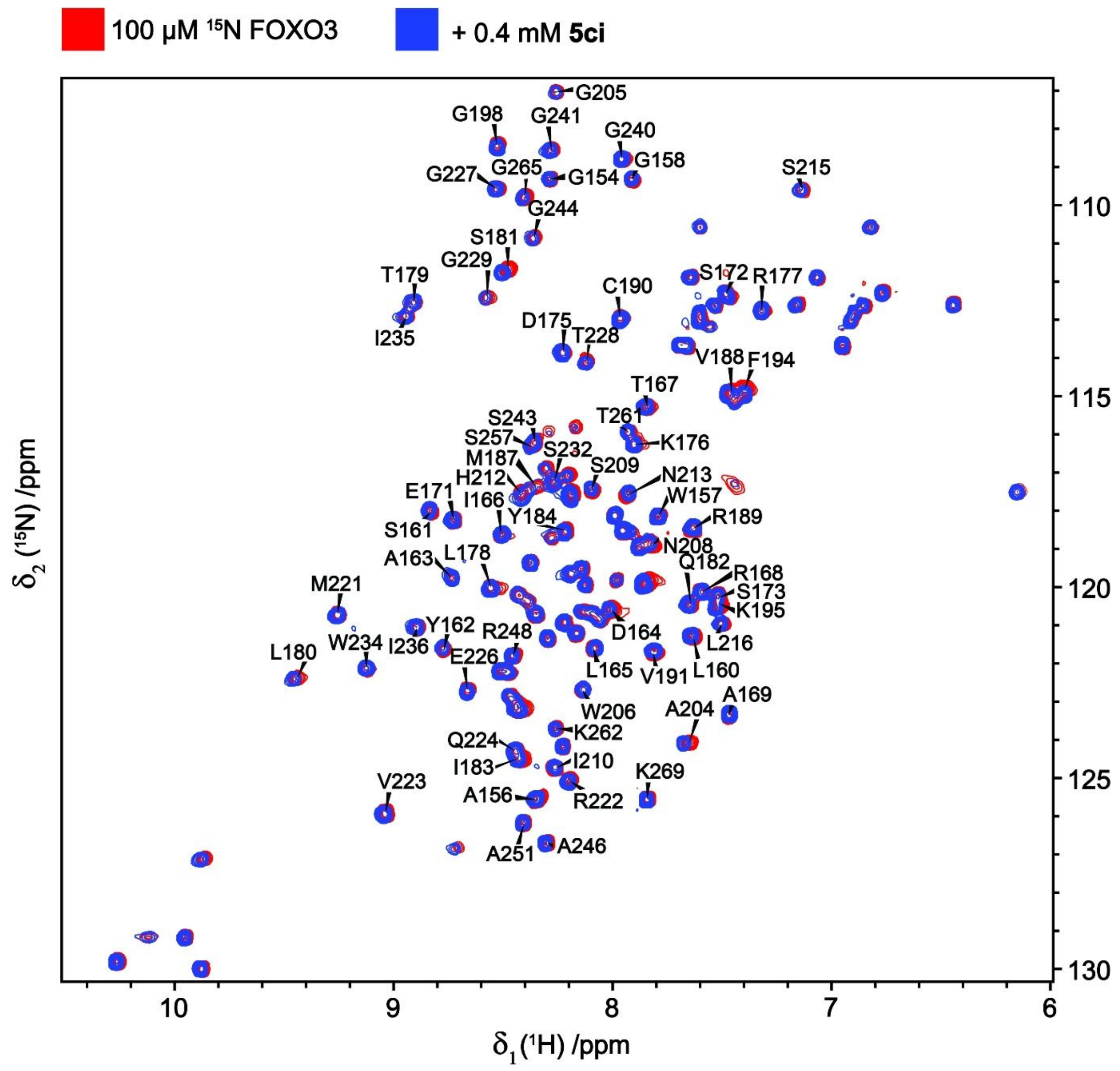

Figure S14. ^{1}H-^{15}N HSQC spectrum of 100 μM ^{15}N-labeled FOXO3-DBD in the absence and presence of 400 μM **5ci**.

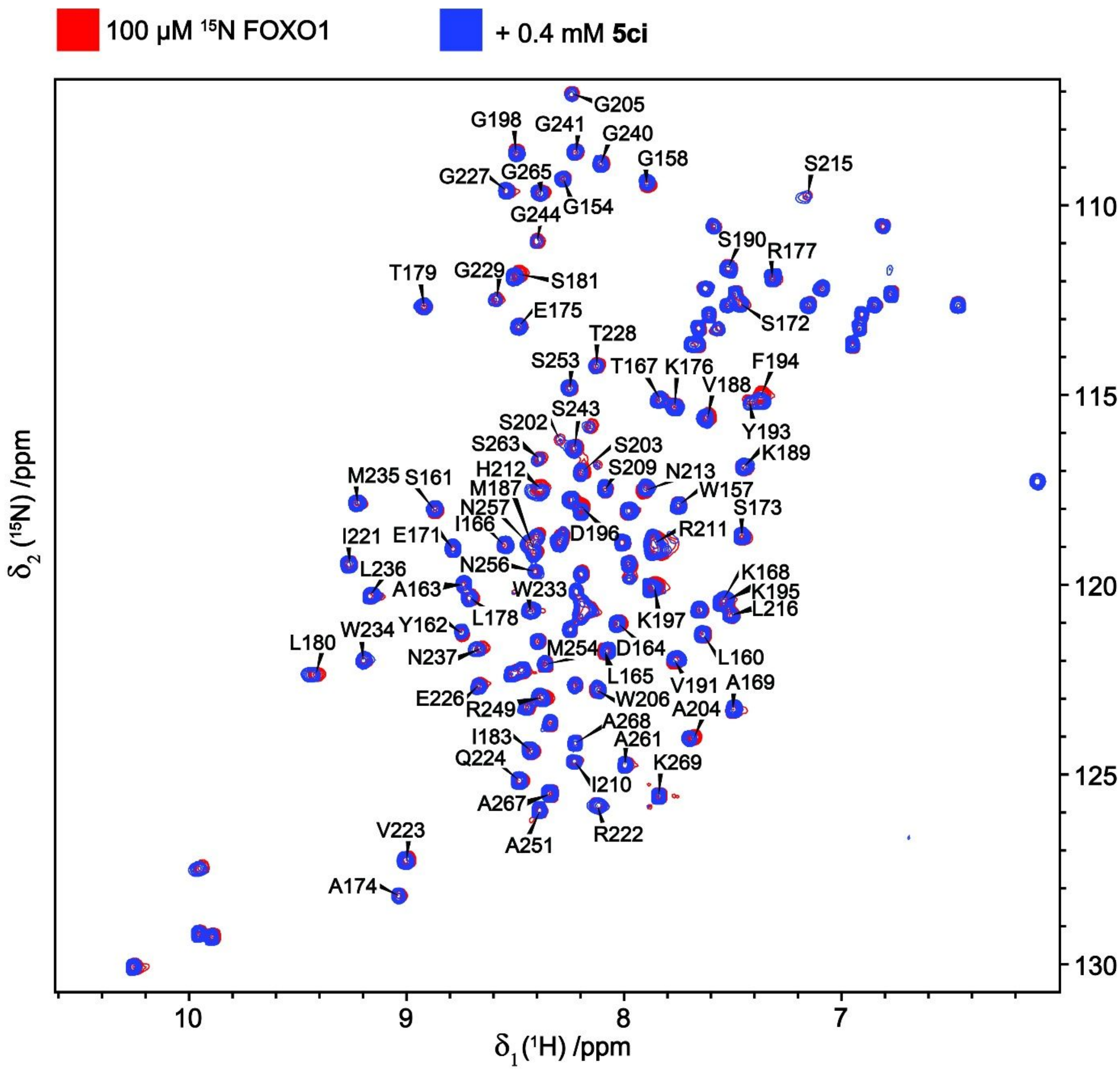

Figure S15. ^1H-^{15}N HSQC spectrum of 100 μM ^{15}N-labeled FOXO1-DBD in the absence and presence of 400 μM **5ci**.

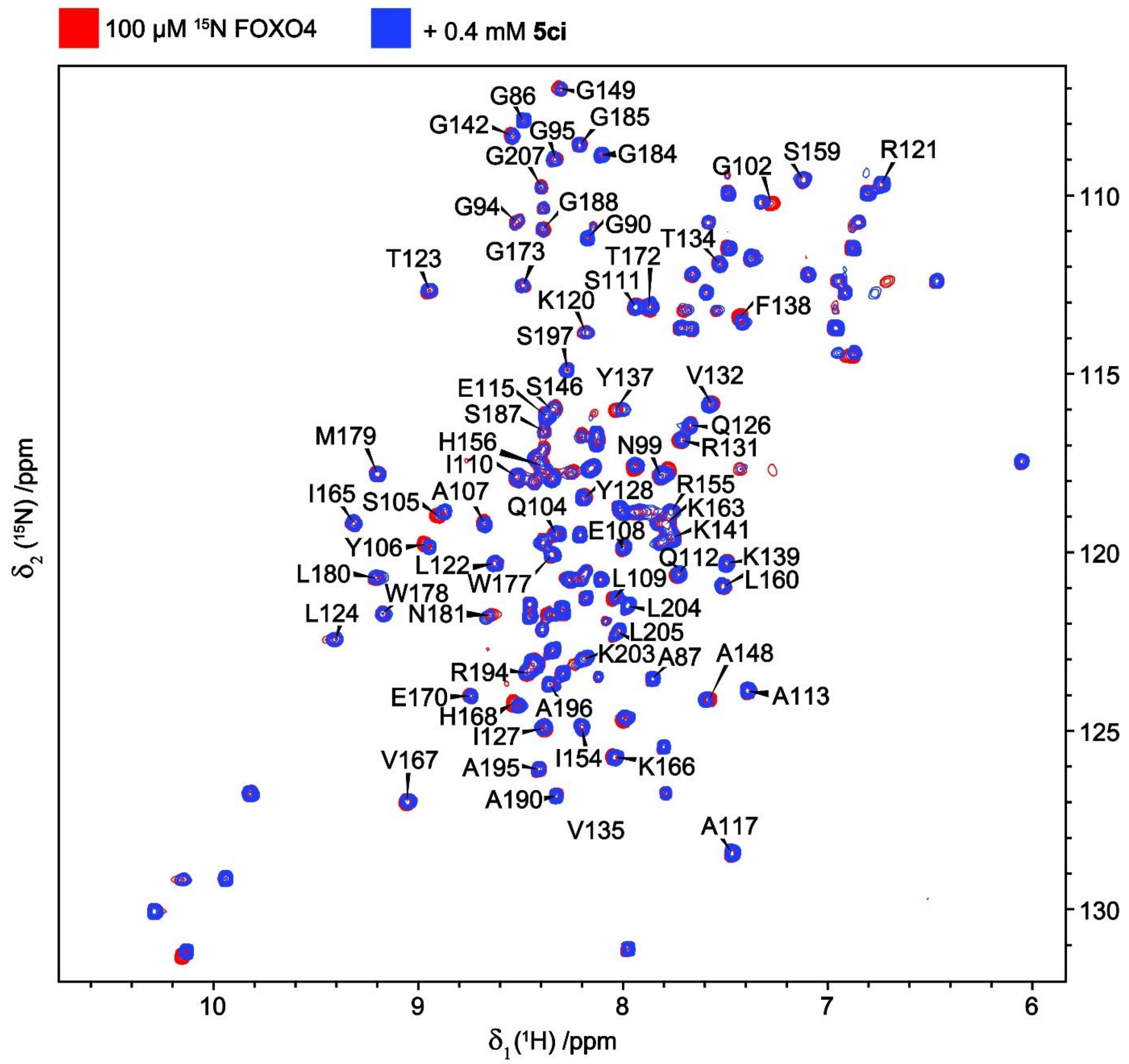

Figure S16. ^{1}H-^{15}N HSQC spectrum of 100 µM ^{15}N-labeled FOXO4-DBD in the absence and presence of 400 µM **5ci**.

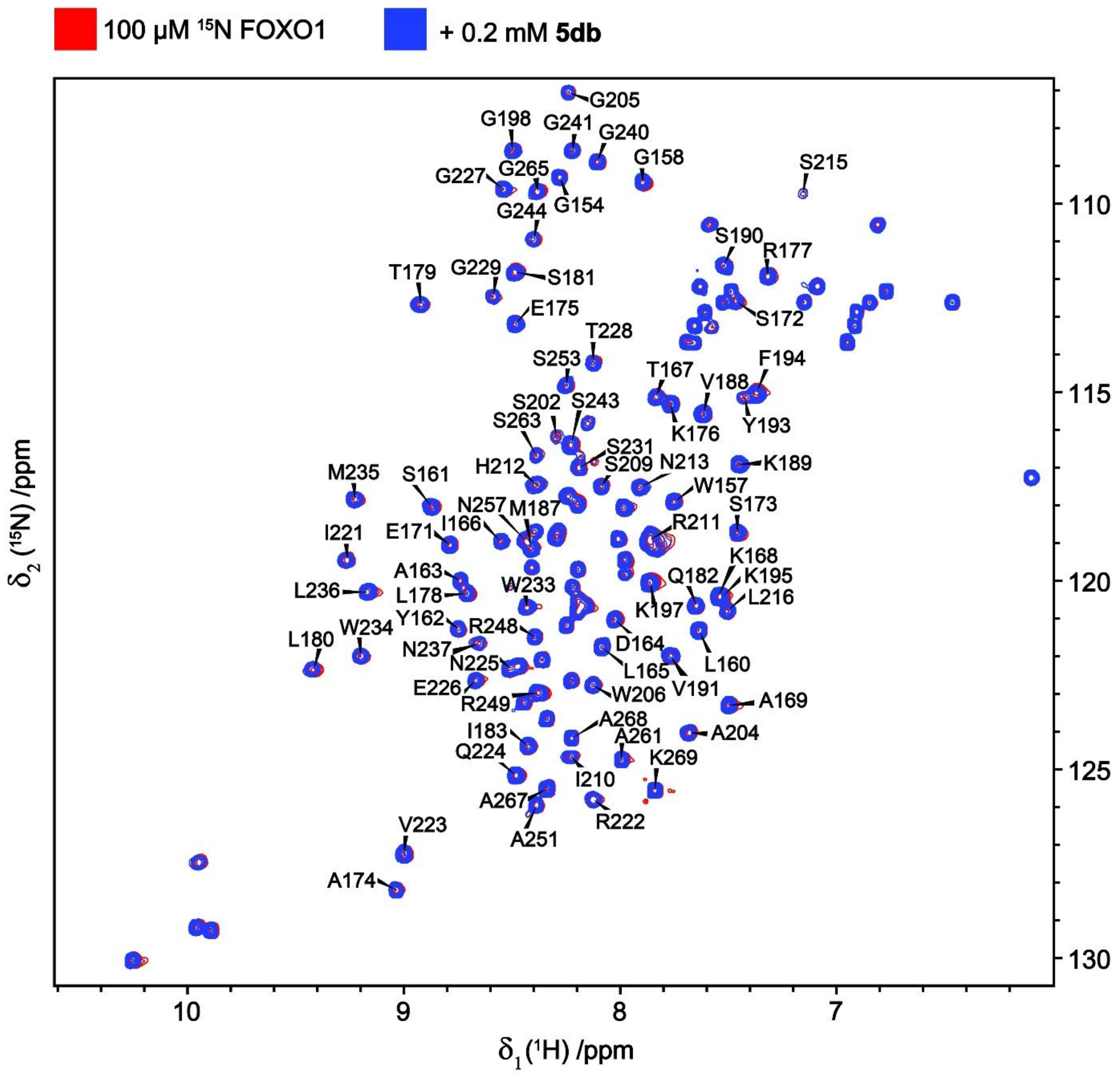

Figure S17. ^{1}H-^{15}N HSQC spectrum of 100 μM ^{15}N-labeled FOXO1-DBD in the absence and presence of 200 μM **5db**.

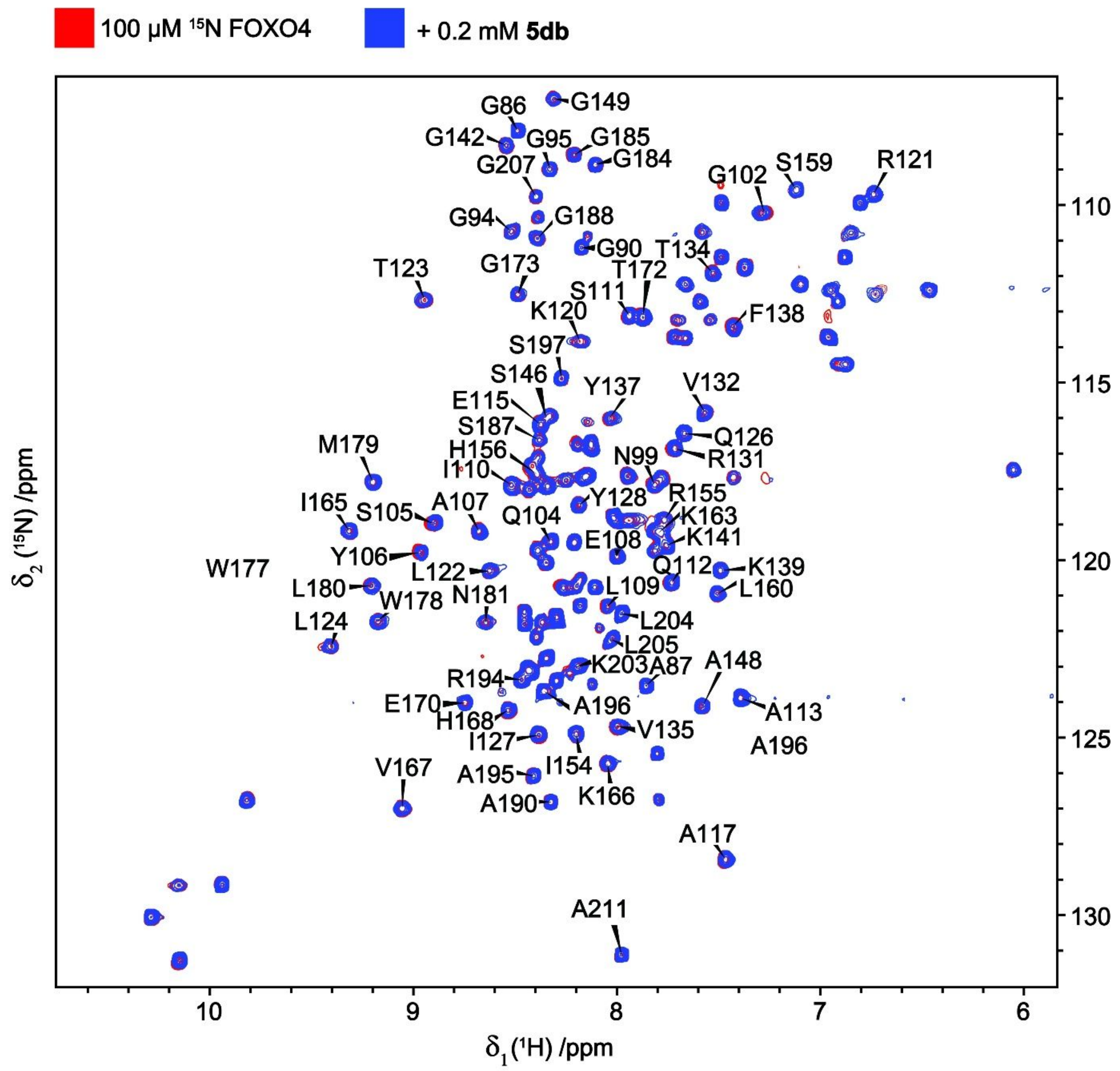

Figure S18. ^{1}H-^{15}N HSQC spectrum of 100 μM ^{15}N-labeled FOXO4-DBD in the absence and presence of 200 μM **5db**.

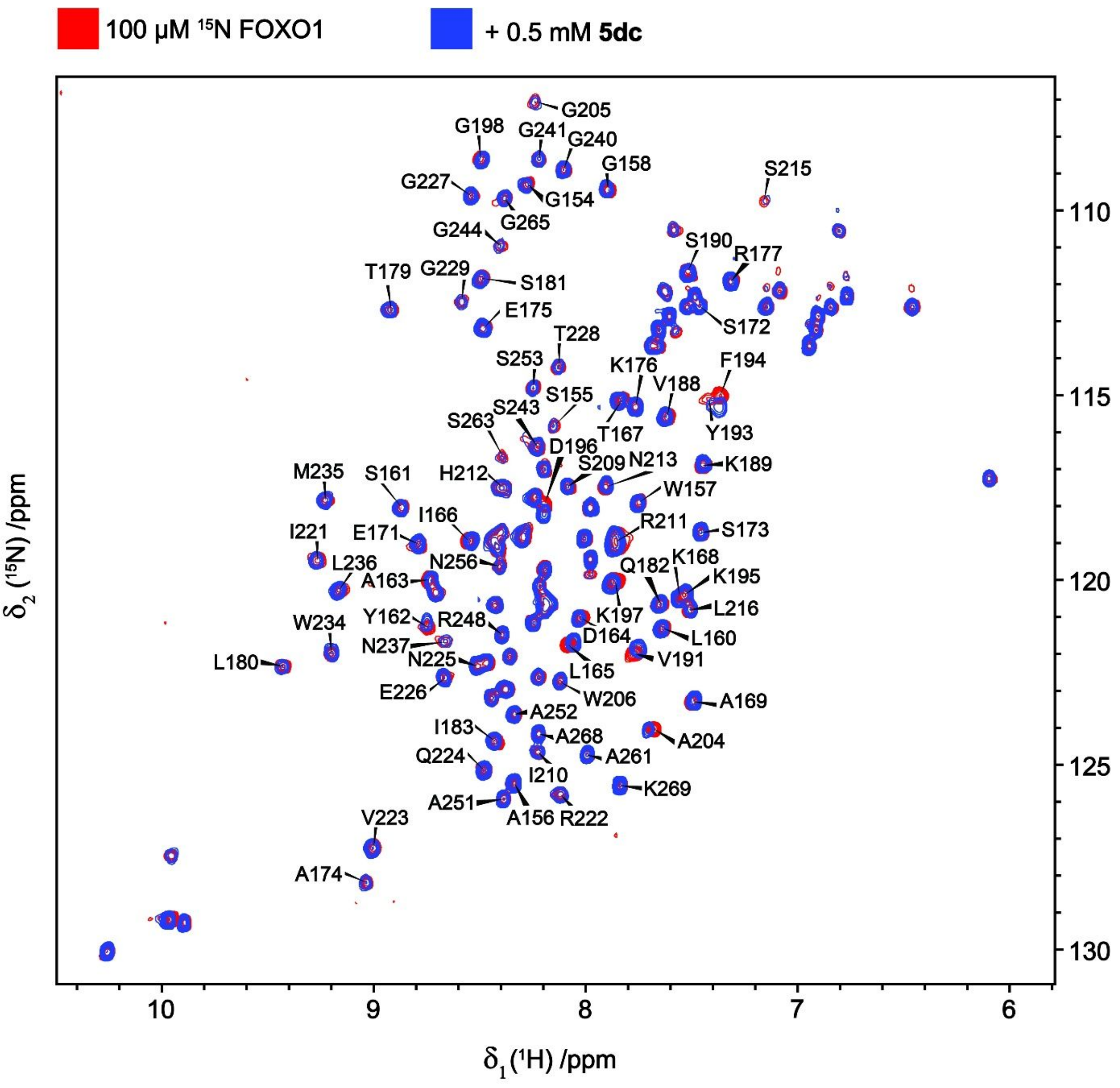

Figure S19. ^{1}H-^{15}N HSQC spectrum of 100 μM ^{15}N-labeled FOXO1-DBD in the absence and presence of 500 μM **5dc**.

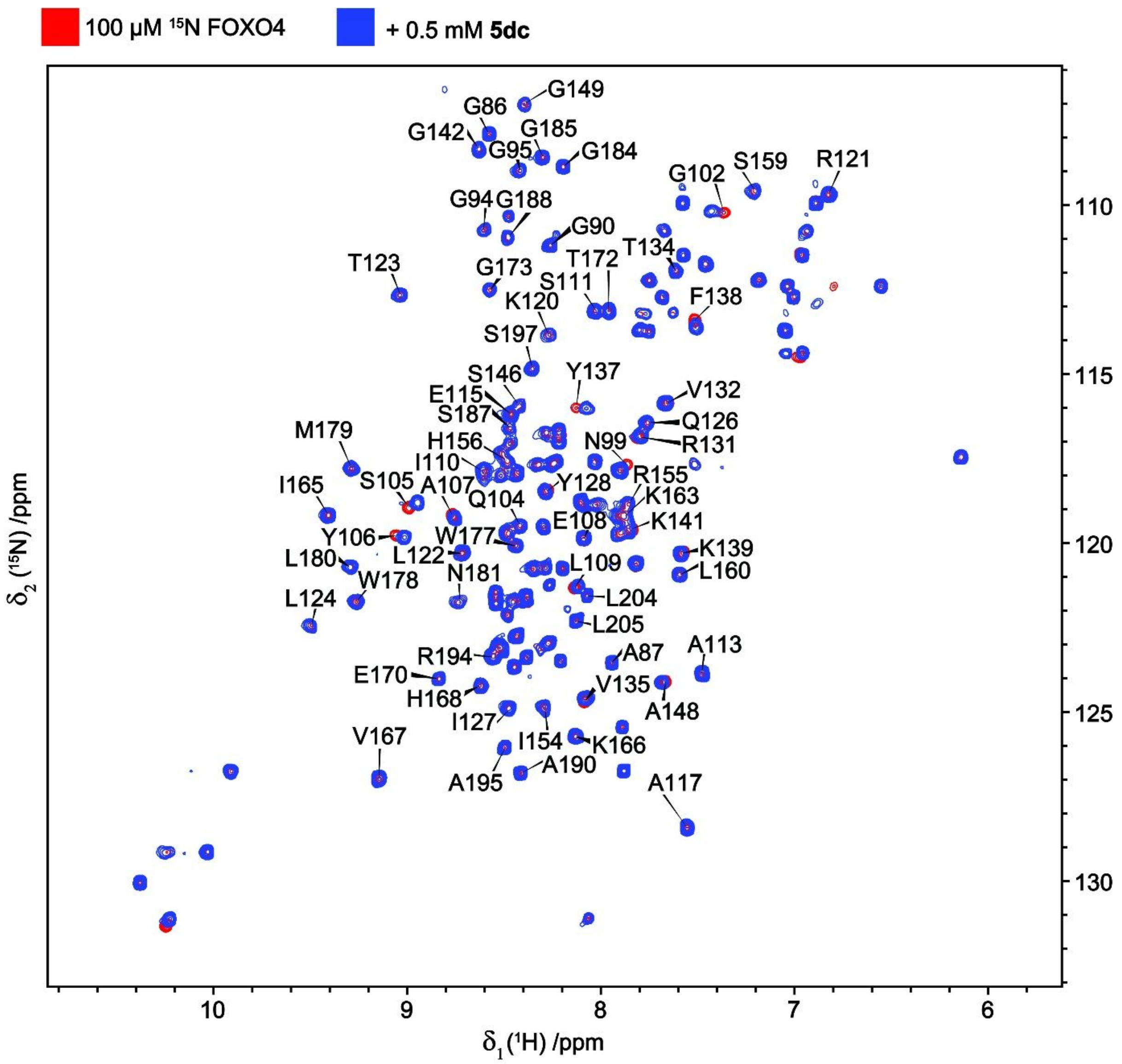

Figure S20. ^{1}H-^{15}N HSQC spectrum of 100 μM ^{15}N-labeled FOXO4-DBD in the absence and presence of 500 μM **5dc**.

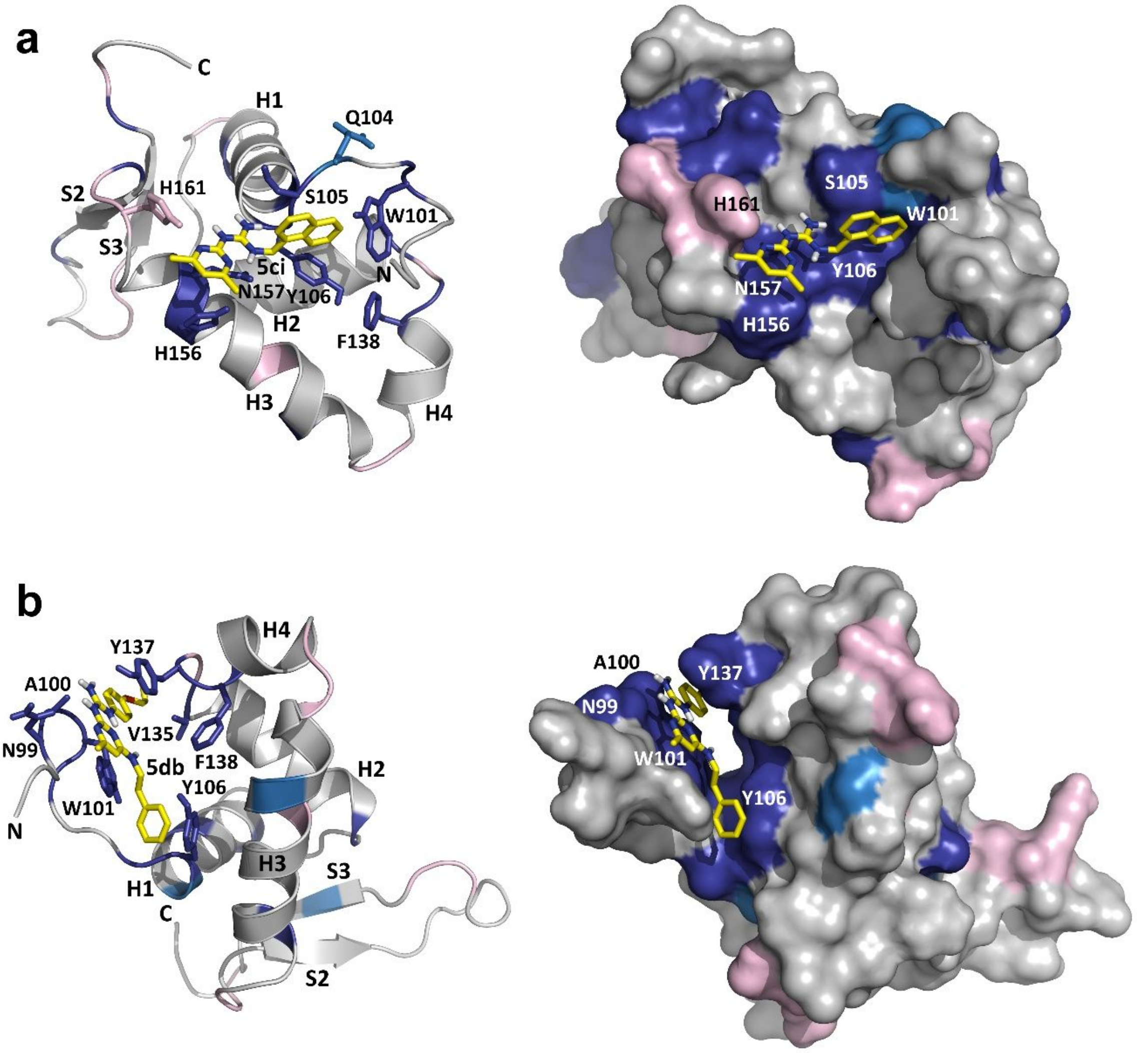

Figure S21. HADDOCK models of FOXO4-DBD complexes. (a) The top-ranked HADDOCK model of the FOXO4-DBD:**5ci** complex. FOXO4-DBD is shown either in ribbon (left) or surface (right) representation. Residues located in close proximity to **5ci** are shown as sticks. (b) (a) The top-ranked HADDOCK model of the FOXO4-DBD:**5db** complex. FOXO4-DBD is shown either in ribbon (left) or surface (right) representation. Residues located in close proximity to **5db** are shown as sticks.

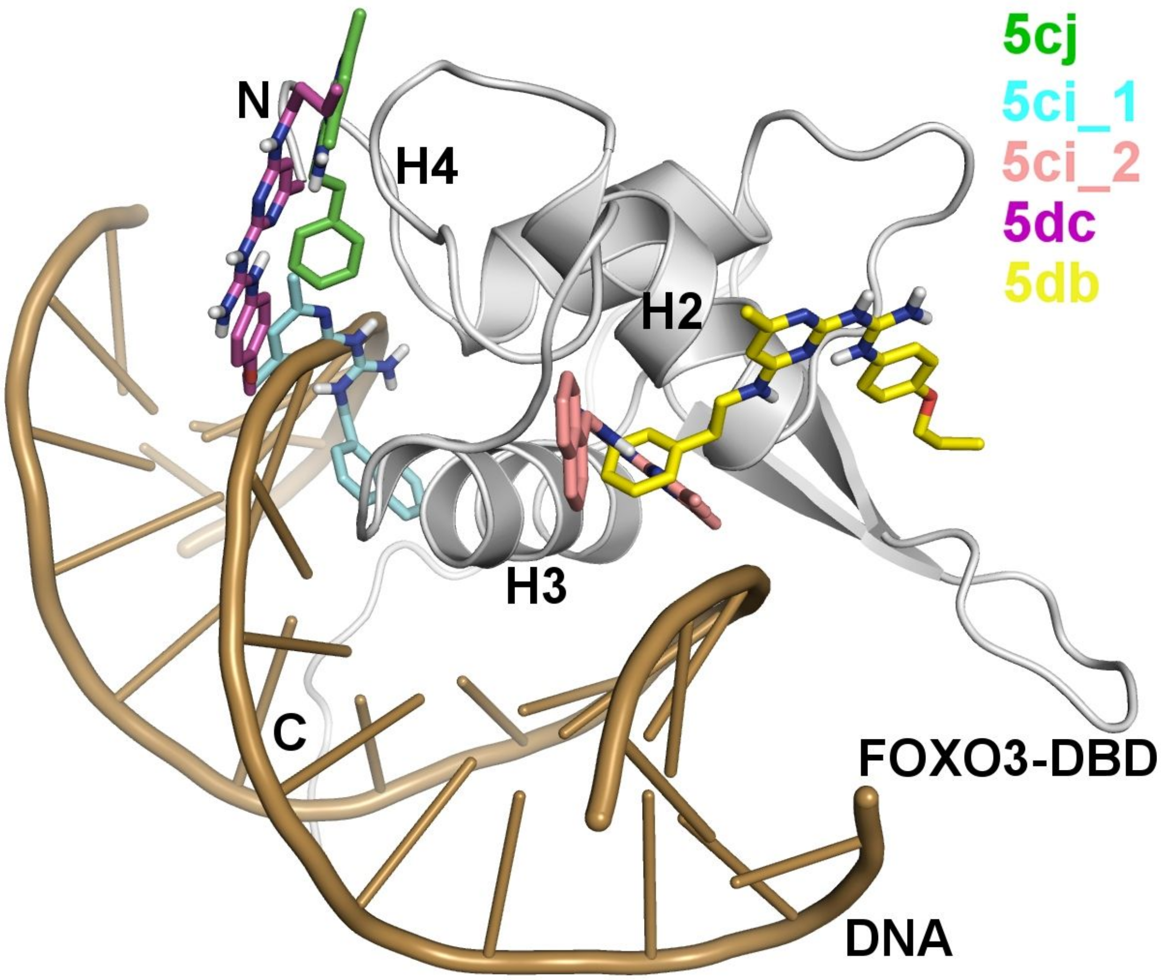

Figure S22. Comparison of HADDOCK models of FOXO3-DBD complexes with 5ci, 5cj, 5db and 5dc. HADDOCK poses were superimposed to the crystal structure of the FOXO3-DBD:DNA complex {Tsai, 2007 #6189}.

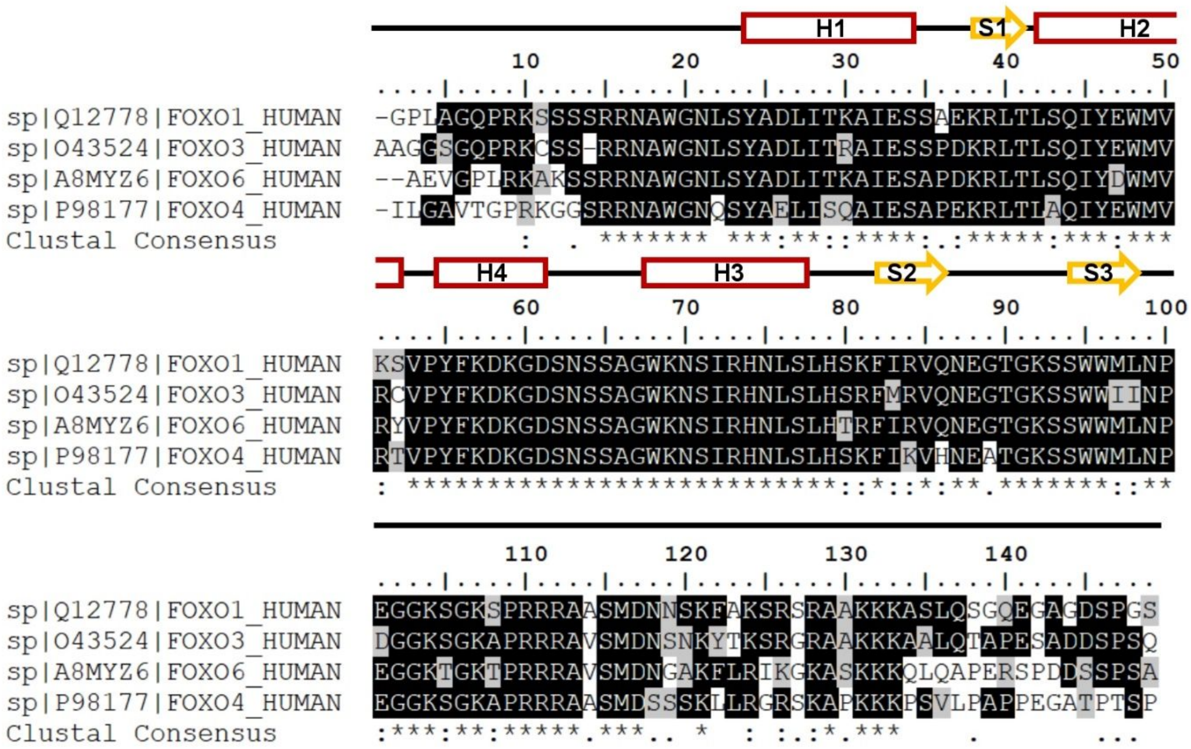

Figure S23. Sequence alignment of human FOXO subgroup members using the CLUSTALW server (https://www.genome.jp/tools-bin/clustalw). The secondary structure is shown at the top.

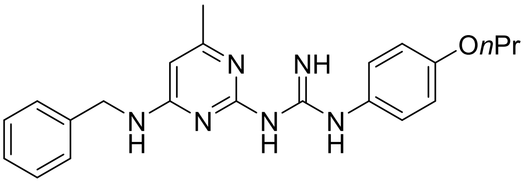

5da: t_R = 6.5 min

HPLC purity = 99%

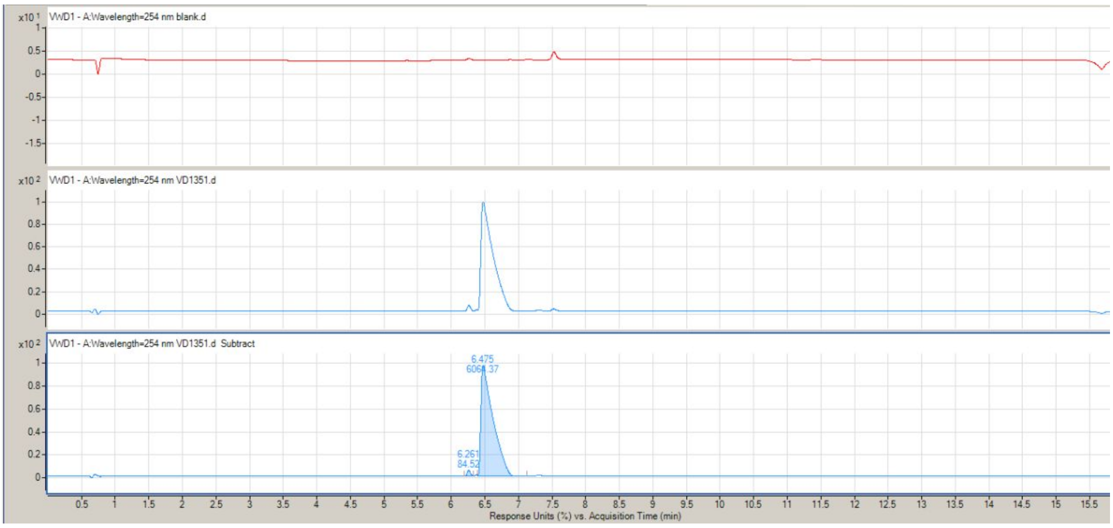

Figure S24. HPLC chromatogram of **5da**.

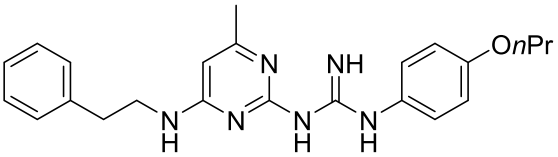

5db: t_R = 6.8 min

HPLC purity = 97%

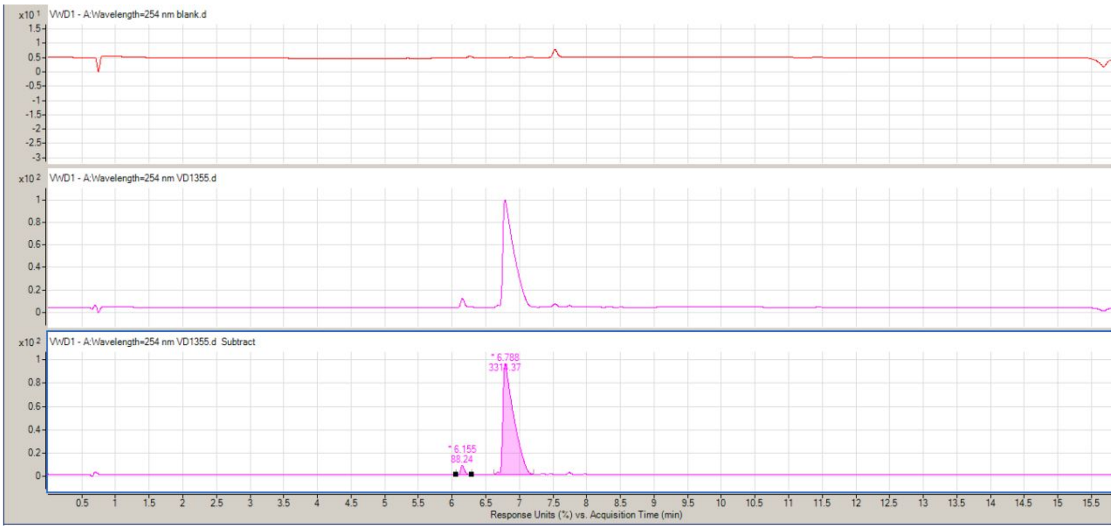

Figure S25. HPLC chromatogram of **5db**.

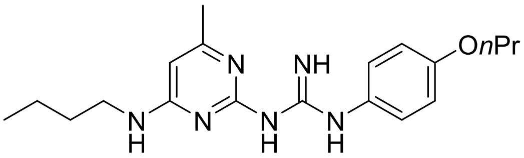

5dc: t_R = 6.6 min

HPLC purity ≥ 99%

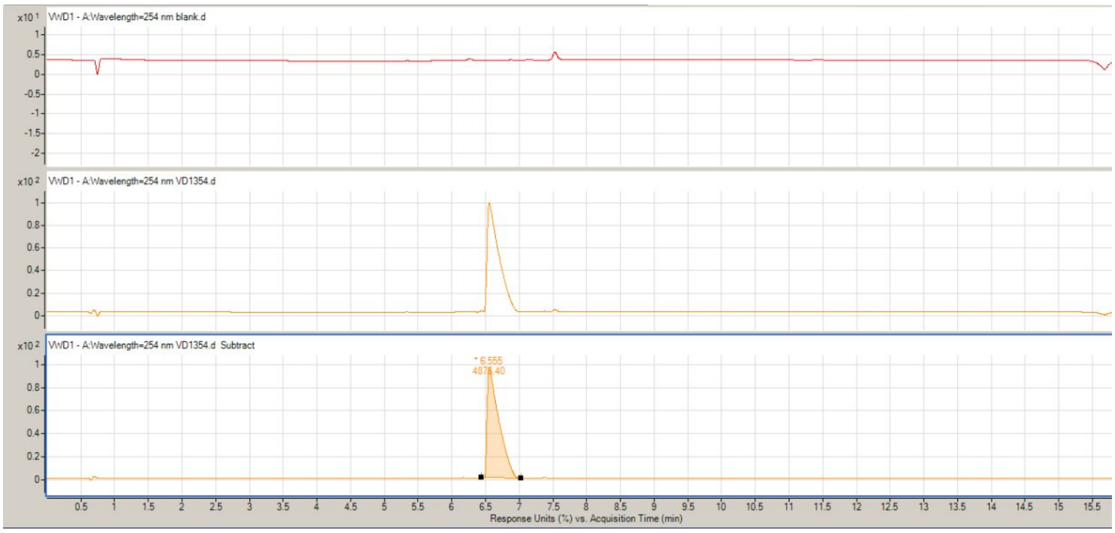

Figure S26. HPLC chromatogram of **5dc**.

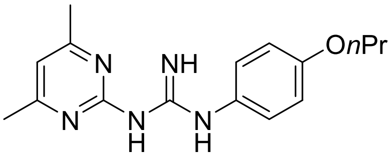

5ca: t_R = 5.8 min

HPLC purity = 99%

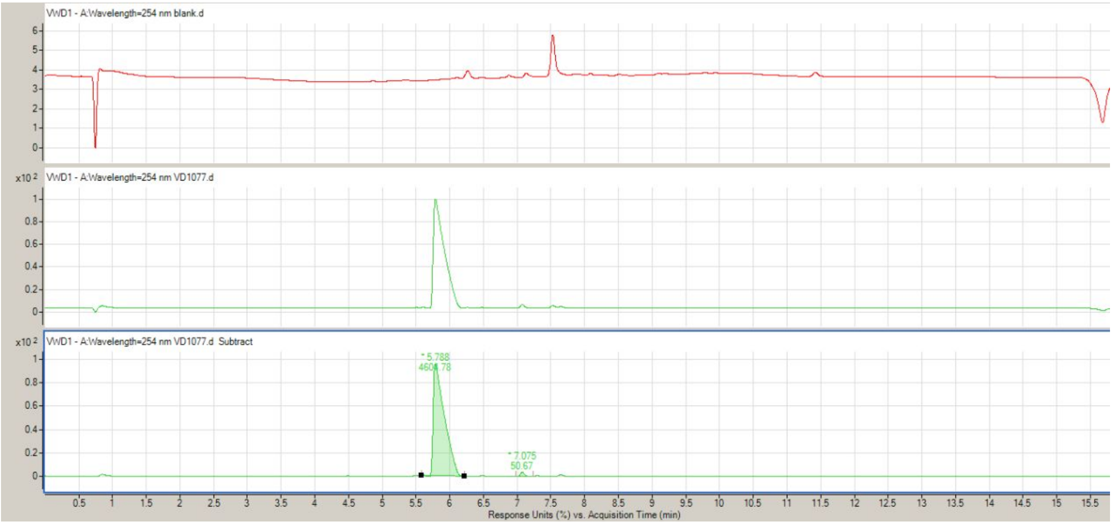

Figure S27. HPLC chromatogram of **5ca**.

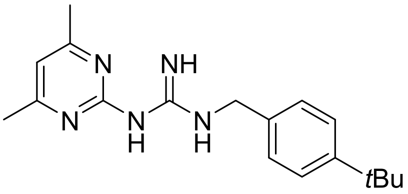

5cg: $t_R = 6.5$ min

HPLC purity = 99%

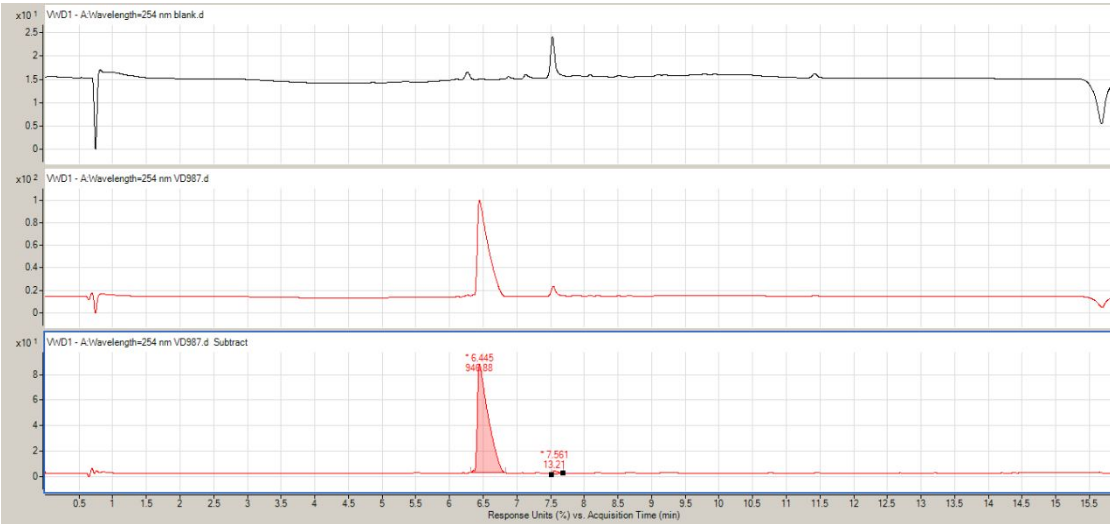

Figure S28. HPLC chromatogram of **5cg**.

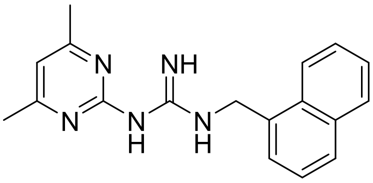

5ci: $t_R = 6.0$ min

HPLC purity = 99%

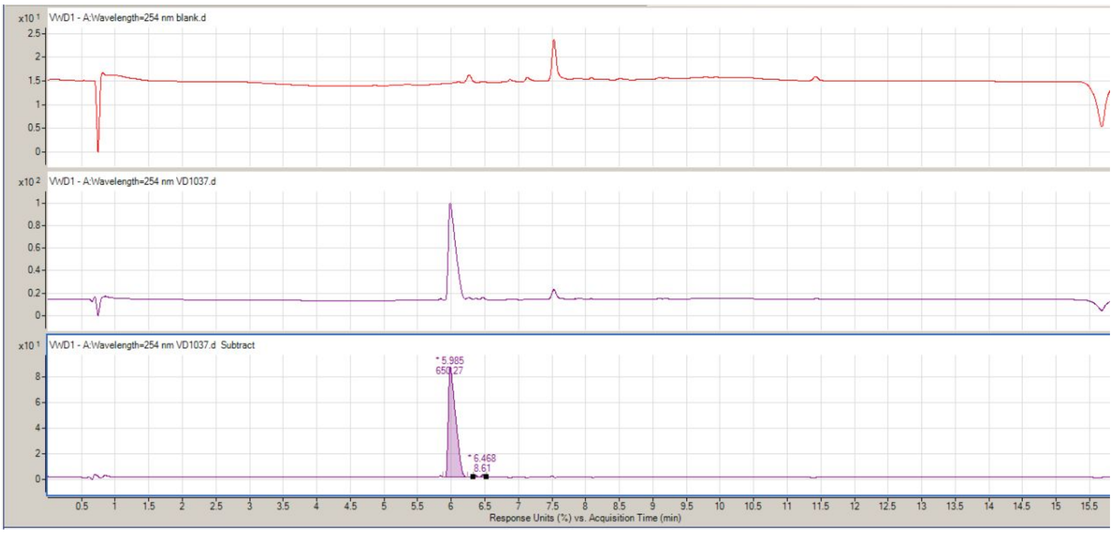

Figure S29. HPLC chromatogram of **5ci**.

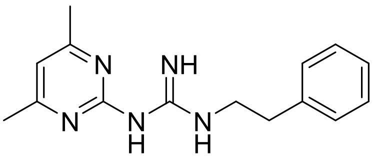

5cj: t_R = 5.4 min
HPLC purity = 98%

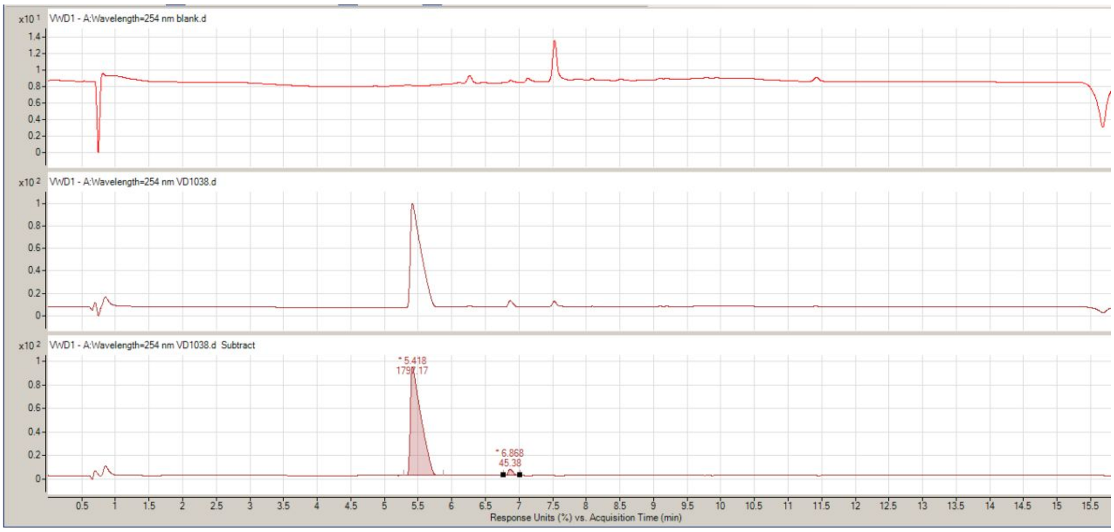

Figure S30. HPLC chromatogram of **5cj**.

^{1}H NMR of **3a** (400 MHz, DMSO-d_6)

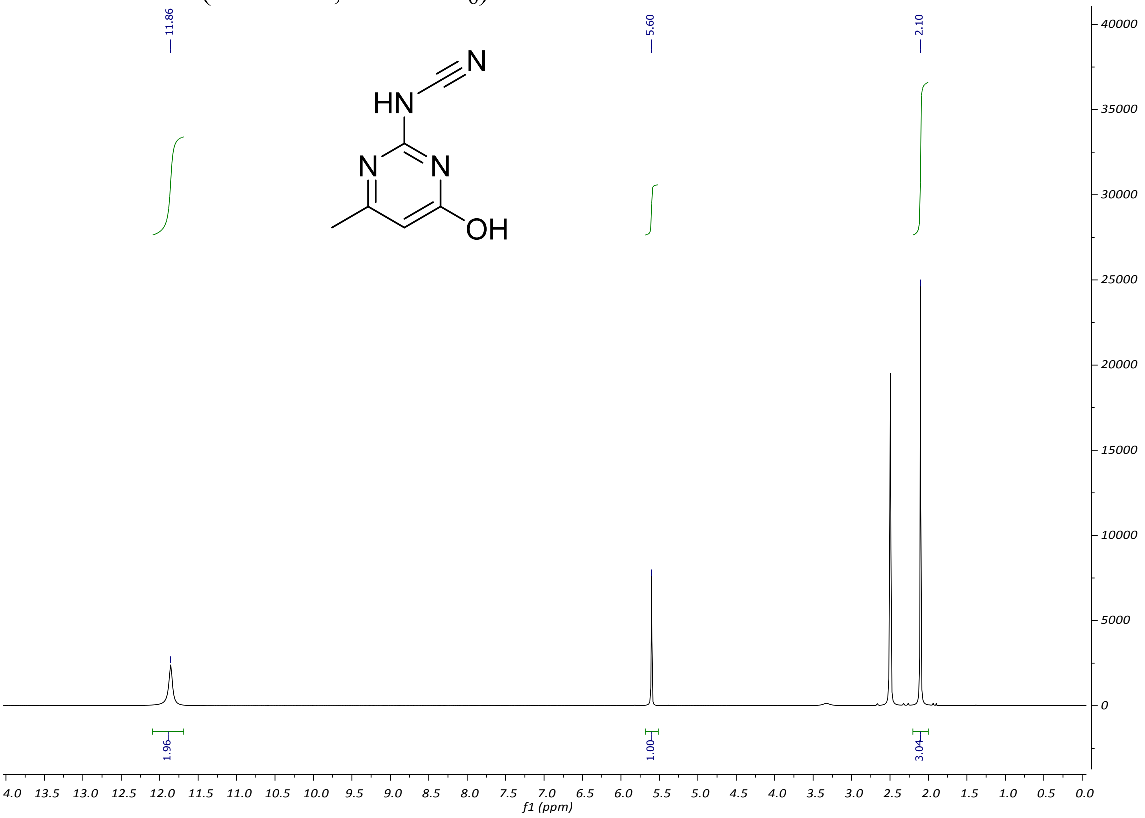

^{13}C{^{1}H} NMR of **3a** (101 MHz, DMSO-d_6)

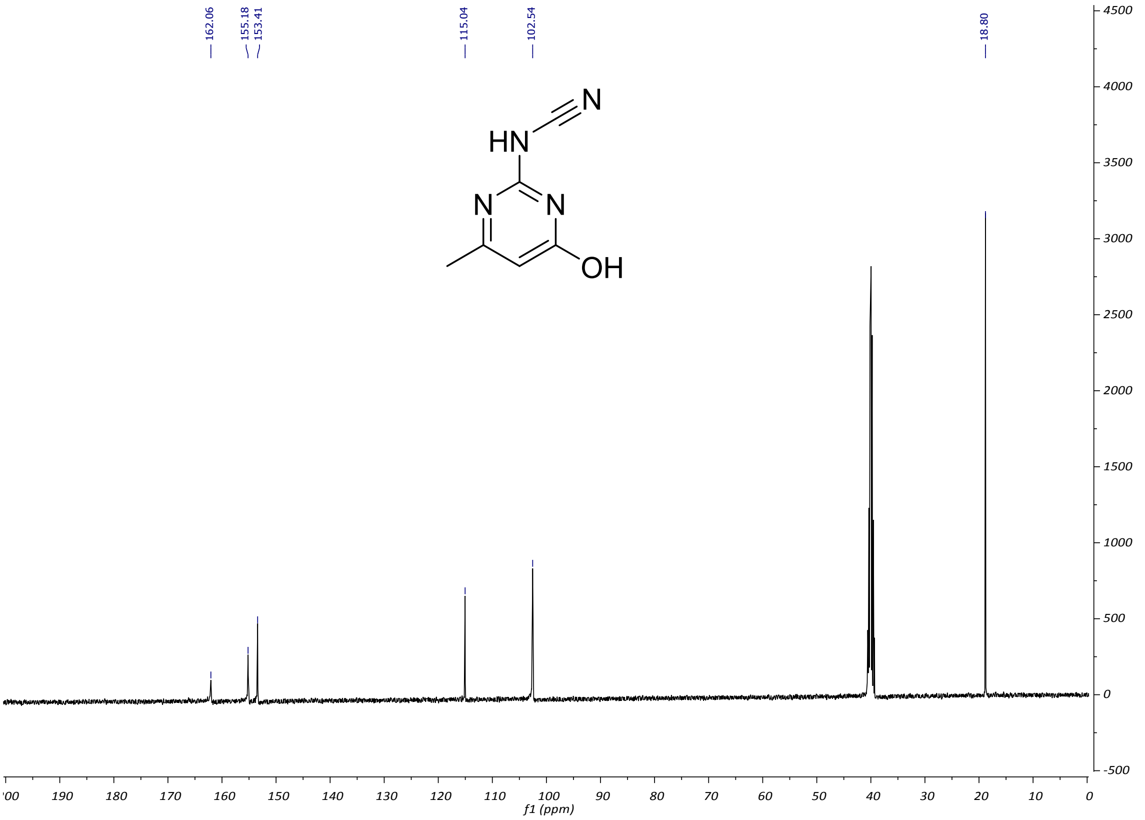

Figure S31. ^{1}H and ^{13}C{^{1}H} NMR spectra of *N*-(4-hydroxy-6-methylpyrimidin-2-yl)cyanamide (**3a**).

^{1}H NMR of **5aa** (400 MHz, DMSO-d_6)

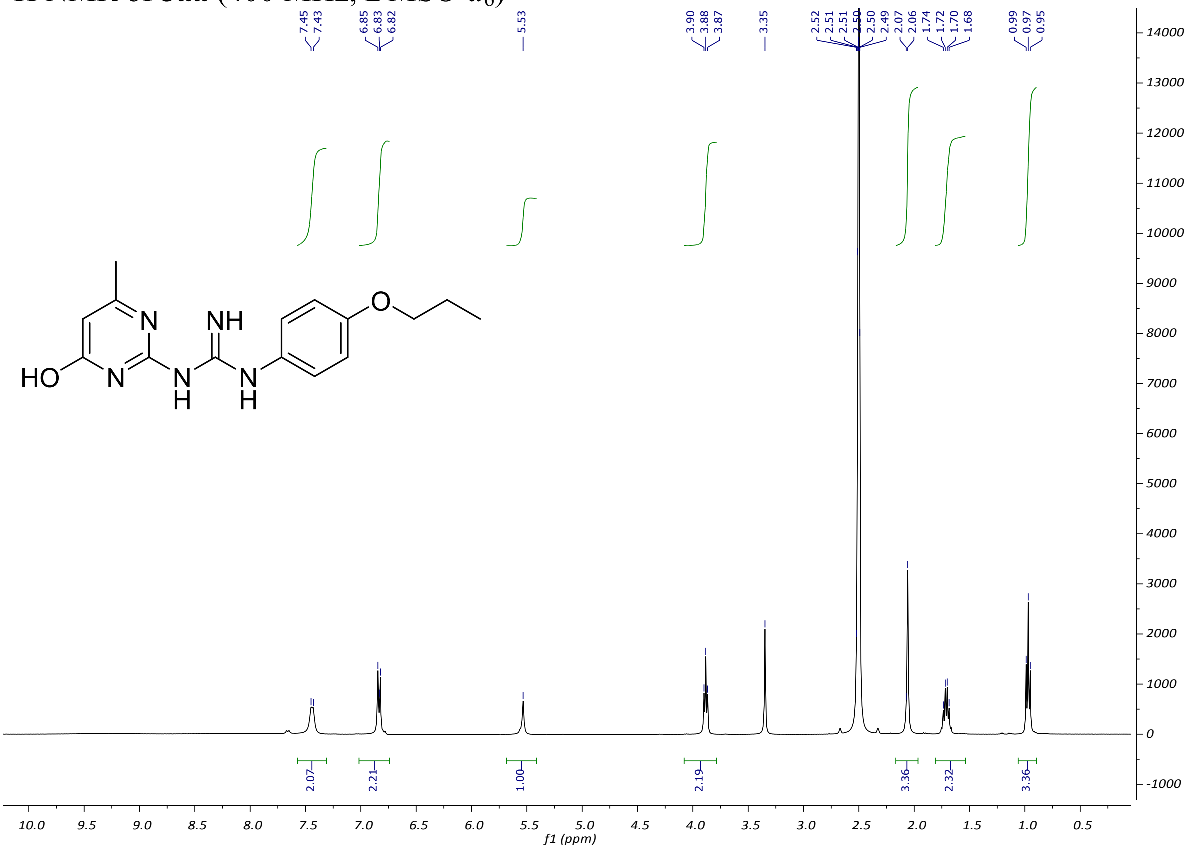

^{13}C{^{1}H} NMR of **5aa** (101 MHz, DMSO-d_6)

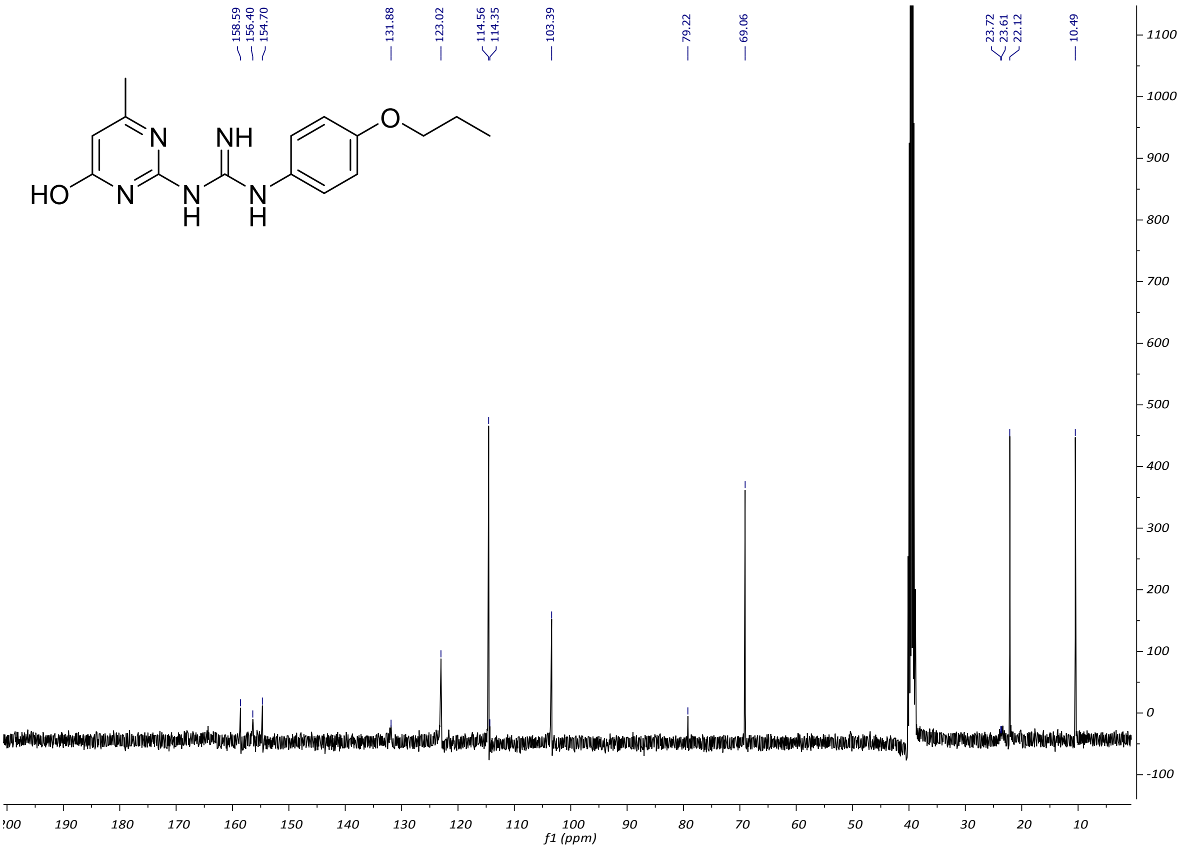

Figure S32. ^{1}H and ^{13}C{^{1}H} NMR spectra of 1-(4-hydroxy-6-methylpyrimidin-2-yl)-3-(4-propoxyphenyl)guanidine (**5aa**).

^{1}H NMR of **5ba** (400 MHz, DMSO-d_6)

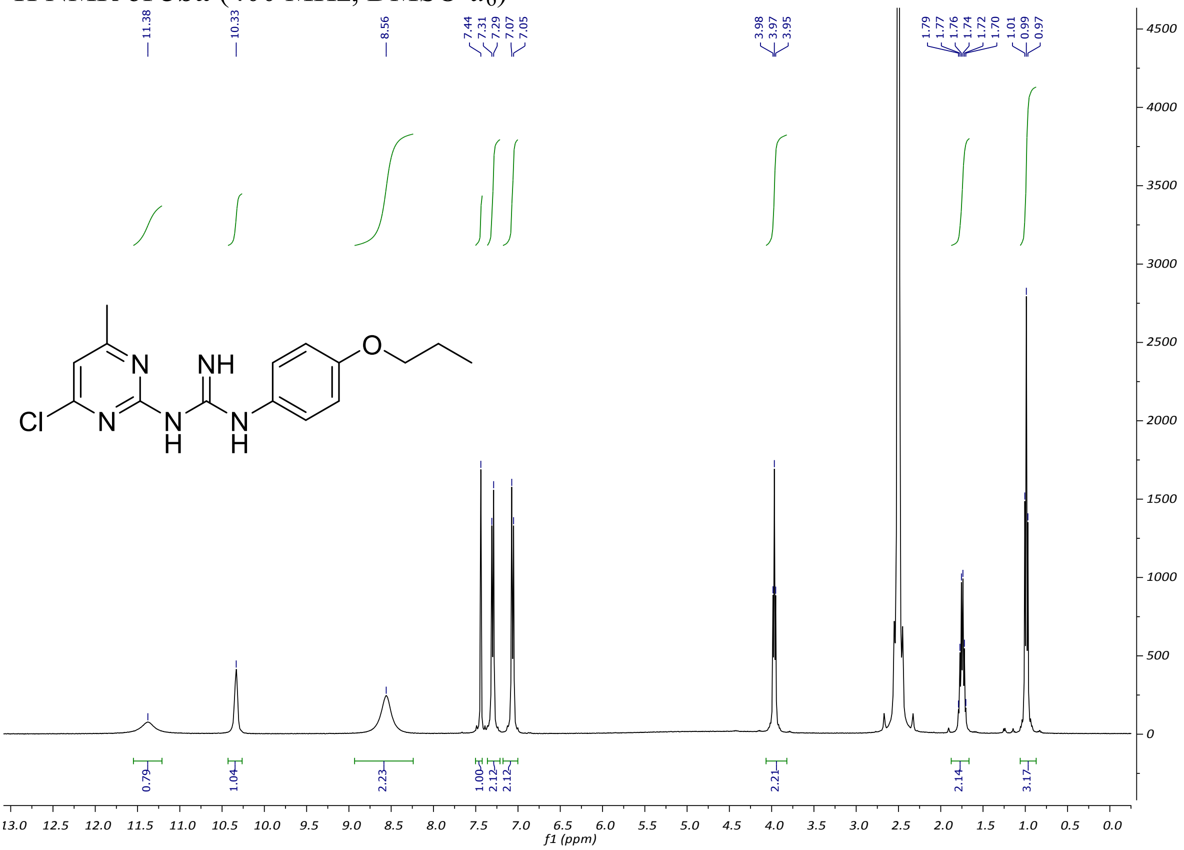

^{13}C{^{1}H} NMR of **5ba** (101 MHz, DMSO-d_6)

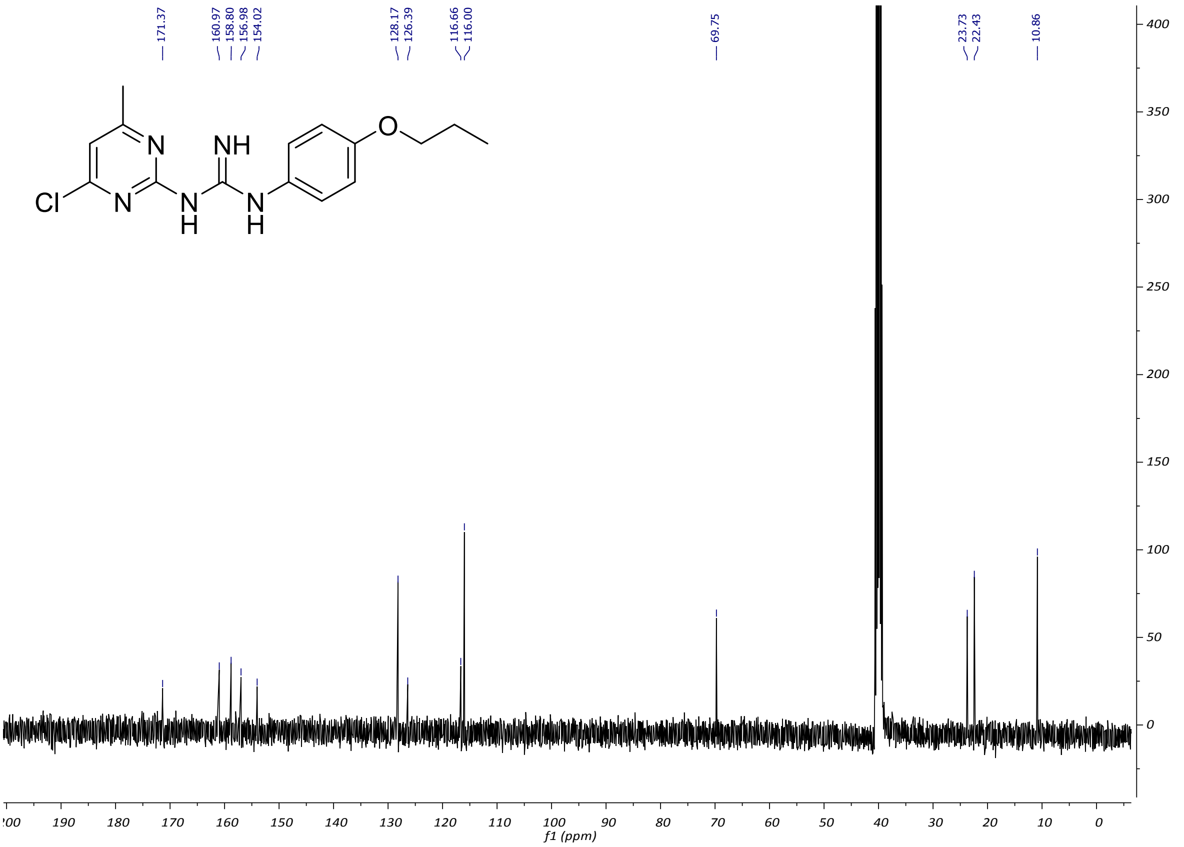

Figure S33. ^{1}H and ^{13}C{^{1}H} NMR spectra of 1-(4-chloro-6-methylpyrimidin-2-yl)-3-(4-propoxyphenyl)guanidine (**5ba**).

^{1}H NMR of **5da** (400 MHz, $CDCl_3$)

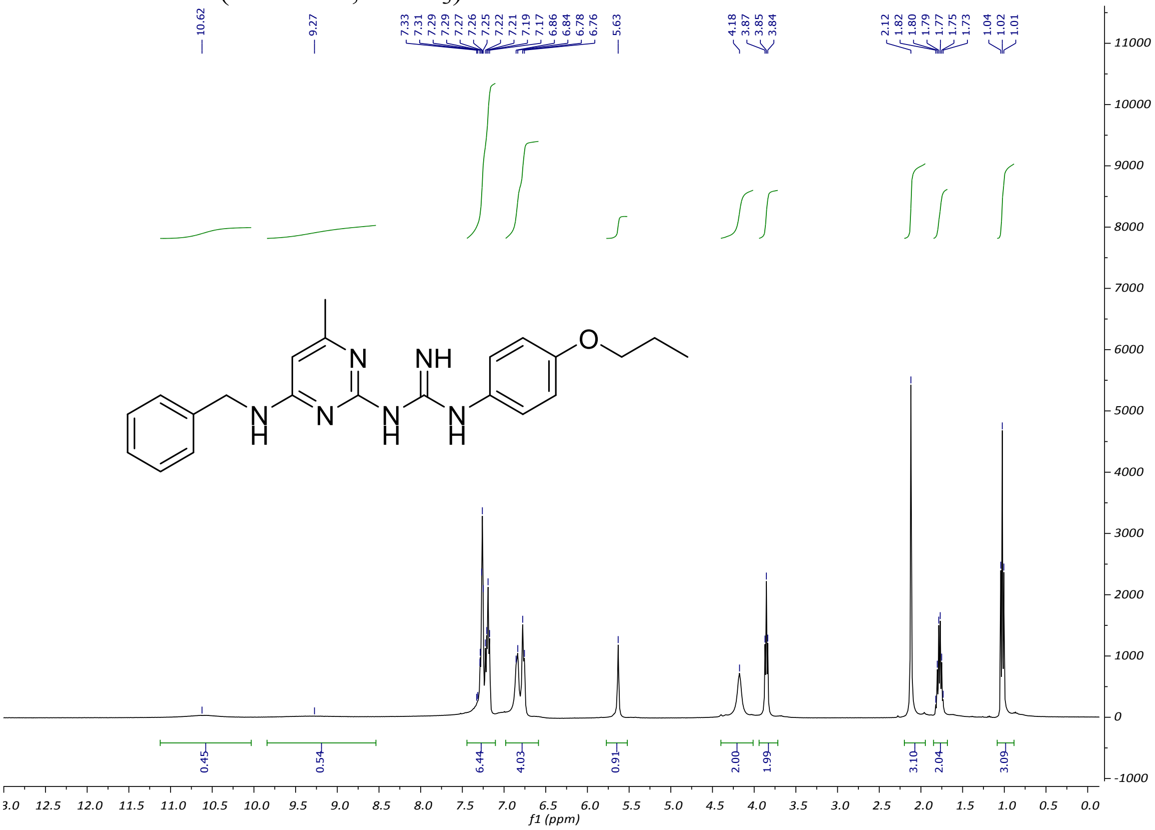

^{13}C{^{1}H} NMR of **5da** (101 MHz, $CDCl_3$)

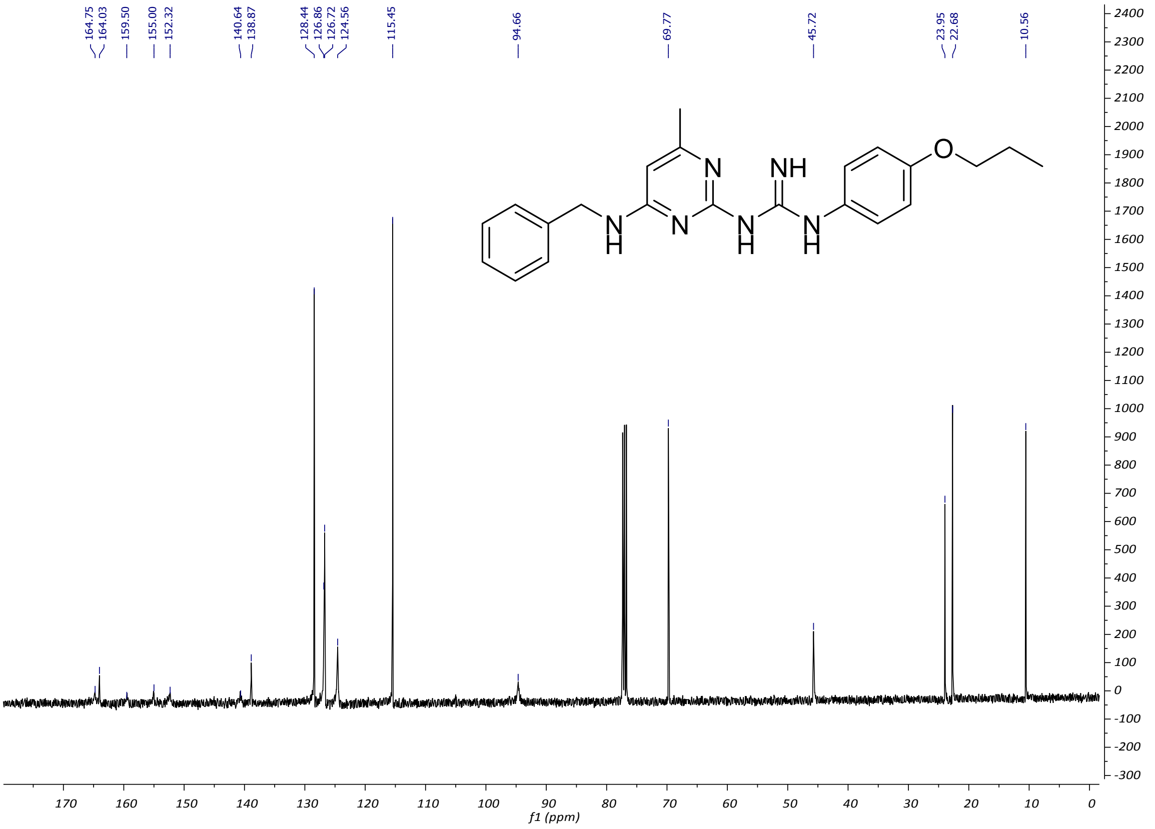

Figure S34. ^{1}H and ^{13}C{^{1}H} NMR spectra of 1-(4-(benzylamino)-6-methylpyrimidin-2-yl)-3-(4-propoxyphenyl)guanidine (**5da**).

^1H NMR of **5db** (400 MHz, DMSO-d_6)

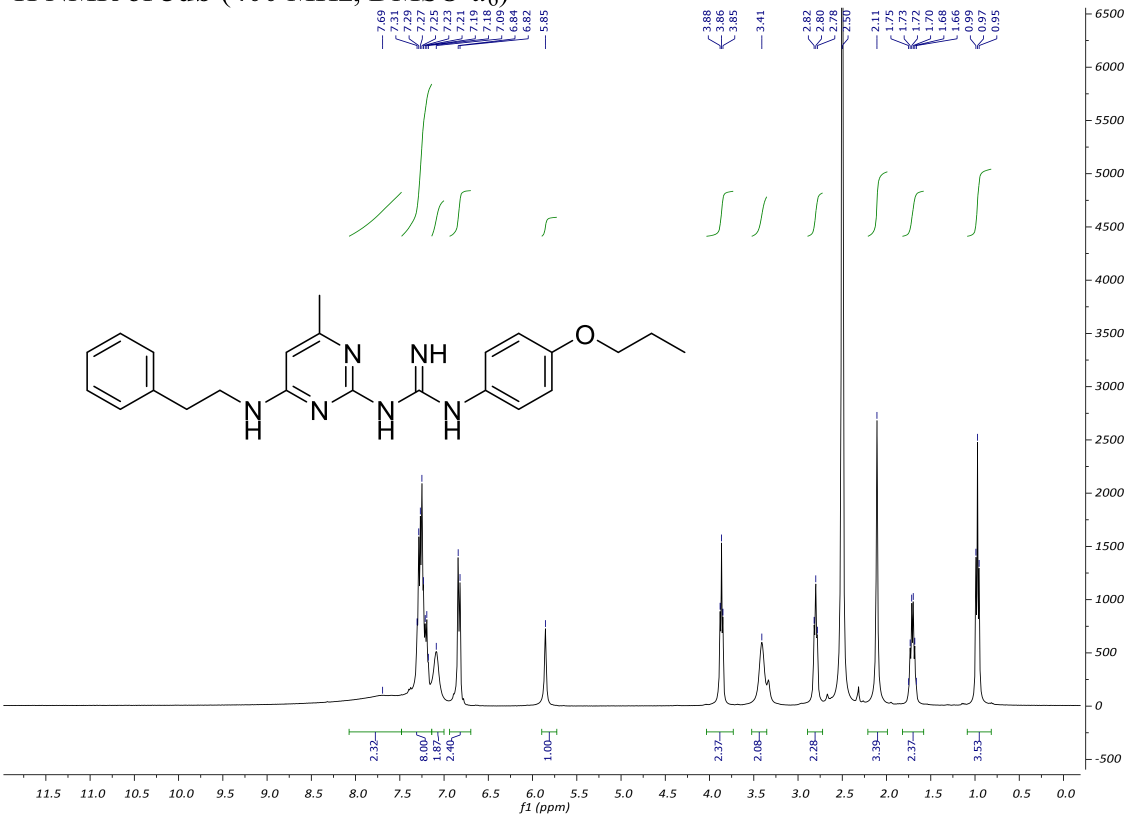

$^{13}C\{^1H\}$ NMR of **5db** (101 MHz, DMSO-d_6)

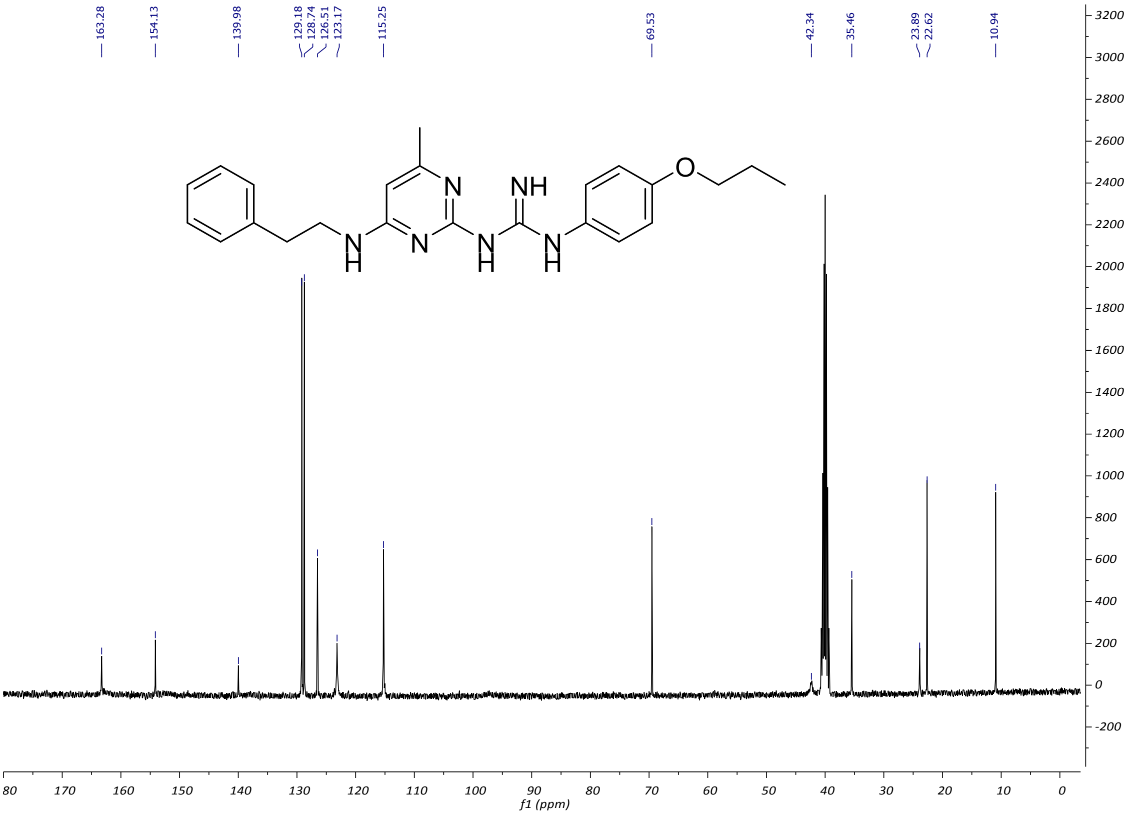

Figure S35. ^1H and $^{13}C\{^1H\}$ NMR spectra of 1-(4-methyl-6-(phenethylamino)pyrimidin-2-yl)-3-(4-propoxyphenyl)guanidine (**5db**).

^{1}H NMR of **5dc** (400 MHz, DMSO-d_6)

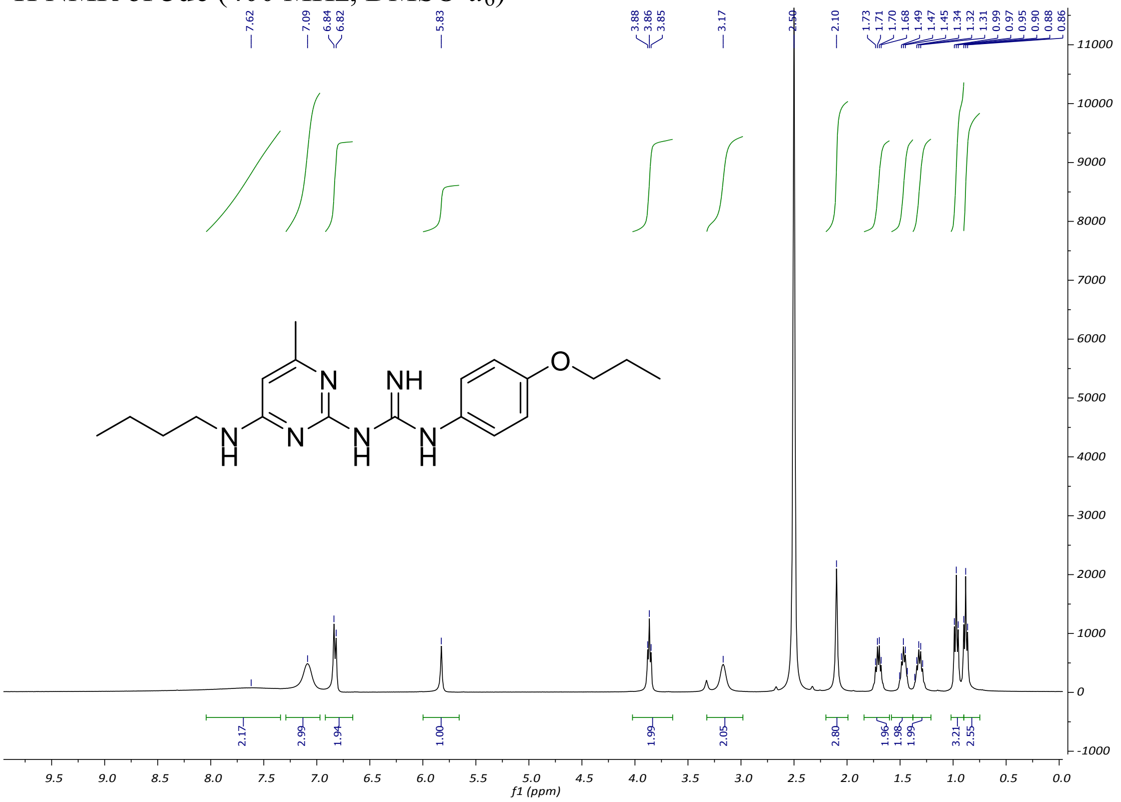

$^{13}C\{^{1}H\}$ NMR of **5dc** (101 MHz, DMSO-d_6)

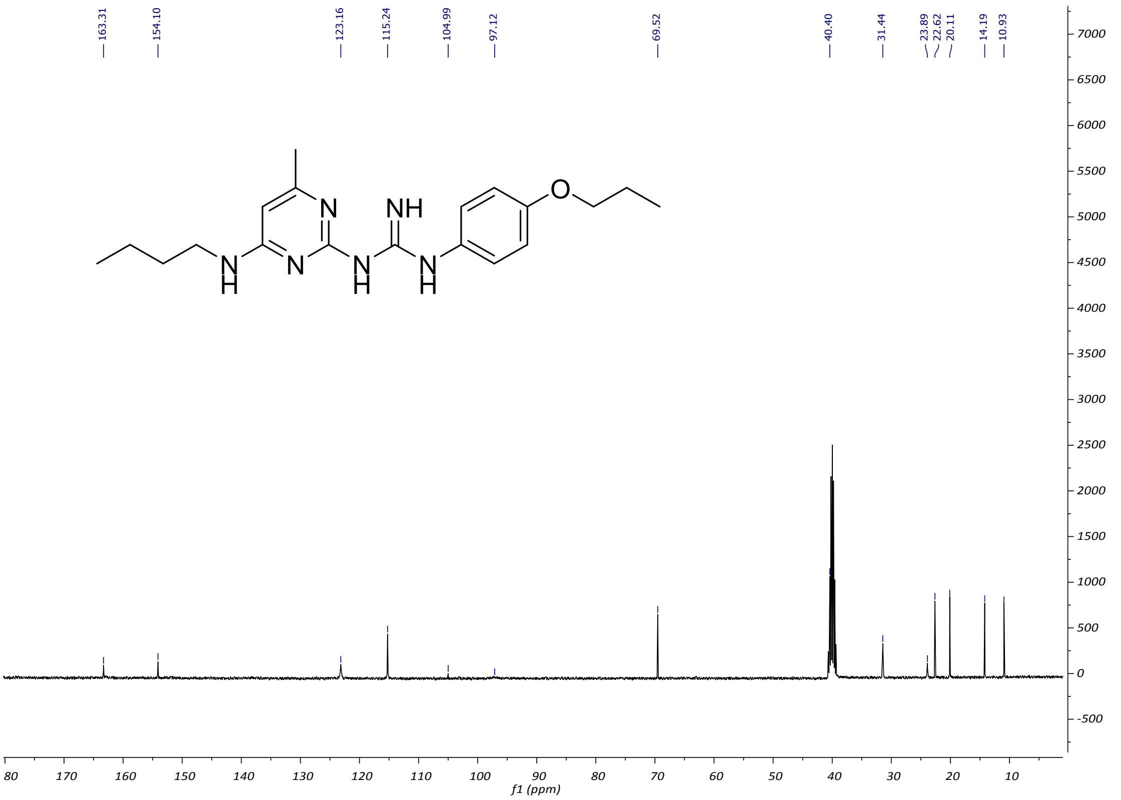

Figure S36. ^{1}H and $^{13}C\{^{1}H\}$ NMR spectra of 1-(4-(butylamino)-6-methylpyrimidin-2-yl)-3-(4-propoxyphenyl)guanidine (**5dc**).

^{1}H NMR of **3c** (400 MHz, DMSO-d_6)

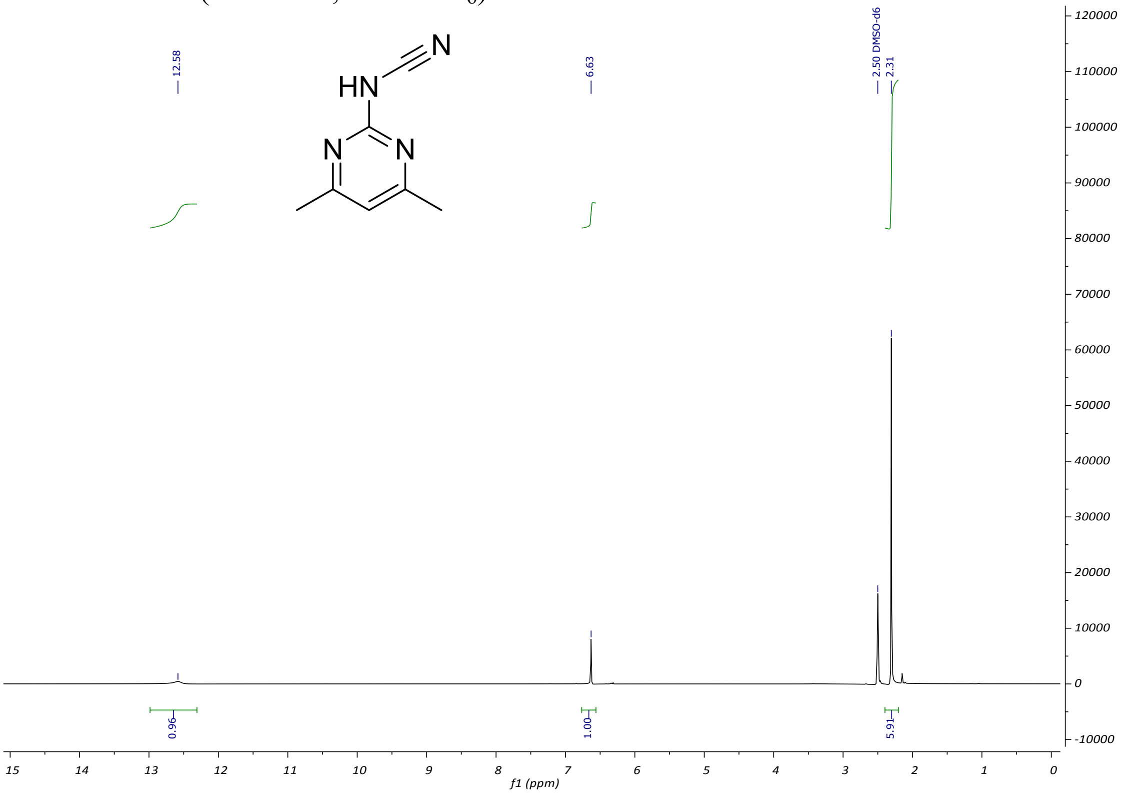

^{13}C{^{1}H} NMR of **3c** (101 MHz, DMSO-d_6)

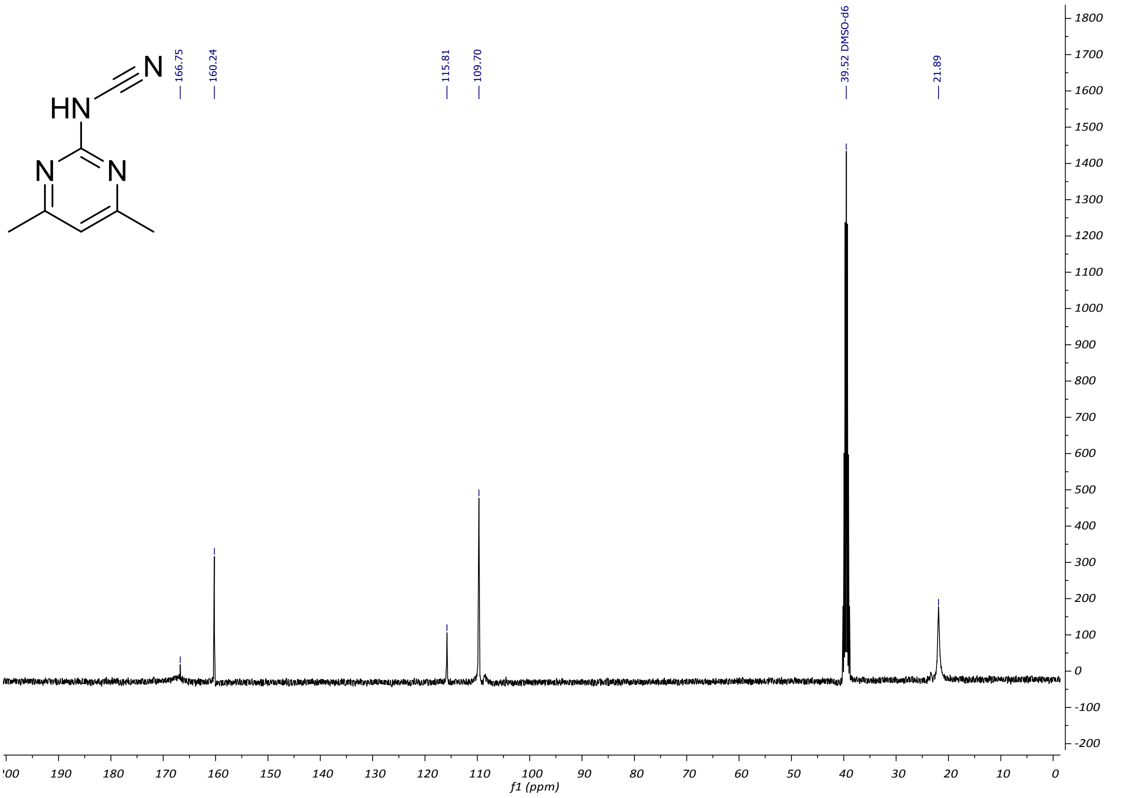

Figure S37. ^{1}H and ^{13}C{^{1}H} NMR spectra of *N*-(4,6-dimethylpyrimidin-2-yl)cyanamide (**3c**).

^{1}H NMR of **5ca** (400 MHz, MeOD-d_4)

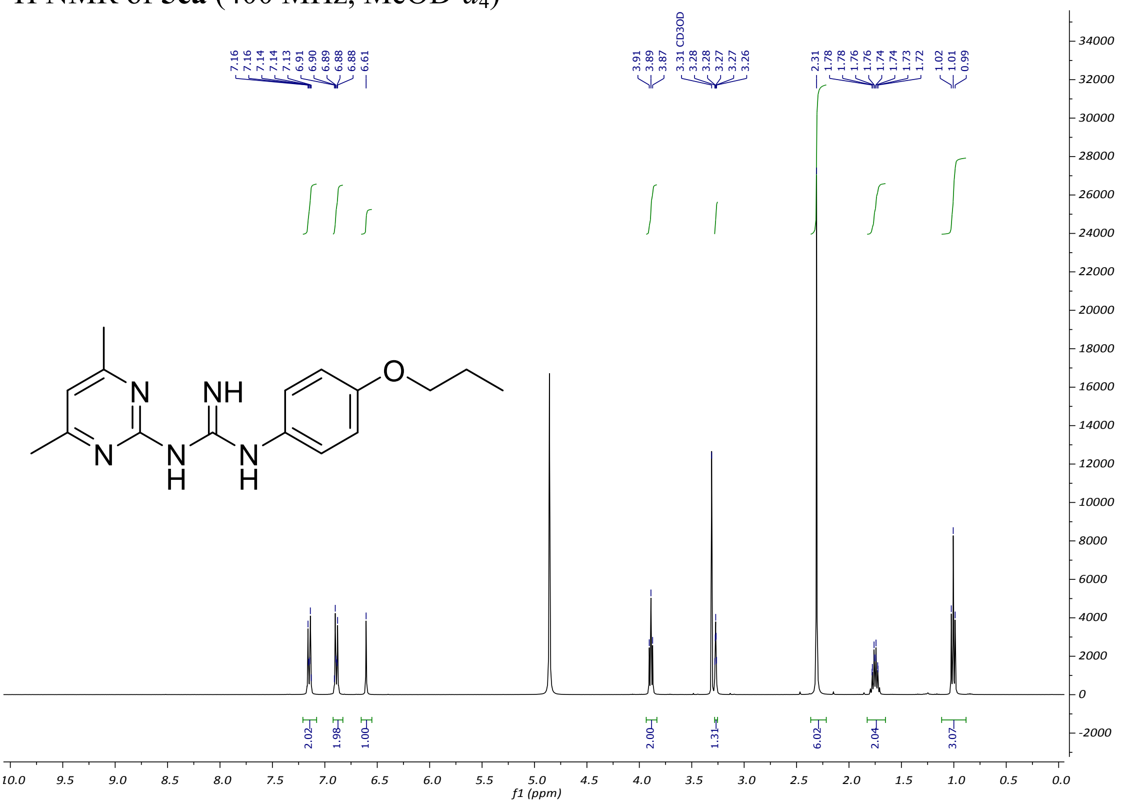

^{13}C{^{1}H} NMR of **5ca** (101 MHz, MeOD-d_4)

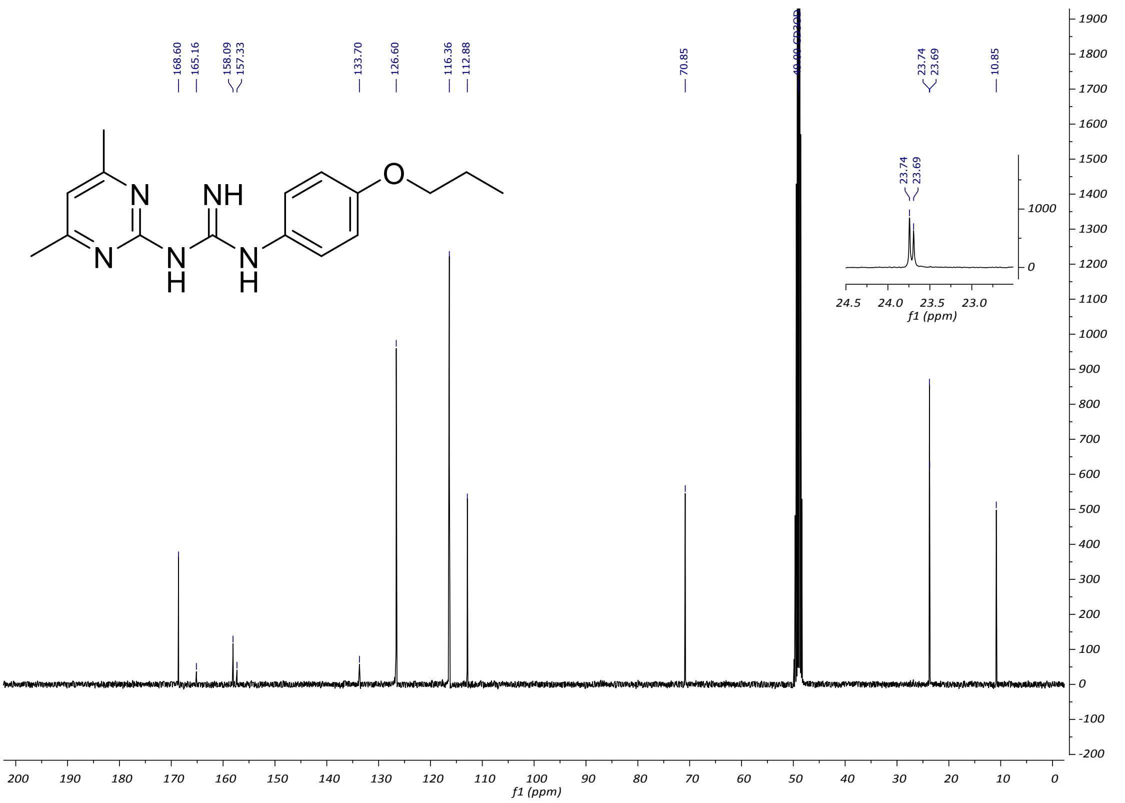

Figure S38. ^{1}H and ^{13}C{^{1}H} NMR spectra 1-(4,6-dimethylpyrimidin-2-yl)-3-(4-propoxyphenyl)guanidine (**5ca**).

^{1}H NMR of **5cb** (400 MHz, DMSO-d_6)

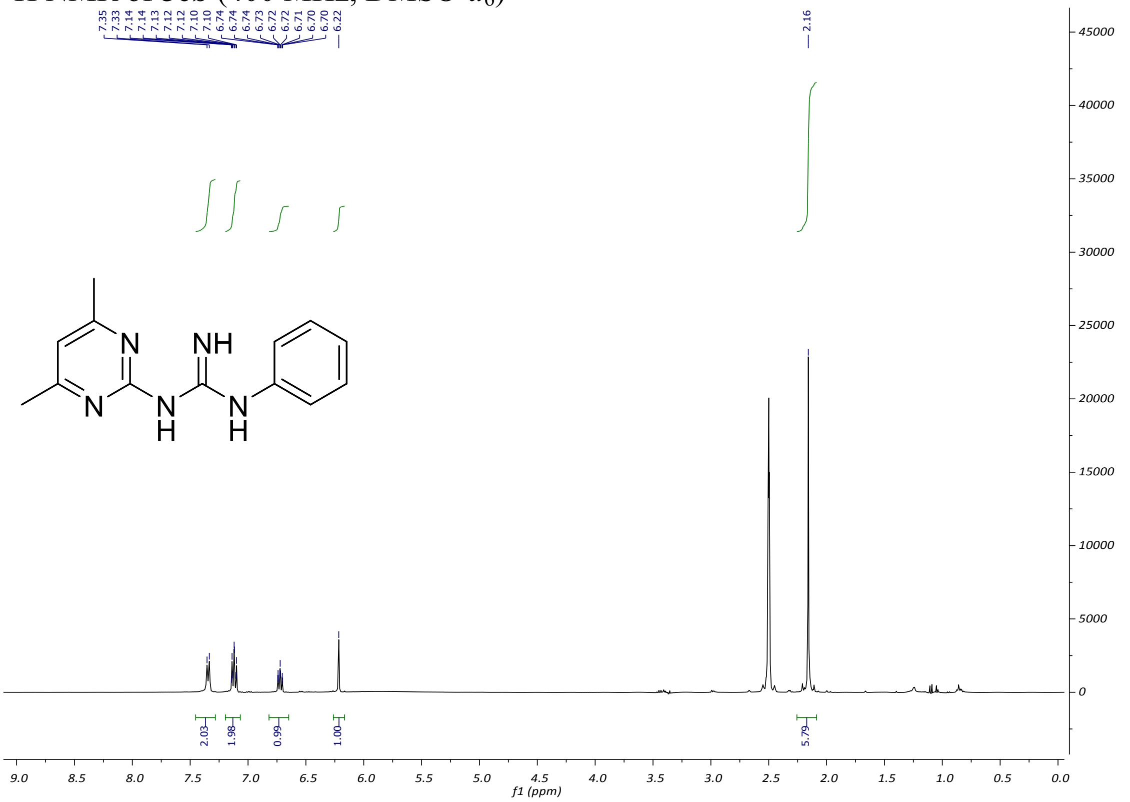

^{13}C{^{1}H} NMR of **5cb** (101 MHz, DMSO-d_6)

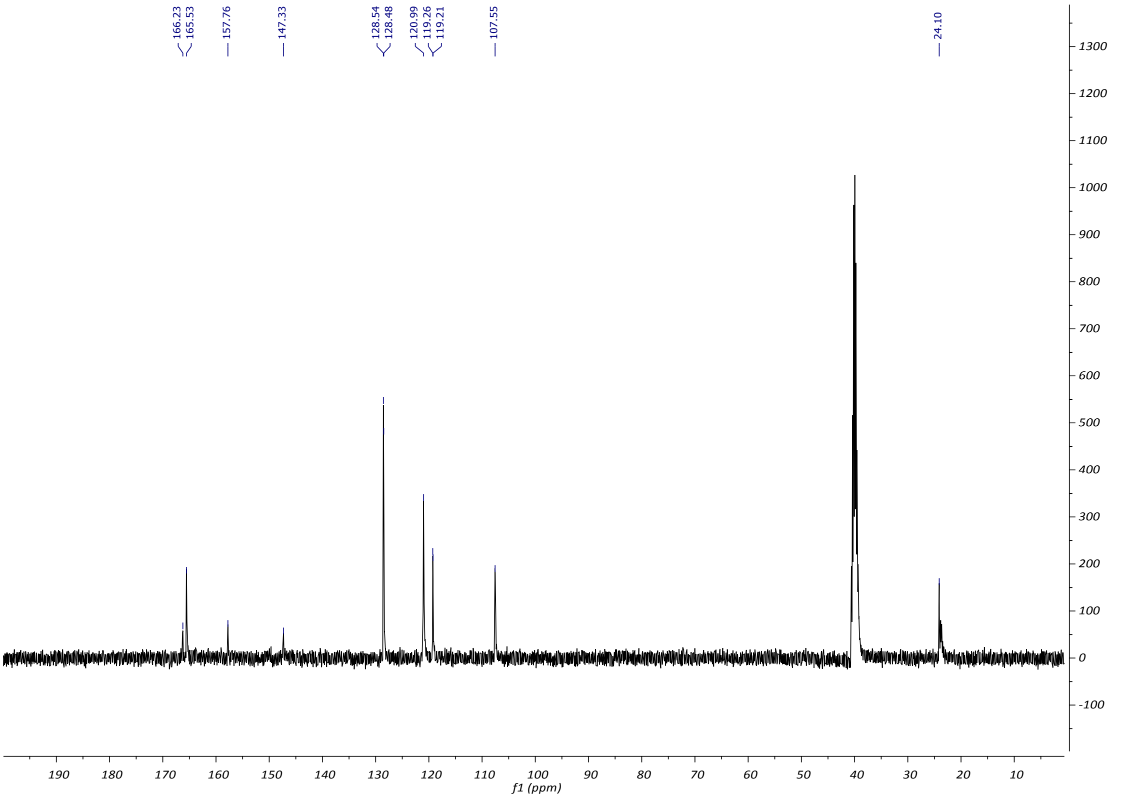

Figure S39. ^{1}H and ^{13}C{^{1}H} NMR spectra of 1-(4,6-dimethylpyrimidin-2-yl)-3-phenylguanidine (**5cb**).

^{1}H NMR of **5cc** (400 MHz, DMSO-d_6)

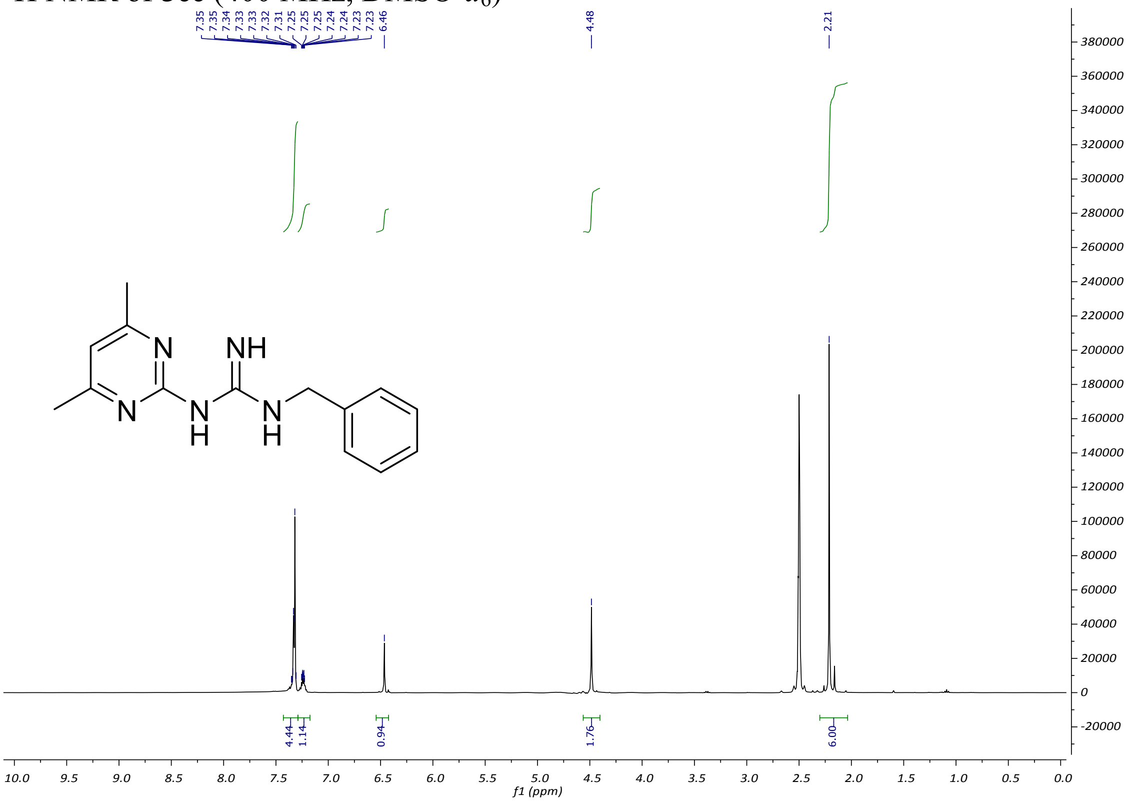

^{13}C{^{1}H} NMR of **5cc** (101 MHz, DMSO-d_6)

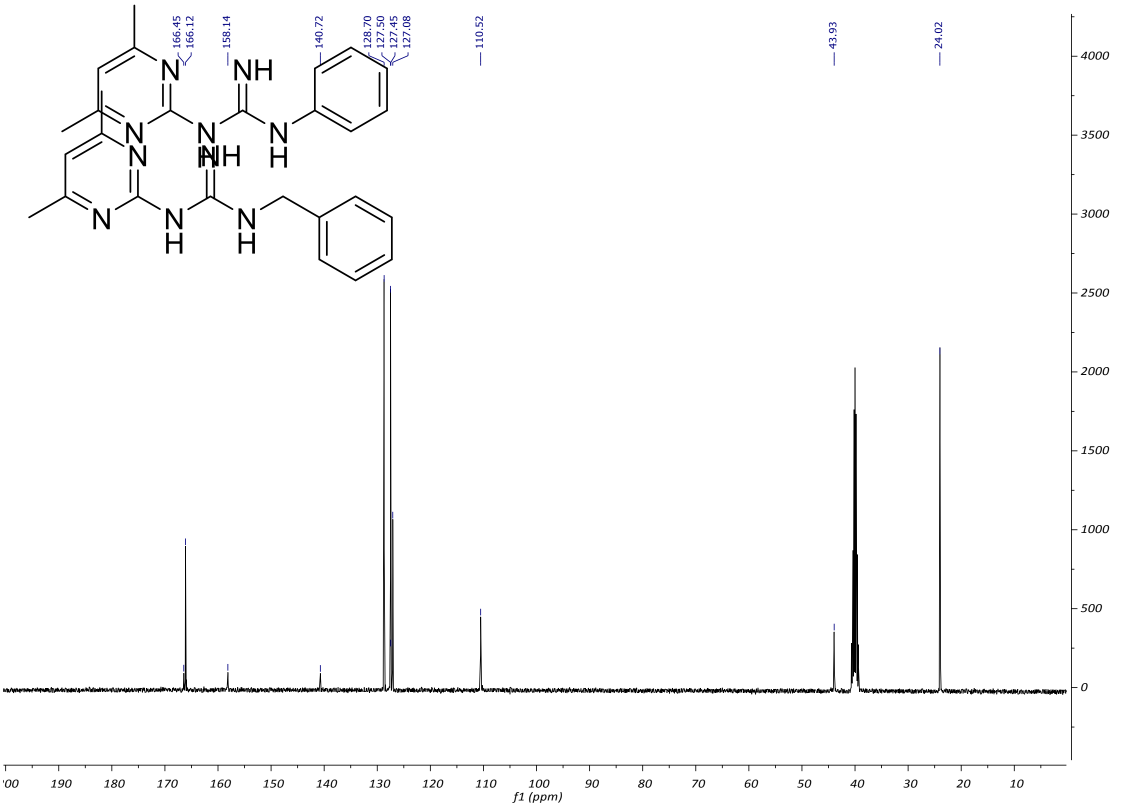

Figure S40. ^{1}H and ^{13}C{^{1}H} NMR spectra of 1-benzyl-3-(4,6-dimethylpyrimidin-2-yl)guanidine (**5cc**).

^{1}H NMR of **5cd** (400 MHz, DMSO-d_6)

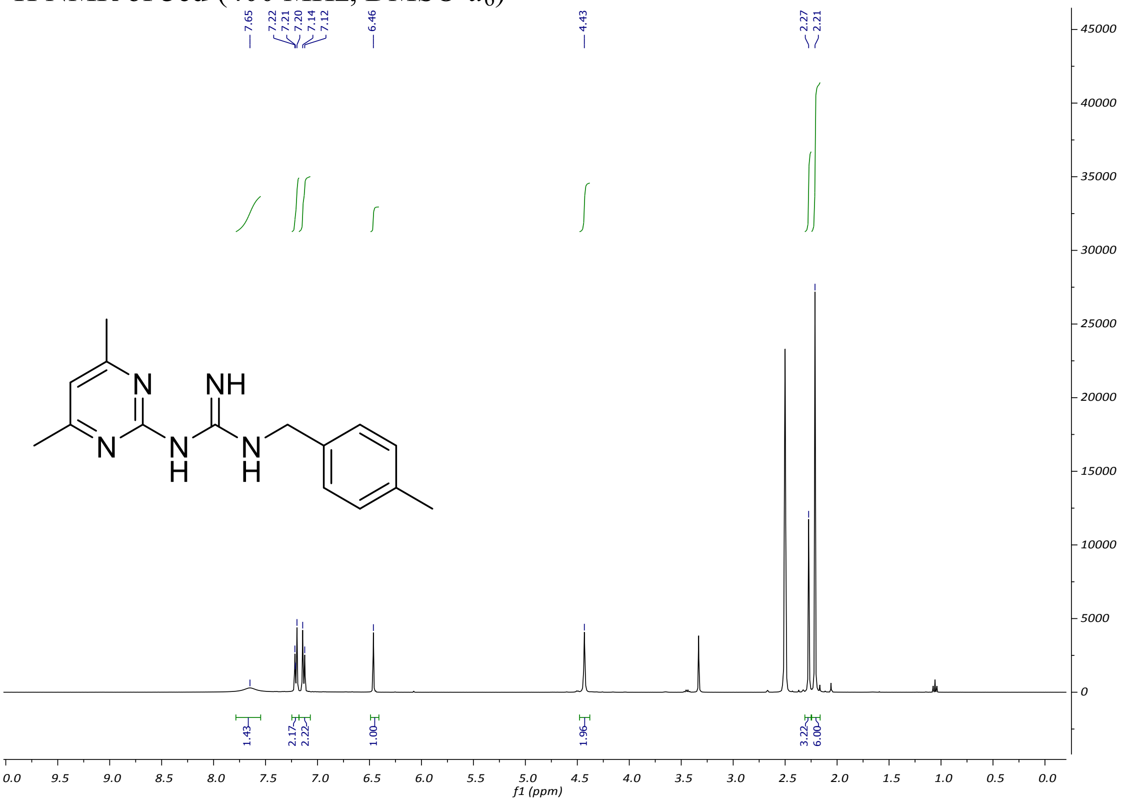

^{13}C{^{1}H} NMR of **5cd** (101 MHz, DMSO-d_6)

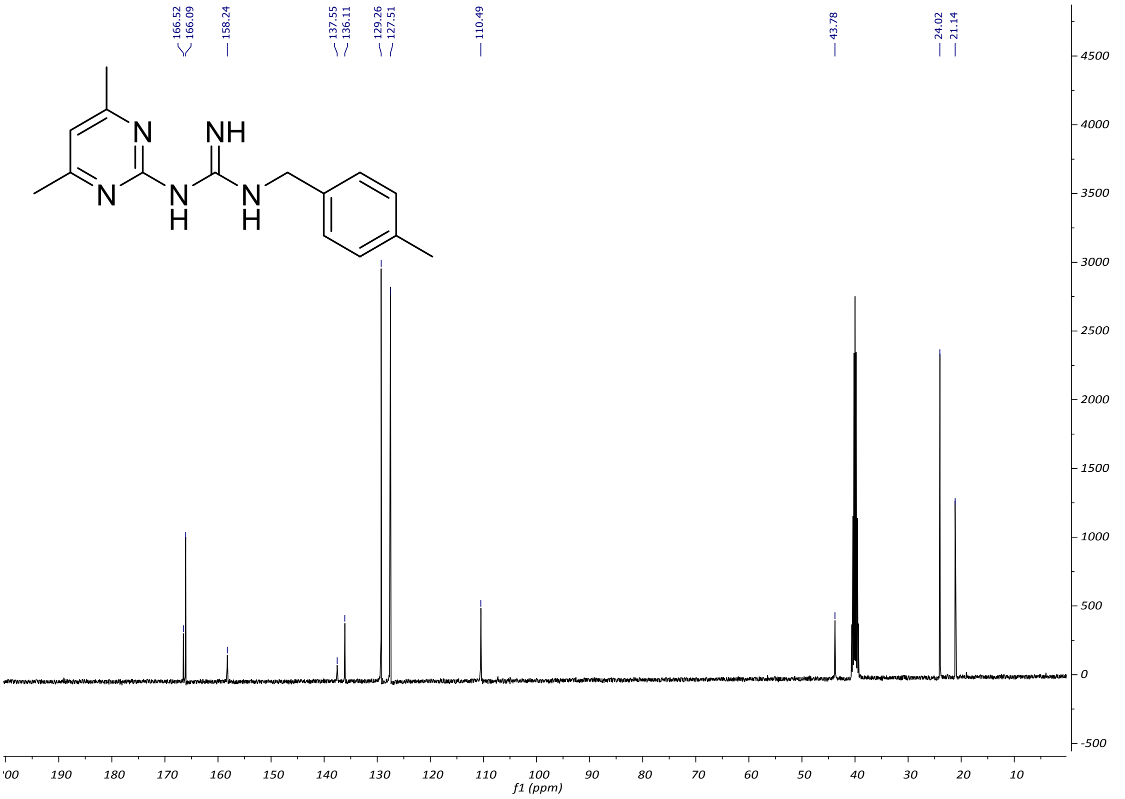

Figure S41. ^{1}H and ^{13}C{^{1}H} NMR spectra of 1-(4,6-dimethylpyrimidin-2-yl)-3-(4-methylbenzyl)guanidine (**5cd**).

^{1}H NMR of **5ce** (400 MHz, DMSO-d_6)

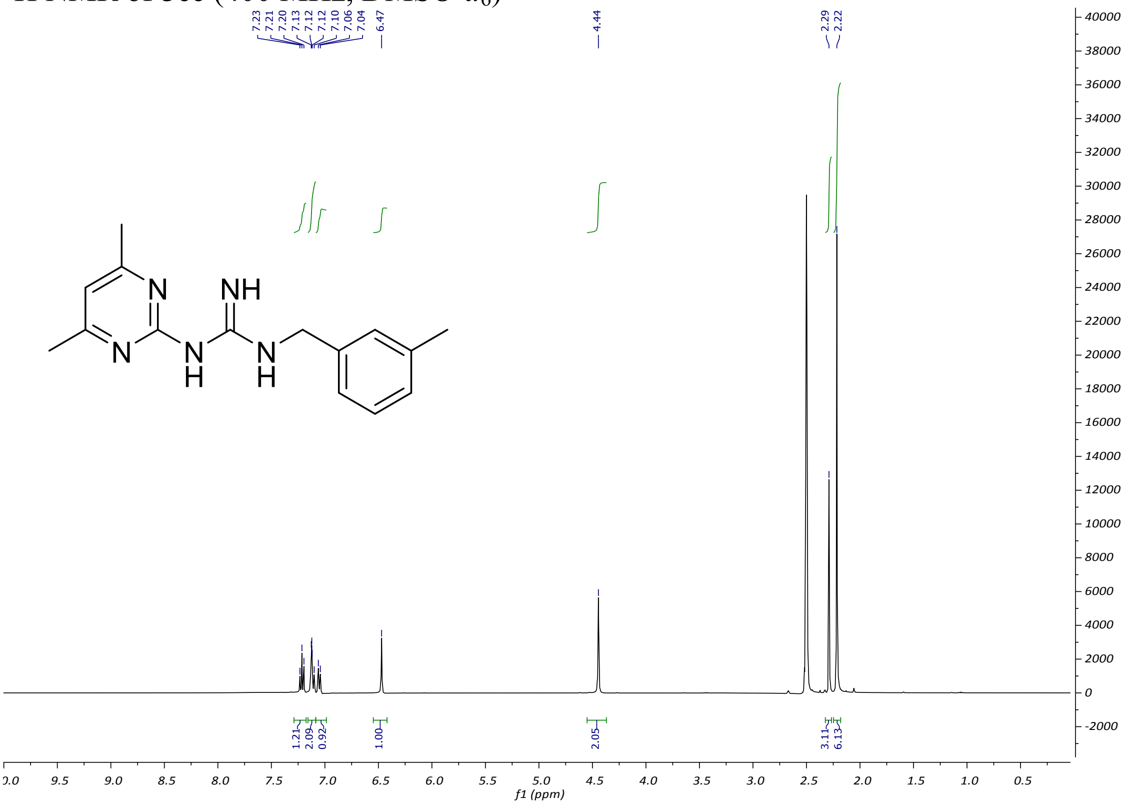

$^{13}C\{^{1}H\}$ NMR of **5ce** (101 MHz, DMSO-d_6)

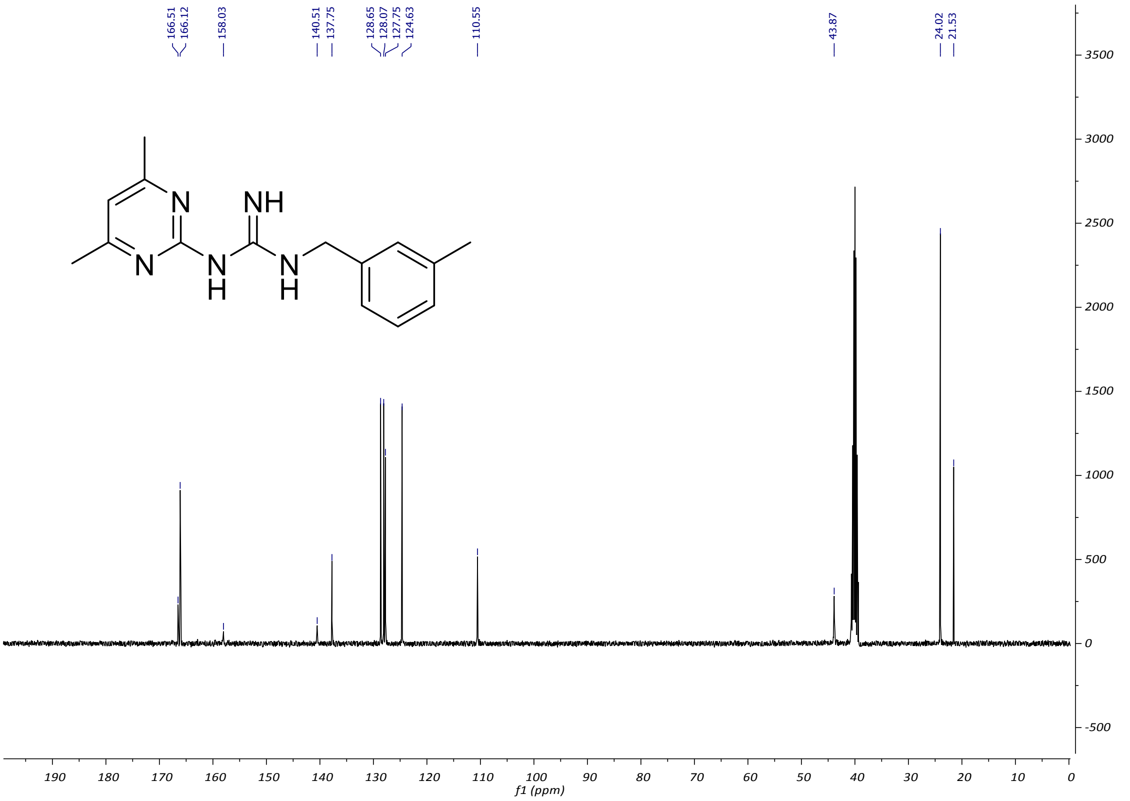

Figure S42. ^{1}H and $^{13}C\{^{1}H\}$ NMR spectra of 1-(4,6-dimethylpyrimidin-2-yl)-3-(3-methylbenzyl)guanidine (**5ce**).

^{1}H NMR of **5cf** (400 MHz, DMSO-d_6)

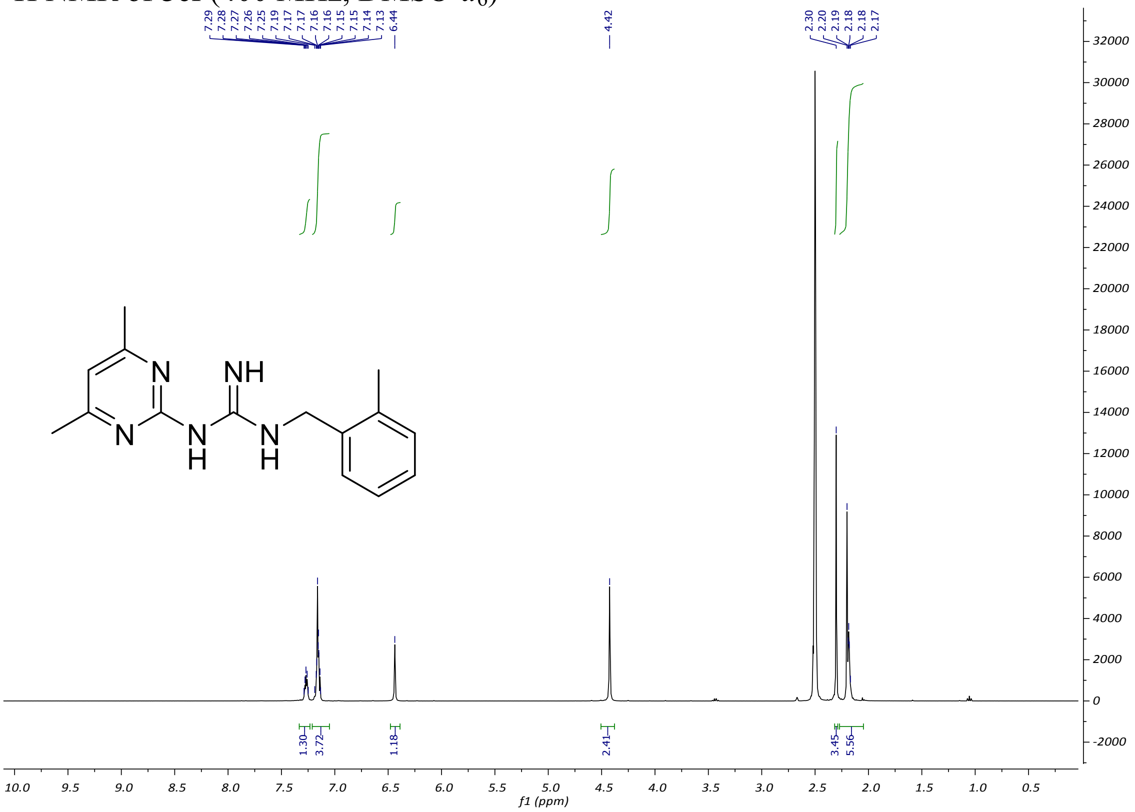

$^{13}C\{^{1}H\}$ NMR of **5cf** (101 MHz, DMSO-d_6)

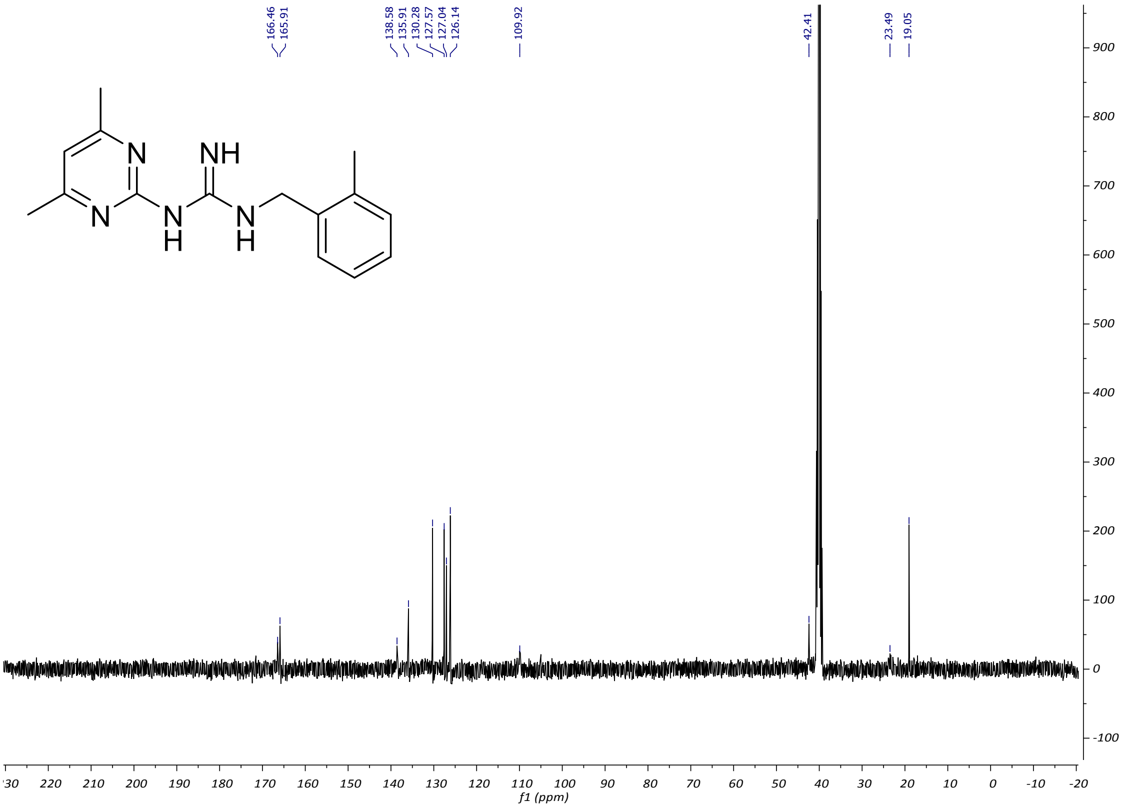

Figure S43. ^{1}H and $^{13}C\{^{1}H\}$ NMR spectra of 1-(4,6-dimethylpyrimidin-2-yl)-3-(2-methylbenzyl)guanidine (**5cf**).

^{1}H NMR of **5cg** (400 MHz, $CDCl_3$)

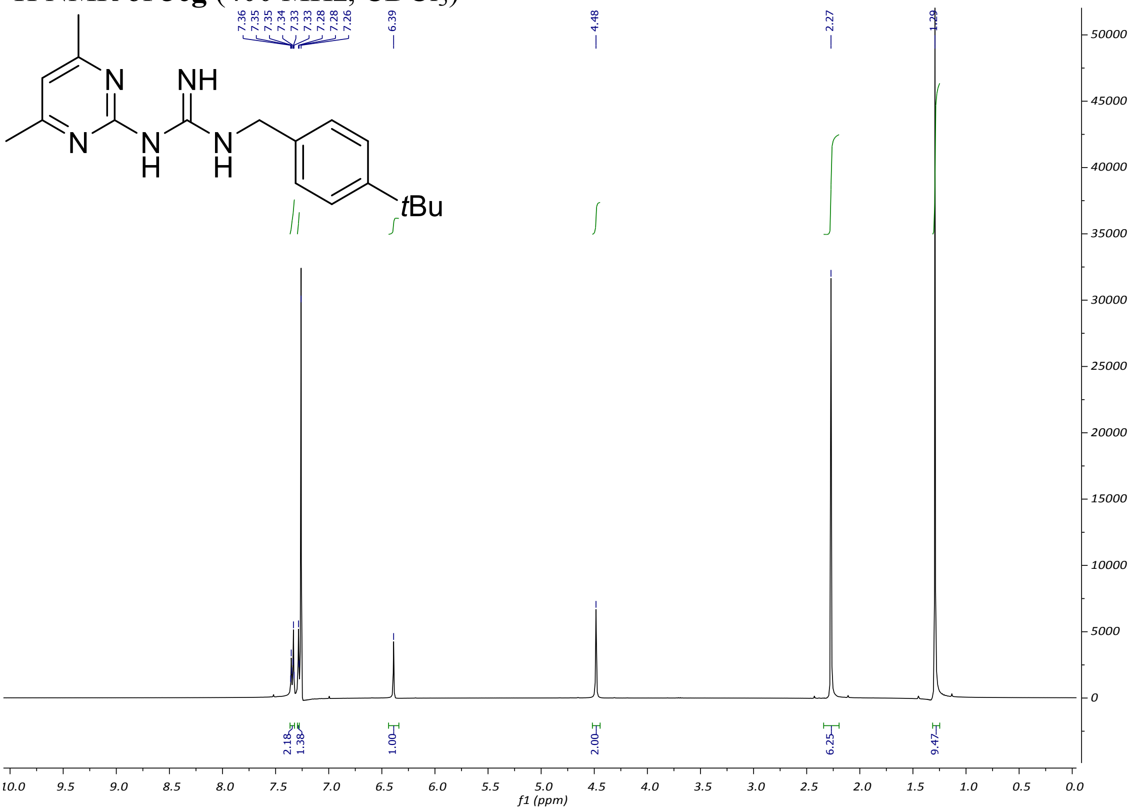

^{13}C{^{1}H} NMR of **5cg** (101 MHz, $CDCl_3$)

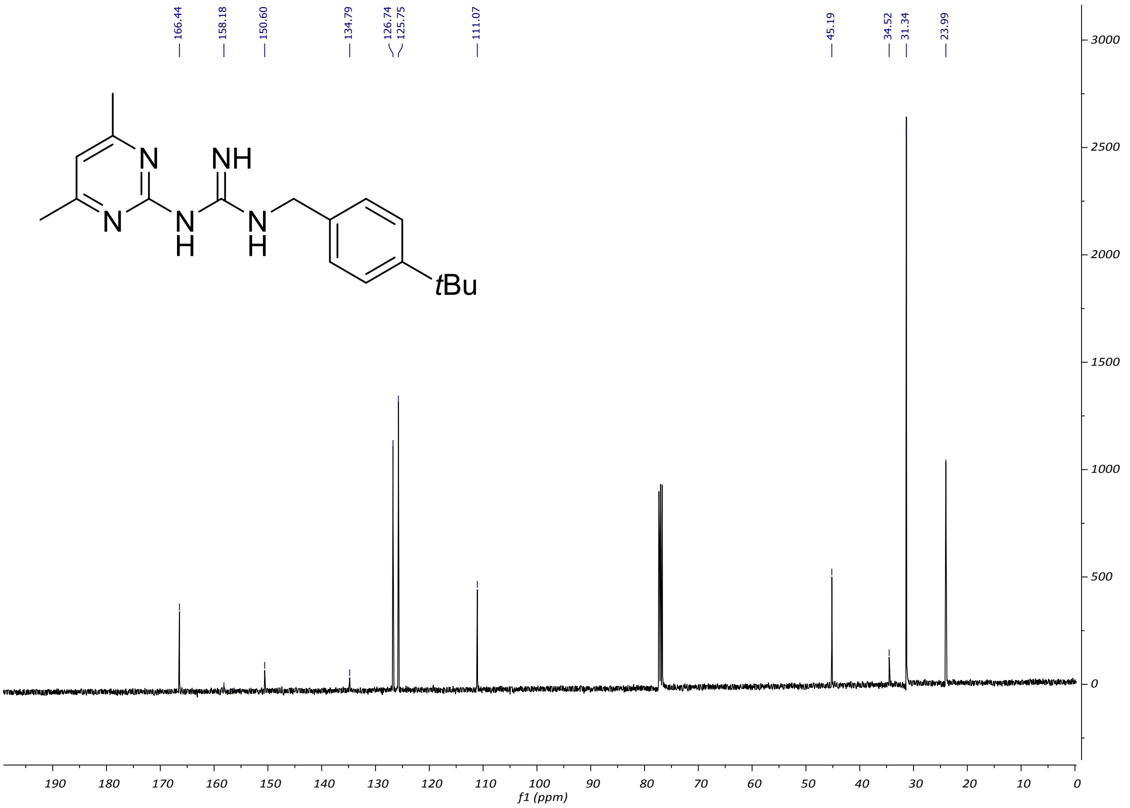

Figure S44. ^{1}H and ^{13}C{^{1}H} NMR spectra of 1-(4-(*tert*-butyl)benzyl)-3-(4,6-dimethylpyrimidin-2-yl)guanidine (**5cg**).

^{1}H NMR of **5ch** (400 MHz, DMSO-d_6)

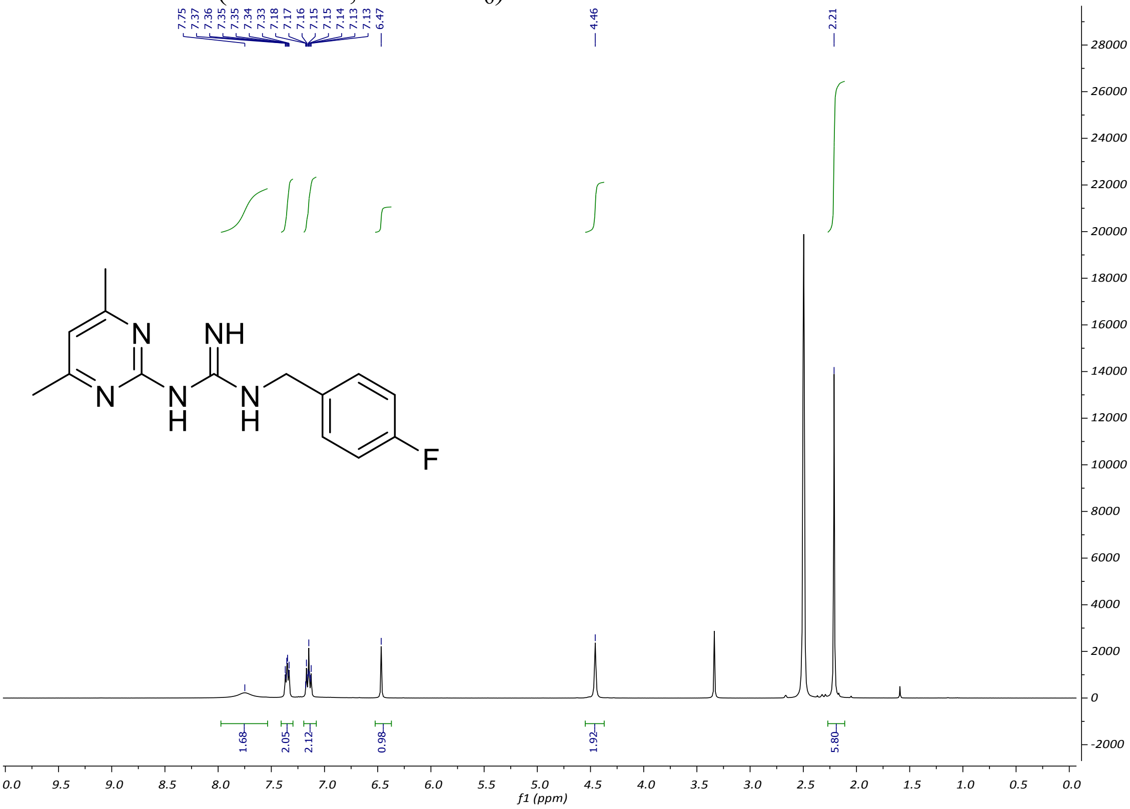

$^{13}C\{^{1}H\}$ NMR of **5ch** (101 MHz, DMSO-d_6)

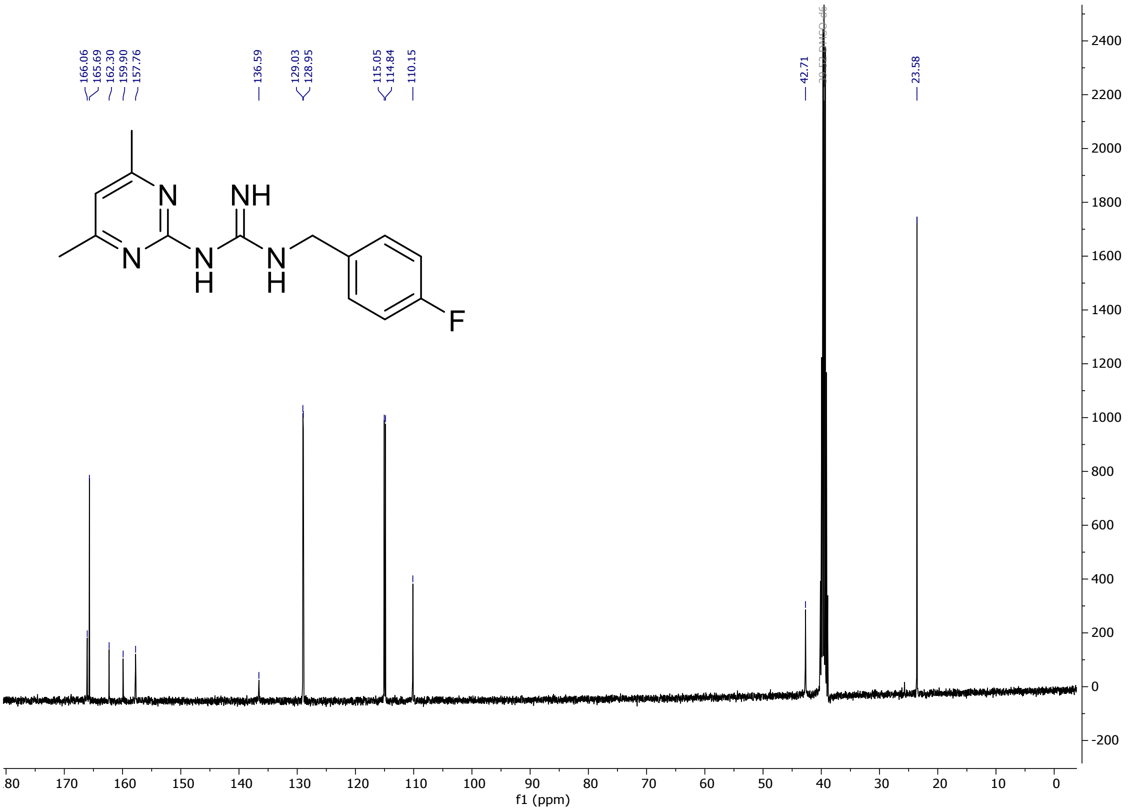

Figure S45. ^{1}H and $^{13}C\{^{1}H\}$ NMR spectra of 1-(4,6-dimethylpyrimidin-2-yl)-3-(4-fluorobenzyl)guanidine (**5ch**).

^{19}F NMR of **5ch** (376 MHz, DMSO-d_6)

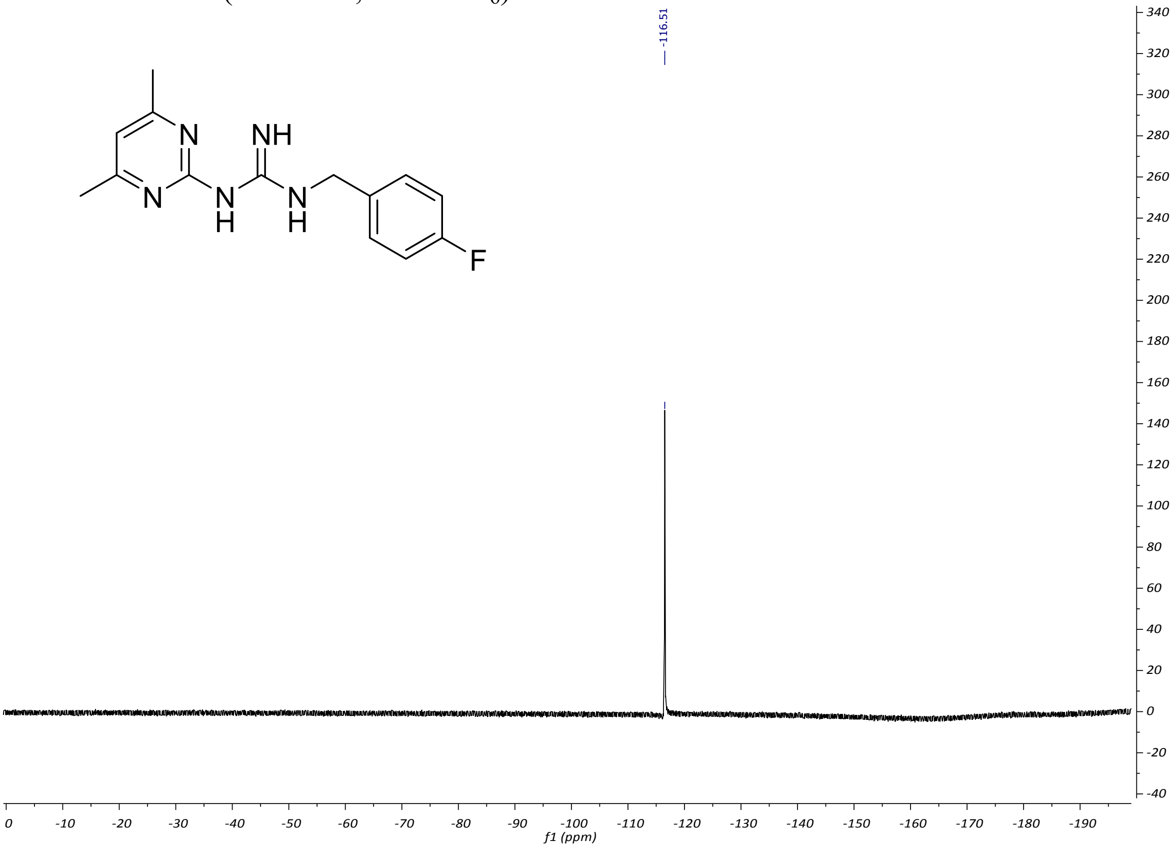

Figure S46. ^{19}F NMR spectra of 1-(4,6-dimethylpyrimidin-2-yl)-3-(4-fluorobenzyl)guanidine (**5ch**).

^{1}H NMR of **5ci** (400 MHz, DMSO-d_6)

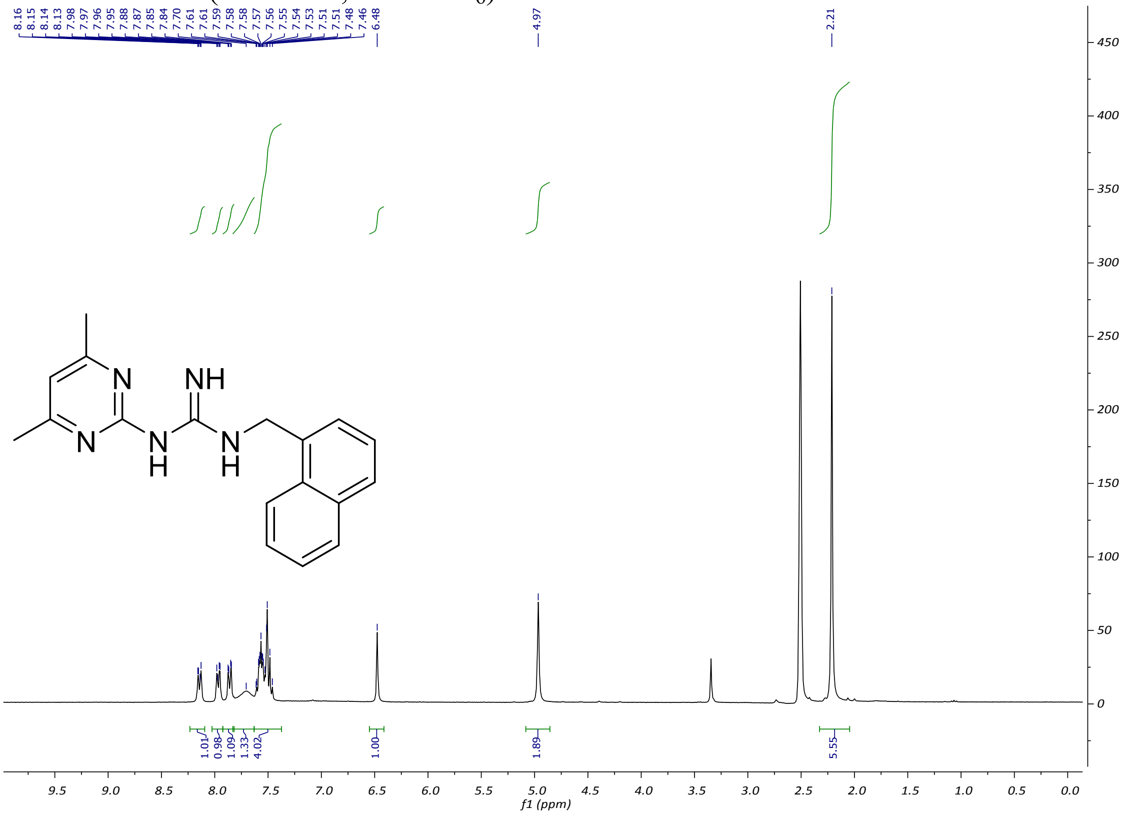

^{13}C{^{1}H} NMR of **5ci** (101 MHz, DMSO-d_6)

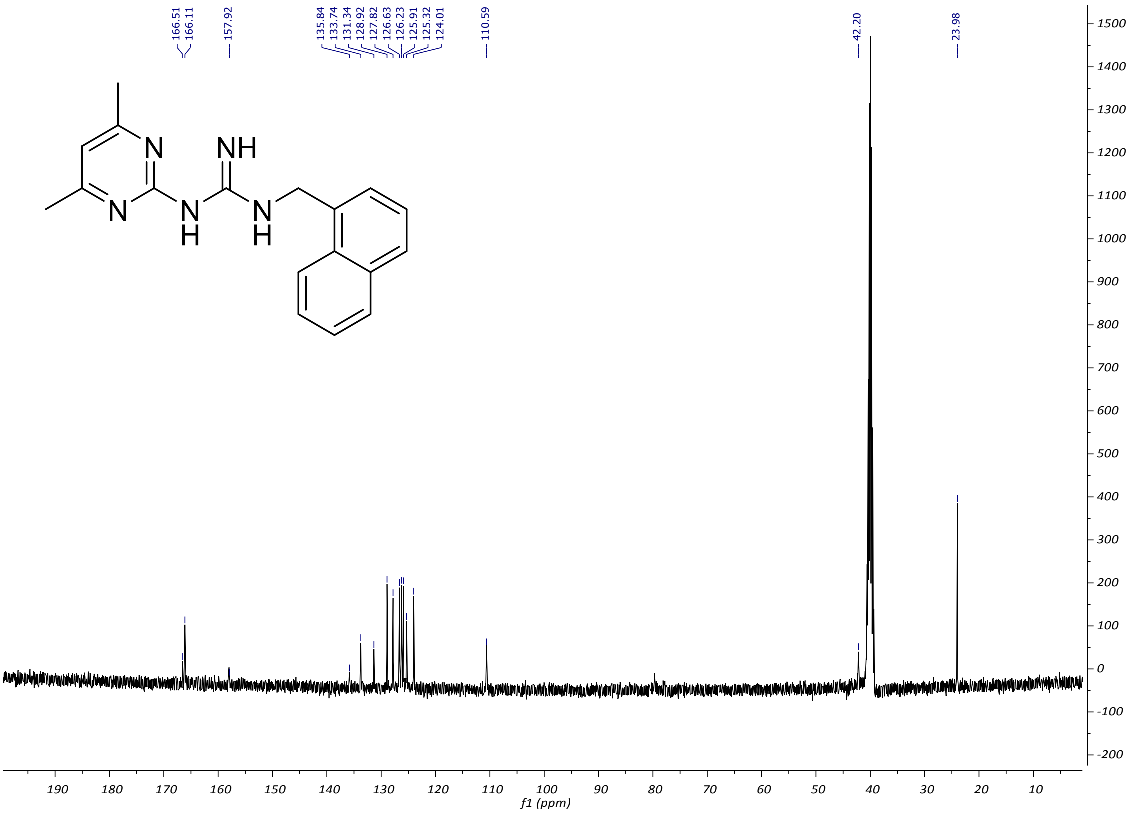

Figure S47. ^{1}H and ^{13}C{^{1}H} NMR spectra of 1-(4,6-dimethylpyrimidin-2-yl)-3-(naphthalen-1-ylmethyl)guanidine (**5ci**).

^{1}H NMR of **5cj** (400 MHz, DMSO-d_6)

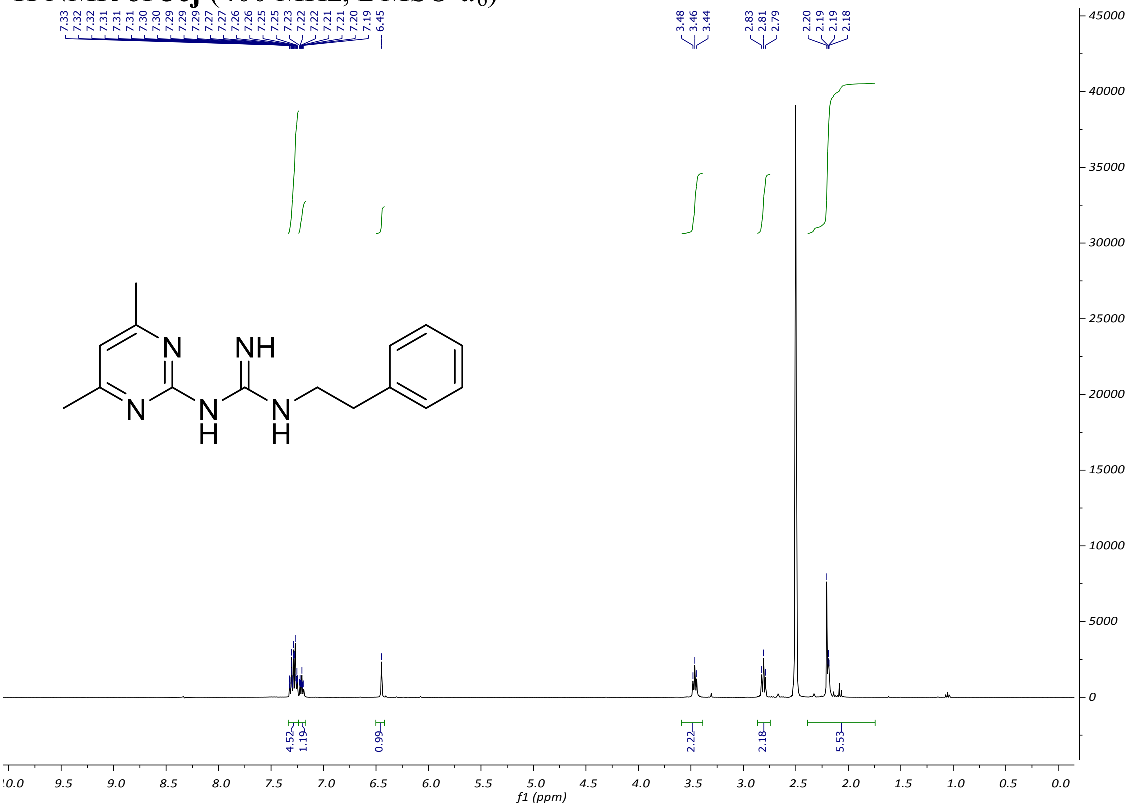

^{13}C{^{1}H} NMR of **5cj** (101 MHz, DMSO-d_6)

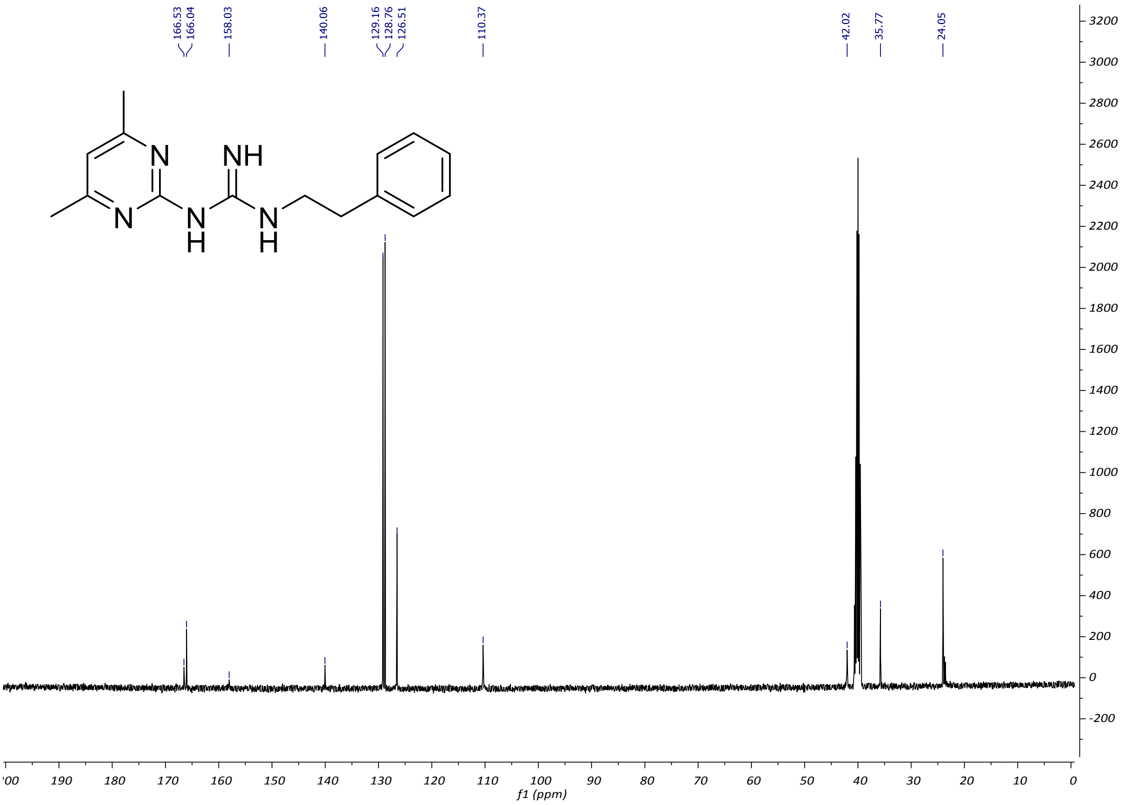

Figure S48. ^{1}H and ^{13}C{^{1}H} NMR spectra of 1-(4,6-dimethylpyrimidin-2-yl)-3-phenethylguanidine (**5cj**).
