# Supplementary material for: Lengthening the Guanidine–Aryl Linker of Phenylpyrimidinylguanidines Increases Their Potency as Inhibitors of FOXO3-Induced Gene Transcription
Source: ACS Omega. 2022 Sep 14;7(38):34632–46. doi: 10.1021/acsomega.2c04613 (PMC9521028; doi:10.1021/acsomega.2c04613)
Supplement: Supplementary file 2 — ao2c04613_si_002.zip [file ao2c04613_si_002.zip › 1-(4,6-dimethylpyrimidin-2-yl)-3-(4-methylbenzyl)guanidine_(5cd).pdf]

Automatic Evaluation Report from CSEARCH
created on 2022-08-09 at 16:51:29
based on 340,554 reference spectra

**Did you know ?**	**You can check the integrity of any webpage generated by the CSEARCH-Robot-Referee using the mechanism available on http://nmrpredict.orc.univie.ac.at/c13robot/signaturecheck.php**

Request from: vojtech.docekal@natur.cuni.cz

Compound: 1-[4,6-Dimethylpyrimidin-2-yl_]-3-[4-methylbenzyl_]guanidine_[5cd_]

Project: Lengthening_the_Guanidine-Aryl_Linker_of_Phenylpyrimidinylguanidines_Increases_t

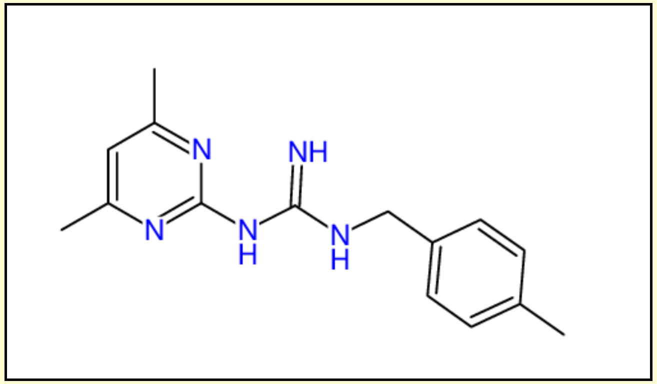

Database	Number of Entries	Owner of Database
**Please cite the CSEARCH-Robot-Referee as:**<br>**N. Haider, W. Robien; http://nmrpredict.orc.univie.ac.at/c13robot/robot.php**		

CSEARCH	74,997 (A)	CSEARCH-Data / Wolfgang Robien
CSEARCH	56,549 (B)	CSEARCH-Data / Wolfgang Robien
CSEARCH	28,196 (C)	CSEARCH-Data / Wolfgang Robien
CSEARCH	33,587 (D)	CSEARCH-Data / Wolfgang Robien
CSEARCH	39,132 (E)	CSEARCH-Data / Wolfgang Robien + NMR-Database University of Mainz
CSEARCH	26,196 (F)	CSEARCH-Data / Wolfgang Robien
CSEARCH	50,594 (I)	Upcoming CSEARCH-Data / Wolfgang Robien
CSEARCH	31,307 (L)	NMRShiftDB-Data / Version February 2012

Permanent URL

This page can be verified by a digital signature

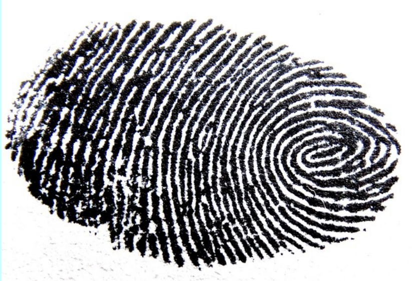

CSEARCH-Version: 9.4.0
Robot-Referee: 2017:06:10

Accept Minor revision CSEARCH Robot-Referee Major revision Reject

Request from: vojtech.docekal@natur.cuni.cz

Compound: 1-[4,6-Dimethylpyrimidin-2-yl_]-3-[4-methylbenzyl_]guanidine_[5cd_]

Project: Lengthening_the_Guanidine-Aryl_Linker_of_Phenylpyrimidinylguanidines_Increases_t

Accept Minor revision CSEARCH Robot-Referee Major revision Reject

Recommendation given here

Details of Prediction given here

Summary of Supplied Data

Understanding the Color Coding Scheme

Structure Proposal

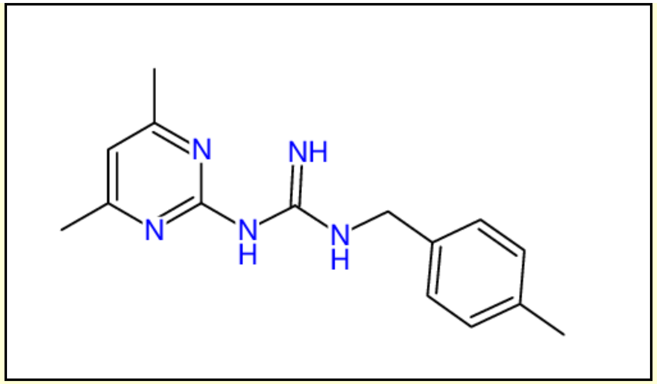

Molecular formula is: $C_{15}H_{19}N_5$ Molecular weight is: 269.36 amu

INCHIKEY is: YXSUMTZEYLAKDC-UHFFFAOYAQ

Numbering Scheme derived from the drawing sequence used during the calculation

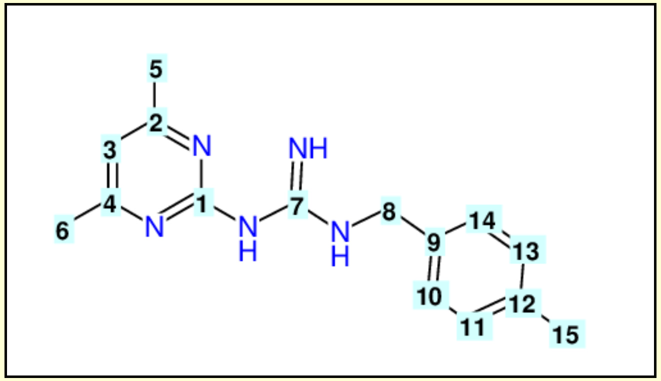

The marked carbons have been fully assigned

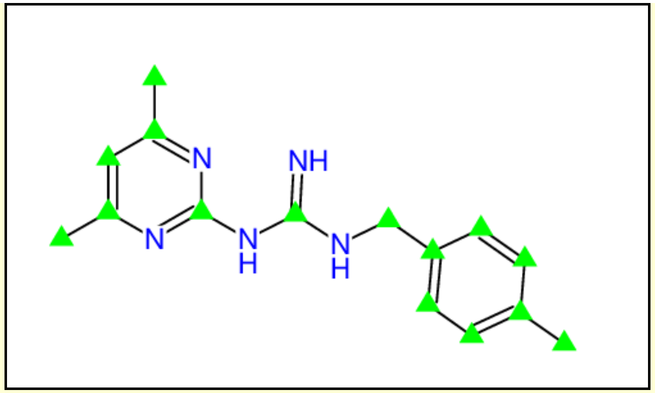

Carbon number	Chemical Shift Value	Multiplicity from Structure	Multiplicity from Experiment
1	158.20	S	-
2	166.10	S	-
3	110.50	D	-
4	166.10	S	-
5	24.00	Q	-
6	24.00	Q	-
7	166.50	S	-
8	43.80	T	-
9	136.10	S	-
10	127.50	D	-
11	129.30	D	-
12	137.60	S	-
13	129.30	D	-
14	127.50	D	-
15	21.10	Q	-

The marked carbons have been fully assigned

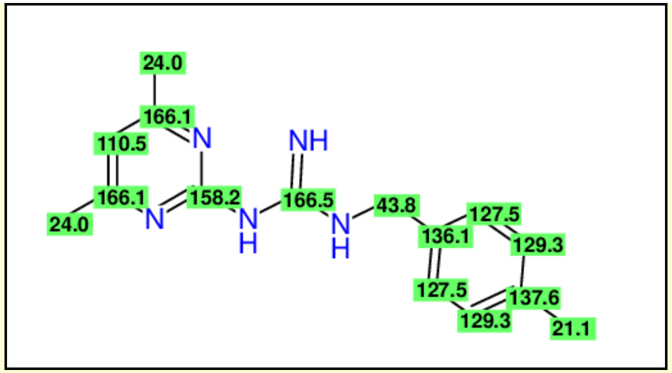

Graphical summary of the Chemical Shift Data

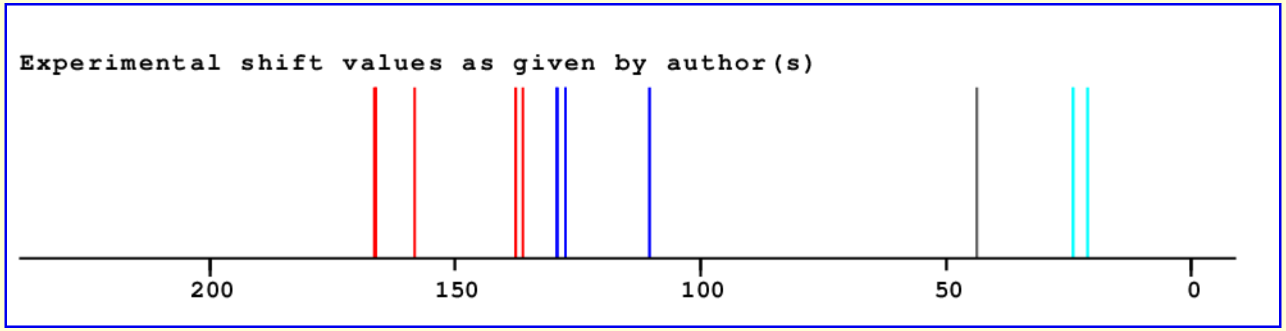

Searching external databases

Recall this Compound from PUBCHEM (Stereo-Match from searching 146,705,909 compounds)

Stereo-match found at Emolecules - 4,400,967 Compounds searched

Search the Internet for this compound (Skeleton only)
Search the Internet for this compound (Skeleton + Stereochemistry)

Search CHEMSPIDER for this compound (Skeleton only)
Search CHEMSPIDER for this compound (Skeleton + Stereochemistry)

Search the Internet for the molecular formula $C_{15}H_{19}N_5$

Search CHEMSPIDER for the molecular formula $C_{15}H_{19}N_5$

(Description)

Basic Evaluation: Checking Multiplicities

Checking lines & multiplicity	Carbons/Lines	Singlet	Dublet	Triplet	Quartet	Odd	Even	None
From structure	15	6	5	1	3	7	8	0
From spectrum	15	6	5	1	3	7	8	0

Overall impression on compatibility of multiplicity from structure and experiment

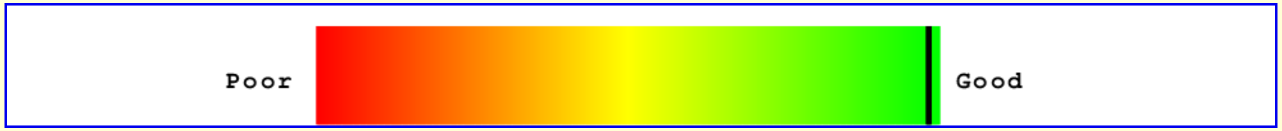

Evaluation based on Spectrum Prediction

Numbering Scheme

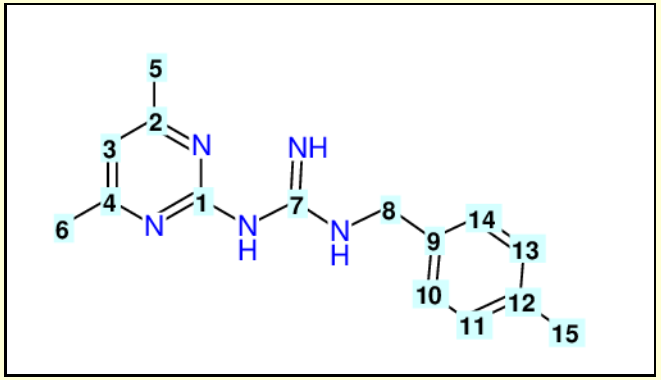

Carbon Number ▲▼	Neural Network Prediction ▲▼	HOSE-Code Prediction ▲▼	Preferred Value from both Predictions ▲▼	Experimental values ▲▼	Difference (Exp-Pred/ppm) ▲▼	Assignment	Prediction Quality
1	155.7	160.0	157.8	158.2	0.4	Assigned by author	Only reference material with low similarity
2	164.4	167.5	165.9	166.1	0.2	Assigned by author	
3	120.5	114.5	115.7	110.5	5.2	Assigned by author	Large Difference between NET & HOSE
4	164.4	167.5	165.9	166.1	0.2	Assigned by author	
5	24.8	23.7	23.9	24.0	0.1	Assigned by author	
6	24.8	23.7	23.9	24.0	0.1	Assigned by author	
7	164.7	156.8	160.7	166.5	5.8	Assigned by author<br>Check assignment - maybe 166.10<br>?	Large Difference between NET & HOSE<br>Only reference material with low similarity<br>Only very few similar structures
8	46.1	45.7	45.8	43.8	2.0	Assigned by author	
9	135.5	136.0	135.8	136.1	0.3	Assigned by author<br>Check assignment - maybe 137.60<br>?	
10	128.5	126.5	126.9	127.5	0.6	Assigned by author	
11	129.1	129.9	129.9	129.3	0.6	Assigned by author	
12	136.5	136.0	136.0	137.6	1.6	Assigned by author<br>Check assignment - maybe 136.10<br>?	
13	129.1	129.9	129.9	129.3	0.6	Assigned by author	
14	128.5	126.5	126.9	127.5	0.6	Assigned by author	

Carbon Number ▴▾	Neural Network Prediction ▴▾	HOSE-Code Prediction ▴▾	Preferred Value from both Predictions ▴▾	Experimental values ▴▾	Difference (Exp-Pred/ppm) ▴▾	Assigned by author Assignment	Prediction Quality
15	21.2	21.1	21.1	21.1	0.0		
Absolute<br>Signed	1.74ppm (15)<br>-0.41ppm (15)	0.73ppm (15)<br>0.15ppm (15)	1.22ppm (15)<br>0.09ppm (15)			1.19ppm (15)<br>0.09ppm (15)	Average deviation to experimental values<br>(Number of shift pairs used)

Structure representation by reference data over 3.7 shells on average

Visualization of the differences between predicted and experimental values

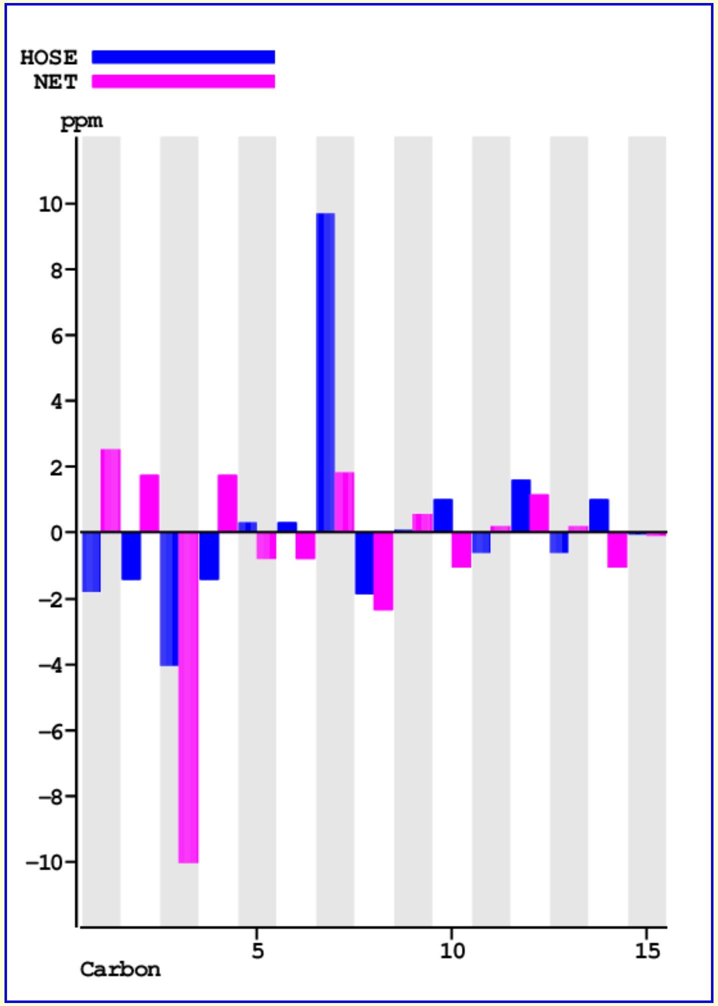

Quality of the Spectrum Prediction

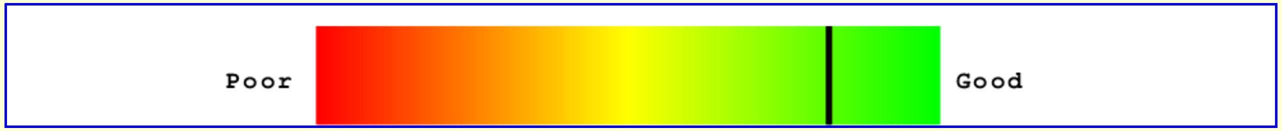

Experimental Chemical Shift Values as given

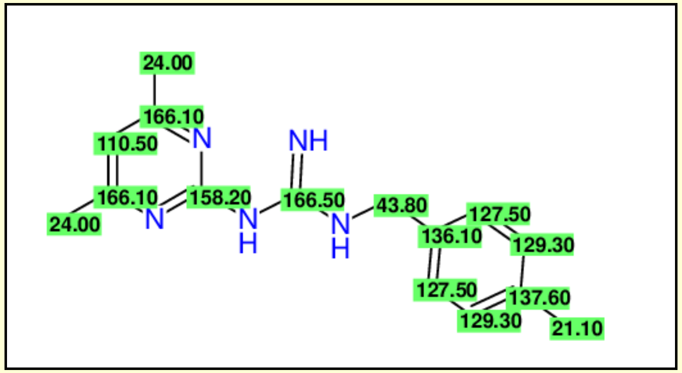

Experimental Chemical Shift Values using Symmetry

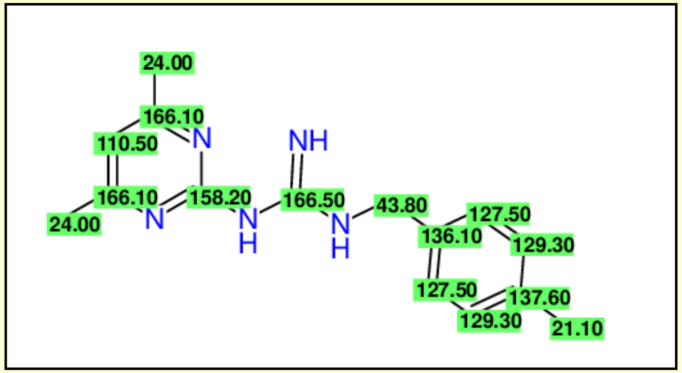

Preferred Chemical Shift Values from both predictions

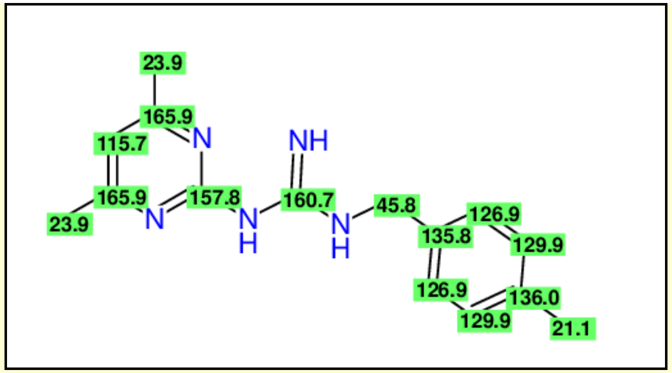

[Comparison of Prediction Techniques]()

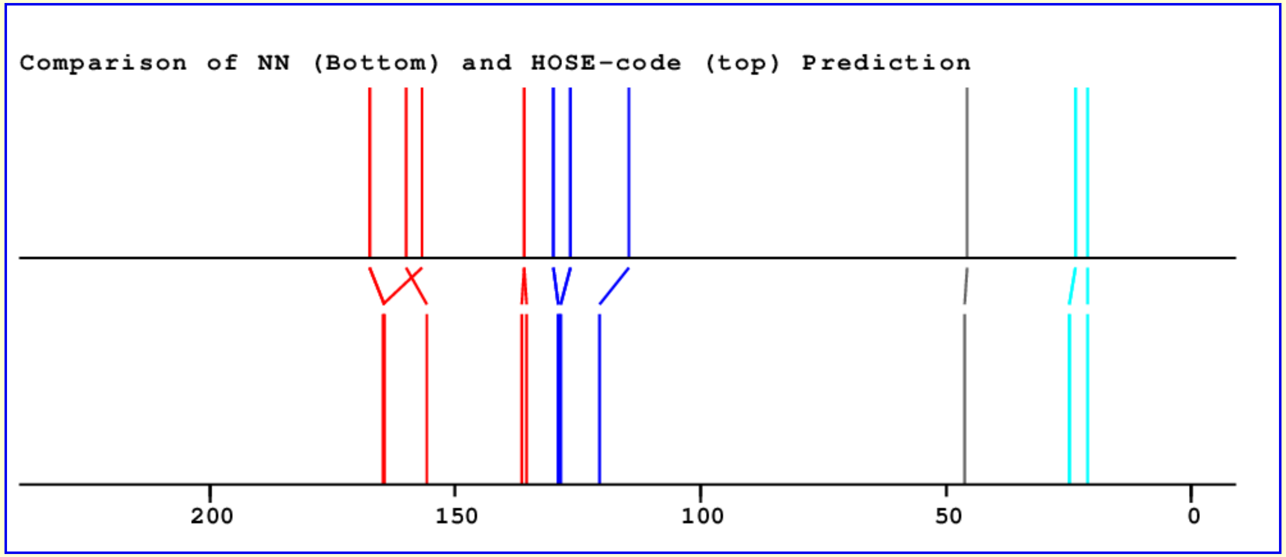

[Contribution of the methods]()

HOSE	NET	NET&HOSE	NONE

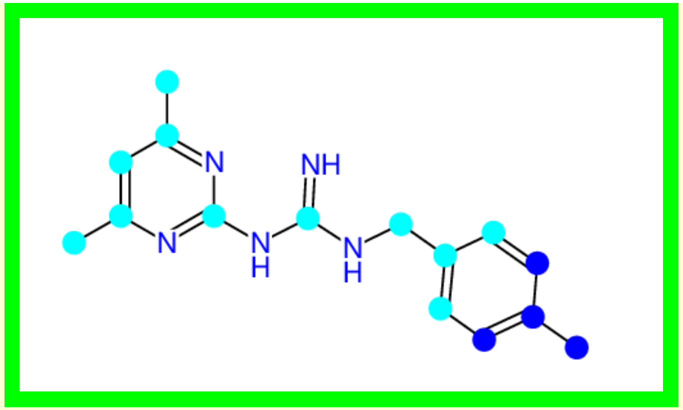

Similarity between predicted and experimental data based on positions

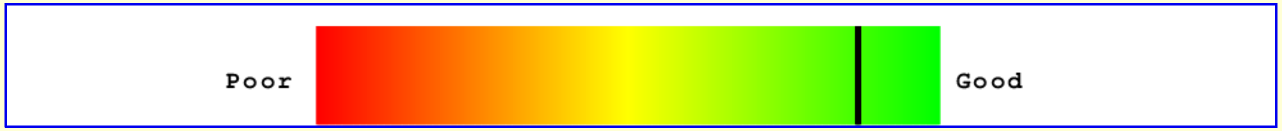

Matching map of predicted versus experimental data

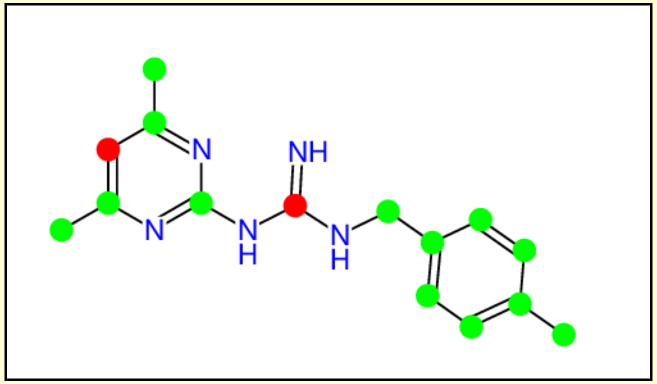

Differences between predicted and experimental data in ppm

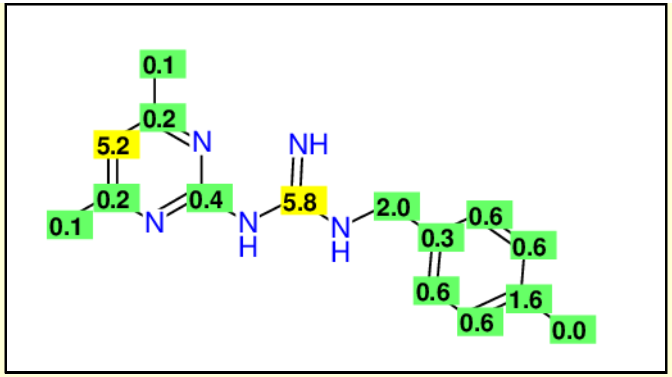

Comparison of Experimental versus Predicted Chemical Shift Values

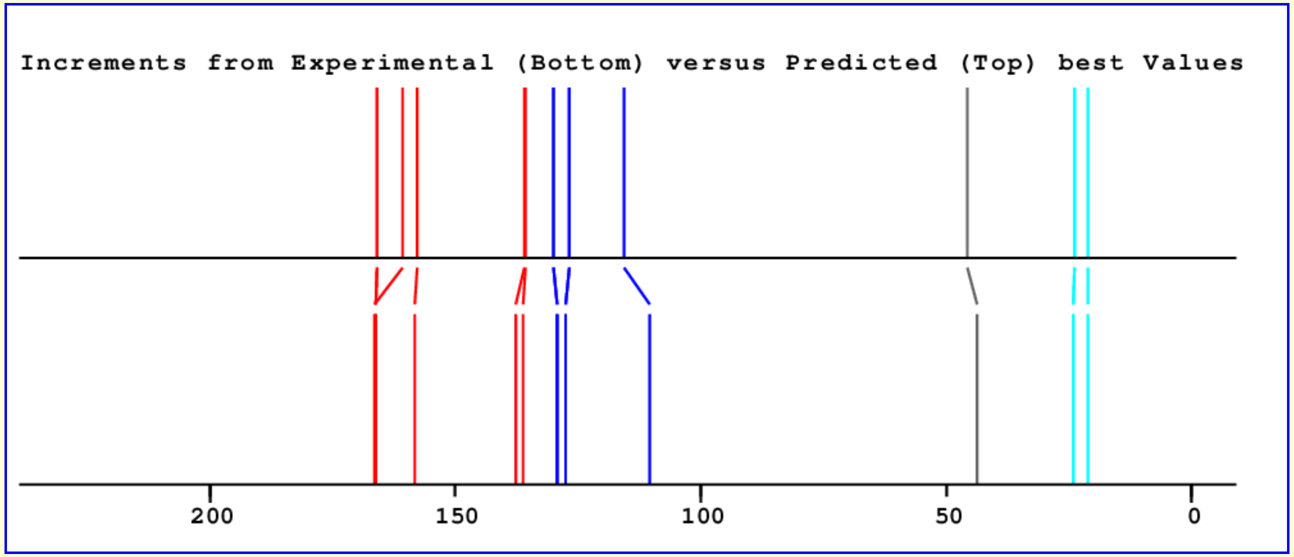

Overall deviation between predicted and experimental data is 1.2ppm

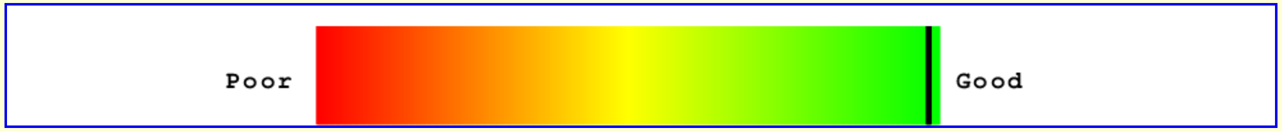

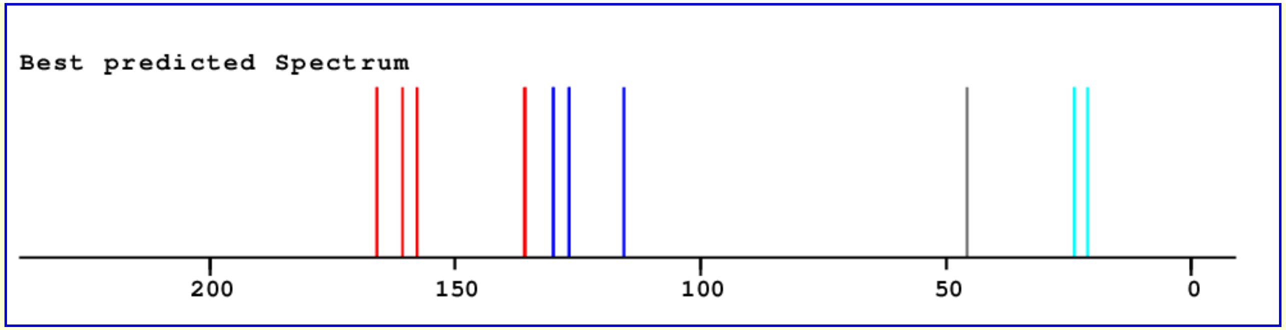

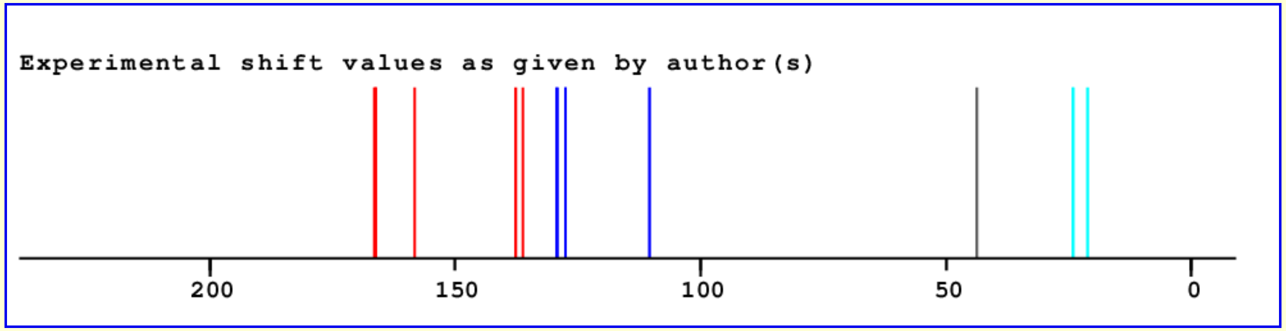

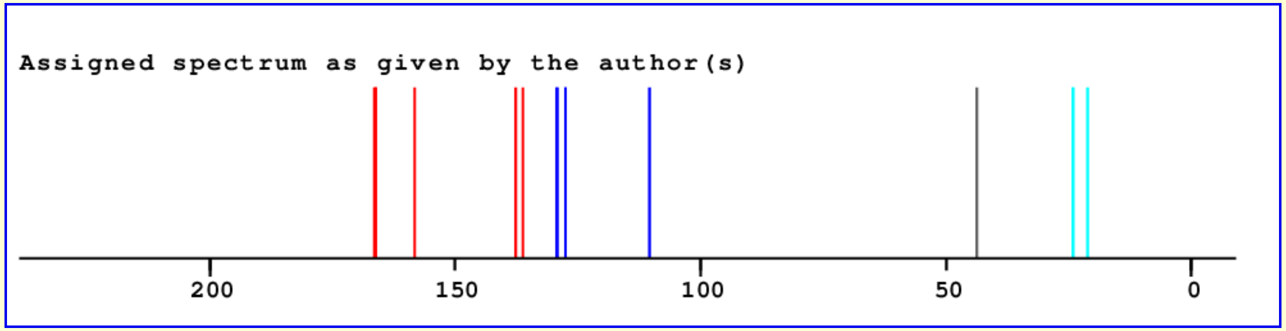

	Your assignment	Difference to predicted values

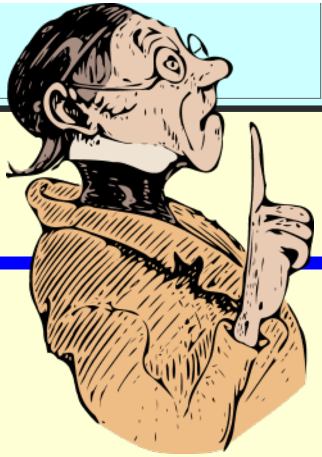

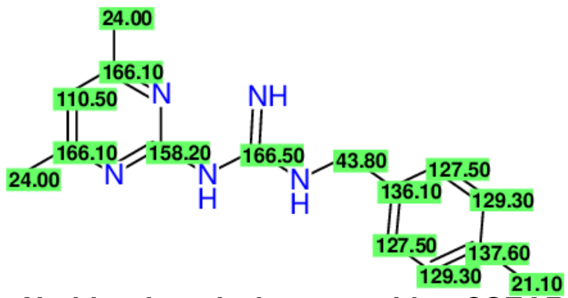

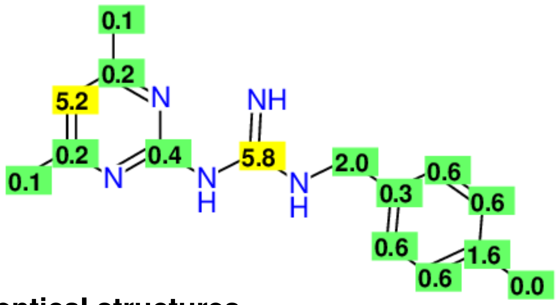

Nothing found when searching CSEARCH for identical structures

(Description)

No alternative structure found when searching CSEARCH for identical spectra

(Description)

Overall Impression

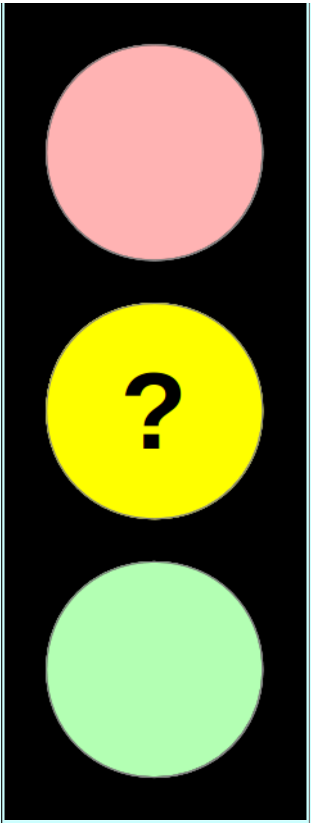

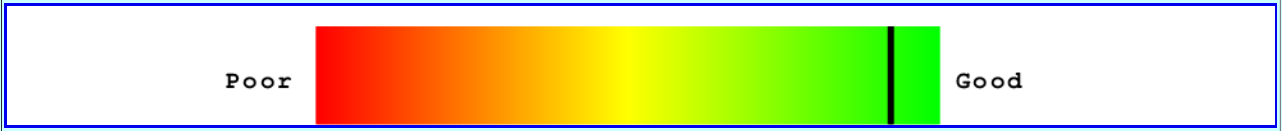

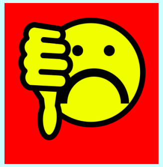

Minor revision might be necessary - please check

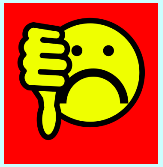

Compound: 1-[4,6-Dimethylpyrimidin-2-yl_]-3-[4-methylbenzyl_]guanidine_[5cd_]

Project: Lengthening_the_Guanidine-Aryl_Linker_of_Phenylpyrimidinylguanidines_Increases_t

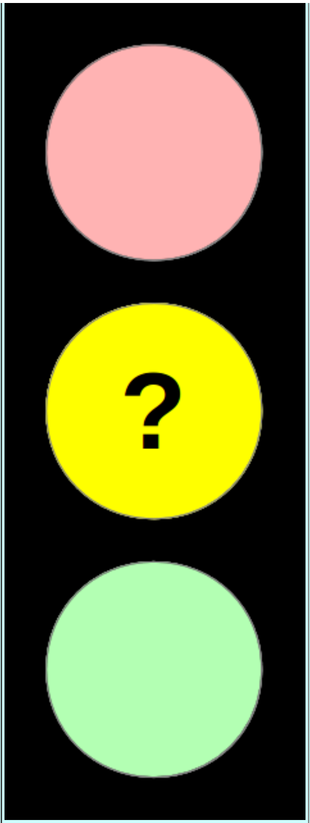

The CSEARCH Robot Referee recommends: Minor revision might be necessary - please check

Check integrity of page via electronic fingerprint

- **NN-Prediction and HOSE-Code prediction differs significantly at 2 carbon positions**
- **Assignment can be probably improved at 3 positions**
- **2 Carbon positions (out of 15) have a severe assignment problem**
- **Spectrum prediction - minor inconsistencies found**

Experimental values

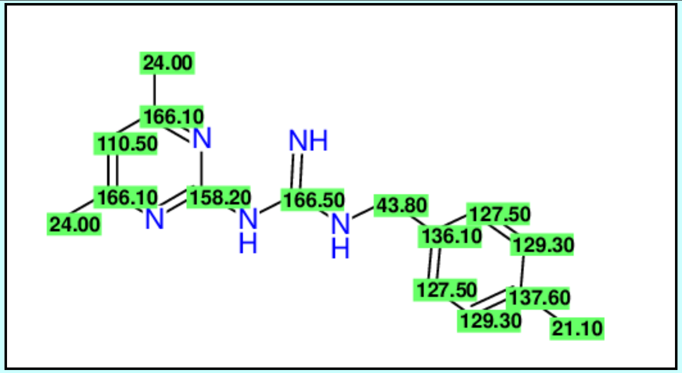

Predicted values

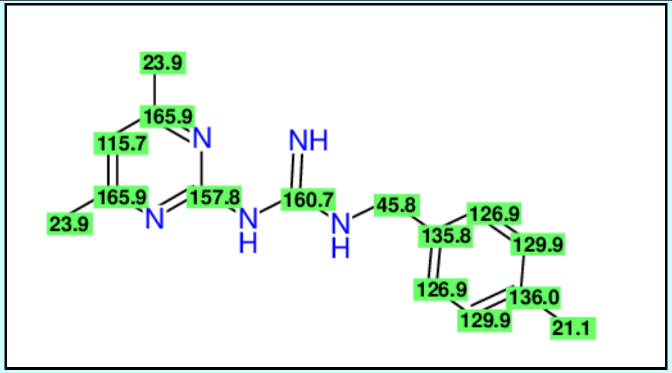

Matching map

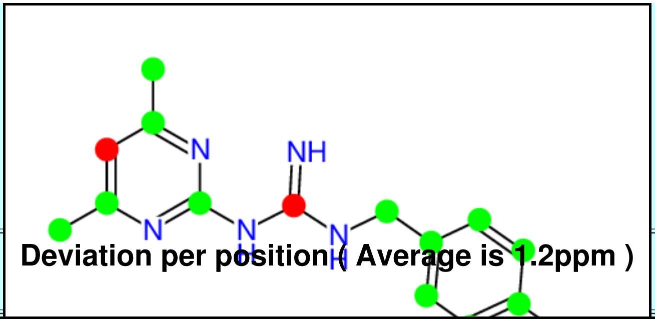

Deviation per position (Average is 1.2ppm)

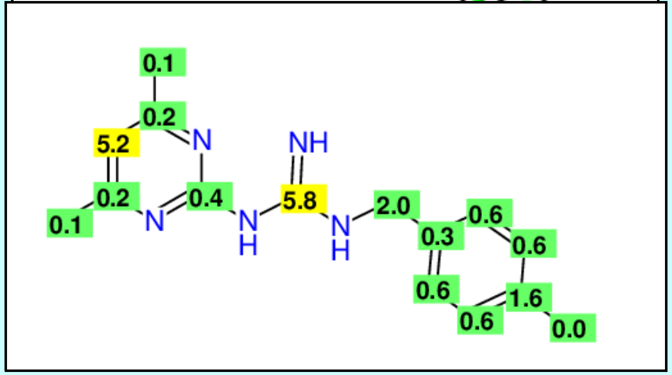

Contribution of methods

HOSE NET NET&HOSE NONE

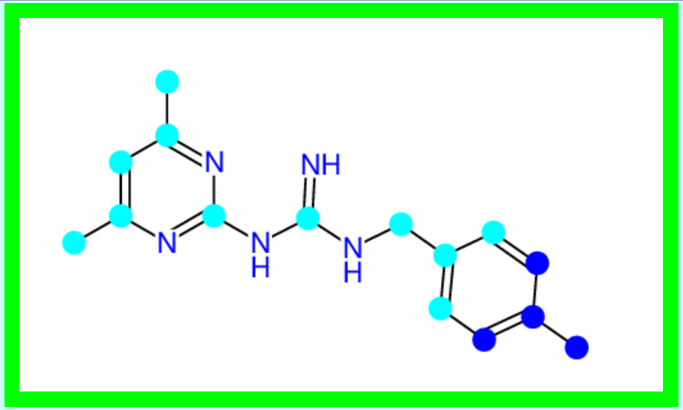

Overall Similarity Index is 1.8

0.0 is a "perfect match", up to approximately 3.0 it is "reasonable", above 5.0 it is more or less "unbelievable"

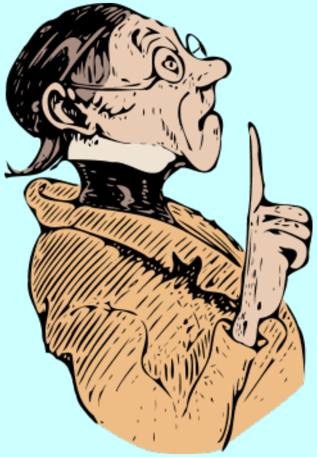

Your assignment	Difference to predicted values
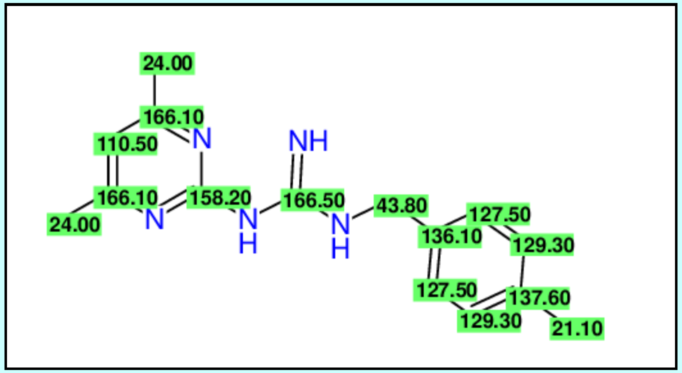	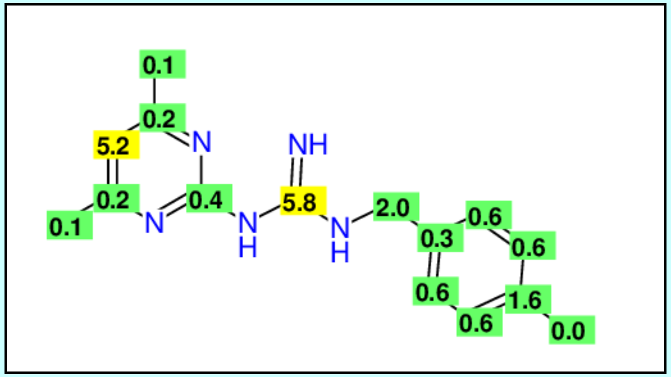

Recall this Compound from PUBCHEM (Stereo-Match from searching 146,705,909 compounds)

Stereo-match found at Emolecules - 4,400,967 Compounds searched

Search the Internet for this compound (Skeleton only)
Search the Internet for this compound (Skeleton + Stereochemistry)

Search CHEMSPIDER for this compound (Skeleton only)
Search CHEMSPIDER for this compound (Skeleton + Stereochemistry)

Search the Internet for the molecular formula $C_{15}H_{19}N_5$

Search CHEMSPIDER for the molecular formula $C_{15}H_{19}N_5$

(Description)

History of your requests for this compound

Date/Time	Result	Method	Assigned Lines	Unassigned Lines	Stereoisomer	Permanent URL	Remark	Comparison of experimental and predicted data (Evaluation only)
2022-08-08 16:48:30	Minor	Evaluation	15	0	NO			Picture not available

This request	Minor	Evaluation	15	0		

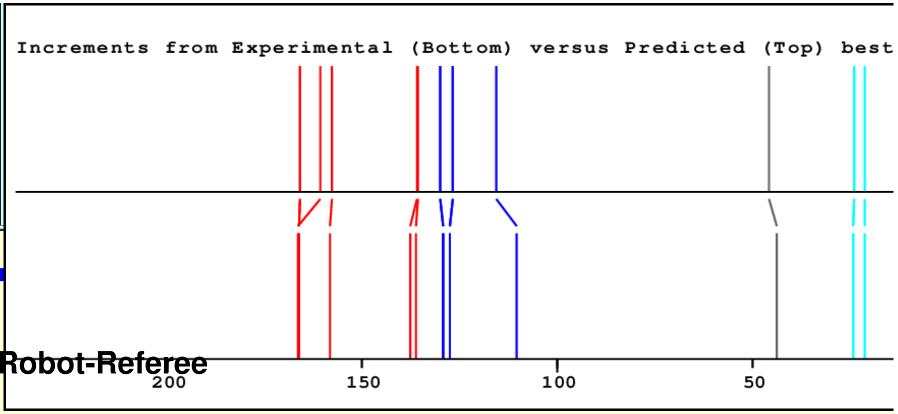

Your Total Usage of the CSEARCH-Robot-Referee

29 Requests have been launched by vojtech.docekal@natur.cuni.cz					
Year	Accept	Minor Revision	Major Revision	Reject	Only Prediction
2022		26	3		

Top

Page has been automatically written by CSEARCH
CPU-Usage: Evaluation needed 8.007 seconds
Wolfgang.Robien(at)univie.ac.at
