# Supplementary material for: Lengthening the Guanidine–Aryl Linker of Phenylpyrimidinylguanidines Increases Their Potency as Inhibitors of FOXO3-Induced Gene Transcription
Source: ACS Omega. 2022 Sep 14;7(38):34632–46. doi: 10.1021/acsomega.2c04613 (PMC9521028; doi:10.1021/acsomega.2c04613)
Supplement: Supplementary file 2 — ao2c04613_si_002.zip [file ao2c04613_si_002.zip › 1-(4-(benzylamino)-6-methylpyrimidin-2-yl)-3-(4-propoxyphenyl)guanidine_(5da).pdf]

Automatic Evaluation Report from CSEARCH
created on 2022-08-08 at 13:10:31
based on 340,554 reference spectra

Did you know ?

Every webpage created by the CSEARCH-Robot-Referee is marked with a digital fingerprint - editing of these webpages is therefore prohibited ! The chance to manipulate such a page successfully is like isolating one, single molecule out of one Peta-MOL of substance !

Request from: vojtech.docekal@natur.cuni.cz

Compound: 1-[4-[Benzylamino_]-6-methylpyrimidin-2-yl_]-3-[4-propoxyphenyl_]guanidine_[

Project: Lengthening_the_Guanidine-Aryl_Linker_of_Phenylpyrimidinylguanidines_Increases_t

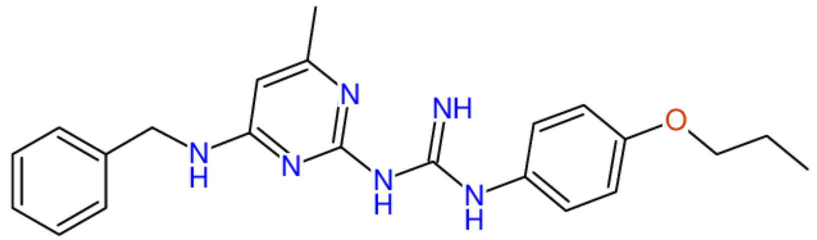

Database	Number of Entries	Owner of Database
Please cite the CSEARCH-Robot-Referee as:<br>N. Haider, W. Robien; http://nmrpredict.orc.univie.ac.at/c13robot/robot.php		

CSEARCH	74,997 (A)	CSEARCH-Data / Wolfgang Robien
CSEARCH	56,549 (B)	CSEARCH-Data / Wolfgang Robien
CSEARCH	28,196 (C)	CSEARCH-Data / Wolfgang Robien
CSEARCH	33,587 (D)	CSEARCH-Data / Wolfgang Robien
CSEARCH	39,132 (E)	CSEARCH-Data / Wolfgang Robien + NMR-Database University of Mainz
CSEARCH	26,196 (F)	CSEARCH-Data / Wolfgang Robien
CSEARCH	50,594 (I)	Upcoming CSEARCH-Data / Wolfgang Robien
CSEARCH	31,307 (L)	NMRShiftDB-Data / Version February 2012

Permanent URL

This page can be verified by a digital signature

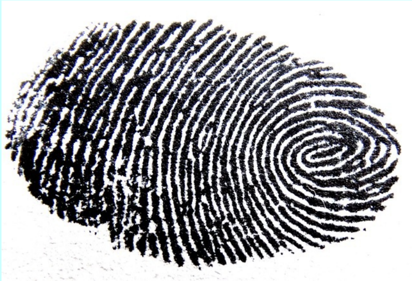

CSEARCH-Version: 9.4.0
Robot-Referee: 2017:06:10

Request from: vojtech.docekal@natur.cuni.cz

Compound: 1-[4-[Benzylamino_]-6-methylpyrimidin-2-yl_]-3-[4-propoxyphenyl_]guanidine_[

Project: Lengthening_the_Guanidine-Aryl_Linker_of_Phenylpyrimidinylguanidines_Increases_t

Recommendation given here

Details of Prediction given here

Summary of Supplied Data

Understanding the Color Coding Scheme

Structure Proposal

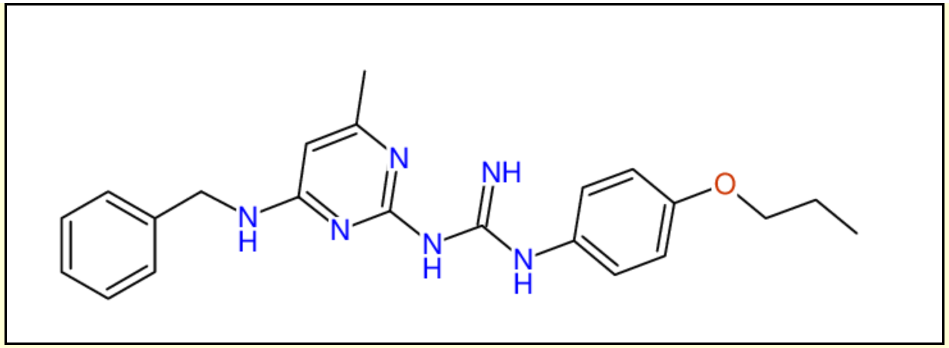

Molecular formula is: $C_{22}H_{26}N_6O$ **Molecular weight is: 390.50 amu**

INCHIKEY is: XNPGWMPKIOFQJF-UHFFFAOYAG

Numbering Scheme derived from the drawing sequence used during the calculation

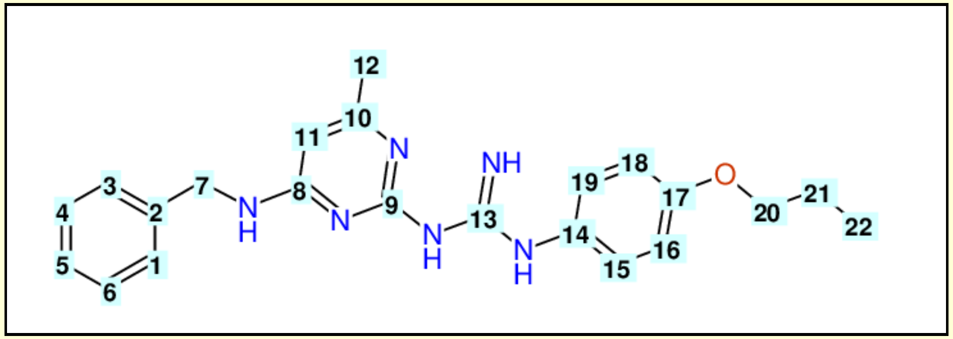

The marked carbons have been fully assigned

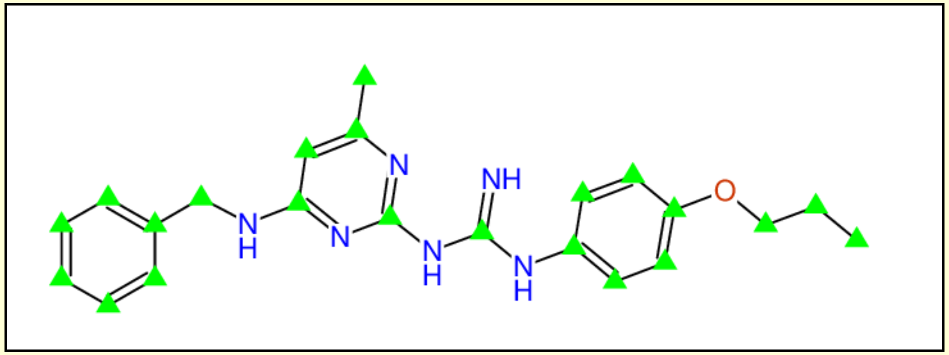

Carbon number	Chemical Shift Value	Multiplicity from Structure	Multiplicity from Experiment
1	126.90	D	-
2	140.60	S	-
3	126.90	D	-
4	126.70	D	-
5	126.40	D	-
6	126.70	D	-
7	45.70	T	-
8	164.00	S	-
9	155.00	S	-
10	164.80	S	-
11	94.70	D	-
12	24.00	Q	-
13	159.50	S	-
14	138.90	S	-
15	128.40	D	-
16	115.50	D	-
17	152.30	S	-
18	115.50	D	-
19	128.40	D	-
20	69.80	T	-
21	22.70	T	-
22	10.60	Q	-

The marked carbons have been fully assigned

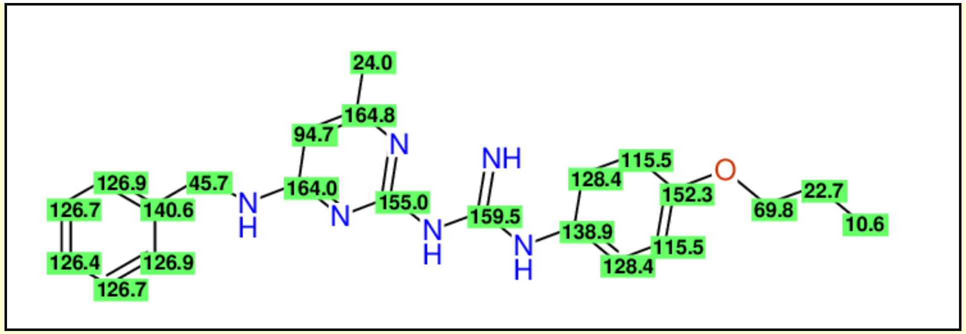

Graphical summary of the Chemical Shift Data

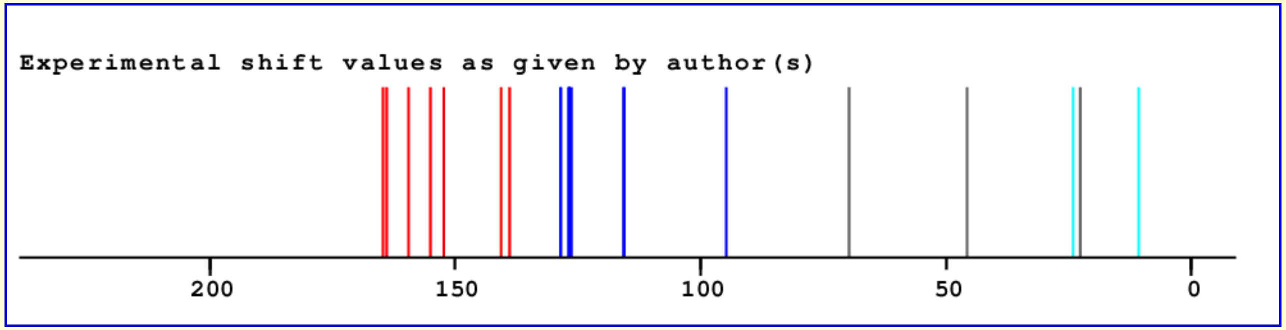

Searching external databases

146,705,909 Compounds searched in PUBCHEM - nothing found

4,400,967 Compounds searched in Emolecules - nothing found

Search the Internet for this compound (Skeleton only)
Search the Internet for this compound (Skeleton + Stereochemistry)

Search CHEMSPIDER for this compound (Skeleton only)
Search CHEMSPIDER for this compound (Skeleton + Stereochemistry)

Search the Internet for the molecular formula $C_{22}H_{26}N_6O$

Search CHEMSPIDER for the molecular formula $C_{22}H_{26}N_6O$

(Description)

Basic Evaluation: Checking Multiplicities

Checking lines & multiplicity	Carbons/Lines	Singlet	Dublet	Triplet	Quartet	Odd	Even	None
From structure	22	7	10	3	2	10	12	0
From spectrum	22	7	10	3	2	10	12	0

Overall impression on compatibility of multiplicity from structure and experiment

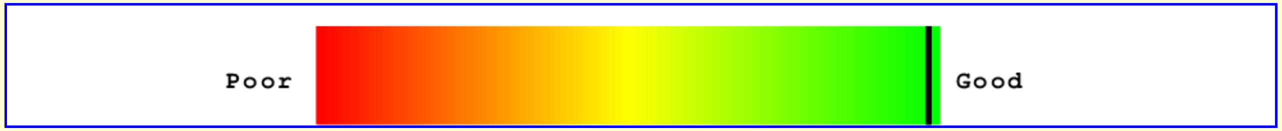

Evaluation based on Spectrum Prediction

Numbering Scheme

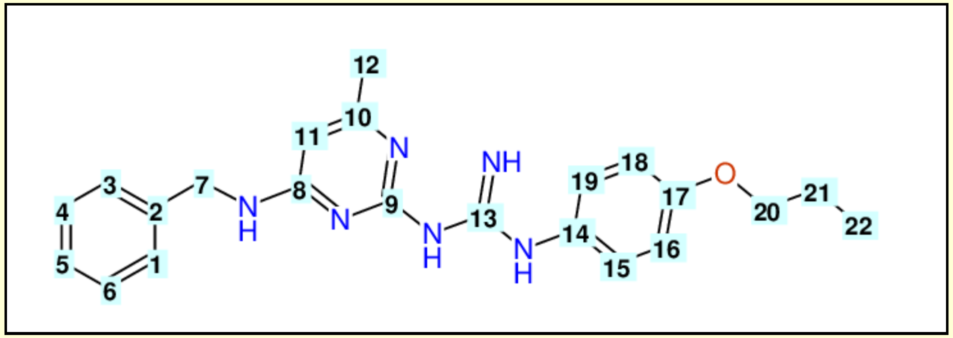

Carbon Number ▴▾	Neural Network Prediction ▴▾	HOSE-Code Prediction ▴▾	Preferred Value from both Predictions ▴▾	Experimental values ▴▾	Difference (Exp-Pred/ppm) ▴▾	Assignment	Prediction Quality
1	127.6	127.9	127.9	126.9	1.0	Assigned by author<br>Check assignment - maybe 128.40<br>?	
2	139.8	140.1	140.1	140.6	0.5	Assigned by author	
3	127.6	127.9	127.9	126.9	1.0	Assigned by author<br>Check assignment - maybe 128.40<br>?	
4	128.5	128.0	128.0	126.7	1.3	Assigned by author<br>Check assignment - maybe 128.40<br>?	
5	126.9	127.6	127.6	126.4	1.2	Assigned by author<br>Check assignment - maybe 126.90<br>?	
6	128.5	128.0	128.0	126.7	1.3	Assigned by author<br>Check assignment - maybe 128.40<br>?	
7	43.8	45.0	44.7	45.7	1.0	Assigned by author	
8	155.0	165.9	160.5	164.0	3.5	Assigned by author	Large Difference between NET & HOSE<br>Only reference material with low similarity
9	154.8	160.0	157.4	155.0	2.4	Assigned by author<br>Check assignment - maybe 152.30<br>?	Large Difference between NET & HOSE<br>Only reference material with low similarity
10	169.1	164.5	166.8	164.8	2.0	Assigned by author	Only very few similar structures
11	98.8	104.0	101.4	94.7	6.7	Assigned by author	Large Difference between NET & HOSE

Carbon Number ▴▾	Neural Network Prediction ▴▾	HOSE-Code Prediction ▴▾	Preferred Value from both Predictions ▴▾	Experimental values ▴▾	Difference (Exp-Pred/ppm) ▴▾	Assignment	Prediction Quality
12	24.8	23.3	23.6	24.0	0.4	Assigned by author	Only reference material with low similarity
13	163.7	156.9	160.3	159.5	0.8	Assigned by author	Large Difference between NET & HOSE<br>Only reference material with low similarity<br>Only very few similar structures
14	134.8	138.7	136.7	138.9	2.2	Assigned by author	
15	122.0	122.2	122.1	128.4	6.3	Assigned by author<br>Check assignment - maybe 126.40 ?	
16	115.1	115.8	115.7	115.5	0.2	Assigned by author	
17	153.0	159.2	156.1	152.3	3.8	Assigned by author<br>Check assignment - maybe 155.00 ?	Large Difference between NET & HOSE
18	115.1	115.8	115.7	115.5	0.2	Assigned by author	
19	122.0	122.2	122.1	128.4	6.3	Assigned by author<br>Check assignment - maybe 126.40 ?	
20	71.9	69.6	69.6	69.8	0.2	Assigned by author	
21	21.9	22.4	22.4	22.7	0.3	Assigned by author	
22	10.6	10.2	10.2	10.6	0.4	Assigned by author	
Absolute<br>Signed	2.37ppm (22)<br>0.39ppm (22)	2.16ppm (22)<br>-0.51ppm (22)	1.94ppm (22)<br>-0.03ppm (22)			1.61ppm (22)<br>-0.03ppm (22)	Average deviation to experimental values<br>(Number of shift pairs used)
Structure representation by reference data over 3.8 shells on average							

Visualization of the differences between predicted and experimental values

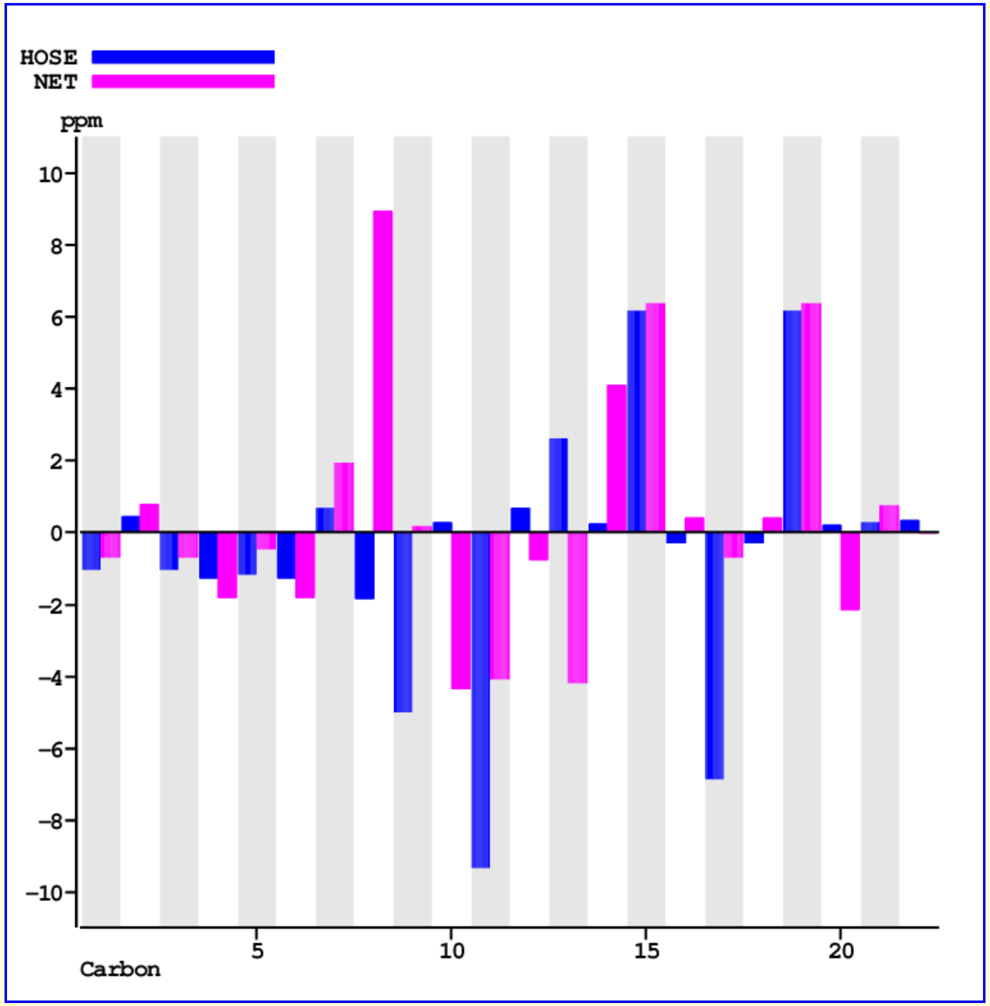

Quality of the Spectrum Prediction

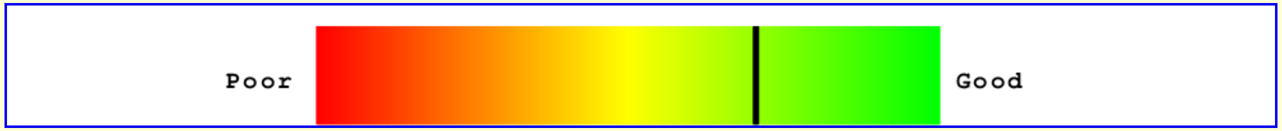

Experimental Chemical Shift Values as given

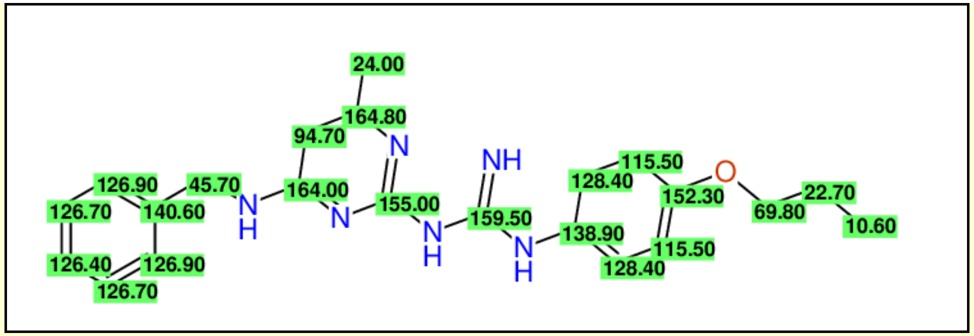

Experimental Chemical Shift Values using Symmetry

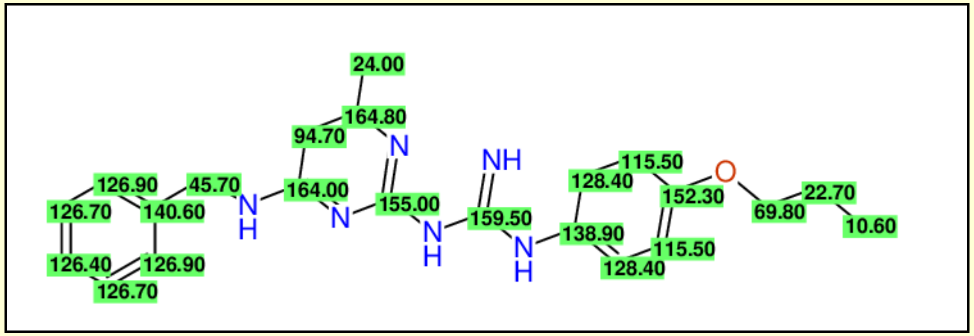

Preferred Chemical Shift Values from both predictions

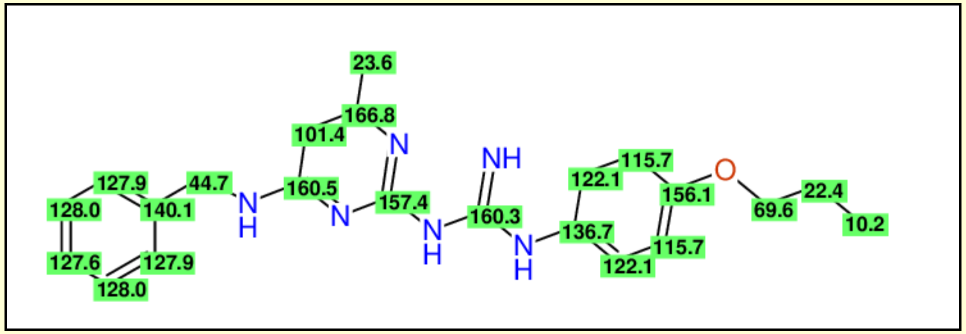

Comparison of Prediction Techniques

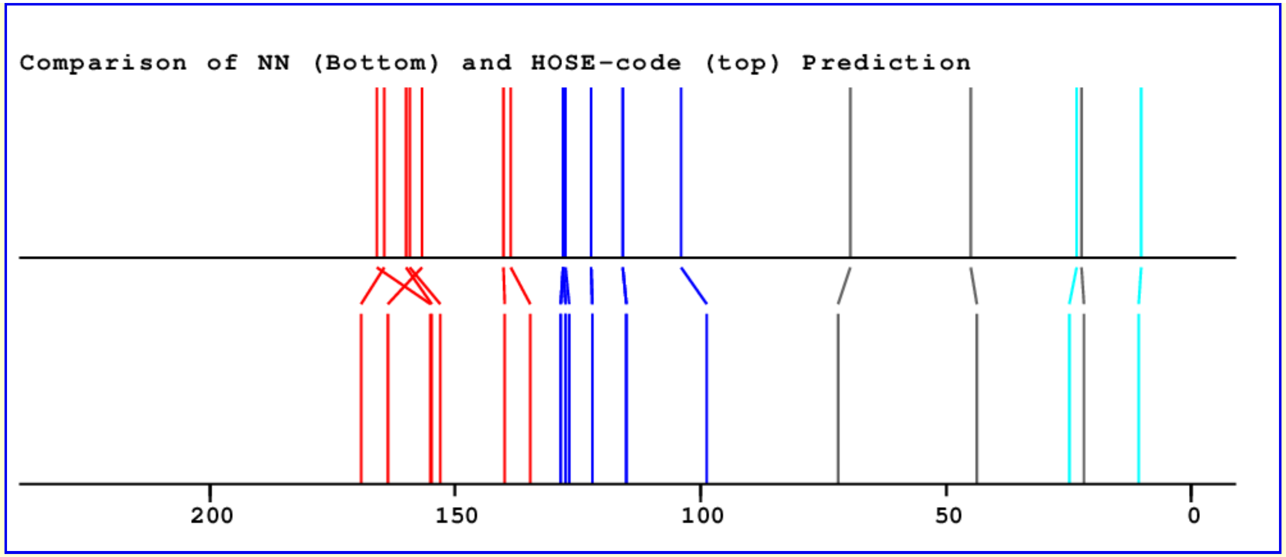

Contribution of the methods

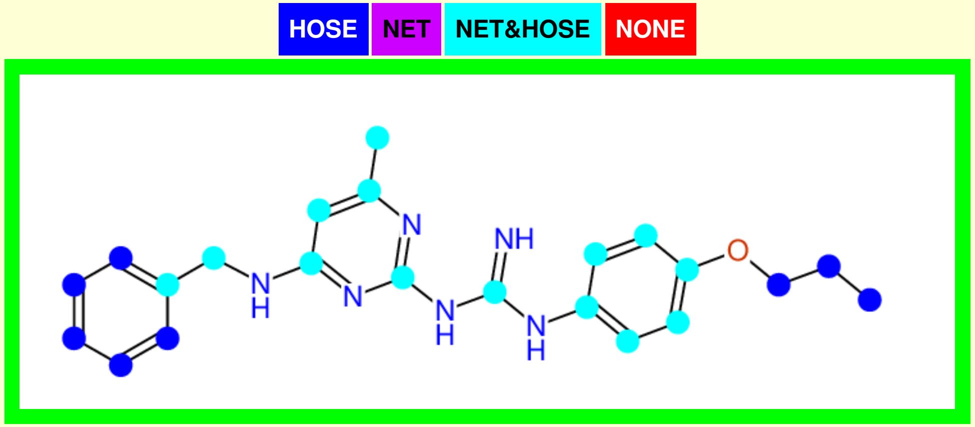

Similarity between predicted and experimental data based on positions

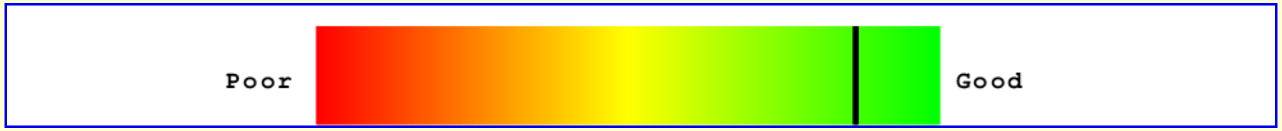

Matching map of predicted versus experimental data

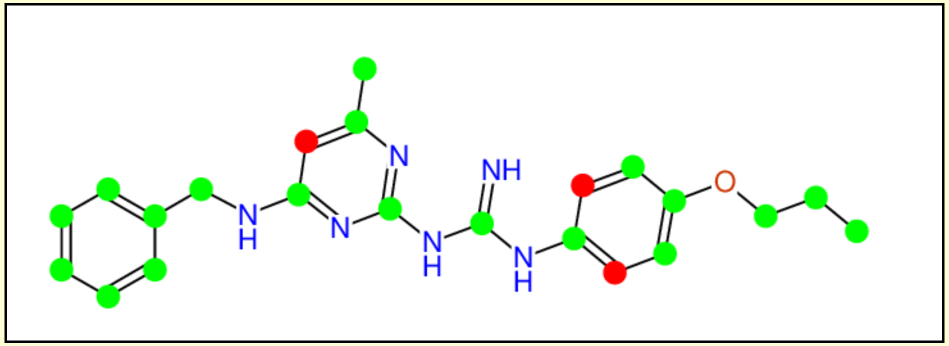

Differences between predicted and experimental data in ppm

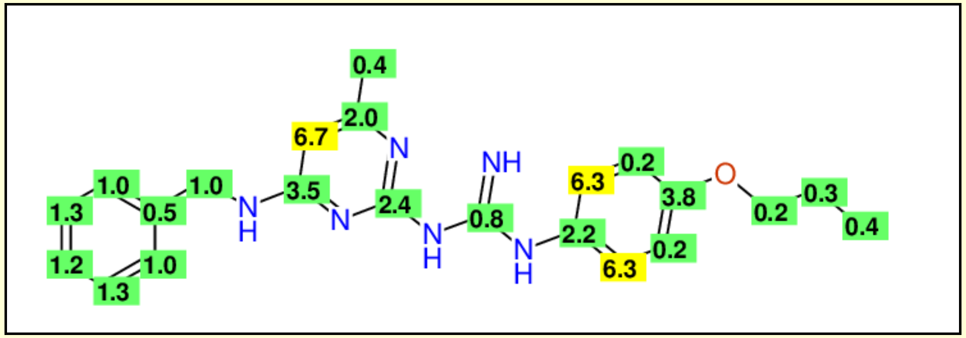

Comparison of Experimental versus Predicted Chemical Shift Values

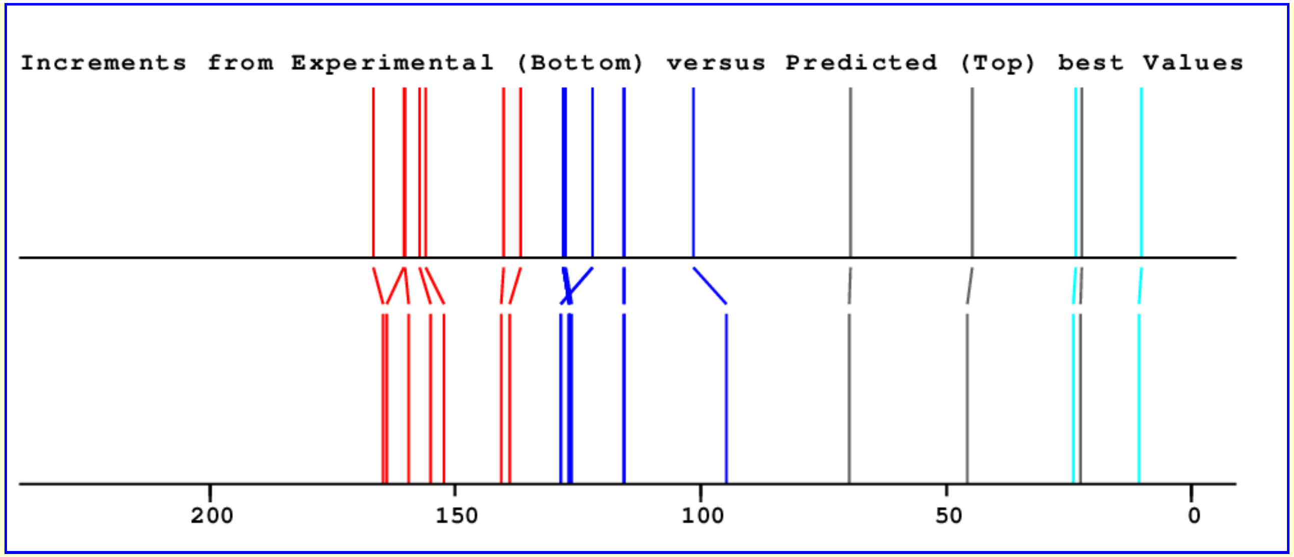

Overall deviation between predicted and experimental data is 1.9ppm

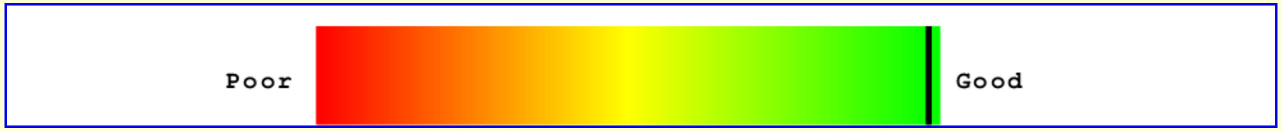

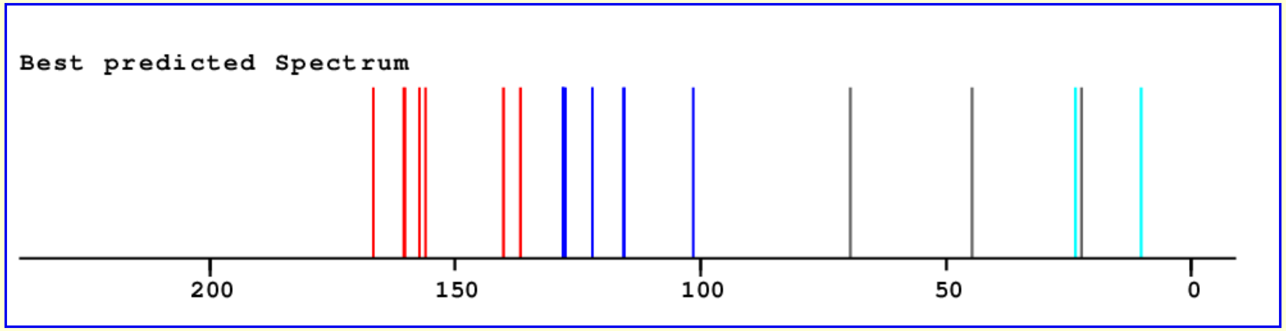

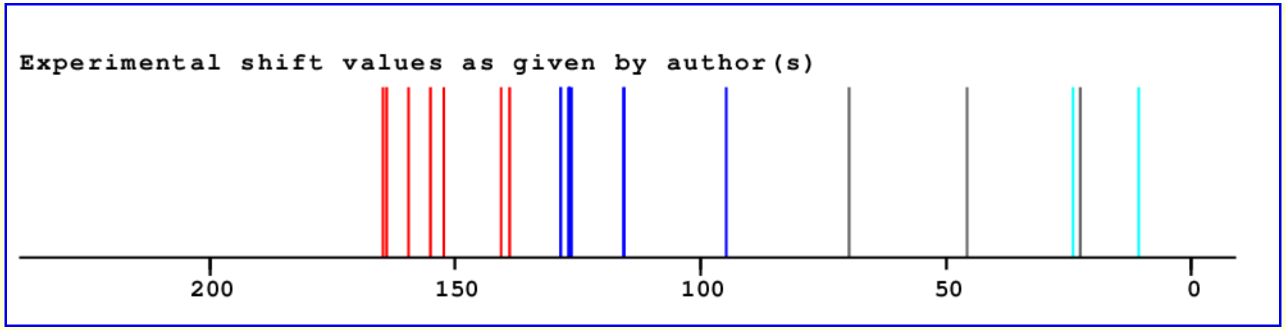

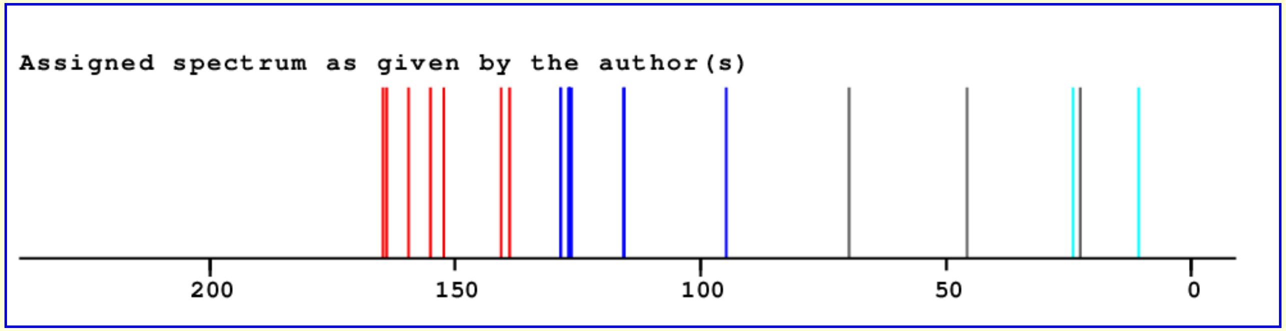

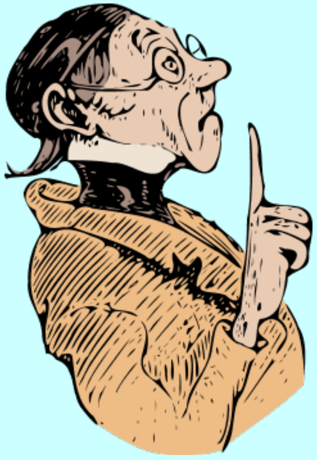

Your assignment	Difference to predicted values

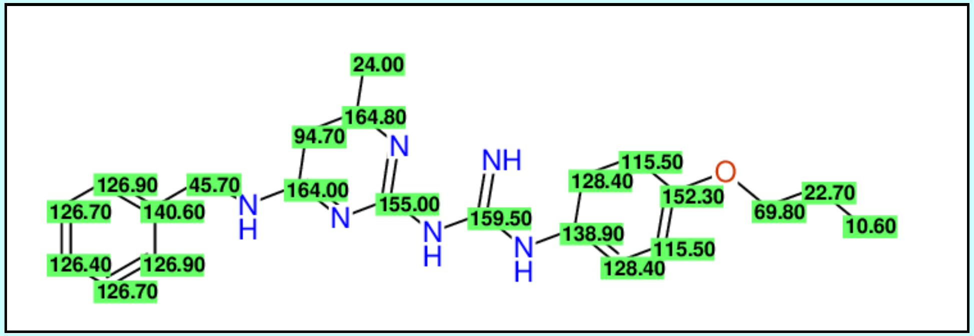

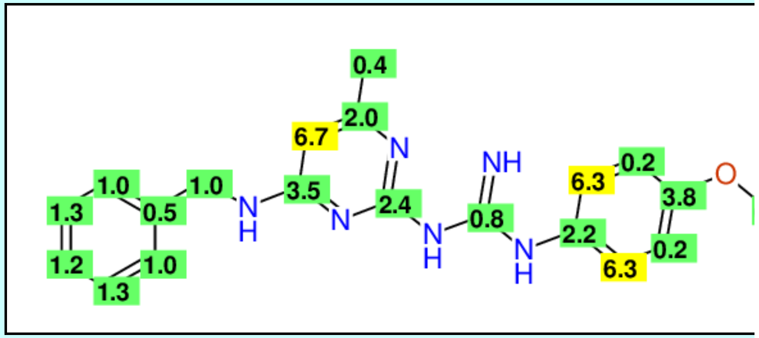

Nothing found when searching CSEARCH for identical structures

(Description)

No alternative structure found when searching CSEARCH for identical spectra

(Description)

Overall Impression

Poor Good

Minor revision might be necessary - please check

Compound: 1-[4-[Benzylamino_]-6-methylpyrimidin-2-yl_]-3-[4-propoxyphenyl_]guanidine_[

Project: Lengthening_the_Guanidine-Aryl_Linker_of_Phenylpyrimidinylguanidines_Increases_t

The CSEARCH Robot Referee recommends: Minor revision might be necessary - please check

[Check integrity of page via electronic fingerprint]

- NN-Prediction and HOSE-Code prediction differs significantly at 5 carbon positions
- Assignment can be probably improved at 9 positions
- 3 Carbon positions (out of 22) have a severe assignment problem
- Spectrum prediction - minor inconsistencies found

Experimental values

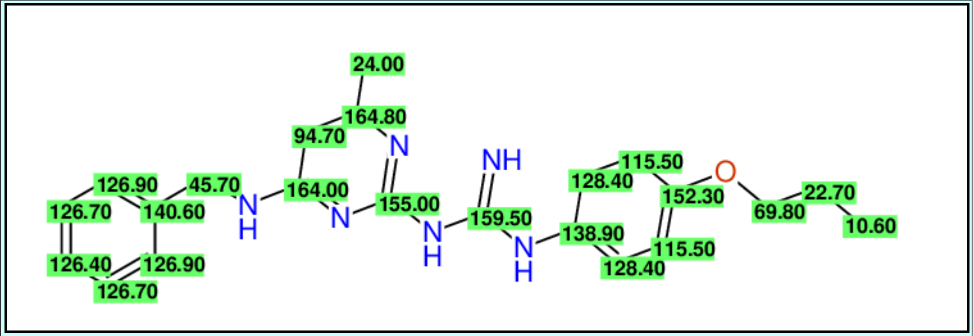

Predicted values

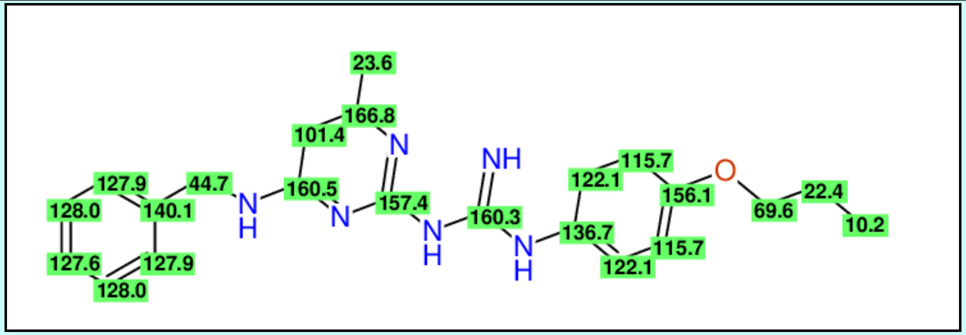

Matching map

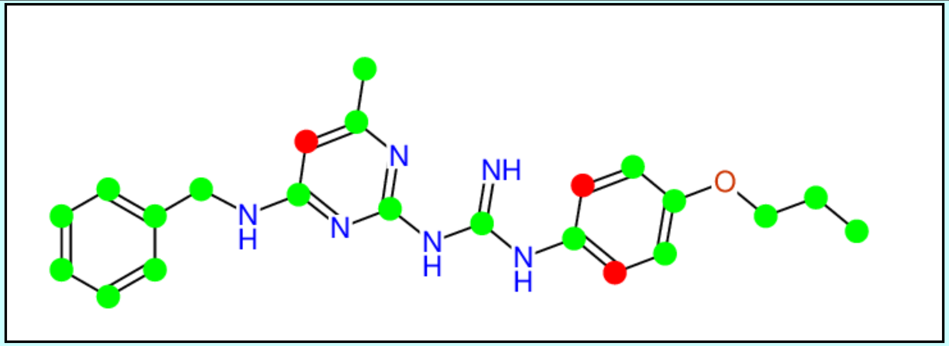

Deviation per position (Average is 1.9ppm)

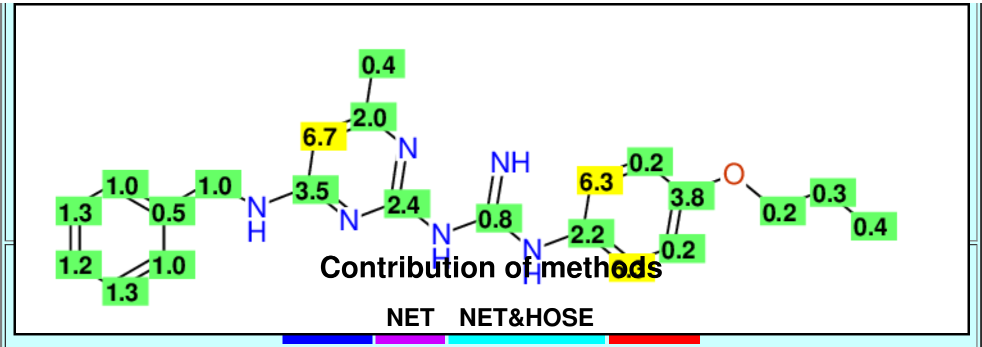

Contribution of methods

NET NET&HOSE

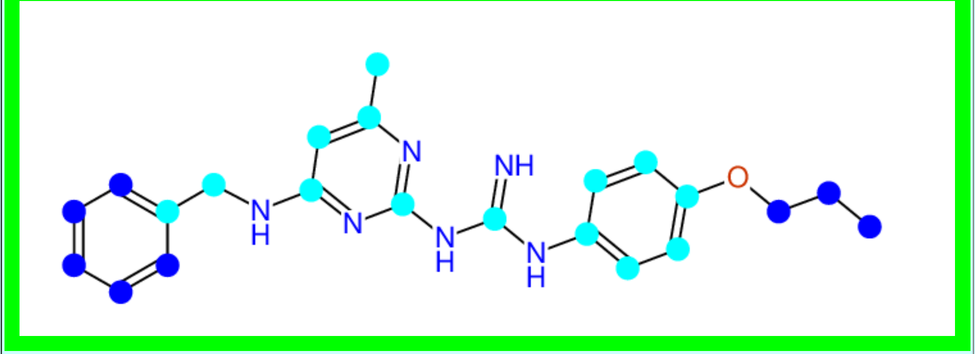

Overall Similarity Index is 2.0

0.0 is a "perfect match", up to approximately 3.0 it is "reasonable", above 5.0 it is more or less "unbelievable"

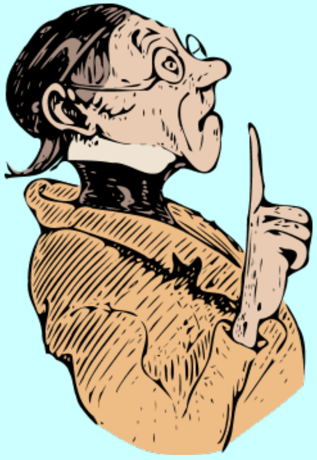

Your assignment	Difference to predicted values
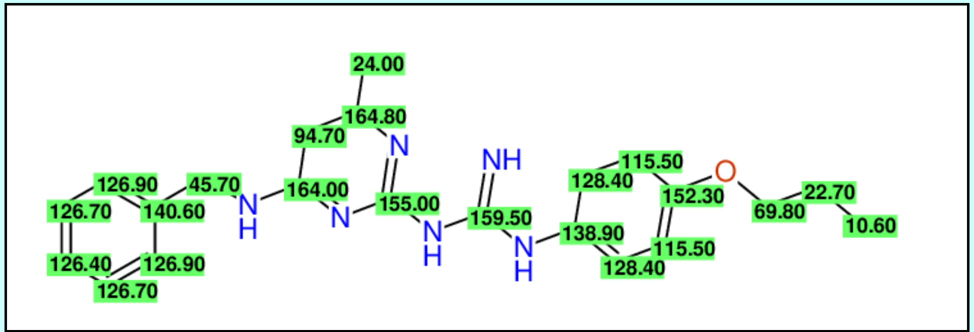	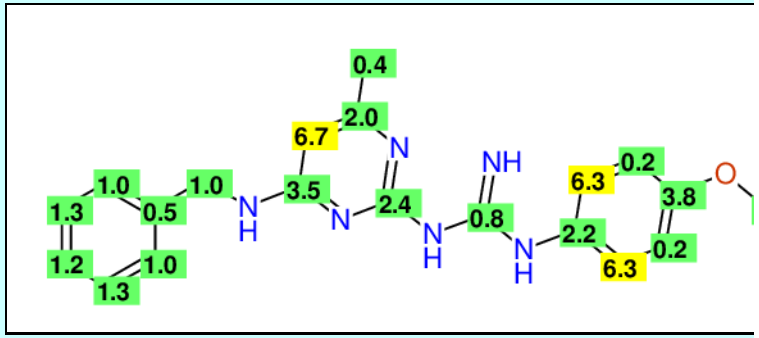

146,705,909 Compounds searched in PUBCHEM - nothing found

4,400,967 Compounds searched in Emolecules - nothing found

Search the Internet for this compound (Skeleton only)
Search the Internet for this compound (Skeleton + Stereochemistry)

Search CHEMSPIDER for this compound (Skeleton only)
Search CHEMSPIDER for this compound (Skeleton + Stereochemistry)

Search the Internet for the molecular formula $C_{22}H_{26}N_6O$

Search CHEMSPIDER for the molecular formula $C_{22}H_{26}N_6O$

(Description)

Your Total Usage of the CSEARCH-Robot-Referee

3 Requests have been launched by vojtech.docekal@natur.cuni.cz

Year	Accept	Minor Revision	Major Revision	Reject	Only Prediction
2022		1	2		

Top

Page has been automatically written by CSEARCH
CPU-Usage: Evaluation needed 8.392 seconds
Wolfgang.Robien(at)univie.ac.at
