# Supplementary material for: Lengthening the Guanidine–Aryl Linker of Phenylpyrimidinylguanidines Increases Their Potency as Inhibitors of FOXO3-Induced Gene Transcription
Source: ACS Omega. 2022 Sep 14;7(38):34632–46. doi: 10.1021/acsomega.2c04613 (PMC9521028; doi:10.1021/acsomega.2c04613)
Supplement: Supplementary file 2 — ao2c04613_si_002.zip [file ao2c04613_si_002.zip › 1-(4-chloro-6-methylpyrimidin-2-yl)-3-(4-propoxyphenyl)guanidine_(5ba).pdf]

Automatic Evaluation Report from CSEARCH
created on 2022-08-08 at 12:56:29
based on 340,554 reference spectra

Did you know ?

When entering signal multiplicities use only couplings to directly bound hydrogens.
Couplings to ^{19}F, ^{31}P or other nuclei have to be ignored when using multiplicity information,

Request from: vojtech.docekal@natur.cuni.cz

Compound: 1-[4-Chloro-6-methylpyrimidin-2-yl_]-3-[4-propoxyphenyl_]guanidine_[5ba_]

Project: Lengthening_the_Guanidine-Aryl_Linker_of_Phenylpyrimidinylguanidines_Increases_t

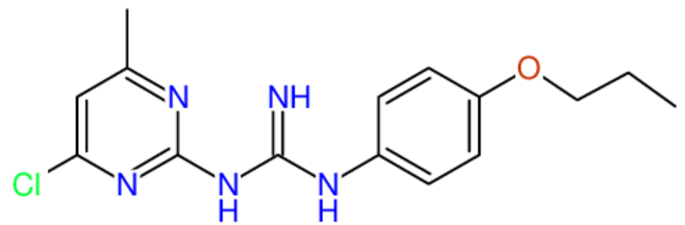

Database	Number of Entries	Owner of Database
Please cite the CSEARCH-Robot-Referee as: N. Haider, W. Robien; http://nmrpredict.orc.univie.ac.at/c13robot/robot.php		

CSEARCH	74,997 (A)	CSEARCH-Data / Wolfgang Robien
CSEARCH	56,549 (B)	CSEARCH-Data / Wolfgang Robien
CSEARCH	28,196 (C)	CSEARCH-Data / Wolfgang Robien
CSEARCH	33,587 (D)	CSEARCH-Data / Wolfgang Robien
CSEARCH	39,132 (E)	CSEARCH-Data / Wolfgang Robien + NMR-Database University of Mainz
CSEARCH	26,196 (F)	CSEARCH-Data / Wolfgang Robien
CSEARCH	50,594 (I)	Upcoming CSEARCH-Data / Wolfgang Robien
CSEARCH	31,307 (L)	NMRShiftDB-Data / Version February 2012

Permanent URL

This page can be verified by a digital signature

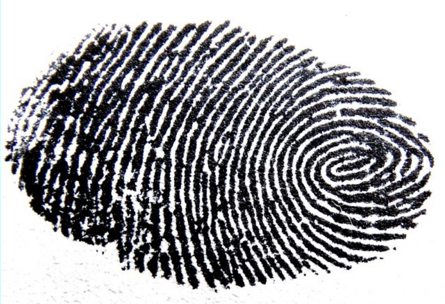

CSEARCH-Version: 9.4.0
Robot-Referee: 2017:06:10

Request from: vojtech.docekal@natur.cuni.cz

Compound: 1-[4-Chloro-6-methylpyrimidin-2-yl_]-3-[4-propoxyphenyl_]guanidine_[5ba_]

Project: Lengthening_the_Guanidine-Aryl_Linker_of_Phenylpyrimidinylguanidines_Increases_t

Recommendation given here

Details of Prediction given here

Summary of Supplied Data

Understanding the Color Coding Scheme

Structure Proposal

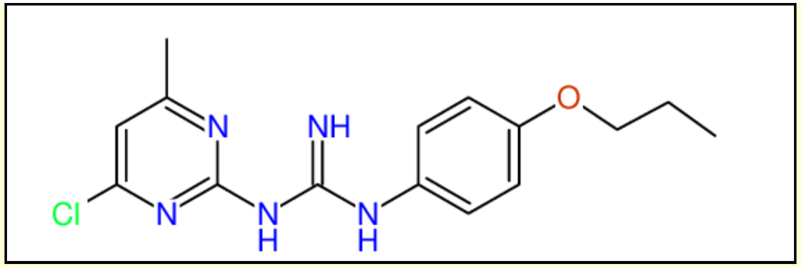

Molecular formula is: $C_{15}H_{18}ClN_5O$ Molecular weight is: 319.81 amu

INCHIKEY is: XSLDOYOEOVQZFT-UHFFFAOYAY

Numbering Scheme derived from the drawing sequence used during the calculation

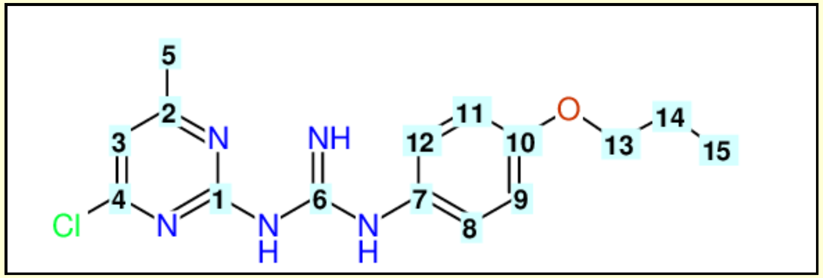

The marked carbons have been fully assigned

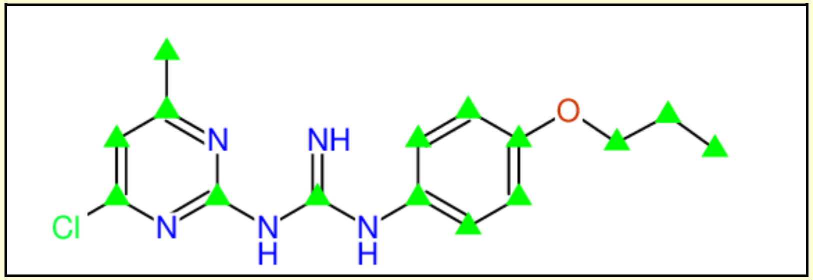

Carbon number	Chemical Shift Value	Multiplicity from Structure	Multiplicity from Experiment

1	157.00	S	-
2	171.40	S	-
3	116.00	D	-
4	161.00	S	-
5	23.70	Q	-
6	158.80	S	-
7	126.40	S	-
8	128.20	D	-
9	116.70	D	-
10	154.00	S	-
11	116.70	D	-
12	128.20	D	-
13	69.80	T	-
14	22.40	T	-
15	10.90	Q	-

The marked carbons have been fully assigned

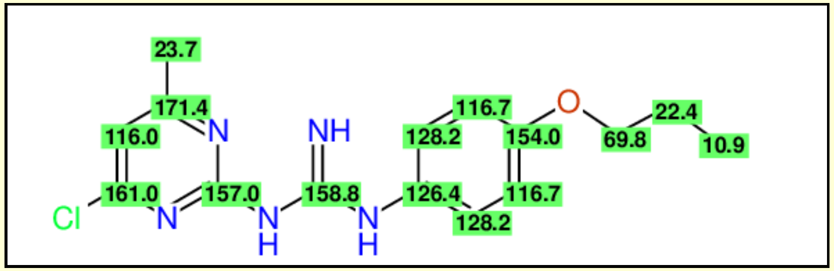

Graphical summary of the Chemical Shift Data

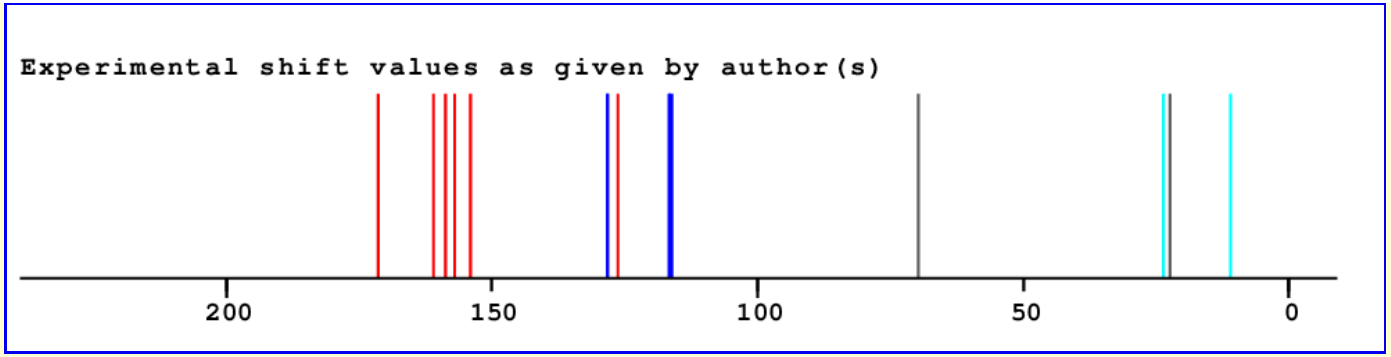

Searching external databases

146,705,909 Compounds searched in PUBCHEM - nothing found

4,400,967 Compounds searched in Emolecules - nothing found

Search the Internet for this compound (Skeleton only)
Search the Internet for this compound (Skeleton + Stereochemistry)

Search CHEMSPIDER for this compound (Skeleton only)
Search CHEMSPIDER for this compound (Skeleton + Stereochemistry)

Search the Internet for the molecular formula $C_{15}H_{18}ClN_5O$

Search CHEMSPIDER for the molecular formula $C_{15}H_{18}ClN_5O$

[(Description)]

Basic Evaluation: Checking Multiplicities

Checking lines & multiplicity	Carbons/Lines	Singlet	Dublet	Triplet	Quartet	Odd	Even	None
From structure	15	6	5	2	2	8	7	0
From spectrum	15	6	5	2	2	8	7	0

Overall impression on compatibility of multiplicity from structure and experiment

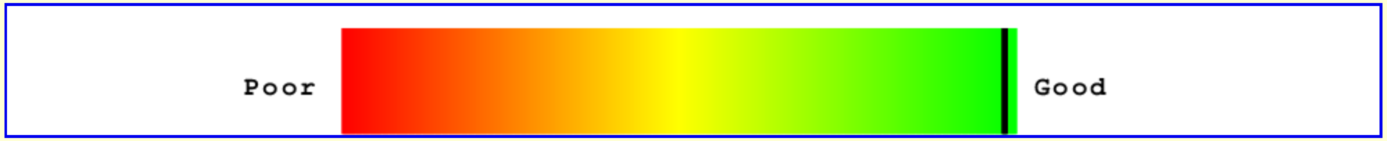

Evaluation based on Spectrum Prediction

Numbering Scheme

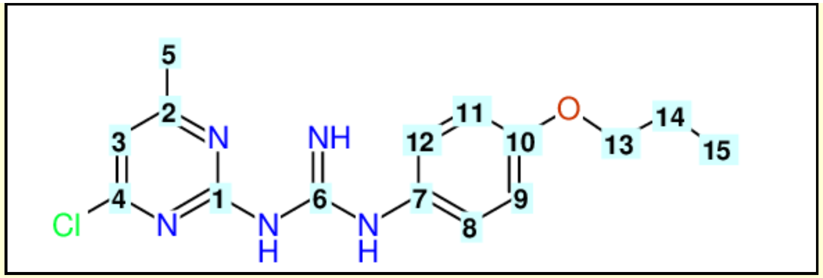

Carbon Number ▲▼	Neural Network Prediction ▲▼	HOSE-Code Prediction ▲▼	Preferred Value from both Predictions ▲▼	Experimental values ▲▼	Difference (Exp-Pred/ppm) ▲▼	Assignment	Prediction Quality
1	159.0	160.0	159.5	157.0	2.5	Assigned by author<br>Check assignment - maybe 158.80<br>?	Only reference material with low similarity
2	170.0	169.7	169.9	171.4	1.5	Assigned by author	
3	119.1	110.1	111.9	116.0	4.1	Assigned by author<br>Check assignment - maybe 116.70<br>?	Large Difference between NET & HOSE<br>Only very few similar structures
4	159.0	169.1	164.0	161.0	3.0	Assigned by author<br>Check assignment - maybe 154.00<br>?	Large Difference between NET & HOSE<br>Only very few similar structures
5	24.8	23.8	24.0	23.7	0.3	Assigned by author	
6	163.7	156.9	160.3	158.8	1.5	Assigned by author<br>Check assignment - maybe 161.00<br>?	Large Difference between NET & HOSE<br>Only reference material with low similarity<br>Only very few similar structures
7	134.8	138.7	136.7	126.4	10.3	Assigned by author<br>Check assignment - maybe 128.20<br>?	
8	122.0	122.2	122.1	128.2	6.1	Assigned by author<br>Check assignment - maybe 126.40<br>?	
9	115.1	115.8	115.7	116.7	1.0	Assigned by author<br>Check assignment - maybe 116.00<br>?	
10	153.0	159.2	156.1	154.0	2.1	Assigned by author<br>Check assignment - maybe 157.00<br>?	Large Difference between NET & HOSE
11	115.1	115.8	115.7	116.7	1.0	Assigned by author<br>Check assignment - maybe 116.00<br>?	

Carbon Number ▴▾	Neural Network Prediction ▴▾	HOSE Code Prediction ▴▾	Preferred Value from both Predictions ▴▾	Experimental values ▴▾	Difference (Exp-Pred/ppm) ▴▾	Assignment	Prediction Quality
12	122.0	122.2	122.1	128.2	6.1	Assigned by author<br>Check assignment - maybe 126.40 ?	
13	71.9	69.6	69.6	69.8	0.2	Assigned by author	
14	21.9	22.4	22.4	22.4	0.0	Assigned by author	
15	10.6	10.2	10.2	10.9	0.7	Assigned by author	
Absolute<br>Signed	2.82ppm (15)<br>-0.06ppm (15)	3.52ppm (15)<br>-0.29ppm (15)	2.70ppm (15)<br>0.07ppm (15)			2.51ppm (15)<br>0.07ppm (15)	Average deviation to experimental values<br>(Number of shift pairs used)
Structure representation by reference data over 3.5 shells on average							

Visualization of the differences between predicted and experimental values

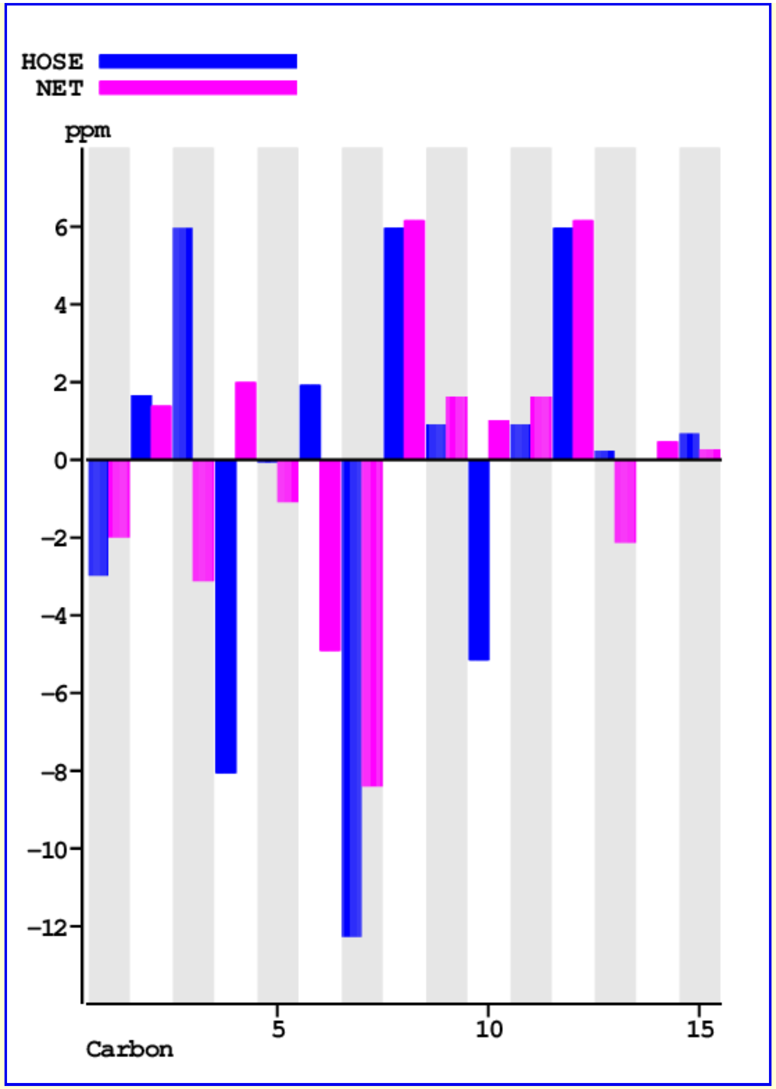

Quality of the Spectrum Prediction

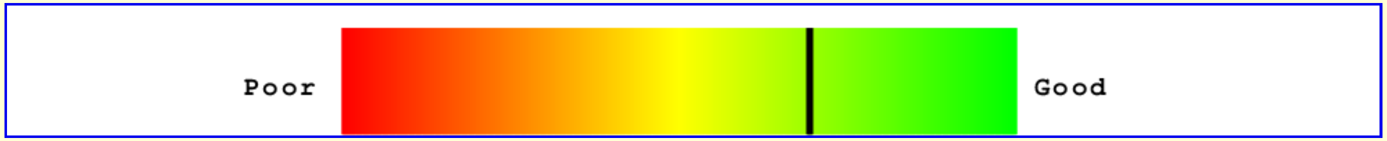

Experimental Chemical Shift Values as given

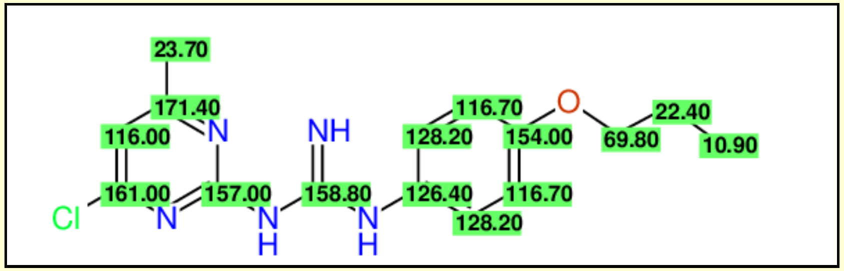

Experimental Chemical Shift Values using Symmetry

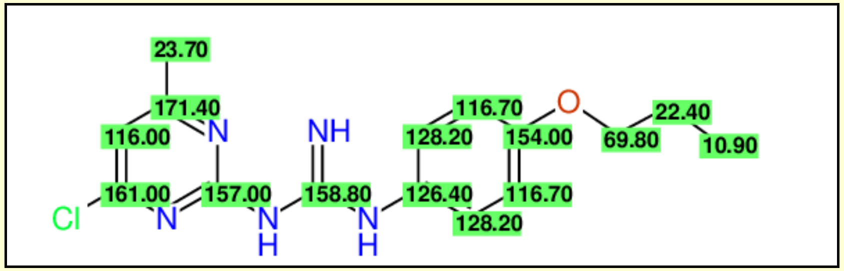

Preferred Chemical Shift Values from both predictions

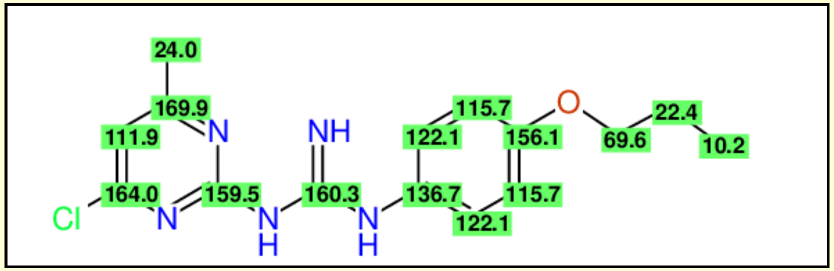

Comparison of Prediction Techniques

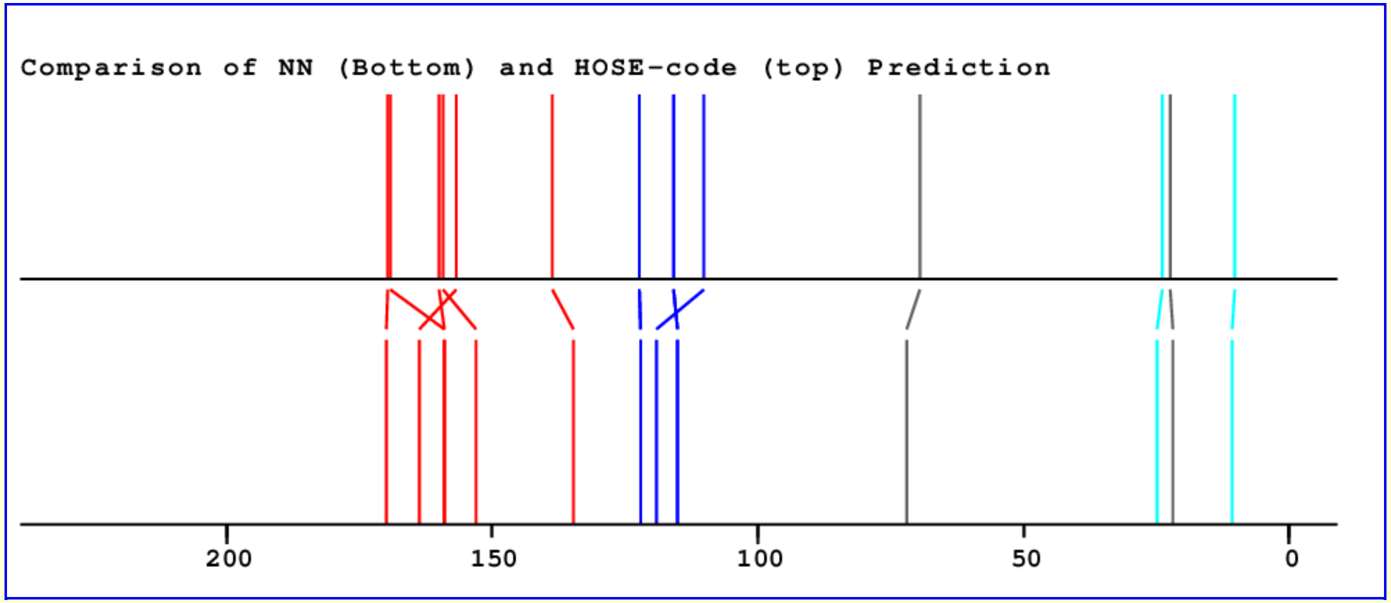

Contribution of the methods

HOSE | NET | NET&HOSE | NONE

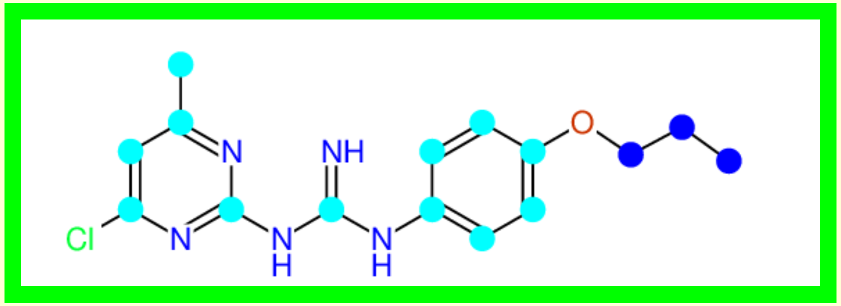

Similarity between predicted and experimental data based on positions

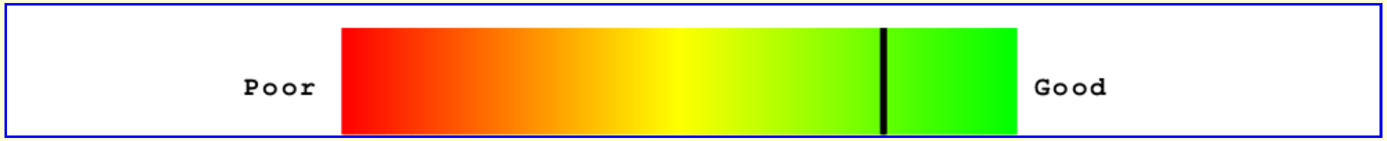

Matching map of predicted versus experimental data

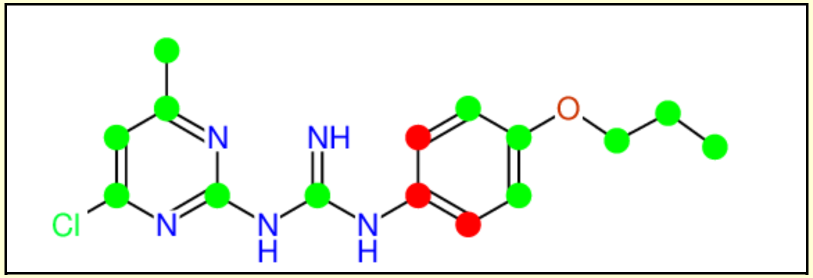

Differences between predicted and experimental data in ppm

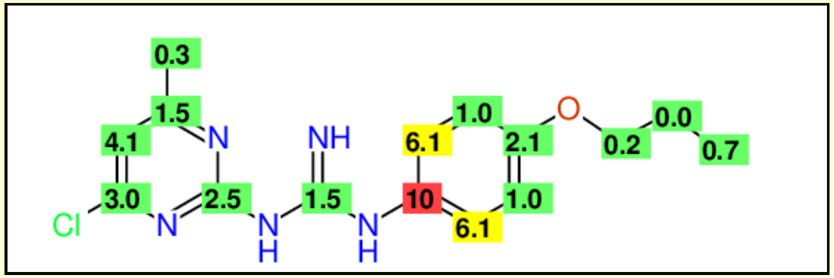

Comparison of Experimental versus Predicted Chemical Shift Values

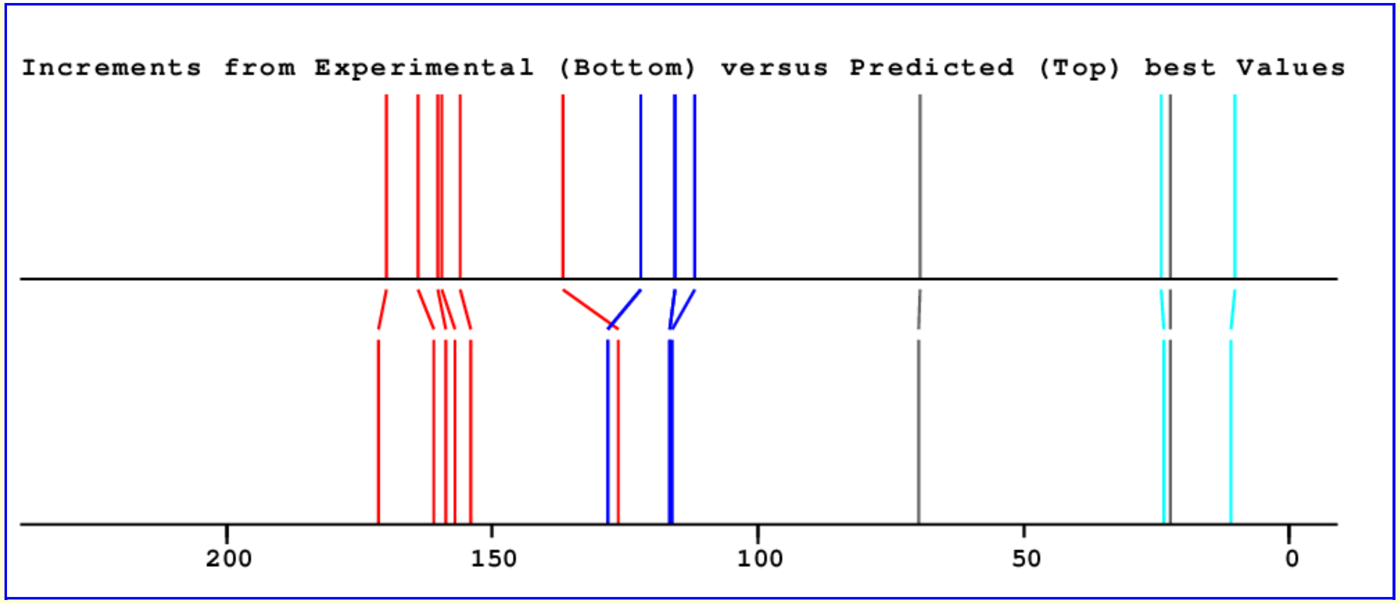

Overall deviation between predicted and experimental data is 2.7ppm

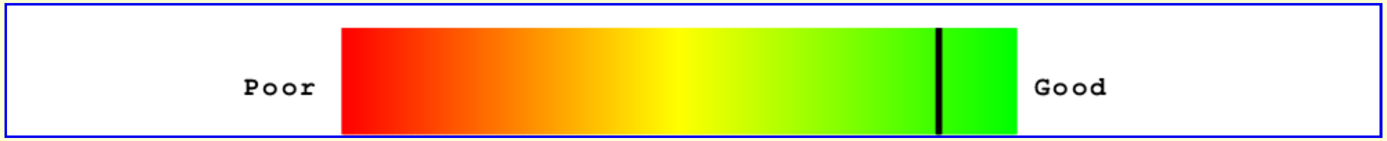

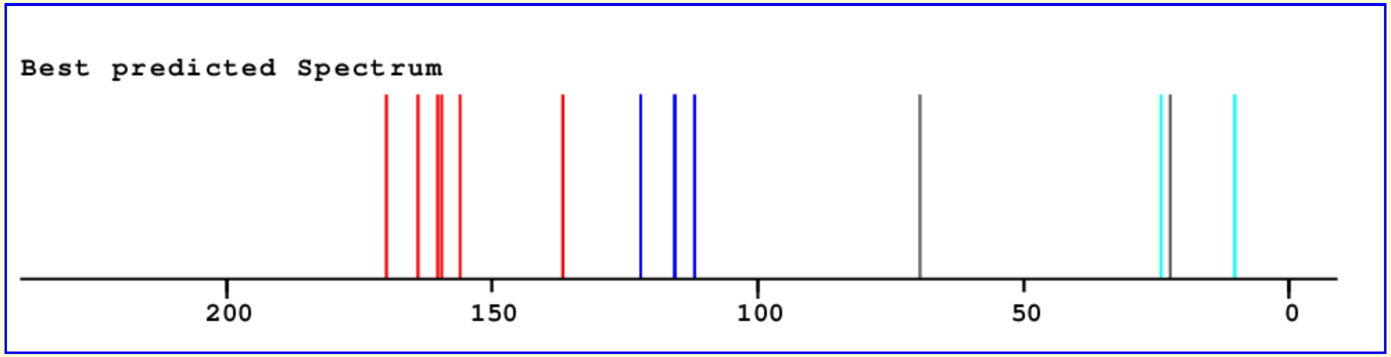

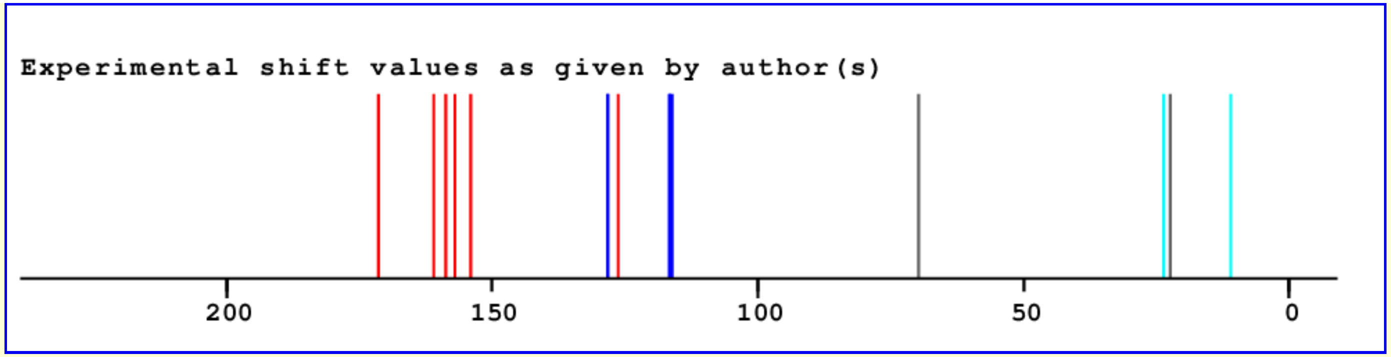

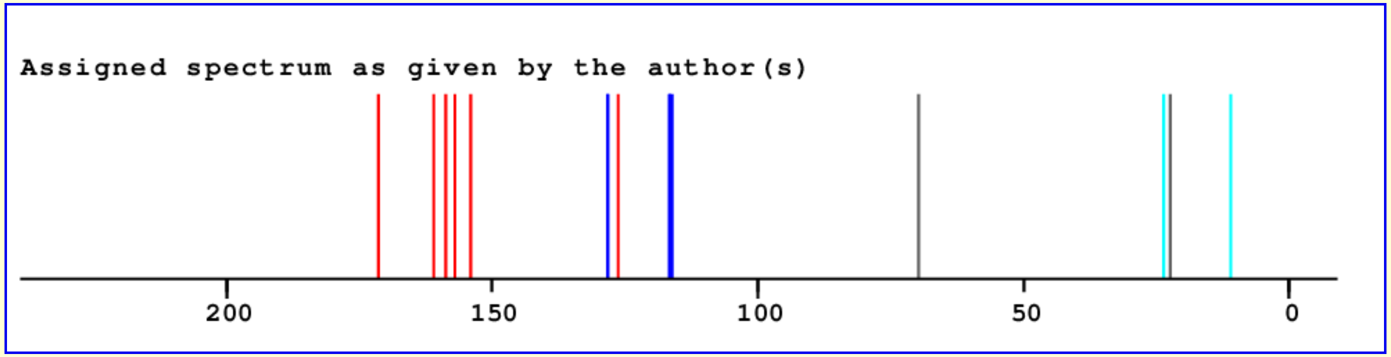

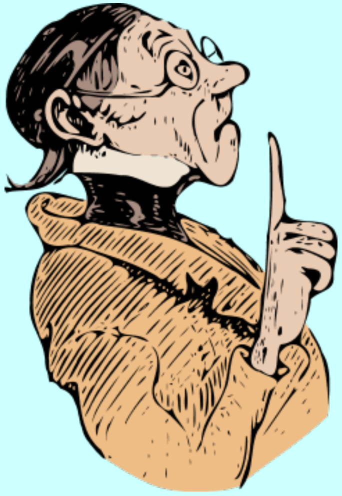

	Your assignment	Difference to predicted values
	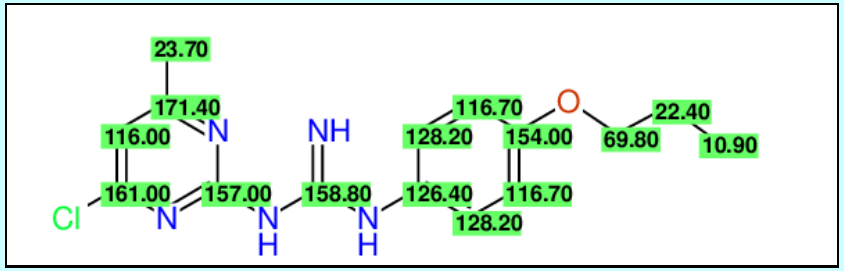	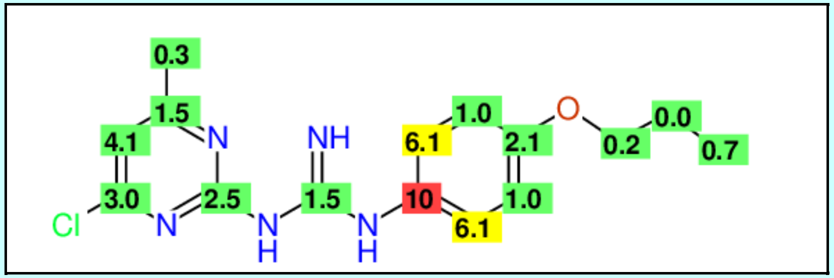

Nothing found when searching CSEARCH for identical structures

(Description)

No alternative structure found when searching CSEARCH for identical spectra

(Description)

Overall Impression

Poor Good

Minor revision might be necessary - please check

Compound: 1-[4-Chloro-6-methylpyrimidin-2-yl_]-3-[4-propoxyphenyl_]guanidine_[5ba_]

Project: Lengthening_the_Guanidine-Aryl_Linker_of_Phenylpyrimidinylguanidines_Increases_t

The CSEARCH Robot Referee recommends: Minor revision might be necessary - please check

[Check integrity of page via electronic fingerprint]

- NN-Prediction and HOSE-Code prediction differs significantly at 4 carbon positions
- Assignment can be probably improved at 10 positions
- 1 Experimental shift value differs more than 10ppm from prediction
- 3 Carbon positions (out of 15) have a severe assignment problem
- Spectrum prediction - minor inconsistencies found

Experimental values

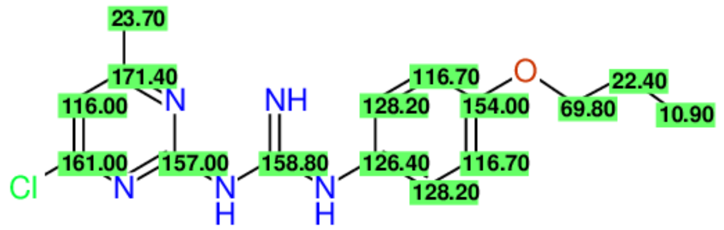

Predicted values

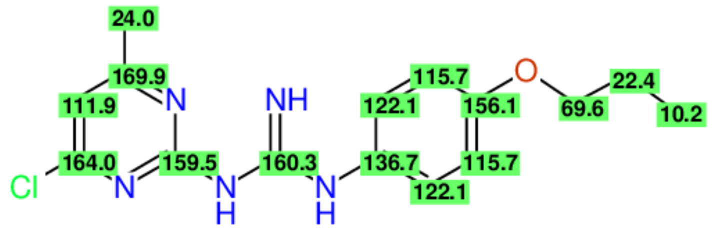

Matching map

Deviation per position (Average is 2.7ppm)

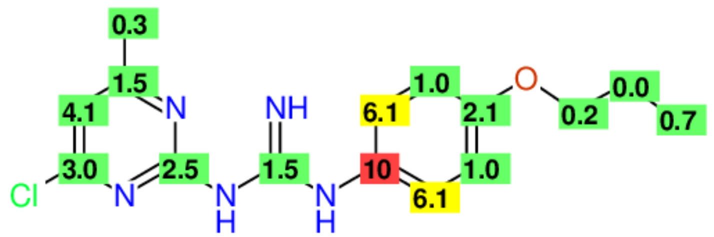

Contribution of methods

HOSE NET NET&HOSE NONE

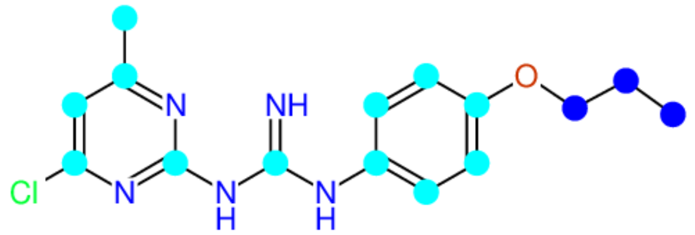

Overall Similarity Index is 2.7

0.0 is a "perfect match", up to approximately 3.0 it is "reasonable", above 5.0 it is more or less "unbelievable"

	Your assignment	Difference to predicted values

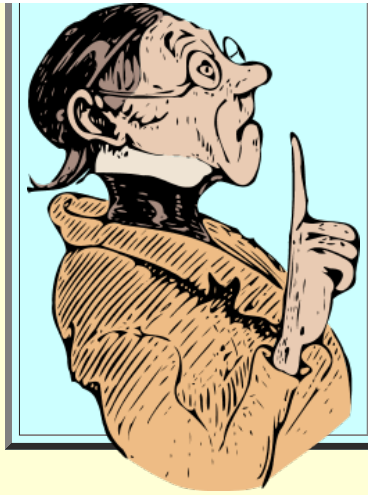

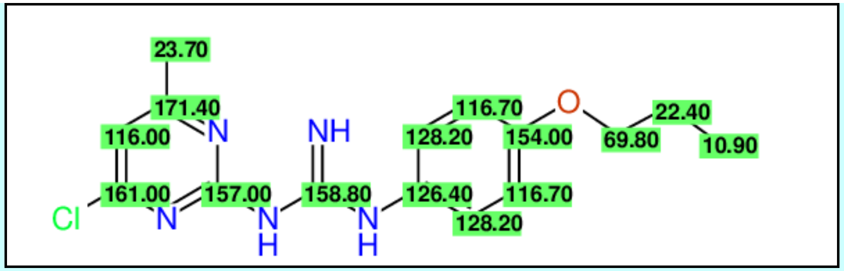

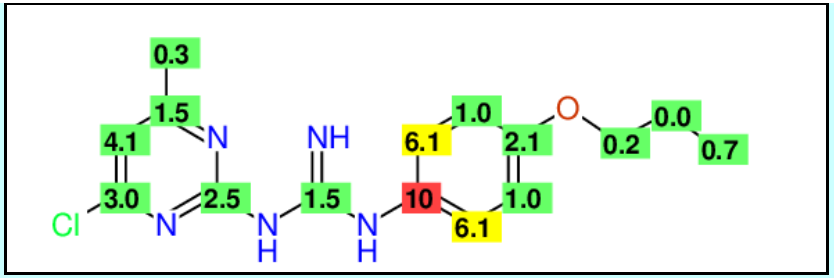

146,705,909 Compounds searched in PUBCHEM - nothing found

4,400,967 Compounds searched in Emolecules - nothing found

Search the Internet for this compound (Skeleton only)
Search the Internet for this compound (Skeleton + Stereochemistry)

Search CHEMSPIDER for this compound (Skeleton only)
Search CHEMSPIDER for this compound (Skeleton + Stereochemistry)

Search the Internet for the molecular formula $C_{15}H_{18}ClN_5O$

Search CHEMSPIDER for the molecular formula $C_{15}H_{18}ClN_5O$

(Description)

Your Total Usage of the CSEARCH-Robot-Referee

2 Requests have been launched by vojtech.docekal@natur.cuni.cz					
Year	Accept	Minor Revision	Major Revision	Reject	Only Prediction
2022			2		

Page has been automatically written by CSEARCH
CPU-Usage: Evaluation needed 8.236 seconds
Wolfgang.Robien(at)univie.ac.at
